# A miRNA catalogue and ncRNA annotation of the short-living fish *Nothobranchius furzeri*

**DOI:** 10.1186/s12864-017-3951-8

**Published:** 2017-09-05

**Authors:** Mario Baumgart, Emanuel Barth, Aurora Savino, Marco Groth, Philipp Koch, Andreas Petzold, Ivan Arisi, Matthias Platzer, Manja Marz, Alessandro Cellerino

**Affiliations:** 1 Leibniz Institute for Age Research - Fritz Lipmann Institute (FLI), Beutenbergstraße 11, 07745 Jena, Germany; 2 Bioinformatics/High Throughput Analysis, Friedrich Schiller University Jena, Leutragraben 1, 07743 Jena, Germany; 3 Laboratory of Biology, Scuola Normale Superiore, 56126 Pisa, Italy; 4 Babraham Institute, Cambridge, England; 5 Dresden University of Technology, Dresden, Germany; 6 European Brain Research Institute (EBRI), Rome, Italy

**Keywords:** miRNome, Fish miRNA evolution, *Nothobranchius furzeri*, ncRNA

## Abstract

**Background:**

The short-lived fish *Nothobranchius furzeri* is the shortest-lived vertebrate that can be cultured in captivity and was recently established as a model organism for aging research. Small non-coding RNAs, especially miRNAs, are implicated in age dependent control of gene expression.

**Results:**

Here, we present a comprehensive catalogue of miRNAs and several other non-coding RNA classes (ncRNAs) for *Nothobranchius furzeri*. Analyzing multiple small RNA-Seq libraries, we show most of these identified miRNAs are expressed in at least one of seven *Nothobranchius* species. Additionally, duplication and clustering of *N. furzeri* miRNAs was analyzed and compared to the four fish species *Danio rerio*, *Oryzias latipes*, *Gasterosteus aculeatus* and *Takifugu rubripes*. A peculiar characteristic of *N. furzeri*, as compared to other teleosts, was a duplication of the miR-29 cluster.

**Conclusion:**

The completeness of the catalogue we provide is comparable to that of the zebrafish. This catalogue represents a basis to investigate the role of miRNAs in aging and development in this species.

**Electronic supplementary material:**

The online version of this article (doi:10.1186/s12864-017-3951-8) contains supplementary material, which is available to authorized users.

## Background

The annual teleost *Nothobranchius furzeri* is a recent experimental animal model in biomedical research. In the wild, this fish inhabits ephemeral pools in semi-arid *bushveld* of Southern Mozambique. It has adapted to the seasonal drying of its natural environment by producing desiccation-resistant eggs, which can remain dormant in the dry mud for one and maybe more years by entering into diapause. Due to the very short duration of the rainy season in its habitat, the natural lifespan of these animals is limited to a few months. They represent the vertebrate species with the shortest captive lifespan of only 4–12 months and also with the fastest maturation. In addition, they express a series of conserved aging markers and are amenable to genetic manipulations, making them an attractive model system for aging research (for a review, see [11, 49]). A striking characteristic of *N. furzeri* is the existence of laboratory strains differing in lifespan and expression of aging phenotypes [15, 60]: an extremely short-lived strain (GRZ: median lifespan 3–4 months) and several longer-lived strains (e.g., MZM-04/10; median lifespan 7–9 months). The molecular basis for this striking difference in aging is unknown. A previous miRNA-Seq study of brain aging that predated genome sequencing and used homology to miRBase to annotate *N. furzeri* miRNAs revealed that the two strains have different global patterns of miRNA expression [2].

Here, we provide a comprehensive microRNA (miRNA) catalogue for *N. furzeri*. MiRNAs are abundant non-coding RNAs between 18 and 24 nucleotides in length that are produced in a complex biosynthetic process starting from longer transcripts and are established as key players in the post-transcriptional regulation of gene expression. MiRNA genes can be hosted within an intron of a protein-coding gene (and their transcriptional regulation follows that of the hosting gene) or can arise from primary transcripts that are regulated independently of any protein-coding RNA. Several miRNAs are grouped in genomic clusters containing mostly two to six individual miRNAs with an intra-miRNA distance of less than 10 kb, which are co-transcribed. However, unusually large clusters were also found in some species, like the miR-430 cluster in zebrafish, consisting of 57 miRNAs [41, 61, 68]. The advantage of this accumulation is unclear. It could be possible that multiple loci are required to increase the copy-number and therefore the expression level of specific miRNAs in particular conditions, like miR-430 in the maternal-zygotic transition in zebrafish (*Danio rerio*) [19]. MiRNA genes are grouped into families based on sequence homology and can be defined as a collection of miRNAs that are derived from a common ancestor [20]. On the contrary, miRNA clusters may contain miRNAs belonging to different miRNA families, but are located in relative close proximity to each other. Both the evolutionary conservation of some miRNA families and the innovations leading to appearance of novel miRNAs are well-described. An expansion of the miRNA inventory due to genome duplications in early vertebrates and in ancestral teleosts has already been described [24].

MiRNAs bind target mRNAs, due to sequence complementarity in the seed region (nucleotides 2–7), mostly in the 3′ untranslated region, thereby silencing expression of the gene product via translational repression and/or transcript degradation. Up to now, several thousands of miRNAs have been predicted and identified in animals, plants and viruses, and one single species can express more than one thousand miRNAs [21]. They frequently represent the central nodes of regulatory networks and may act as “rheostat” to provide stability and fine-tuning to gene expression networks [47, 53]. Before a sequence of the *N. furzeri* genome assembly became available [50], we could show by use of the *Danio rerio* reference from miRBase that aging in the *N. furzeri* brain displays evolutionary conserved miRNA regulation, converging in a regulatory network centred on the antagonistic actions of the oncogenic MYC and tumor-suppressor TP53 [2], and the expression of miR-15a and the miR-17/92 cluster is mainly localized in neurogenetic regions of the adult brain [10]. Two draft genome sequences for *N. furzeri* were recently produced [50, 67]. In this paper, we now provide a comprehensive annotation of the *N. furzeri* miRNome based on a combination of Illumina-based small RNA-Seq data, different in silico prediction methods on the genome assembly and a final manual curation. Using the newly created miRNA reference, we analyzed a large dataset of 162 small RNA-Seq libraries and report tissue-specific miRNA expression of conserved and non-conserved miRNAs in *N. furzeri*. We further used the *N. furzeri* reference to analyze the miRNA expression in other Nothobranchius species and one closely-related non-annual killifish species, which were previously used to analyze positive selection [50] to identify when in the evolutionary history of *N. furzeri* non-conserved miRNAs arose.

## Results and discussion

### Small RNA-Seq libraries

For this study, 150 small RNA-Seq libraries of *N. furzeri* from different ages and tissues were sequenced, making a total of around 2.2 billion reads. In more detail, we had 75 libraries for both of the strains *N. furzeri MZM-0410* (in the following called MZM) and *N. furzeri GRZ*. We investigated the three tissues brain, liver and skin at five different ages in *N. furzeri GRZ* (5, 7, 10, 12, 14 weeks) and *N. furzeri MZM* (5, 12, 20, 27, 39 weeks) with five biological replicates each. The only exception are the *N. furzeri MZM* brain libraries, where we have four biological replicates for each age, but an additional time point of 32 weeks with five replicates. Additionally, seven embryonic small RNA-Seq libraries of *N. furzeri* were sequenced (two of the strain *MZM-0403* and five of *MZM-0410*). After pre-processing all libraries, a total of around 1.9 billion high-quality reads was used for further analysis (see Methods section and Supplement Table 1).

**Table 1:** Number of annotated ncRNAs

ncRNA class	No.	ncRNA class	No.
18S rRNA	3	7SK	1
5.8S rRNA	11	Vault	12
28S rRNA	6	TPP	1
5S rRNA	28	IRE^a^	3
tRNA	570	CAESAR^a^	1
U1	6	DPB^a^	1
U2	8	Vimentin3^b^	1
U4	8	Antizyme FSE^c^	2
U5	8	U1A PIE^c^	1
U6	6	KRES^c^	26
U11	1	GABA3^c^	6
U12	1	Y RNA	4
U4atac	1	TERC	1
U6atac	1	mascRNA-menRNA^d^	42
RNase P	1	SPYR-IT1^d^	1
RNase MRP	1	MEG8^d^	1
SRP	3		

Besides housekeeping RNAs, RNA elements controlling metabolism and protein editing were identified. ^a^mRNA regulation RNA element, ^b^mRNA localization RNA element, ^c^editing signal, ^d^conserved lncRNA element

For each of the six killifish species, *A. striatum, N. kadleci, N. rachovii, N. pienaari, N. kunthae* and *N. korthausae*, we generated two biological replicates of small RNA-Seq libraries from the brain of mature animals. The average size per library was 24.5 million reads with a minimum of 16.8 million and a maximum of 36.1 million reads (for more details about the small RNA-Seq libraries, see Supplement Table 1).

### Annotation of ncRNAs

We could identify more than 750 non-coding RNA (ncRNA) genes in the *N. furzeri* genome based on small RNA-Seq reads, including editing signals, RNA elements located in the UTRs of mRNAs either controlling localization or regulation and conserved lncRNA element (see Table 1, Additional file 1 and Supplement Table 5). In line with other eukaryotes, we identified multiple gene copies of rRNAs, tRNAs, several major spliceosomal RNAs, signal recognition particle (SRP) RNAs and one copy of a minor spliceosomal RNA set. Further housekeeping RNA genes, such as RNase P, RNase MRP, and the 7SK RNA, are found, as expected, once in the entire genome. We annotated the widely distributed TPP riboswitch, capable of binding thiamine pyrophosphate and thereby regulating genes that are in charge of the thiamine balance. We could also identify more RNA elements located in the UTRs of mRNAs, being directly involved in the regulation of gene expression (3 copies of *IRE* – controlling iron responsive proteins, CAESAR – controlling tissue growth factor CTGF, DPB – controlling DNA polymerase β), localization of mRNAs (Vimentin3), DNA replication (four copies of the *Y RNA* gene, and Telomerase RNA TERC) or of unknown function (12 vault RNAs). Additionally, ncRNAs responsible for editing certain mRNAs have also been found (two copies of Antizyme FSE, one U1A polyadenylation inhibition element (PIE), 26 Potassium channel RNA editing signals (KRES), and six copies of *GABA3*). Two promising candidate long non-coding RNAs (lncRNAs), SPYR-IT1 and MEG8, were also included in the annotation, even though we were not able to identify all of their exons. Two vague candidates for XIST and MALAT can be viewed in the supplemental material. The MALAT-derived masc and men RNA gene was clearly detected in 42 copies throughout the genome of *N. furzeri*.

### Mapping and miRNA prediction results

**Fig. 1:**
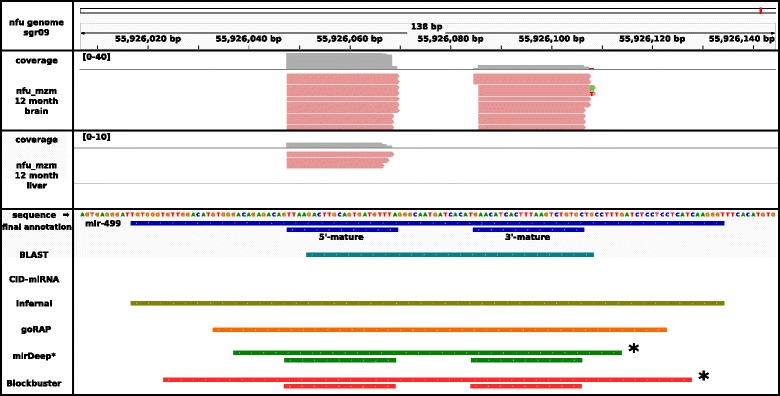
A three-dimensional PCA plot of the *N. furzeri MZM* small RNA-Seq libraries of all three tissues (brain – *red*, liver – *green*, *blue* – skin) and all investigated ages (from light to dark: 5, 12, 20, 27, 39 weeks). Whereas the samples cluster well according to their tissue belongings, a distinct separation regarding the ages can only be observed for the youngest samples in each tissue. A PCA plot of the *GRZ* strain, can be found in Supplement Table 2

For the identification of putative miRNA genes, we used five methods, each following a different prediction approach (BLAST, CID-miRNA, Infernal, GoRAP, miRDeep*) and *Blockbuster* as verification (see Fig. 1 for an example).

**Fig. 2:**
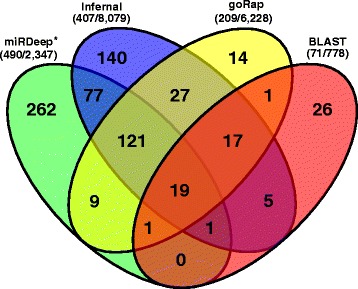
Annotation, expression profiles and prediction comparison for miR-499.We annotated the pre-miR-499 on sgr09, position 55,926,017–55,926,134 and the two mature miRNAs at 55,926,048–55,926,069 and 55,926,085–55,926,106. The six methods used for miRNA detection are displayed, *CID-miRNA* was not able to detect this miRNA. Tools working independent of the small RNA-Seq data *BLAST* (*cyan*), *Infernal* (*olive green*) and *goRAP* (*orange*) vary in their annotation length. The latter two programs are based on covariance models, identifying mostly the complete pre-miRNA. The remaining two programs miRDeep* and *Blockbuster* are based on small RNA-Seq data (*) and therefore accurately annotate the mature miRNAs. MiR-499 is expressed weakly within *N. furzeri MZM* 12 month liver library and therefore could not be detected by miRDeep* and *Blockbuster*. In the *N. furzeri MZM* 12 month brain library, miR-499 was expressed strongs enough to be detected by both programs

The five tools identified 71, 33, 407, 209, 490 miRNA candidates, respectively (Fig. 2 shows the variety and the overlap of the different tools). All predictions were merged, and redundant loci were removed (for details, see Methods section). Of the remaining 788 candidate miRNAs, 617 (78.3%) showed expressions and were verified by *Blockbuster* and then manually verified for the correct mapping of the reads on the predicted precursor, with rectencular peaks corresponding to the mature 5p and/or 3p separated by a short gap devoid of mapped reads, while cases with more extended mappings were excluded. By this, 34 (4.3%) candidates were removed, all predicted by miRDeep*. Candidates showing no expression in any of the sequenced small RNA-Seq libraries were still kept as putative miRNAs, since they were predicted based on conserved and already characterized miRNA genes. In total, we predict a final amount of 754 miRNAs in *N. furzeri* by union of these methods (see Additional file 2).

Most of the small RNA-Seq reads (up to 88.81%) mapped onto the identified 754 miRNAs. Interestingly, the number of miRNA related reads varies broadly between the tissue samples (see Table 2). Possibly, this difference correlates with different regenerative capacities of these tissues. Mature brain cells are hardly proliferating, whereas liver cells are constantly renewed [17, 43, 58]. This regeneration might be additionally under the control of certain yet unknown tissue specific miRNAs. About half of the miRNA annotations are overlapping genes coding for proteins and are therefore intragenic. A minor fraction of reads (see Table 2) maps to other ncRNAs and proteins. Whereas 333 of the predicted miRNAs can be assigned to one of the known miRBase families, based on sequence identity, 421 miRNAs did not match any known family and can therefore be considered as novel or non-conserved miRNAs (for details see Table 3).

**Table 2:** Total number of genes known in *N. furzeri* and number of ncRNAs covered by small RNA-Seq reads and percental distribution of reads for brain, skin and liver of *N. furzeri*. See Supplement Table 1 for more details and Table 4 for details about the read libraries

	Total	Brain		Skin		Liver	
miRNA	754	470	88.81%	438	75.01%	368	58.10%
tRNA	570	293	0.35%	361	1.33%	361	4.27%
rRNA	48	37	1.73%	43	3.61%	43	6.97%
ncRNA	250	136	0.09%	217	0.15%	204	0.41%
protein*	22,627	0	6.60%	0	4.59%	0	6.35%
none			2.42%		15.31%		23.90%
sum	24,125	936	100%	1059	100%	976	100%

Asterisks indicates annotation of protein-coding genes from NCBI. ncRNA – all ncRNAs except miRNA, tRNA and rRNA; none – reads mapping to non-annotated genomic areas

**Table 3:** Amount of annotated miRNAs, identified miRNA clusters and the number of miRNAs in clusters, as well as known conserved and non-conserved miRNA families in *N. furzeri* (Nfu), *D. rerio* (Dre), *O. latipes* (Ola), *G. aculeatus* (Gac) and *T. rubripes* (Fru)

Species	#miRNAs	#miRNA clusters	#miRNAs in clusters	#mirBase families	#unknown familes
nfu	754	83	213	94 (333)	383 (421)
dre	765	96	305	99 (307)	302 (458)
ola	366	58	151	104 (269)	55 (97)
gac	504	68	299	102 (413)	63 (91)
fru	337	59	143	99 (278)	51 (59)

In brackets, the amount of miRNAs associated to the identified miRNA families are given. For detailed lists of miRNA family assignments, see Supplement Table 4

The age-dependent expression of the following miRNAs was previously demonstrated by qPCR: tni-miR-15a, tni-miR-101a, tni-miR-101b, dre-miR-145, hsa-miR 29c-1 (100% identical to dre-miR-29a), hsa-let-7a-5p, hsa-miR-124a-1, hsa-miR-1-2, olamiR-21, ola-miR-183-5p and, from cluster dre-miR-17a/18a/19a, and dre-miR-20a (the used primers were Qiagen miScript primer). Expression changes detected by sequencing were validated on an independent set of specimens. All 13 miRNAs showed concordant changes in their expression, of which six reached statistical significance [2]. The expression of the following miRNAs in the brain was confirmed by in situ hybridisation using LNA probes (Exiqon): miR-9, miR-124 [63] and miR-15a, miR-20a [64].

### Target prediction of the miRNA candidates

In order to get a first insight of the potential regulatory functions of our putative miRNA genes, we performed a target prediction based on the miRNA seed regions and the aligned homologous 3′-UTR mRNA regions of *N. furzeri* and *D. rerio*. Additionally, we repeated this target prediction analysis, including homologous 3′- UTR mRNA regions of *M. musculus* and *H. sapiens* to have a more conservative target list for each miRNA candidate, since in silico miRNA target predictions tend to have a high number of false positive results [48]. Using only the two fish 3′-UTR alignments, we predicted for 438 of our miRNA candidates potential mRNA targets with a median of 47 putative targets per miRNA. With our more conservative approach, still 419 miRNA candidates showed targeting potential with a median of 25 putative targets per miRNA. To further examine these potential targets, we calculated enrichment scores of miRNA binding sites in already known sets of down-regulated genes in the brain of *N. furzeri* during aging [3] and in different tissues between young and very old *N. furzeri* individuals [50]. In the first study, both clusters, containing genes with decreasing activity during aging, show a significant enrichment of miRNA targets (cluster1: *p* = 8.67^−25^; cluster5: *p* = 1.78^−5^). For all three investigated tissues in the second study, we also found a significant enrichment of miRNA target sites within the downregulated genes (brain: *p* = 6.19^−32^; liver: *p* = 7.72^−17^; skin: *p* = 1.49^−9^). Additionally, we identified single miRNA candidates, whose targets were enriched in any of the above-mentioned gene sets (for details, see online supplement section *miRNA target prediction*). We found e.g., *miR-10*, *miR-29* and *miR-92* showing potential to be significantly involved in the down-regulation of genes in the aging brain of *N. furzeri*, like cell cycle regulators (*ccne2* [22], *nek6* [38], *cdk13* [42]) or cancer related genes (*mycn* [8, 12], *vav2* [13, 28]), both processes involved in aging.

### Effects of tissue and age on global miRNA expression

**Fig. 3:**
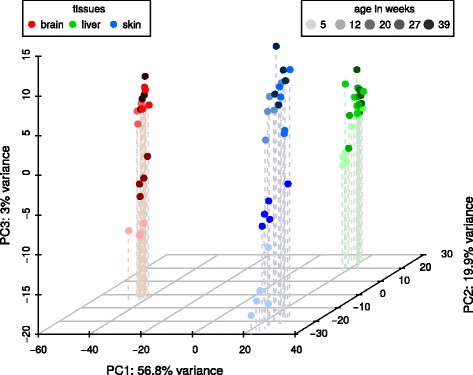
Venn diagram of predicted miRNA genes from four tools miRDeep*, *Infernal*, *goRap* and *BLAST*. Only 2 of the 33 candidates predicted by *CID-miRNA* overlapped with any of the other miRNA candidates. Nevertheless, all 33 candidates were selected as miRNAs after manual inspectations. The total number of miRNA predictions after and before applying any filtering step are shown in *brackets* for each tool

We used principal component analysis (PCA) to visualize the effects of tissue type and age on the global miRNA expression (see Fig. 3). A strong component of tissue-specific expression was detected and samples corresponding to different tissues clustered tightly and widely apart in the plane defined by the first two principal component axes (collectively accounting for 77% of variance). Remarkably, the third principal component axis (3% of variance explained) identifies an age-dependent component of miRNA expression that is common to all three tissues with the youngest samples (5 weeks), clearly separated from the rest. A detailed analysis of age- and tissue-dependent miRNA expression, including embryonic development, will be part of a separate publication.

### miRNA expression in closely related killifish

**Fig. 4:**
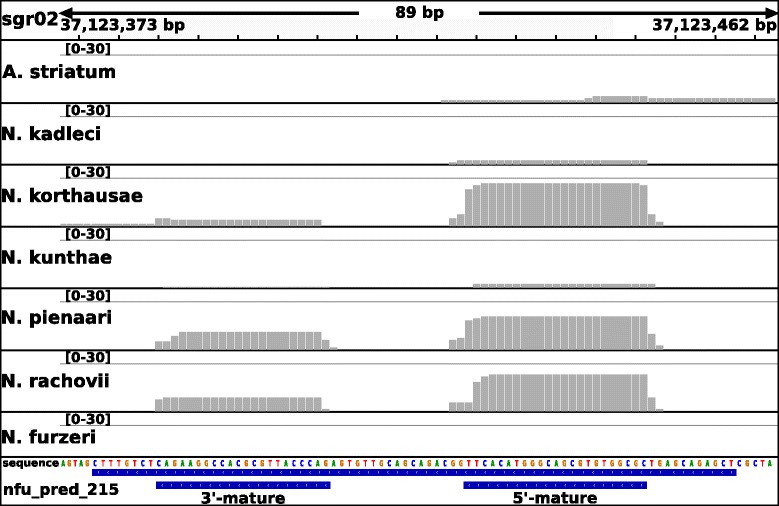
Expression profiles of the predicted miR-215. Gray bars indicate the number of aligned reads and therefore coverage at the specific positions. Whereas no expression can be observed for this miRNA in *N. furzeri*, clear activation can be seen in *N. korthausae*, *N. pienaari* and *N. rachovii*. *A. striatum*, *N. kadleci* and *N. kunthae* show a weak expression for at least the 5′-mature variant of this miRNA

**Fig. 5:**
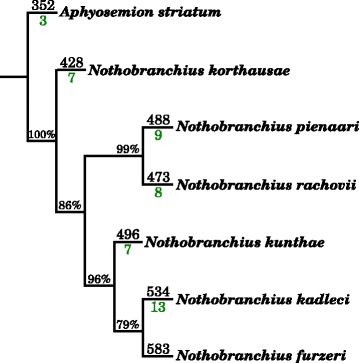
Killifish phylogeny based on the expressed miRNAs calculated via hierarchical clustering using the R package *pvclust* [55]. Bootstrap values are given as percentages at the corresponding branches. The amount of identified expressed miRNAs is given next to the species names. The numbers in *green* indicate the number of these expressed miRNAs, which were annotated but not expressed in any of the sequenced *N. furzeri* samples

To compare and validate the miRNA composition in *N. furzeri*, we created for each of the six related killifish species two small RNA-Seq libraries (see Table 4). These libraries were mapped simultaneously on all available miRBase (release 21) sequences and our annotated miRNAs of *N. furzeri* to observe *N. furzeri* miRNA candidates expressed in other killifish and conserved miRNAs possibly missing in *N. furzeri* but not in the closely related species. In total, 546 (93.7%) of the 583 expressed and 17 (9.9%) of the 171 non-expressed miRNA candidates in *N. furzeri* showed expression in at least one of the related killifish (Fig. 4 shows a miRNA not expressed in *N. furzeri* but in several of the other killifish). Of these expressed miRNAs, 299 belong to the 421 non-conserved *N. furzeri* miRNA genes. To investigate whether miRNA sequences reflect known phylogenetic relationships, we concatenated the sequences of all expressed miRNAs and constructed a phylogenetic tree. This tree perfectly reflected the evolution of the *Nothobranchius* lineage [16]. It is also interesting that the number of *N. furzeri* miRNAs expressed in other killifish species (indicated above the branch in Fig. 5) is inversely correlated to the evolutionary distance, i.e., this number is higher for killifish the closer they are related to *N. furzeri*. *A. striatum*, *N. korthausae*, *N. pienaari*, *N. rachovii*, *N. kunthae* and *N. kadleci* showed expression for 352, 428, 488, 473, 496 and 534 miRNAs, respectively. Most of these expressed miRNAs (>89%) are among the 333 conserved miRNAs of *N. furzeri* (see Supplement Table 3). The composition of expressed miRNAs from the six killifish varies only marginally. The *Nothobranchius* species (except *N. furzeri*) had in total 395 expressed miRNAs in common (of which 148 are non-conserved), and *A. striatum* expressed 324 of them (of which 116 are non-conserved). These 324 miRNAs represent a core of miRNAs from *Nothobranchiidae*, whose origin predates the emergence of annualism in this clade.

**Table 4:** SmallRNA-Seq samples from *Nothobranchius* strains generated in this study. * – unknown; # – number of replicates; + − two weeks post-fertilization plus diapause

Species	Tissue	Age	No. library	#
*N. furzeri MZM*	whole embryos	diapause II^+^	7	7
*N. furzeri MZM*	brain, liver, skin	5, 12, 20, 27, 32, 39	75	4–5
*N. furzeri GRZ*	brain, liver, skin	5, 7, 10, 12, 14	75	5
*A. striatum*	brain	*	2	2
*N. kadleci*	brain	*	2	2
*N. rachovii*	Brain	*	2	2
*N. pienaari*	Brain	*	2	2
*N. kunthae*	Brain	*	2	2
*N. korthausae*	brain	*	2	2

### miRNA clusters and gene duplications

**Fig. 6:**
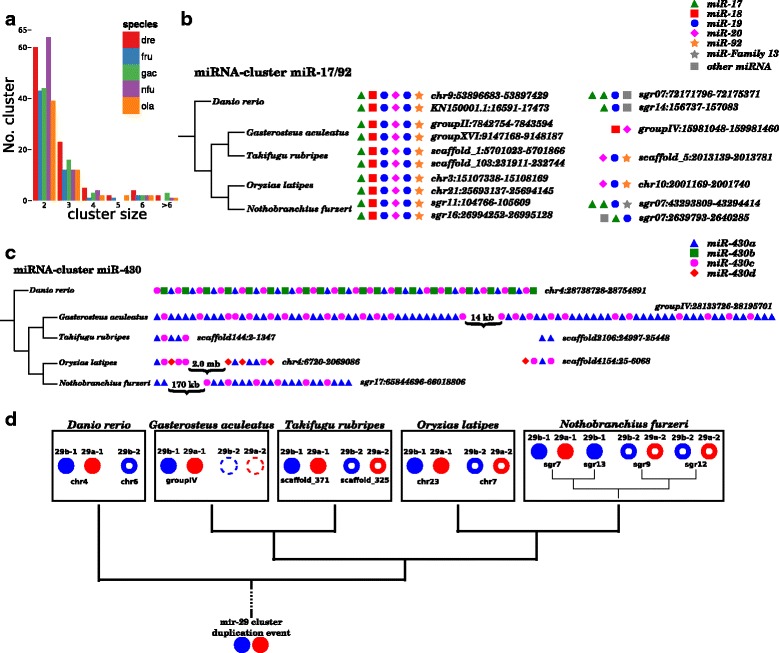
MiRNA cluster comparison between fish. **a** Amount of clusters and their respective sizes with a maximum distance of 10,000 bp between two miRNAs. (nfu - *Nothobranchius furzeri*, dre - *Danio rerio*, ola - *Oryzias latipes*, gac - *Gasterosteus aculeatus*, tru - *Takifugu rubripes*). **b** Structure comparison of the miR-17/92 cluster. Two highly conserved clusters could be identified for each species, as well as some smaller less conserved clusters, containing at least two miRNAs of the miR-17/92 cluster. **c** Structure comparison of the miR-430 cluster. No structural similarity between the different species can be observed. However, *D. rerio*, *G. aculeatus* and *N. furzeri* show some distinct but individual repeating pattern. Even though the gene variants miR-430b and miR-430d seem to be unique to *D. rerio* and *O. latipes*, they can be clearly distinguished, based on sequence alignments. **d** After the ancestral duplication event, the mir-29 cluster is distinguished in the mir-29a/b-1 (*filled red* and *blue dots*) and the mir-29a/b-2 cluster (*red* and *blue circles*). Whereas for *D. rerio* the mir-29a-2 gene seems to be lost, we assume that for *G. aculeatus* the whole second mir-29 cluster (*dashed circles*) is only missing, because of the low quality genome sequencing and assembly. In *N. furzeri* we observe an additional copy for each of the two clusters, except that the mir-29a/b-1 pair was only partially duplicated or the second mir-29a-1 gene was lost again

MiRNAs are known to often occur in clusters [24]. We define a miRNA cluster to consist of at least two miRNAs, with a maximum distance of 10 kb. Examining the localization and distances of the miRNA genes in the five fish species with assembled genomes, we identified 83, 96, 58, 68 and 59 different clusters in *N. furzeri*, *D. rerio*, *O. latipes*, *G. aculeatus* and *T. rubripes*, respectively (see Table 3, Fig. 6a).

In all investigated fish species but *T. rupripes*, the largest cluster is the miR-430 cluster (containing 7 to 55 miRNAs; see Fig. 6c). This cluster is extremely divergent and evolving relatively quickly in each lineage. Not only the number of miR-430 copies within each cluster varies greatly but also the number and organization of the members of this miRNA family. Whereas miR-430a and miR-430c can be found in all five fish species, miR-430b and miR-430d seem to occur only in *D. rerio* and *O. latipes*, respectively. Additionally, no structural similarities or shared repetition patterns can be observed for this miRNA cluster, which is an additional indication of the low purifying selection on this specific gene cluster. However, a clear duplication pattern can be observed for the miR-430 cluster in *D. rerio* (the order miR-430c/b/a is repeated with only a few exceptions) and *N. furzeri* (the order miR-430c/a/a/c/a/a/a is repeated). For *O. latipes* and *G. aculeatus*, the order of miR-430 variants appears to be more random, and *T. rubripes* has too few copies to show any repeated pattern.

Figure 6b depicting the miR-17-92 cluster shows an example of the other extreme: in all five investigated fish species, two perfectly conserved clusters can be found. These represent a duplication of an ancestral cluster present in all vertebrates, and the order of the different members is perfectly conserved. It is known that the miR-17-92 cluster is transcribed polycystronically and acts in oncogenic and tumor suppressor pathways [23, 57]. Furthermore, up to two smaller and lesser conserved clusters, containing at least two miRNAs of the miR-17 or miR-92 family, were identified per fish species, similar to what is known for mammals. Having correctly identified this highly conserved cluster in *N. furzeri* is again good evidence for the high quality of its newly assembled genome and completeness of our miRNA catalogue.

Another example for an evolutionary conserved miRNA cluster is the miR-29 cluster depicted in Fig. 6d. Mir-29 family members are up-regulated during aging in a variety of different tissues including muscle, skin, brain and aorta [2, 18, 46, 54, 56, 66] and appear to be key regulators of age-dependent gene expression [6, 51]. This cluster consists of miR-29a (which is identical to the mammalian miR-29c) and its variant miR-29b and is duplicated at least once. In some fish species, an additional variant miR-29c is known, which is identical to the miR-29a in mammals, with one nucleotide being different outside the seed region [40]. As from RFAM (version 12.1) and miRBase (release 21), miR-29 genes are mainly identified in vertebrates as well as one *Hemichordata* and one *Arthropoda*, so we can only speculate that the original cluster duplication event arose in the early metazoa lineage. In *O. latipes* and *T. rubripes*, both miR-29 clusters are still present, whereas *D. rerio* seems to has lost one copy of the miR-29a gene. For *G. aculeatus*, we were only able to identify one miR-29 cluster. However, since its genome assembly is incomplete, we assume that the second cluster may not be lost but is missing in the current version of its miRNA annotation. Interestingly, in *N. furzeri*, we identified an additional miR-29a/b pair and a fourth single copy of miR-29b. Assuming a complete genome assembly, different scenarios could explain this finding: (1) both original miR-29 clusters were individually duplicated once more, and the fourth miR-29a gene was later lost, (2) one of the two clusters was duplicated as a whole, whereas in the other only miR-29b was copied or (3) both original clusters were duplicated during the same event, and again one of the miR-29a genes was later lost.

About the same amount of different miRBase miRNA families could be identified for all five fish species, despite their big differences in the number of identified miRNA genes. All miRNA genes not matching any known mirRBase family were clustered based on their sequence identity in order to estimate the amount of miRNA ‘families’ not covered by the miRBase database (see Table 3 and Supplement Table 4).

## Conclusion

This study involves a multitude of small RNA-Seq libraries from several tissues, ages, strains and embryos of *N. furzeri* and closely related species. The aim was the characterization of the *N. furzeri* miRNome and a detailed annotation in the recently published genome [50]. The inclusion of other killifish species allowed us to analyze the occurrence of novel miRNAs in the group of annual fish. Due to the fact that we identified roughly the same number of miRNAs in *N. furzeri* as known in *D. rerio* and both fish species share almost equal amounts of miRBase families and unknown miRNA families, we assume that our miRNA catalogue is comparable to the one of the model organism *D. rerio*.

## Methods

### RNA extraction

Animal maintenance was performed as described [59, 60]. To avoid effects of circadian rhythms and feeding, the animals were always sacrificed at 10 a.m. in a fasted state. Animals were sacrificed by an overdose of anesthetics in accordance with the Annex IV of the EU directive 2010/63. They were placed approx. 5–10 min in a methanesulfonate (MS222) solution at a concentration of 1 mg/ml in buffered ethyl 3-aminobenzoate methanesulfonate without prior sedation and observed until no vital signs (body and operculum movement, righting reflex) appeared. At death, animals were transferred on crushed ice, decapitated and organs were harvested. The protocols of animal maintenance and experiments were approved by the local authority in the State of Thuringia (Veterinaer- und Lebensmittelueberwachungsamt). Total RNA was extracted as described [2]. The RNA quality and amount was determined using the Agilent Bioanalyzer 2100 and the RNA 6000 Nano Kit (Agilent Technologies).

### Small RNA library preparation and sequencing

The library preparation and sequencing was done using *Illumina‘s* NGS platform [5]. One μg of total RNA was used for the library preparation, using *Illumina‘s* TruSeq small RNA sample preparation kit, following the manufacturer‘s instruction. The libraries were quantified on the Agilent DNA 1000 chip and subjected to sequencing-by-synthesis on an *Illumina* HiSeq2500 in high-output, 50 bp single-read mode. Sequencing chemistry v3 was used. The read data were extracted in FastQ format, using the Illumina supported tool *bcl2fastq* (v1.8.3 and v1.8.4). The only exceptions were three of the *N. furzeri* embryo samples, which were sequenced on an Illumina HiSeq2000 in 50 bp single-read mode and where read data was extracted in FastQ format using the tool *CASAVA* (v1.8.2). The sequencing resulted in around 4–50 million reads per sample with pooling eight samples per lane.

In total, 169 small RNA-Seq libraries from seven different killifish species were created. 157 of them were obtained from *N. furzeri* strains *GRZ* and *MZM-0410* at several ages from the three tissues brain, liver and skin. The remaining RNA-Seq libraries obtained from *Aphyosemion striatum*, *N. kadleci*, *N. rachovii*, *N. pienaari*, *N. kunthae* and *N. korthausae* were used to identify expression patterns at predicted miRNA locations in *N. furzeri* and miRbase pre-mature miRNA sequences. For details see Table 4, Supplement Table 1 and Supplement Table 2.

### Small RNA-Seq library processing and mapping

In-house scripts were used to cut the RA3 adapter of the TruSeq small RNA preparation kit (5′-TGGAATTCTCGGGTGCCAAGG) from the reads. Additionally, *PRINSEQ* (v0.20.3) [52] was used to trim the reads from both sides in order that the read bases had a minimum quality of 20 and reads were at least 15 bases long. The mapping onto the *N. furzeri* genome was performed with segemehl (v0.2.0) [26] using the *-H 1* option, allowing single reads to be mapped to multiple best fitting locations. The visualization of mapped reads was done using *IGV* (v2.0.34) [62]. Since *Bowtie* (v1.0.0) [37] is the built-in method in miRDeep* for mapping, it was also used for the genomes of *N. furzeri*, *D. rerio*, *O. latipes* and *T. rubripes*.

### Genomes and annotations

The recently published high-quality draft genome assembly and annotation of *N. furzeri* and the small RNA-Seq libraries described above were used for mapping as well as for miRNA and other ncRNA predictions and annotations [50]. Additionally, these RNASeq libraries were also mapped on the following fish genomes: *Danio rerio* (GRCz10) [27], *Oryzias latipes* (HdrR) [31] and *Takifugu rubripes* (FUGU5) [29]. For the annotation comparison, the latest complete genomic information of those three fish and of *Gasterosteus aculeatus* (BROAD S1) [29] were downloaded from the ensembl database [14] Additionally, for miRNA target prediction, the recent genomes and annotations of *Homo sapiens* (GRCh38) and *Mus musculus* (GRCm38) from the ensemble database were used.

### ncRNA and miRNA annotation

Already characterized and conserved non-coding RNAs were annotated with *GoRAP 2.0*, which is based on the RFAM database, currently holding 2450 ncRNA families (v12.0) [44]. For an initial prediction of candidate miRNAs, a combination of five tools was used, each of them following a different annotation strategy: miRDeep* (v32) [1], *Infernal* (v1.1) [45], *BLAST* (v2.2.30) [9], *GoRAP* (v2.0, unpublished) and *CID-miRNA* (version from April 2015) [65]. A detailed description of the individual searches can be found below. All results were merged and putative miRNAs overlapping with genes of the recently published *N. furzeri* annotation were removed. The expression profiles of the remaining non-redundant candidate miRNA genes were analyzed automatically using *Blockbuster* (v1) [35] and in-house scripts in order to mark candidates that did not exhibit a typical miRNA expression profile (according to [30, 36]). All candidates were additionally manually examined and filtered by carefully checking the features of the potential hairpin secondary structure as well as the precise mapping of reads supporting the predicted precursor miRNA, leading to the final set of miRNA predictions.

#### miRDeep* search

Mappings of 39 MZM brain, 15 GRZ brain, 25 GRZ liver, 28 MZM liver, 3 MZM skin and 7 MZM embryo small RNA-Seq libraries were used on four different fish genomes (*N. furzeri*, *D. rerio*, *O. latipes*, *T. rubripes*) as input for miRDeep* (for a detailed list of used libraries, see Supplement Table 1). Predictions from all 117 mappings were pooled together in order to obtain a comprehensive representation of the miRDeep* results. To each predicted miRNA hairpin sequence, we assigned the average of the miRDeep* score computed across the multiple samples were the sequence was found. The merged non-redundant list of identified miRNA sequences was re-mapped with *BLAT* [32] on the *N. furzeri* genome, and only gap-free alignments were accepted. These loci underwent further filtering steps: (i) a hairpin sequence was considered reliable if it showed a *BLAT* hit (one mismatch allowed) in miRbase (release 20) [33] or a miRDeep* score equal or more than 7 and (ii) overlapping hairpin loci (i.e., within 100 nt) were discarded, and the sequence with the highest score was kept. Predictions where no hits in miRBase could be obtained were further analyzed based on their secondary structure. Therefore, corresponding sequences were extended by 50 nt on either side and were compared with Rfam using *Infernal*. All predicted loci that had a significant hit to a known miRNA secondary structure or no hit at all were kept, while loci hitting other ncRNAs were discarded.

#### Infernal search

For the *Infernal* search on the *N. furzeri* genome, 155 hand-curated pre-miRNA covariance models were used as input [7, 25] and only significant hits with a *p*-value of *p* < 0.005 were kept.

#### BLAST search

In order to identify candidates from the most conserved miRNA families, *blastn* was used with all mature and pre-mature miRNA sequences available on miRBase (release 21) [34]. Only non-redundant hits were kept if they spanned the complete sequences of their corresponding input miRNAs to at least 90% with no gaps allowed. To further reduce false positive hits, a stringent cut-off of *p* < 10^−7^ was chosen.

#### CID-miRNA search

Being based on a stochastic context-free grammar model to identify possible pre-miRNAs, *CID-miRNA* follows a similar approach as *Infernals* covariance models. The *N. furzeri* genome was given as input with the following thresholds: putative miRNAs have a length between 60 bp and 120 bp, and the grammar and structural cut-off were set to the recommended values of −0.609999 and 23, respectively.

### miRNA target prediction

To determine putative *N. furzeri* mRNA targets of the miRNA candidates the *TargetScan* tool was used [39]. As input the putative miRNA seed regions and the known 3′-UTR sites of all annotated mRNAs of *N. furzeri* as well as the ones from *D. rerio*, *M. musculus* and *H. sapiens* were used. The input files and the resulting output can be found in the online supplement. Enrichment scores of miRNA targets within different published sets of differentially expressed *N. furzeri* genes were calculated using the hypergeometric test:

$$ p- value=\frac{R!n!\left(N-R\right)!\left(N-n\right)!}{N!}{\sum}_{i=r}^{\mathit{\min}\left(n,R\right)}\frac{1}{i!\left(R-i\right)!\left(n-i\right)!\left(N-R-n+i\right)i} $$

where *N* is the total amount of known protein coding genes in *N. furzeri*, *R* the amount of differentially expressed genes of one of the given sets, *n* the number of protein coding genes with predicted miRNA target sites and *r* the size of differentially expressed genes with predicted miRNA target sites. Enrichment of individual miRNAs, being enriched in any of the gene sets, were calculated similarly, with *N* being the total amount of protein coding genes with predicted miRNA target sites and *n* the number of genes, showing a target site of the respective miRNA. The resulting *p*-values were adjusted using Benjamini and Hochbergs FDR approach and were considered significant if *p* was less than 0.05 [4].

## Additional files

Additional file 1: Annotation of all identified ncRNAs in Danio rerio in gff format. (DOCX 7 kb)

Additional file 2: Annotation of all identified pre-miRNAs and corresponding mature miRNAs in Danio rerio in gff format. (DOCX 48 kb)

## Abbreviations

*A. striatum*: *Aphyosemion striatum*
*D. rerio*: *Danio rerio*
Dre: *Danio rerio*
*G. aculeatus*: *Gasterosteus aculeatus*
Gac: *Gasterosteus aculeatus*
*H. sapiens*: *Homo sapiens*
Hsa: *Homo sapiens*
lncRNA: long non-coding RNA
*M. musculus*: *Mus musculus*
miRNA: microRNA
Mmu: *Mus musculus*
mRNA: messenger RNA
*N. furzeri*: *Nothobranchius furzeri*
N. kadleci: Nothobranchius kadleci
*N. korthausae*: *Nothobranchius korthausae*
N. kunthae: Nothobranchius kunthae
N. pienaari: Nothobranchius pienaari
*N. rachovii*: *Nothobranchius rachovii*
ncRNA: non-coding RNA
Nfu: *Nothobranchius furzeri*
*O. latipes*: *Oryzias latipes*
Ola: *Oryzias latipes*
PCA: Principal component analysis
rRNA: ribosomal RNA
*T. rubripes*: *Takifugu rubripes*
tRNA: transfer RNA
Tru: *Takifugu rubripes*
UTR: Untranslated region

## References

[1] An J, Lai J, Lehman ML, Nelson CC (2013). miRDeep*: an integrated application tool for miRNA identification from RNA sequencing data. Nucleic Acids Res..

[2] Baumgart M, Groth M, Priebe S, Appelt J, Guthke R, Platzer M, Cellerino A (2012). Age-dependent regulation of tumor-related microRNAs in the brain of the annual fish Nothobranchius Furzeri. Mech Ageing Dev.

[3] Baumgart M, Groth M, Priebe S, Savino A, Testa G, Dix A, Ripa R, Spallotta F, Gaetano C, Ori M (2014). RNA-Seq of the aging brain in the short-lived fish n. furzeri–conserved pathways and novel genes associated with neurogenesis. Aging Cell.

[4] Benjamini Y, Hochberg Y (1995). Controlling the false discovery rate: a practical and powerful approach to multiple testing. J Royal Stat Soc Ser B (Methodol).

[5] Bentley DR, Balasubramanian S, Swerdlow HP, Smith GP, Milton J, Brown CG, Hall KP, Evers DJ, Barnes CL, Bignell HR (2008). Accurate whole human genome sequencing using reversible terminator chemistry. Nature.

[6] Boon RA, Iekushi K, Lechner S, Seeger T, Fischer A, Heydt S, Kaluza D, Tr’eguer K, Carmona G, Bonauer A (2013). Microrna-34a regulates cardiac ageing and function. Nature.

[7] Braasch I, Gehrke AR, Smith JJ, Kawasaki K, Manousaki T, Pasquier J, Amores A, Desvignes T, Batzel P, Catchen J (2016). The spotted gar genome illuminates vertebrate evolution and facilitates human-teleost comparisons. Nat Genet.

[8] Brodeur GM, Seeger RC, Schwab M, Varmus HE, Bishop JM (1984). Amplification of n-myc in untreated human neuroblastomas correlates with advanced disease stage. Science.

[9] Camacho C, Coulouris G, Avagyan V, Ma N, Papadopoulos J, Bealer K, Madden TL (2009). BLAST+: architecture and applications. BMC bioinformatics.

[10] Cellerino A. Regulation of microRNA expression in the neuronal stem cell niches during aging of the short-lived annual fish Nothobranchius Furzeri. Regulatory RNAs in the Nervous System, 8 vol. 2014. 10.3389/fncel.2014.00051 PMC3930850 24600353

[11] Cellerino A, Valenzano DR, Reichard M (2016). From the bush to the bench: the annual Nothobranchius fishes as a new model system in biology. Biol Rev.

[12] Cheng JM, Hiemstra JL, Schneider SS, Naumova A, Cheung N-KV, Cohn SL, Diller L, Sapienza C, Brodeur GM (1993). Preferential amplification of the paternal allele of the n–myc gene in human neuroblastomas. Nat Genet.

[13] Citterio C, Menacho-M’arquez M, Garc’ıa-Escudero R, Larive RM, Barreiro O, S’anchez-Madrid F, Paramio JM, Bustelo XR (2012). The rho exchange factors vav2 and vav3 control a lung metastasis-specific transcriptional program in breast cancer cells. Sci Signal.

[14] Cunningham F, Amode MR, Barrell D, Beal K, Billis K, Brent S, Carvalho-Silva D, Clapham P, Coates G, Fitzgerald S (2015). Ensembl 2015. Nucleic Acids Res.

[15] Di Cicco E, Tozzini ET, Rossi G, Cellerino A (2011). The short-lived annual fish nothobranchius furzeri shows a typical teleost aging process reinforced by high incidence of age-dependent neoplasias. Exp Gerontol.

[16] Dorn A, Musilov’a Z, Platzer M, Reichwald K, Cellerino A (2014). The strange case of east african annual fishes: aridification correlates with diversification for a savannah aquatic group?. BMC Evol Biol.

[17] Fausto N, Campbell JS, Riehle KJ (2006). Liver regeneration. Hepatology.

[18] Fenn AM, Smith KM, Lovett-Racke AE, Guerau-de Arellano M, Whitacre CC, Godbout JP (2013). Increased micro-rna 29b in the aged brain correlates with the reduction of insulin-like growth factor-1 and fractalkine ligand. Neurobiol Aging.

[19] Giraldez AJ, Mishima Y, Rihel J, Grocock RJ, Van Dongen S, Inoue K, Enright AJ, Schier AF (2006). Zebrafish MiR-430 promotes deadenylation and clearance of maternal mRNAs. Science.

[20] Griffiths-Jones S, Bateman A, Marshall M, Khanna A, Eddy SR (2003). Rfam: an RNA family database. Nucleic Acids Res.

[21] Griffiths-Jones S, Grocock RJ, Van Dongen S, Bateman A, Enright AJ (2006). miRBase: microRNA sequences, targets and gene nomenclature. Nucleic Acids res.

[22] Gudas JM, Payton M, Thukral S, Chen E, Bass M, Robinson MO, Coats S (1999). Cyclin e2, a novel g1 cyclin that binds cdk2 and is aberrantly expressed in human cancers. Mol Cell Biol.

[23] He L, Thomson JM, Hemann MT, Hernando-Monge E, Mu D, Goodson S, Powers S, Cordon-Cardo C, Lowe SW, Hannon GJ (2005). A microrna polycistron as a potential human oncogene. Nature.

[24] Hertel J, Lindemeyer M, Missal K, Fried C, Tanzer A, Flamm C, Hofacker IL, Stadler PF (2006). The expansion of the metazoan microRNA repertoire. BMC Genomics.

[25] Hertel J, Stadler PF (2015). The expansion of animal microRNA families revisited. Life.

[26] Hoffmann S, Otto C, Kurtz S, Sharma CM, Khaitovich P, Vogel J, Stadler PF, Hackermüller J (2009). Fast mapping of short sequences with mismatches, insertions and deletions using index structures. PLoS Comput Biol.

[27] Howe K, Clark MD, Torroja CF, Torrance J, Berthelot C, Muffato M, Collins JE, Humphray S, McLaren K, Matthews L (2013). The zebrafish reference genome sequence and its relationship to the human genome. Nature.

[28] Jiang Y, Prabakaran I, Wan F, Mitra N, Furstenau DK, Hung RK, Cao S, Zhang PJ, Fraker DL, Guvakova MA (2014). Vav2 protein overexpression marks and may predict the aggressive subtype of ductal carcinoma in situ. Biomark Res.

[29] Jones FC, Grabherr MG, Chan YF, Russell P, Mauceli E, Johnson J, Swofford R, Pirun M, Zody MC, White S (2012). The genomic basis of adaptive evolution in threespine sticklebacks. Nature.

[30] Jung C-H, Hansen MA, Makunin IV, Korbie DJ, Mattick JS (2010). Identification of novel non-coding RNAs using profiles of short sequence reads from next generation sequencing data. BMC Genomics.

[31] Kasahara M, Naruse K, Sasaki S, Nakatani Y, Qu W, Ahsan B, Yamada T, Nagayasu Y, Doi K, Kasai Y (2007). The medaka draft genome and insights into vertebrate genome evolution. Nature.

[32] Kent WJ (2002). BLAT – the BLAST-like alignment tool. Genome Res.

[33] Kozomara A, Griffiths-Jones S (2010). miRBase: integrating microRNA annotation and deep-sequencing data. Nucleic Acids Res.

[34] Kozomara A, Griffiths-Jones S (2013). miRBase: annotating high confidence microRNAs using deep sequencing data. Nucleic Acids Res.

[35] Langenberger D, Bermudez-Santana C, Hertel J, Hoffmann S, Khaitovich P, Stadler PF (2009). Evidence for human microrna-offset rnas in small rna sequencing data. Bioinformatics.

[36] Langenberger D, Bermudez-Santana C, Stadler PF, Hoffmann S (2010). Identification and classification of small RNAs in transcriptome sequence data. Pac Symp Biocomput.

[37] Langmead B, Trapnell C, Pop M, Salzberg SL (2009). Ultrafast and memoryefficient alignment of short DNA sequences to the human genome. Genome Biol.

[38] Lee M-Y, Kim H-J, Kim M-A, Jee HJ, Kim AJ, Bae Y-S, Park J-I, Chung JH, Yun J (2008). Nek6 is involved in g2/m phase cell cycle arrest through dna damage-induced phosphorylation. Cell Cycle.

[39] Lewis BP, Burge CB, Bartel DP (2005). Conserved seed pairing, often flanked by adenosines, indicates that thousands of human genes are microrna targets. Cell.

[40] Liston A, Papadopoulou AS, Danso-Abeam D, Dooley J (2012). Microrna-29 in the adaptive immune system: setting the threshold. Cell Mol Life Sci.

[41] Marco A, Ninova M, Ronshaugen M, Griffiths-Jones S (2013). Clusters of microRNAs emerge by new hairpins in existing transcripts. Nucleic Acids Res.

[42] Marqu’es F, Moreau J-L, Peaucellier G, Lozano J-C, Schatt P, Picard A, Callebaut I, Perret E, Genevi’ere A-M (2000). A new subfamily of high molecular mass cdc2-related kinases with pitai/vre motifs. Biochem. Biophys. Res. Commun..

[43] Michalopoulos GK, DeFrances MC (1997). Liver regeneration. Science.

[44] Nawrocki EP, Burge SW, Bateman A, Daub J, Eberhardt RY, Eddy SR, Floden EW, Gardner PP, Jones TA, Tate J (2014). Rfam 12.0: updates to the RNA families database. Nucleic Acids Res.

[45] Nawrocki EP, Eddy SR (2013). Infernal 1.1: 100-fold faster RNA homology searches. Bioinformatics.

[46] Nolan K, Mitchem MR, Jimenez-Mateos EM, Henshall DC, Concannon CG, Prehn JH (2014). Increased expression of microrna-29a in ALS mice: functional analysis of its inhibition. J Mol Neurosci.

[47] Osella M, Bosia C, Cor’a D, Caselle M (2011). The role of incoherent microRNA-mediated feedforward loops in noise buffering. PLoS Comput Biol.

[48] Pinz’on N, Li B, Martinez L, Sergeeva A, Presumey J, Apparailly F, Seitz H (2017). microrna target prediction programs predict many false positives. Genome Res.

[49] Platzer M, Englert C (2016). Nothobranchius Furzeri: a model for aging research and more. Trends Genet.

[50] Reichwald K, Petzold A, Koch P, Downie BR, Hartmann N, Pietsch S, Baumgart M, Chalopin D, Felder M, Bens M (2015). Insights into sex chromosome evolution and aging from the genome of a short-lived fish. Cell.

[51] Ripa R, Dolfi L, Terrigno M, Pandolfini L, Arcucci V, Groth M, Tozzini ET, Baumgart M, Cellerino A (2017). Microrna mir-29 controls a compensatory response to limit neuronal iron accumulation during adult life and aging. BMC Biol.

[52] Schmieder R, Edwards R (2011). Quality control and preprocessing of metagenomic datasets. Bioinformatics.

[53] Siciliano V, Garzilli I, Fracassi C, Criscuolo S, Ventre S, di Bernardo D. MiRNAs confer phenotypic robustness to gene networks by suppressing biological noise. Nat Commun. 2013;4 10.1038/ncomms3364 PMC3836244 24077216

[54] Somel M, Guo S, Fu N, Yan Z, Hu HY, Xu Y, Yuan Y, Ning Z, Hu Y, Menzel C (2010). Microrna, mRNA, and protein expression link development and aging in human and macaque brain. Genome Res.

[55] Suzuki R, Shimodaira H (2006). Pvclust: an r package for assessing the uncertainty in hierarchical clustering. Bioinformatics.

[56] Takahashi M, Eda A, Fukushima T, Hohjoh H (2012). Reduction of type IV collagen by upregulated mir-29 in normal elderly mouse and klotho-deficient, senescence-model mouse. PloS One.

[57] Tanzer A, Stadler PF (2004). Molecular evolution of a microrna cluster. J Mol Biol.

[58] Taub R (2004). Liver regeneration: from myth to mechanism. Nat Rev Mol Cell Biol.

[59] Terzibasi E, Lefrançois C, Domenici P, Hartmann N, Graf M, Cellerino A (2009). Effects of dietary restriction on mortality and age-related phenotypes in the shortlived fish *Nothobranchius furzeri*. Aging Cell.

[60] Terzibasi E, Valenzano DR, Benedetti M, Roncaglia P, Cattaneo A, Domenici L, Cellerino A (2008). Large differences in aging phenotype between strains of the short-lived annual fish *Nothobranchius furzeri*. PLoS One.

[61] Thatcher EJ, Bond J, Paydar I, Patton JG (2008). Genomic organization of zebrafish microRNAs. BMC Genomics.

[62] Thorvaldsd’ottir H, Robinson JT, Mesirov JP (2013). Integrative Genomics Viewer (IGV): high-performance genomics data visualization and exploration. Brief Bioinform.

[63] Tozzini ET, Baumgart M, Battistoni G, Cellerino A (2012). Adult neurogenesis in the short-lived teleost nothobranchius furzeri: localization of neurogenic niches, molecular characterization and effects of aging. Aging Cell.

[64] Tozzini ET, Savino A, Ripa R, Battistoni G, Baumgart M, Cellerino A. Regulation of microrna expression in the neuronal stem cell niches during aging of the short-lived annual fish nothobranchius furzeri. Front Cell Neurosci. 2014;8 10.3389/fncel.2014.00051 PMC3930850 24600353

[65] Tyagi S, Vaz C, Gupta V, Bhatia R, Maheshwari S, Srinivasan A, Bhattacharya A (2008). CID-miRNA: a web server for prediction of novel miRNA precursors in human genome. Biochem Biophys Res Commun.

[66] Ugalde AP, Ramsay AJ, de la Rosa J, Varela I, Mariño G, Cadiñanos J, Lu J, Freije JM, López-Otín C (2011). Aging and chronic dna damage response activate a regulatory pathway involving mir-29 and p53. EMBO J.

[67] Valenzano DR, Benayoun BA, Singh PP, Zhang E, Etter PD, Hu C-K, Clément-Ziza M, Willemsen D, Cui R, Harel I (2015). The African turquoise killifish genome provides insights into evolution and genetic architecture of lifespan. Cell.

[68] Wang Y, Luo J, Zhang H, Lu J (2016). microRNAs in the same clusters evolve to coordinately regulate functionally related genes. Mol Biol Evol.

