# Correction to: A miRNA catalogue and ncRNA annotation of the short-living fish *Nothobranchius furzeri*

**DOI:** 10.1186/s12864-019-6312-y

**Published:** 2019-11-27

**Authors:** Mario Baumgart, Emanuel Barth, Aurora Savino, Marco Groth, Philipp Koch, Andreas Petzold, Ivan Arisi, Matthias Platzer, Manja Marz, Alessandro Cellerino

**Affiliations:** 1 Leibniz Institute for Age Research - Fritz Lipmann Institute (FLI), Beutenbergstraße 11, 07745 Jena, Germany; 2 Bioinformatics/High Throughput Analysis, Friedrich Schiller University Jena, Leutragraben 1, 07743 Jena, Germany; 3 Babraham Institute, Cambridge, England; 4 Dresden University of Technology, Dresden, Germany; 5 European Brain Research Institute (EBRI), Rome, Italy; 6 Laboratory of Biology, Scuola Normale Superiore, 56126 Pisa, Italy

**Correction to: BMC Genomics (2017) 18:693**

**https://doi.org/10.1186/s12864-017-3951-8**

Following the publication of this article [1], the authors reported that the images of Figs. 1, 2 and 3 were published in the incorrect order, whereby they mismatch with their captions. The figures are reproduced in the correct sequence with the correct captions in this Correction article.

**Fig. 1:**
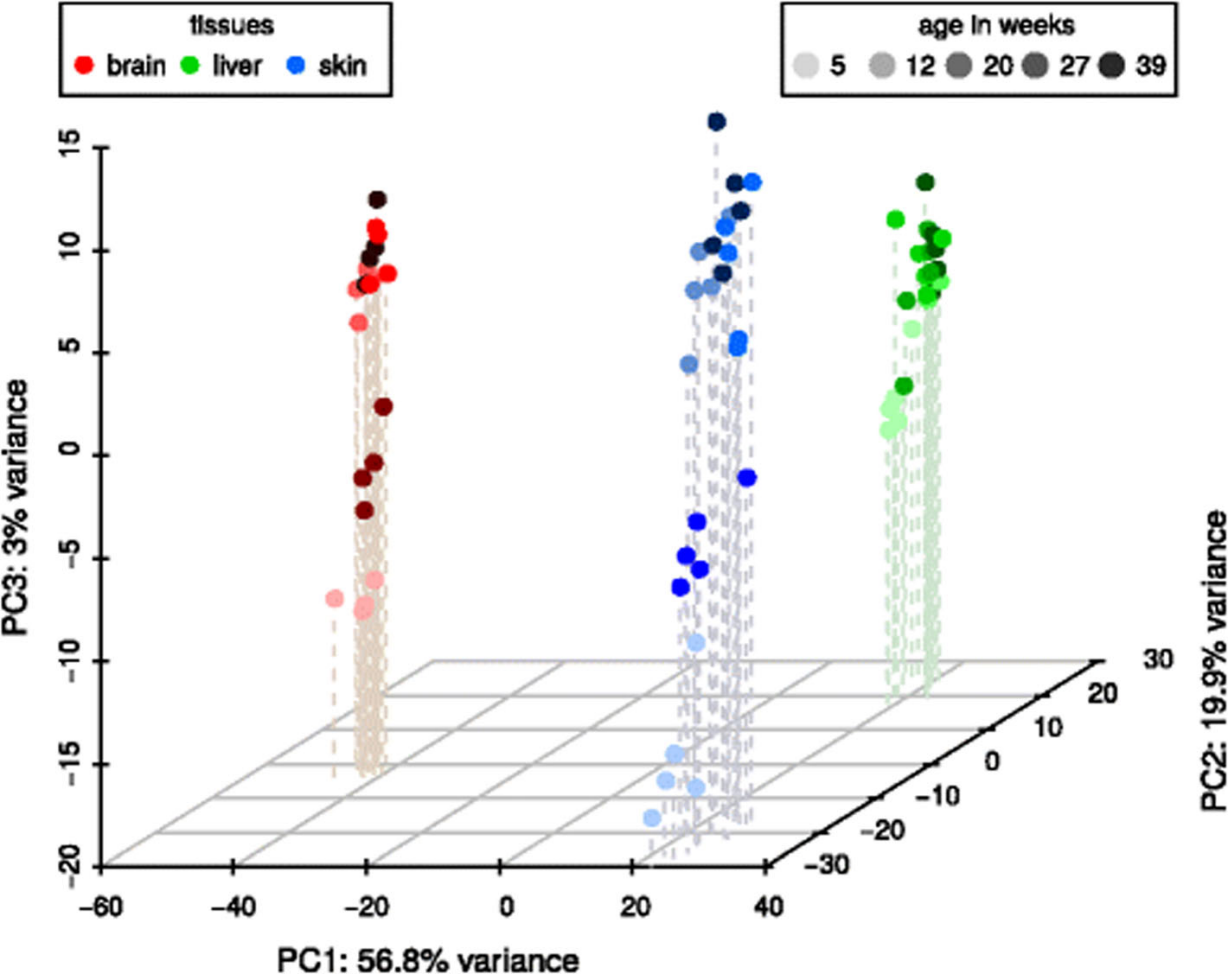
A three-dimensional PCA plot of the *N. furzeri* MZM small RNA-Seq libraries of all three tissues (brain – red, liver – green, blue – skin) and all investigated ages (from light to dark: 5, 12, 20, 27, 39 weeks). Whereas the samples cluster well according to their tissue belongings, a distinct separation regarding the ages can only be observed for the youngest samples in each tissue. A PCA plot of the GRZ strain, can be found in Supplement Table 2

**Fig. 2:**
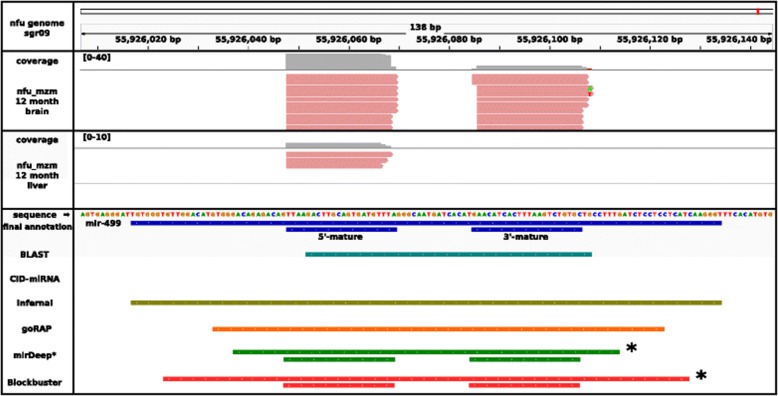
Annotation, expression profiles and prediction comparison for miR-499.We annotated the pre-miR-499 on sgr09, position 55,926,017– 55,926,134 and the two mature miRNAs at 55,926,048–55,926,069 and 55,926,085–55,926,106. The six methods used for miRNA detection are displayed, CID-miRNA was not able to detect this miRNA. Tools working independent of the small RNA-Seq data BLAST (cyan), Infernal (olive green) and goRAP (orange) vary in their annotation length. The latter two programs are based on covariance models, identifying mostly the complete pre-miRNA. The remaining two programs miRDeep* and Blockbuster are based on small RNA-Seq data (*) and therefore accurately annotate the mature miRNAs. MiR-499 is expressed weakly within *N. furzeri* MZM 12 month liver library and therefore could not be detected by miRDeep* and Blockbuster. In the *N. furzeri* MZM 12 month brain library, miR-499 was expressed strongs enough to be detected by both programs

**Fig. 3:**
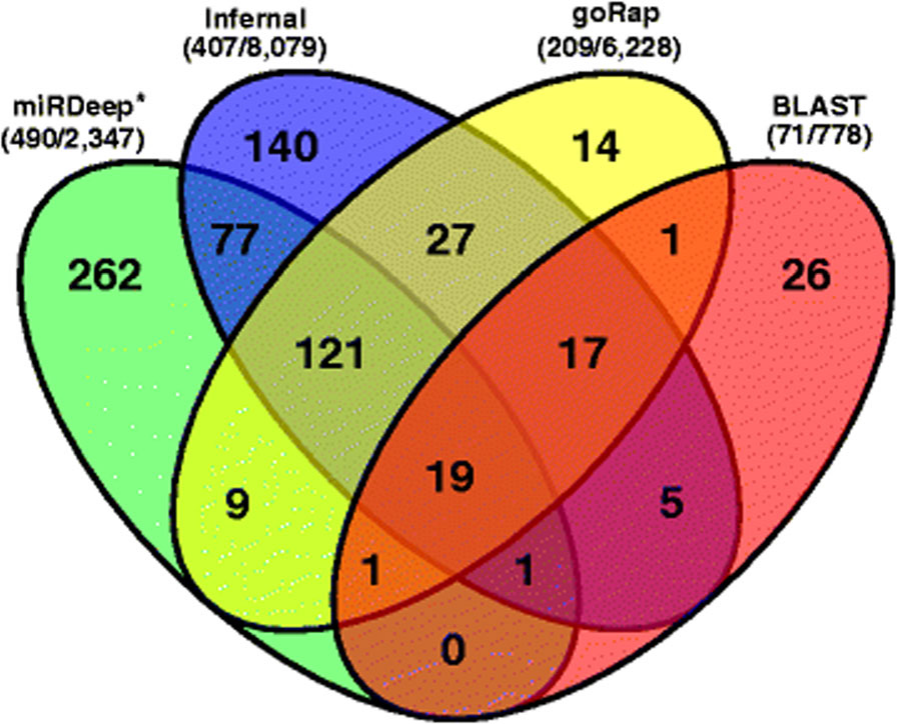
Venn diagram of predicted miRNA genes from four tools miRDeep*, Infernal, goRap and BLAST. Only 2 of the 33 candidates predicted by CID-miRNA overlapped with any of the other miRNA candidates. Nevertheless, all 33 candidates were selected as miRNAs after manual inspectations. The total number of miRNA predictions after and before applying any filtering step are shown in brackets for each tool

Furthermore, in the ‘Availability of data and materials’ declaration the sentence “Supplementary material can be found online at http://www.rna.uni-jena.de/en/supplements/nothobranchius-furzeri-mirnome/“ should now read “Supplementary material can be found online at https://osf.io/25mxb/ (DOI 10.17605/OSF.IO/25MXB).”
