# Supplementary material for: A miRNA catalogue and ncRNA annotation of the short-living fish Nothobranchius furzeri
Source: BMC Genomics. 2017 Sep 5;18:693. doi: 10.1186/s12864-017-3951-8 (PMC5584509; doi:10.1186/s12864-017-3951-8)
Supplement: Supplementary file 1 — Annotation of all identified ncRNAs in Danio rerio in gff format. (DOCX 7 kb) [file 12864_2017_3951_MOESM1_ESM.docx]

scaffold00044 goRAP RF01684_mascRNA-menRNA 97303 97354 . - . RPKM=.;Reads=.;Filter=X;Overlaps=.

scaffold00078 goRAP RF01684_mascRNA-menRNA 104304 104355 . - . RPKM=.;Reads=.;Filter=X;Overlaps=.

scaffold00152 goRAP RF00001_5S_rRNA 152711 152825 . + . RPKM=.;Reads=.;Filter=!;Overlaps=.

scaffold00195 goRAP RF00001_5S_rRNA 143121 143235 . + . RPKM=.;Reads=.;Filter=!;Overlaps=.

scaffold00218 goRAP RF01803_GABA3 42766 42819 . - . RPKM=.;Reads=.;Filter=!;Overlaps=.

scaffold00300 goRAP RF00485_K_chan_RES 82653 82765 . - . RPKM=.;Reads=.;Filter=X;Overlaps=.

scaffold00366 goRAP RF02543_LSU_rRNA_eukarya 67212 74242 . + . RPKM=.;Reads=.;Filter=X;Overlaps=.

scaffold00430 goRAP RF00381_Antizyme_FSE 75961 76019 . + . RPKM=.;Reads=.;Filter=!;Overlaps=.

scaffold00448 goRAP RF01803_GABA3 25588 25641 . + . RPKM=.;Reads=.;Filter=!;Overlaps=.

scaffold00522 goRAP RF02543_LSU_rRNA_eukarya 81715 86105 . - . RPKM=.;Reads=.;Filter=!;Overlaps=.

scaffold00552 goRAP RF01684_mascRNA-menRNA 41013 41067 . + . RPKM=.;Reads=.;Filter=B;Overlaps=.

scaffold00641 goRAP RF00001_5S_rRNA 6548 6662 . + . RPKM=.;Reads=.;Filter=!;Overlaps=.

scaffold00676 goRAP RF00001_5S_rRNA 43126 43244 . - . RPKM=.;Reads=.;Filter=X;Overlaps=.

scaffold00982 goRAP RF01684_mascRNA-menRNA 52546 52597 . - . RPKM=.;Reads=.;Filter=X;Overlaps=.

scaffold01015 goRAP RF00001_5S_rRNA 48161 48274 . + . RPKM=.;Reads=.;Filter=!;Overlaps=.

scaffold01293 goRAP RF00001_5S_rRNA 36095 36209 . + . RPKM=.;Reads=.;Filter=!;Overlaps=.

scaffold01293 goRAP RF00001_5S_rRNA 36557 36671 . + . RPKM=.;Reads=.;Filter=!;Overlaps=.

scaffold01293 goRAP RF00001_5S_rRNA 37029 37143 . + . RPKM=.;Reads=.;Filter=!;Overlaps=.

scaffold01687 goRAP RF00002_5_8S_rRNA 27112 27265 . - . RPKM=.;Reads=.;Filter=!;Overlaps=.

scaffold01744 goRAP RF00002_5_8S_rRNA 17425 17524 . - . RPKM=.;Reads=.;Filter=X;Overlaps=.

scaffold01784 goRAP RF00002_5_8S_rRNA 16701 16803 . + . RPKM=.;Reads=.;Filter=X;Overlaps=.

scaffold01820 goRAP RF02543_LSU_rRNA_eukarya 2198 5408 . - . RPKM=.;Reads=.;Filter=!;Overlaps=.

scaffold02057 goRAP RF00003_U1 19124 19288 . + . RPKM=.;Reads=.;Filter=!;Overlaps=.

scaffold02588 goRAP RF01684_mascRNA-menRNA 1213 1268 . - . RPKM=.;Reads=.;Filter=P;Overlaps=.

scaffold02633 goRAP RF01684_mascRNA-menRNA 1491 1546 . - . RPKM=.;Reads=.;Filter=X;Overlaps=.

scaffold02634 goRAP RF00001_5S_rRNA 10587 10701 . - . RPKM=.;Reads=.;Filter=!;Overlaps=.

scaffold02634 goRAP RF00001_5S_rRNA 11044 11186 . + . RPKM=.;Reads=.;Filter=X;Overlaps=.

scaffold02634 goRAP RF00001_5S_rRNA 11047 11165 . - . RPKM=.;Reads=.;Filter=X;Overlaps=.

scaffold02728 goRAP RF01684_mascRNA-menRNA 2662 2713 . + .RPKM=.;Reads=.;Filter=X;Overlaps=.

scaffold02728 goRAP RF01684_mascRNA-menRNA 4173 4224 . - .RPKM=.;Reads=.;Filter=X;Overlaps=.

scaffold03244 goRAP RF00001_5S_rRNA 3035 3149 . - . RPKM=.;Reads=.;Filter=!;Overlaps=.

scaffold03466 goRAP RF01960_SSU_rRNA_eukarya 60 1749 . - . RPKM=.;Reads=.;Filter=!;Overlaps=.

scaffold03567 goRAP RF00004_U2 266 457 . - . RPKM=.;Reads=.;Filter=!;Overlaps=.

scaffold03794 goRAP RF01684_mascRNA-menRNA 1274 1326 . + . RPKM=.;Reads=.;Filter=X;Overlaps=.

scaffold04079 goRAP RF01684_mascRNA-menRNA 26 81 . + . RPKM=.;Reads=.;Filter=X;Overlaps=.

scaffold04232 goRAP RF00003_U1 263 427 . - . RPKM=.;Reads=.;Filter=!;Overlaps=.

scaffold05023 goRAP RF01684_mascRNA-menRNA 1017 1069 . + . RPKM=.;Reads=.;Filter=X;Overlaps=.

sgr01 goRAP RF00003_U1 46363361 46363525 . + . RPKM=.;Reads=.;Filter=!;Overlaps=.

sgr01 goRAP RF00003_U1 67712149 67712313 . - . RPKM=.;Reads=.;Filter=!;Overlaps=.

sgr01 goRAP RF00024_Telomerase-vert 13321493 13321827 . + . TPM=.;FPKM=.;Reads=.;Filter=!;Note=.;Overlaps=.

sgr01 goRAP RF00485_K_chan_RES 6395466 6395579 . - . RPKM=.;Reads=.;Filter=X;Overlaps=.

sgr02 goRAP RF00004_U2 1170560 1170749 . + . RPKM=.;Reads=.;Filter=!;Overlaps=.

sgr02 goRAP RF01684_mascRNA-menRNA 19189476 19189529 . - . RPKM=.;Reads=.;Filter=X;Overlaps=.

sgr02 goRAP RF01803_GABA3 27599485 27599538 . - . RPKM=.;Reads=.;Filter=!;Overlaps=.

sgr02 goRAP RF01960_SSU_rRNA_eukarya 8630304 8633208 . - . RPKM=.;Reads=.;Filter=!;Overlaps=.

sgr03 goRAP RF00001_5S_rRNA 18773184 18773298 . + . RPKM=.;Reads=.;Filter=!;Overlaps=.

sgr03 goRAP RF00026_U6 15721712 15721818 . + . RPKM=.;Reads=.;Filter=!;Overlaps=.

sgr03 goRAP RF00485_K_chan_RES 52339274 52339387 . - . RPKM=.;Reads=.;Filter=X;Overlaps=.

sgr04 goRAP RF00485_K_chan_RES 13757420 13757533 . - . RPKM=.;Reads=.;Filter=X;Overlaps=.

sgr04 goRAP RF01684_mascRNA-menRNA 2656790 2656844 . - .RPKM=.;Reads=.;Filter=P;Overlaps=.

sgr04 goRAP RF01803_GABA3 10129742 10129795 . + . RPKM=.;Reads=.;Filter=!;Overlaps=.

sgr05 goRAP RF00001_5S_rRNA 12019114 12019228 . + . RPKM=.;Reads=.;Filter=!;Overlaps=.

sgr05 goRAP RF00001_5S_rRNA 14425711 14425825 . + . RPKM=.;Reads=.;Filter=!;Overlaps=.

sgr05 goRAP RF00001_5S_rRNA 46897686 46897804 . + . RPKM=.;Reads=.;Filter=X;Overlaps=.

sgr05 goRAP RF00004_U2 15447431 15447621 . + . RPKM=.;Reads=.;Filter=!;Overlaps=.

sgr05 goRAP RF00004_U2 15855563 15855753 . + . RPKM=.;Reads=.;Filter=!;Overlaps=.

sgr05 goRAP RF00004_U2 15861146 15861330 . + . RPKM=.;Reads=.;Filter=!;Overlaps=.

sgr05 goRAP RF00004_U2 15875060 15875243 . + . RPKM=.;Reads=.;Filter=!;Overlaps=.

sgr05 goRAP RF00004_U2 15881830 15882020 . + . RPKM=.;Reads=.;Filter=!;Overlaps=.

sgr05 goRAP RF00006_Vault 12170483 12170582 . + . RPKM=.;Reads=.;Filter=!;Overlaps=.

sgr05 goRAP RF00006_Vault 12171414 12171512 . + . RPKM=.;Reads=.;Filter=!;Overlaps=.

sgr05 goRAP RF00100_7SK 3101874 3102181 . + . RPKM=.;Reads=.;Filter=!;Overlaps=.

sgr05 goRAP RF00460_U1A_PIE 21780561 21780636 . + . RPKM=.;Reads=.;Filter=!;Overlaps=.

sgr05 goRAP RF01803_GABA3 60639101 60639154 . + . RPKM=.;Reads=.;Filter=!;Overlaps=.

sgr06 goRAP RF00001_5S_rRNA 63397080 63397194 . - . RPKM=.;Reads=.;Filter=!;Overlaps=.

sgr06 goRAP RF00019_Y_RNA 30573750 30573835 . - . TPM=.;FPKM=.;Reads=.;Filter=!;Note=.;Overlaps=.

sgr06 goRAP RF00019_Y_RNA 30573754 30573839 . + . TPM=.;FPKM=.;Reads=.;Filter=!;Note=.;Overlaps=RF01848_ACEA_U3,RF00012_U3

sgr06 goRAP RF00026_U6 69180805 69180910 . - . RPKM=.;Reads=.;Filter=!;Overlaps=.

sgr06 goRAP RF00172_CAESAR 74229353 74229429 . - . TPM=.;FPKM=.;Reads=.;Filter=!;Note=.;Overlaps=.

sgr06 goRAP RF00381_Antizyme_FSE 53880946 53881004 . - . RPKM=.;Reads=.;Filter=!;Overlaps=.

sgr06 goRAP RF01684_mascRNA-menRNA 17134317 17134371 . + . RPKM=.;Reads=.;Filter=X;Overlaps=.

sgr06 goRAP RF02039_SPRY4-IT1_2 51597673 51597776 . + . RPKM=.;Reads=.;Filter=P;Overlaps=.

sgr06 goRAP RF02145_MEG8_1 23771209 23771280 . + . RPKM=.;Reads=.;Filter=P;Overlaps=.

sgr06 goRAP RF02543_LSU_rRNA_eukarya 26728992 26733983 . + . RPKM=.;Reads=.;Filter=!;Overlaps=.

sgr07 goRAP RF00001_5S_rRNA 16105314 16105422 . - . RPKM=.;Reads=.;Filter=!;Overlaps=.

sgr07 goRAP RF00001_5S_rRNA 84579037 84579155 . - . RPKM=.;Reads=.;Filter=X;Overlaps=.

sgr07 goRAP RF00002_5_8S_rRNA 1845397 1845496 . - . RPKM=.;Reads=.;Filter=!;Overlaps=.

sgr07 goRAP RF00002_5_8S_rRNA 92911369 92911496 . - . RPKM=.;Reads=.;Filter=!;Overlaps=.

sgr07 goRAP RF00006_Vault 27862845 27862940 . + . RPKM=.;Reads=.;Filter=!;Overlaps=.

sgr07 goRAP RF00006_Vault 27864447 27864545 . + . RPKM=.;Reads=.;Filter=!;Overlaps=.

sgr07 goRAP RF00006_Vault 27872348 27872447 . + . RPKM=.;Reads=.;Filter=!;Overlaps=.

sgr07 goRAP RF00006_Vault 27872754 27872852 . + . RPKM=.;Reads=.;Filter=!;Overlaps=.

sgr07 goRAP RF00006_Vault 27876103 27876201 . + . RPKM=.;Reads=.;Filter=!;Overlaps=.

sgr07 goRAP RF00006_Vault 27876948 27877046 . + . RPKM=.;Reads=.;Filter=!;Overlaps=.

sgr07 goRAP RF00006_Vault 27878483 27878582 . + . RPKM=.;Reads=.;Filter=!;Overlaps=.

sgr07 goRAP RF00006_Vault 27878881 27878979 . + . RPKM=.;Reads=.;Filter=!;Overlaps=.

sgr07 goRAP RF00006_Vault 27887337 27887436 . + . RPKM=.;Reads=.;Filter=!;Overlaps=.

sgr07 goRAP RF00006_Vault 27887740 27887837 . + . RPKM=.;Reads=.;Filter=!;Overlaps=.

sgr07 goRAP RF00109_Vimentin3 40235775 40235833 . + . TPM=.;FPKM=.;Reads=.;Filter=!;Note=.;Overlaps=.

sgr07 goRAP RF00485_K_chan_RES 66120633 66120746 . - . RPKM=.;Reads=.;Filter=X;Overlaps=.

sgr07 goRAP RF00485_K_chan_RES 97854685 97854798 . - . RPKM=.;Reads=.;Filter=X;Overlaps=.

sgr07 goRAP RF00485_K_chan_RES 97889709 97889822 . - . RPKM=.;Reads=.;Filter=X;Overlaps=.

sgr07 goRAP RF01684_mascRNA-menRNA 24076634 24076688 . - . RPKM=.;Reads=.;Filter=X;Overlaps=.

sgr07 goRAP RF01684_mascRNA-menRNA 29717119 29717173 . + . RPKM=.;Reads=.;Filter=P;Overlaps=.

sgr07 goRAP RF01684_mascRNA-menRNA 33613876 33613932 . - . RPKM=.;Reads=.;Filter=!;Overlaps=.

sgr07 goRAP RF01684_mascRNA-menRNA 65465685 65465736 . - . RPKM=.;Reads=.;Filter=X;Overlaps=.

sgr07 goRAP RF01803_GABA3 2789761 2789814 . - . RPKM=.;Reads=.;Filter=!;Overlaps=.

sgr07 goRAP RF01960_SSU_rRNA_eukarya 20997513 20999411 . + . RPKM=.;Reads=.;Filter=X;Overlaps=.

sgr08 goRAP RF00019_Y_RNA 50242295 50242388 . - . RPKM=.;Reads=.;Filter=!;Overlaps=.

sgr08 goRAP RF00020_U5 42058370 42058483 . + . RPKM=.;Reads=.;Filter=!;Overlaps=.

sgr08 goRAP RF00026_U6 3366223 3366328 . + . RPKM=.;Reads=.;Filter=!;Overlaps=.

sgr08 goRAP RF00026_U6 9426094 9426200 . + . RPKM=.;Reads=.;Filter=!;Overlaps=.

sgr08 goRAP RF00485_K_chan_RES 44939418 44939531 . + . RPKM=.;Reads=.;Filter=X;Overlaps=.

sgr08 goRAP RF01684_mascRNA-menRNA 22073133 22073187 . - . RPKM=.;Reads=.;Filter=X;Overlaps=.

sgr08 goRAP RF01684_mascRNA-menRNA 54171114 54171165 . + . RPKM=.;Reads=.;Filter=X;Overlaps=.

sgr09 goRAP RF00001_5S_rRNA 20083592 20083706 . + . RPKM=.;Reads=.;Filter=!;Overlaps=.

sgr09 goRAP RF00002_5_8S_rRNA 33878302 33878400 . - . TPM=.;FPKM=.;Reads=.;Filter=!;Note=.;Overlaps=.

sgr09 goRAP RF00485_K_chan_RES 22520651 22520764 . - . RPKM=.;Reads=.;Filter=X;Overlaps=.

sgr09 goRAP RF00485_K_chan_RES 22545103 22545216 . - . RPKM=.;Reads=.;Filter=X;Overlaps=.

sgr09 goRAP RF00485_K_chan_RES 22560849 22560962 . - . RPKM=.;Reads=.;Filter=X;Overlaps=.

sgr09 goRAP RF00485_K_chan_RES 26869677 26869790 . - . RPKM=.;Reads=.;Filter=X;Overlaps=.

sgr09 goRAP RF00485_K_chan_RES 32640260 32640373 . - . RPKM=.;Reads=.;Filter=X;Overlaps=.

sgr09 goRAP RF00485_K_chan_RES 48523811 48523924 . + . RPKM=.;Reads=.;Filter=X;Overlaps=.

sgr09 goRAP RF00618_U4atac 3497106 3497235 . + . RPKM=.;Reads=.;Filter=!;Overlaps=.

sgr09 goRAP RF00619_U6atac 25132744 25132871 . + . RPKM=.;Reads=.;Filter=!;Overlaps=.

sgr10 goRAP RF00001_5S_rRNA 29842500 29842614 . + . RPKM=.;Reads=.;Filter=!;Overlaps=.

sgr10 goRAP RF00002_5_8S_rRNA 10036524 10036612 . - . RPKM=.;Reads=.;Filter=X;Overlaps=.

sgr10 goRAP RF01684_mascRNA-menRNA 34816158 34816212 . - . RPKM=.;Reads=.;Filter=X;Overlaps=.

sgr11 goRAP RF00020_U5 31260126 31260240 . + . RPKM=.;Reads=.;Filter=!;Overlaps=.

sgr11 goRAP RF00037_IRE_I 13926598 13926633 . - . RPKM=.;Reads=.;Filter=!;Overlaps=.

sgr11 goRAP RF00485_K_chan_RES 50428648 50428761 . + . RPKM=.;Reads=.;Filter=X;Overlaps=.

sgr11 goRAP RF01684_mascRNA-menRNA 12888555 12888606 . + . RPKM=.;Reads=.;Filter=X;Overlaps=.

sgr11 goRAP RF01684_mascRNA-menRNA 21956604 21956658 . - . RPKM=.;Reads=.;Filter=X;Overlaps=.

sgr12 bcheck RF00009_RNaseP_nuc 15309738 15310045 . - . RPKM=.;Reads=.;Filter=!;Overlaps=.

sgr12 goRAP RF00001_5S_rRNA 33293685 33293799 . - . RPKM=.;Reads=.;Filter=!;Overlaps=.

sgr12 goRAP RF00020_U5 11955233 11955346 . + . RPKM=.;Reads=.;Filter=!;Overlaps=.

sgr12 goRAP RF00020_U5 11956057 11956171 . + . RPKM=.;Reads=.;Filter=!;Overlaps=.

sgr12 goRAP RF00020_U5 11956955 11957067 . + . RPKM=.;Reads=.;Filter=!;Overlaps=.

sgr12 goRAP RF00026_U6 2808632 2808737 . + . RPKM=.;Reads=.;Filter=!;Overlaps=.

sgr12 goRAP RF00030_RNase_MRP 15310973 15311211 . + . RPKM=.;Reads=.;Filter=!;Overlaps=.

sgr12 goRAP RF00485_K_chan_RES 17971827 17971940 . + . RPKM=.;Reads=.;Filter=X;Overlaps=.

sgr12 goRAP RF00485_K_chan_RES 22106999 22107112 . - . RPKM=.;Reads=.;Filter=X;Overlaps=.

sgr12 goRAP RF00485_K_chan_RES 22192769 22192882 . - . RPKM=.;Reads=.;Filter=X;Overlaps=.

sgr12 goRAP RF00485_K_chan_RES 22212075 22212188 . - . RPKM=.;Reads=.;Filter=X;Overlaps=.

sgr12 goRAP RF00485_K_chan_RES 23981636 23981747 . + . RPKM=.;Reads=.;Filter=X;Overlaps=.

sgr12 goRAP RF00485_K_chan_RES 39360467 39360578 . + . RPKM=.;Reads=.;Filter=X;Overlaps=.

sgr13 goRAP RF00001_5S_rRNA 12911218 12911333 . + . RPKM=.;Reads=.;Filter=!;Overlaps=.

sgr13 goRAP RF00002_5_8S_rRNA 16999968 17000121 . - . RPKM=.;Reads=.;Filter=!;Overlaps=.

sgr13 goRAP RF00015_U4 12533910 12534050 . - . RPKM=.;Reads=.;Filter=!;Overlaps=.

sgr13 goRAP RF00015_U4 12534391 12534531 . - . RPKM=.;Reads=.;Filter=!;Overlaps=.

sgr13 goRAP RF00015_U4 12534878 12535018 . - . RPKM=.;Reads=.;Filter=!;Overlaps=.

sgr13 goRAP RF00015_U4 12536403 12536535 . - . RPKM=.;Reads=.;Filter=!;Overlaps=.

sgr13 goRAP RF00015_U4 12536882 12537022 . - . RPKM=.;Reads=.;Filter=!;Overlaps=.

sgr13 goRAP RF00015_U4 12537597 12537736 . - . RPKM=.;Reads=.;Filter=!;Overlaps=.

sgr13 goRAP RF00015_U4 12538146 12538286 . - . RPKM=.;Reads=.;Filter=!;Overlaps=.

sgr13 goRAP RF00015_U4 15708528 15708668 . + . RPKM=.;Reads=.;Filter=!;Overlaps=.

sgr13 goRAP RF00485_K_chan_RES 13273316 13273429 . + . RPKM=.;Reads=.;Filter=X;Overlaps=.

sgr13 goRAP RF00485_K_chan_RES 13278083 13278196 . + . RPKM=.;Reads=.;Filter=X;Overlaps=.

sgr13 goRAP RF00485_K_chan_RES 18950523 18950636 . + . RPKM=.;Reads=.;Filter=X;Overlaps=.

sgr13 goRAP RF02543_LSU_rRNA_eukarya 14200400 14205759 . - . RPKM=.;Reads=.;Filter=!;Overlaps=.

sgr14 goRAP RF00001_5S_rRNA 5090847 5090961 . + . RPKM=.;Reads=.;Filter=!;Overlaps=.

sgr14 goRAP RF00002_5_8S_rRNA 8782289 8782397 . + . RPKM=.;Reads=.;Filter=X;Overlaps=.

sgr14 goRAP RF00004_U2 7259082 7259272 . - . RPKM=.;Reads=.;Filter=!;Overlaps=.

sgr14 goRAP RF00548_U11 386065 386202 . - . RPKM=.;Reads=.;Filter=!;Overlaps=.

sgr14 goRAP RF01684_mascRNA-menRNA 3918395 3918449 . + .RPKM=.;Reads=.;Filter=P;Overlaps=.

sgr14 goRAP RF01684_mascRNA-menRNA 5188009 5188063 . + .RPKM=.;Reads=.;Filter=X;Overlaps=.

sgr15 goRAP RF00007_U12 69750278 69750432 . + . RPKM=.;Reads=.;Filter=!;Overlaps=.

sgr15 goRAP RF00017_Metazoa_SRP 8607987 8608284 . + . RPKM=.;Reads=.;Filter=!;Overlaps=.

sgr15 goRAP RF00019_Y_RNA 26708307 26708400 . - . TPM=.;FPKM=.;Reads=.;Filter=!;Note=.;Overlaps=.

sgr15 goRAP RF00037_IRE_I 74796263 74796296 . - . RPKM=.;Reads=.;Filter=!;Overlaps=.

sgr15 goRAP RF01684_mascRNA-menRNA 59227237 59227295 . + . RPKM=.;Reads=.;Filter=P;Overlaps=.

sgr16 goRAP RF00002_5_8S_rRNA 31139722 31139873 . - . RPKM=.;Reads=.;Filter=!;Overlaps=.

sgr16 goRAP RF00026_U6 19026058 19026164 . + . RPKM=.;Reads=.;Filter=!;Overlaps=.

sgr16 goRAP RF00037_IRE_I 6788921 6788956 . + . RPKM=.;Reads=.;Filter=!;Overlaps=.

sgr16 goRAP RF00059_TPP 27011497 27011567 . - . RPKM=.;Reads=.;Filter=!;Overlaps=.

sgr16 goRAP RF01684_mascRNA-menRNA 18031722 18031776 . + . RPKM=.;Reads=.;Filter=P;Overlaps=.

sgr16 goRAP RF01684_mascRNA-menRNA 25480346 25480397 . - . RPKM=.;Reads=.;Filter=P;Overlaps=.

sgr16 goRAP RF01684_mascRNA-menRNA 25838065 25838116 . - . RPKM=.;Reads=.;Filter=P;Overlaps=.

sgr16 goRAP RF01684_mascRNA-menRNA 25976295 25976346 . + . RPKM=.;Reads=.;Filter=X;Overlaps=.

sgr17 goRAP RF00017_Metazoa_SRP 46054378 46054675 . + . RPKM=.;Reads=.;Filter=!;Overlaps=.

sgr17 goRAP RF00017_Metazoa_SRP 48647942 48648239 . - . RPKM=.;Reads=.;Filter=!;Overlaps=.

sgr18 goRAP RF00001_5S_rRNA 2662238 2662352 . - . RPKM=.;Reads=.;Filter=!;Overlaps=.

sgr18 goRAP RF00001_5S_rRNA 4706761 4706875 . + . RPKM=.;Reads=.;Filter=!;Overlaps=.

sgr18 goRAP RF00002_5_8S_rRNA 31118644 31118743 . + . RPKM=.;Reads=.;Filter=X;Overlaps=.

sgr18 goRAP RF00020_U5 15591618 15591731 . + . RPKM=.;Reads=.;Filter=!;Overlaps=.

sgr18 goRAP RF00020_U5 19215979 19216090 . + . RPKM=.;Reads=.;Filter=!;Overlaps=.

sgr18 goRAP RF00020_U5 19217848 19217960 . + . RPKM=.;Reads=.;Filter=!;Overlaps=.

sgr18 goRAP RF00485_K_chan_RES 31519651 31519764 . + . RPKM=.;Reads=.;Filter=X;Overlaps=.

sgr18 goRAP RF00485_K_chan_RES 31567499 31567612 . + . RPKM=.;Reads=.;Filter=X;Overlaps=.

sgr18 goRAP RF01455_DPB 31304719 31304796 . + . RPKM=.;Reads=.;Filter=X;Overlaps=.

sgr18 goRAP RF01684_mascRNA-menRNA 14412946 14413000 . - . RPKM=.;Reads=.;Filter=X;Overlaps=.

sgr18 goRAP RF01684_mascRNA-menRNA 26257685 26257739 . + . RPKM=.;Reads=.;Filter=P;Overlaps=.

sgr18 goRAP RF01684_mascRNA-menRNA 26280541 26280595 . - . RPKM=.;Reads=.;Filter=X;Overlaps=.

sgr19 goRAP RF00001_5S_rRNA 19173567 19173685 . + . RPKM=.;Reads=.;Filter=X;Overlaps=.

sgr19 goRAP RF00001_5S_rRNA 36652278 36652396 . + . RPKM=.;Reads=.;Filter=X;Overlaps=.

sgr19 goRAP RF01684_mascRNA-menRNA 19849233 19849283 . + . RPKM=.;Reads=.;Filter=X;Overlaps=.

sgr19 goRAP RF01684_mascRNA-menRNA 19852233 19852284 . - . RPKM=.;Reads=.;Filter=X;Overlaps=.

sgr19 goRAP RF01684_mascRNA-menRNA 19856171 19856222 . - . RPKM=.;Reads=.;Filter=X;Overlaps=.

sgr19 goRAP RF01684_mascRNA-menRNA 22296637 22296688 . + . RPKM=.;Reads=.;Filter=X;Overlaps=.

sgr19 goRAP RF01684_mascRNA-menRNA 22301072 22301126 . - . RPKM=.;Reads=.;Filter=P;Overlaps=.

sgr19 goRAP RF01684_mascRNA-menRNA 24425224 24425275 . - . RPKM=.;Reads=.;Filter=X;Overlaps=.

sgr19 goRAP RF01684_mascRNA-menRNA 27840519 27840573 . + . RPKM=.;Reads=.;Filter=X;Overlaps=.

sgr19 goRAP RF01684_mascRNA-menRNA 33227819 33227873 . - . RPKM=.;Reads=.;Filter=X;Overlaps=.

sgr19 goRAP RF01684_mascRNA-menRNA 33230018 33230072 . + . RPKM=.;Reads=.;Filter=X;Overlaps=.

sgr19 goRAP RF02543_LSU_rRNA_eukarya 2486524 2493279 . + . RPKM=.;Reads=.;Filter=!;Overlaps=.
