# Supplementary material for: A miRNA catalogue and ncRNA annotation of the short-living fish Nothobranchius furzeri
Source: BMC Genomics. 2017 Sep 5;18:693. doi: 10.1186/s12864-017-3951-8 (PMC5584509; doi:10.1186/s12864-017-3951-8)
Supplement: Supplementary file 2 — Annotation of all identified pre-miRNAs and corresponding mature miRNAs in Danio rerio in gff format. (DOCX 48 kb) [file 12864_2017_3951_MOESM2_ESM.docx]

scaffold00003 nfu_mirna_prediction pre_miRNA 206761 206849 . + . ID=nfu_pred_1;biotype=pre-miRNA;gene=mir-204;geneid=nfu_pred_1_mir-204;coords=scaffold00003:206761-206849

scaffold00003 nfu_mirna_prediction mature_miRNA 206777 206801 . + . ID=nfu_pred_1;biotype=5'-mature-miRNA;gene=mir-204;geneid=nfu_pred_1_mir-204;coords=scaffold00003:206777-206801

scaffold00003 nfu_mirna_prediction mature_miRNA 206818 206838 . + . ID=nfu_pred_1;biotype=3'-mature-miRNA;gene=mir-204;geneid=nfu_pred_1_mir-204;coords=scaffold00003:206818-206838

scaffold00003 nfu_mirna_prediction pre_miRNA 318191 318290 . + . ID=nfu_pred_2;biotype=pre-miRNA;gene=mir-188;geneid=nfu_pred_2_mir-188;coords=scaffold00003:318191-318290

scaffold00003 nfu_mirna_prediction mature_miRNA 318201 318224 . + . ID=nfu_pred_2;biotype=5'-mature-miRNA;gene=mir-188;geneid=nfu_pred_2_mir-188;coords=scaffold00003:318201-318224

scaffold00003 nfu_mirna_prediction mature_miRNA 318238 318259 . + . ID=nfu_pred_2;biotype=3'-mature-miRNA;gene=mir-188;geneid=nfu_pred_2_mir-188;coords=scaffold00003:318238-318259

scaffold00012 nfu_mirna_prediction pre_miRNA 92419 92468 . - . ID=nfu_pred_3;biotype=pre-miRNA;gene=mir-4507;geneid=nfu_pred_3_mir-4507;coords=scaffold00012:92419-92468

scaffold00029 nfu_mirna_prediction pre_miRNA 149993 150073 . - . ID=nfu_pred_4;biotype=pre-miRNA;gene=mir-138;geneid=nfu_pred_4_mir-138;coords=scaffold00029:149993-150073

scaffold00029 nfu_mirna_prediction mature_miRNA 150041 150063 . - . ID=nfu_pred_4;biotype=5'-mature-miRNA;gene=mir-138;geneid=nfu_pred_4_mir-138;coords=scaffold00029:150041-150063

scaffold00032 nfu_mirna_prediction pre_miRNA 200628 200711 . + . ID=nfu_pred_5;biotype=pre-miRNA;gene=mir-3532;geneid=nfu_pred_5_mir-3532;coords=scaffold00032:200628-200711

scaffold00033 nfu_mirna_prediction pre_miRNA 128004 128080 . - . ID=nfu_pred_6;biotype=pre-miRNA;gene=mir-609;geneid=nfu_pred_6_mir-609;coords=scaffold00033:128004-128080

scaffold00033 nfu_mirna_prediction mature_miRNA 128048 128070 . - . ID=nfu_pred_6;biotype=5'-mature-miRNA;gene=mir-609;geneid=nfu_pred_6_mir-609;coords=scaffold00033:128048-128070

scaffold00033 nfu_mirna_prediction mature_miRNA 128010 128030 . - . ID=nfu_pred_6;biotype=3'-mature-miRNA;gene=mir-609;geneid=nfu_pred_6_mir-609;coords=scaffold00033:128010-128030

scaffold00042 nfu_mirna_prediction pre_miRNA 186335 186437 . - . ID=nfu_pred_7;biotype=pre-miRNA;gene=let-7;geneid=nfu_pred_7_let-7;coords=scaffold00042:186335-186437

scaffold00042 nfu_mirna_prediction mature_miRNA 186401 186422 . - . ID=nfu_pred_7;biotype=5'-mature-miRNA;gene=let-7;geneid=nfu_pred_7_let-7;coords=scaffold00042:186401-186422

scaffold00042 nfu_mirna_prediction mature_miRNA 186446 186467 . - . ID=nfu_pred_7;biotype=3'-mature-miRNA;gene=let-7;geneid=nfu_pred_7_let-7;coords=scaffold00042:186446-186467

scaffold00042 nfu_mirna_prediction pre_miRNA 203749 203828 . - . ID=nfu_pred_8;biotype=pre-miRNA;gene=mir-101;geneid=nfu_pred_8_mir-101;coords=scaffold00042:203749-203828

scaffold00042 nfu_mirna_prediction mature_miRNA 203795 203816 . - . ID=nfu_pred_8;biotype=5'-mature-miRNA;gene=mir-101;geneid=nfu_pred_8_mir-101;coords=scaffold00042:203795-203816

scaffold00042 nfu_mirna_prediction mature_miRNA 203760 203782 . - . ID=nfu_pred_8;biotype=3'-mature-miRNA;gene=mir-101;geneid=nfu_pred_8_mir-101;coords=scaffold00042:203760-203782

scaffold00056 nfu_mirna_prediction pre_miRNA 180351 180413 . + . ID=nfu_pred_9;biotype=pre-miRNA;gene=;geneid=nfu_pred_9_;coords=scaffold00056:180351-180413

scaffold00059 nfu_mirna_prediction pre_miRNA 68222 68311 . + . ID=nfu_pred_10;biotype=pre-miRNA;gene=mir-581;geneid=nfu_pred_10_mir-581;coords=scaffold00059:68222-68311

scaffold00059 nfu_mirna_prediction mature_miRNA 68235 68258 . + . ID=nfu_pred_10;biotype=5'-mature-miRNA;gene=mir-581;geneid=nfu_pred_10_mir-581;coords=scaffold00059:68235-68258

scaffold00059 nfu_mirna_prediction mature_miRNA 68271 68293 . + . ID=nfu_pred_10;biotype=3'-mature-miRNA;gene=mir-581;geneid=nfu_pred_10_mir-581;coords=scaffold00059:68271-68293

scaffold00065 nfu_mirna_prediction pre_miRNA 118058 118142 . - . ID=nfu_pred_11;biotype=pre-miRNA;gene=mir-9;geneid=nfu_pred_11_mir-9;coords=scaffold00065:118058-118142

scaffold00065 nfu_mirna_prediction mature_miRNA 118106 118128 . - . ID=nfu_pred_11;biotype=5'-mature-miRNA;gene=mir-9;geneid=nfu_pred_11_mir-9;coords=scaffold00065:118106-118128

scaffold00065 nfu_mirna_prediction mature_miRNA 118069 118090 . - . ID=nfu_pred_11;biotype=3'-mature-miRNA;gene=mir-9;geneid=nfu_pred_11_mir-9;coords=scaffold00065:118069-118090

scaffold00073 nfu_mirna_prediction pre_miRNA 133679 133741 . + . ID=nfu_pred_12;biotype=pre-miRNA;gene=;geneid=nfu_pred_12_;coords=scaffold00073:133679-133741

scaffold00078 nfu_mirna_prediction pre_miRNA 160024 160102 . + . ID=nfu_pred_13;biotype=pre-miRNA;gene=;geneid=nfu_pred_13_;coords=scaffold00078:160024-160102

scaffold00078 nfu_mirna_prediction mature_miRNA 160070 160092 . + . ID=nfu_pred_13;biotype=3'-mature-miRNA;gene=;geneid=nfu_pred_13_;coords=scaffold00078:160070-160092

scaffold00079 nfu_mirna_prediction pre_miRNA 29146 29243 . - . ID=nfu_pred_14;biotype=pre-miRNA;gene=mir-6720;geneid=nfu_pred_14_mir-6720;coords=scaffold00079:29146-29243

scaffold00083 nfu_mirna_prediction pre_miRNA 70461 70548 . - . ID=nfu_pred_15;biotype=pre-miRNA;gene=mir-187;geneid=nfu_pred_15_mir-187;coords=scaffold00083:70461-70548

scaffold00083 nfu_mirna_prediction mature_miRNA 70513 70535 . - . ID=nfu_pred_15;biotype=5'-mature-miRNA;gene=mir-187;geneid=nfu_pred_15_mir-187;coords=scaffold00083:70513-70535

scaffold00084 nfu_mirna_prediction pre_miRNA 107302 107378 . + . ID=nfu_pred_16;biotype=pre-miRNA;gene=;geneid=nfu_pred_16_;coords=scaffold00084:107302-107378

scaffold00084 nfu_mirna_prediction mature_miRNA 107308 107332 . + . ID=nfu_pred_16;biotype=5'-mature-miRNA;gene=;geneid=nfu_pred_16_;coords=scaffold00084:107308-107332

scaffold00084 nfu_mirna_prediction mature_miRNA 107349 107368 . + . ID=nfu_pred_16;biotype=3'-mature-miRNA;gene=;geneid=nfu_pred_16_;coords=scaffold00084:107349-107368

scaffold00089 nfu_mirna_prediction pre_miRNA 127620 127696 . + . ID=nfu_pred_17;biotype=pre-miRNA;gene=;geneid=nfu_pred_17_;coords=scaffold00089:127620-127696

scaffold00089 nfu_mirna_prediction mature_miRNA 127632 127654 . + . ID=nfu_pred_17;biotype=5'-mature-miRNA;gene=;geneid=nfu_pred_17_;coords=scaffold00089:127632-127654

scaffold00089 nfu_mirna_prediction mature_miRNA 127665 127686 . + . ID=nfu_pred_17;biotype=3'-mature-miRNA;gene=;geneid=nfu_pred_17_;coords=scaffold00089:127665-127686

scaffold00089 nfu_mirna_prediction pre_miRNA 133819 133913 . + . ID=nfu_pred_18;biotype=pre-miRNA;gene=mir-148;geneid=nfu_pred_18_mir-148;coords=scaffold00089:133819-133913

scaffold00089 nfu_mirna_prediction mature_miRNA 133838 133860 . + . ID=nfu_pred_18;biotype=5'-mature-miRNA;gene=mir-148;geneid=nfu_pred_18_mir-148;coords=scaffold00089:133838-133860

scaffold00089 nfu_mirna_prediction mature_miRNA 133876 133897 . + . ID=nfu_pred_18;biotype=3'-mature-miRNA;gene=mir-148;geneid=nfu_pred_18_mir-148;coords=scaffold00089:133876-133897

scaffold00099 nfu_mirna_prediction pre_miRNA 107332 107414 . - . ID=nfu_pred_822;biotype=pre-miRNA;gene=mir-150;geneid=nfu_pred_822_mir-150;coords=scaffold00099:107332-107414

scaffold00125 nfu_mirna_prediction pre_miRNA 155987 156066 . + . ID=nfu_pred_20;biotype=pre-miRNA;gene=let-7;geneid=nfu_pred_20_let-7;coords=scaffold00125:155987-156066

scaffold00125 nfu_mirna_prediction mature_miRNA 155993 156015 . + . ID=nfu_pred_20;biotype=5'-mature-miRNA;gene=let-7;geneid=nfu_pred_20_let-7;coords=scaffold00125:155993-156015

scaffold00125 nfu_mirna_prediction mature_miRNA 156041 156062 . + . ID=nfu_pred_20;biotype=3'-mature-miRNA;gene=let-7;geneid=nfu_pred_20_let-7;coords=scaffold00125:156041-156062

scaffold00125 nfu_mirna_prediction pre_miRNA 163330 163408 . + . ID=nfu_pred_21;biotype=pre-miRNA;gene=lin-4;geneid=nfu_pred_21_lin-4;coords=scaffold00125:163330-163408

scaffold00125 nfu_mirna_prediction mature_miRNA 163340 163362 . + . ID=nfu_pred_21;biotype=5'-mature-miRNA;gene=lin-4;geneid=nfu_pred_21_lin-4;coords=scaffold00125:163340-163362

scaffold00127 nfu_mirna_prediction pre_miRNA 64515 64613 . + . ID=nfu_pred_22;biotype=pre-miRNA;gene=mir-1338;geneid=nfu_pred_22_mir-1338;coords=scaffold00127:64515-64613

scaffold00127 nfu_mirna_prediction mature_miRNA 64575 64596 . + . ID=nfu_pred_22;biotype=3'-mature-miRNA;gene=mir-1338;geneid=nfu_pred_22_mir-1338;coords=scaffold00127:64575-64596

scaffold00132 nfu_mirna_prediction pre_miRNA 82541 82621 . + . ID=nfu_pred_23;biotype=pre-miRNA;gene=mir-130;geneid=nfu_pred_23_mir-130;coords=scaffold00132:82541-82621

scaffold00132 nfu_mirna_prediction mature_miRNA 82553 82574 . + . ID=nfu_pred_23;biotype=5'-mature-miRNA;gene=mir-130;geneid=nfu_pred_23_mir-130;coords=scaffold00132:82553-82574

scaffold00132 nfu_mirna_prediction mature_miRNA 82589 82611 . + . ID=nfu_pred_23;biotype=3'-mature-miRNA;gene=mir-130;geneid=nfu_pred_23_mir-130;coords=scaffold00132:82589-82611

scaffold00132 nfu_mirna_prediction pre_miRNA 82772 82856 . + . ID=nfu_pred_24;biotype=pre-miRNA;gene=mir-301;geneid=nfu_pred_24_mir-301;coords=scaffold00132:82772-82856

scaffold00132 nfu_mirna_prediction mature_miRNA 82783 82804 . + . ID=nfu_pred_24;biotype=5'-mature-miRNA;gene=mir-301;geneid=nfu_pred_24_mir-301;coords=scaffold00132:82783-82804

scaffold00132 nfu_mirna_prediction mature_miRNA 82821 82843 . + . ID=nfu_pred_24;biotype=3'-mature-miRNA;gene=mir-301;geneid=nfu_pred_24_mir-301;coords=scaffold00132:82821-82843

scaffold00132 nfu_mirna_prediction pre_miRNA 95320 95444 . + . ID=nfu_pred_25;biotype=pre-miRNA;gene=mir-454;geneid=nfu_pred_25_mir-454;coords=scaffold00132:95320-95444

scaffold00132 nfu_mirna_prediction mature_miRNA 95351 95373 . + . ID=nfu_pred_25;biotype=5'-mature-miRNA;gene=mir-454;geneid=nfu_pred_25_mir-454;coords=scaffold00132:95351-95373

scaffold00132 nfu_mirna_prediction mature_miRNA 95393 95416 . + . ID=nfu_pred_25;biotype=3'-mature-miRNA;gene=mir-454;geneid=nfu_pred_25_mir-454;coords=scaffold00132:95393-95416

scaffold00132 nfu_mirna_prediction pre_miRNA 97419 97497 . + . ID=nfu_pred_26;biotype=pre-miRNA;gene=mir-130;geneid=nfu_pred_26_mir-130;coords=scaffold00132:97419-97497

scaffold00132 nfu_mirna_prediction mature_miRNA 97427 97448 . + . ID=nfu_pred_26;biotype=5'-mature-miRNA;gene=mir-130;geneid=nfu_pred_26_mir-130;coords=scaffold00132:97427-97448

scaffold00132 nfu_mirna_prediction mature_miRNA 97466 97488 . + . ID=nfu_pred_26;biotype=3'-mature-miRNA;gene=mir-130;geneid=nfu_pred_26_mir-130;coords=scaffold00132:97466-97488

scaffold00135 nfu_mirna_prediction pre_miRNA 169895 169958 . + . ID=nfu_pred_27;biotype=pre-miRNA;gene=mir-155;geneid=nfu_pred_27_mir-155;coords=scaffold00135:169895-169958

scaffold00149 nfu_mirna_prediction pre_miRNA 137768 137859 . - . ID=nfu_pred_28;biotype=pre-miRNA;gene=mir-49;geneid=nfu_pred_28_mir-49;coords=scaffold00149:137768-137859

scaffold00149 nfu_mirna_prediction mature_miRNA 137818 137839 . - . ID=nfu_pred_28;biotype=5'-mature-miRNA;gene=mir-49;geneid=nfu_pred_28_mir-49;coords=scaffold00149:137818-137839

scaffold00149 nfu_mirna_prediction mature_miRNA 137783 137804 . - . ID=nfu_pred_28;biotype=3'-mature-miRNA;gene=mir-49;geneid=nfu_pred_28_mir-49;coords=scaffold00149:137783-137804

scaffold00150 nfu_mirna_prediction pre_miRNA 161350 161434 . + . ID=nfu_pred_29;biotype=pre-miRNA;gene=;geneid=nfu_pred_29_;coords=scaffold00150:161350-161434

scaffold00152 nfu_mirna_prediction pre_miRNA 44194 44303 . + . ID=nfu_pred_30;biotype=pre-miRNA;gene=mir-187;geneid=nfu_pred_30_mir-187;coords=scaffold00152:44194-44303

scaffold00152 nfu_mirna_prediction mature_miRNA 44220 44241 . + . ID=nfu_pred_30;biotype=5'-mature-miRNA;gene=mir-187;geneid=nfu_pred_30_mir-187;coords=scaffold00152:44220-44241

scaffold00152 nfu_mirna_prediction mature_miRNA 44261 44283 . + . ID=nfu_pred_30;biotype=3'-mature-miRNA;gene=mir-187;geneid=nfu_pred_30_mir-187;coords=scaffold00152:44261-44283

scaffold00155 nfu_mirna_prediction pre_miRNA 138655 138733 . + . ID=nfu_pred_32;biotype=pre-miRNA;gene=;geneid=nfu_pred_32_;coords=scaffold00155:138655-138733

scaffold00155 nfu_mirna_prediction mature_miRNA 138667 138687 . + . ID=nfu_pred_32;biotype=5'-mature-miRNA;gene=;geneid=nfu_pred_32_;coords=scaffold00155:138667-138687

scaffold00155 nfu_mirna_prediction mature_miRNA 138703 138723 . + . ID=nfu_pred_32;biotype=3'-mature-miRNA;gene=;geneid=nfu_pred_32_;coords=scaffold00155:138703-138723

scaffold00160 nfu_mirna_prediction pre_miRNA 88552 88635 . + . ID=nfu_pred_33;biotype=pre-miRNA;gene=mir-723;geneid=nfu_pred_33_mir-723;coords=scaffold00160:88552-88635

scaffold00160 nfu_mirna_prediction mature_miRNA 88603 88624 . + . ID=nfu_pred_33;biotype=3'-mature-miRNA;gene=mir-723;geneid=nfu_pred_33_mir-723;coords=scaffold00160:88603-88624

scaffold00162 nfu_mirna_prediction pre_miRNA 158967 159049 . - . ID=nfu_pred_34;biotype=pre-miRNA;gene=mir-471;geneid=nfu_pred_34_mir-471;coords=scaffold00162:158967-159049

scaffold00162 nfu_mirna_prediction mature_miRNA 159014 159036 . - . ID=nfu_pred_34;biotype=5'-mature-miRNA;gene=mir-471;geneid=nfu_pred_34_mir-471;coords=scaffold00162:159014-159036

scaffold00162 nfu_mirna_prediction mature_miRNA 158978 159000 . - . ID=nfu_pred_34;biotype=3'-mature-miRNA;gene=mir-471;geneid=nfu_pred_34_mir-471;coords=scaffold00162:158978-159000

scaffold00168 nfu_mirna_prediction pre_miRNA 80308 80390 . - . ID=nfu_pred_35;biotype=pre-miRNA;gene=;geneid=nfu_pred_35_;coords=scaffold00168:80308-80390

scaffold00168 nfu_mirna_prediction mature_miRNA 80318 80341 . - . ID=nfu_pred_35;biotype=3'-mature-miRNA;gene=;geneid=nfu_pred_35_;coords=scaffold00168:80318-80341

scaffold00210 nfu_mirna_prediction pre_miRNA 56535 56616 . - . ID=nfu_pred_38;biotype=pre-miRNA;gene=mir-122;geneid=nfu_pred_38_mir-122;coords=scaffold00210:56535-56616

scaffold00210 nfu_mirna_prediction mature_miRNA 56583 56604 . - . ID=nfu_pred_38;biotype=5'-mature-miRNA;gene=mir-122;geneid=nfu_pred_38_mir-122;coords=scaffold00210:56583-56604

scaffold00210 nfu_mirna_prediction mature_miRNA 56545 56565 . - . ID=nfu_pred_38;biotype=3'-mature-miRNA;gene=mir-122;geneid=nfu_pred_38_mir-122;coords=scaffold00210:56545-56565

scaffold00223 nfu_mirna_prediction pre_miRNA 86722 86800 . + . ID=nfu_pred_39;biotype=pre-miRNA;gene=;geneid=nfu_pred_39_;coords=scaffold00223:86722-86800

scaffold00223 nfu_mirna_prediction mature_miRNA 86732 86753 . + . ID=nfu_pred_39;biotype=5'-mature-miRNA;gene=;geneid=nfu_pred_39_;coords=scaffold00223:86732-86753

scaffold00223 nfu_mirna_prediction mature_miRNA 86768 86791 . + . ID=nfu_pred_39;biotype=3'-mature-miRNA;gene=;geneid=nfu_pred_39_;coords=scaffold00223:86768-86791

scaffold00245 nfu_mirna_prediction pre_miRNA 74071 74152 . + . ID=nfu_pred_41;biotype=pre-miRNA;gene=mir-423;geneid=nfu_pred_41_mir-423;coords=scaffold00245:74071-74152

scaffold00245 nfu_mirna_prediction mature_miRNA 74120 74141 . + . ID=nfu_pred_41;biotype=3'-mature-miRNA;gene=mir-423;geneid=nfu_pred_41_mir-423;coords=scaffold00245:74120-74141

scaffold00253 nfu_mirna_prediction pre_miRNA 17971 18062 . - . ID=nfu_pred_42;biotype=pre-miRNA;gene=mir-4536;geneid=nfu_pred_42_mir-4536;coords=scaffold00253:17971-18062

scaffold00253 nfu_mirna_prediction pre_miRNA 18257 18362 . + . ID=nfu_pred_43;biotype=pre-miRNA;gene=mir-4536;geneid=nfu_pred_43_mir-4536;coords=scaffold00253:18257-18362

scaffold00258 nfu_mirna_prediction pre_miRNA 65404 65480 . + . ID=nfu_pred_44;biotype=pre-miRNA;gene=mir-315;geneid=nfu_pred_44_mir-315;coords=scaffold00258:65404-65480

scaffold00258 nfu_mirna_prediction mature_miRNA 65408 65430 . + . ID=nfu_pred_44;biotype=5'-mature-miRNA;gene=mir-315;geneid=nfu_pred_44_mir-315;coords=scaffold00258:65408-65430

scaffold00258 nfu_mirna_prediction mature_miRNA 65450 65470 . + . ID=nfu_pred_44;biotype=3'-mature-miRNA;gene=mir-315;geneid=nfu_pred_44_mir-315;coords=scaffold00258:65450-65470

scaffold00259 nfu_mirna_prediction pre_miRNA 11794 11895 . - . ID=nfu_pred_45;biotype=pre-miRNA;gene=mir-674;geneid=nfu_pred_45_mir-674;coords=scaffold00259:11794-11895

scaffold00268 nfu_mirna_prediction pre_miRNA 53196 53274 . - . ID=nfu_pred_46;biotype=pre-miRNA;gene=mir-944;geneid=nfu_pred_46_mir-944;coords=scaffold00268:53196-53274

scaffold00268 nfu_mirna_prediction mature_miRNA 53241 53264 . - . ID=nfu_pred_46;biotype=5'-mature-miRNA;gene=mir-944;geneid=nfu_pred_46_mir-944;coords=scaffold00268:53241-53264

scaffold00268 nfu_mirna_prediction mature_miRNA 53207 53228 . - . ID=nfu_pred_46;biotype=3'-mature-miRNA;gene=mir-944;geneid=nfu_pred_46_mir-944;coords=scaffold00268:53207-53228

scaffold00287 nfu_mirna_prediction pre_miRNA 77579 77657 . + . ID=nfu_pred_47;biotype=pre-miRNA;gene=mir-370;geneid=nfu_pred_47_mir-370;coords=scaffold00287:77579-77657

scaffold00287 nfu_mirna_prediction mature_miRNA 77589 77610 . + . ID=nfu_pred_47;biotype=5'-mature-miRNA;gene=mir-370;geneid=nfu_pred_47_mir-370;coords=scaffold00287:77589-77610

scaffold00287 nfu_mirna_prediction mature_miRNA 77625 77646 . + . ID=nfu_pred_47;biotype=3'-mature-miRNA;gene=mir-370;geneid=nfu_pred_47_mir-370;coords=scaffold00287:77625-77646

scaffold00323 nfu_mirna_prediction pre_miRNA 119435 119538 . - . ID=nfu_pred_48;biotype=pre-miRNA;gene=mir-2985-2;geneid=nfu_pred_48_mir-2985-2;coords=scaffold00323:119435-119538

scaffold00323 nfu_mirna_prediction mature_miRNA 119487 119510 . - . ID=nfu_pred_48;biotype=5'-mature-miRNA;gene=mir-2985-2;geneid=nfu_pred_48_mir-2985-2;coords=scaffold00323:119487-119510

scaffold00323 nfu_mirna_prediction mature_miRNA 119556 119577 . - . ID=nfu_pred_48;biotype=3'-mature-miRNA;gene=mir-2985-2;geneid=nfu_pred_48_mir-2985-2;coords=scaffold00323:119556-119577

scaffold00324 nfu_mirna_prediction pre_miRNA 908 1011 . + . ID=nfu_pred_49;biotype=pre-miRNA;gene=mir-2187;geneid=nfu_pred_49_mir-2187;coords=scaffold00324:908-1011

scaffold00341 nfu_mirna_prediction pre_miRNA 28270 28350 . + . ID=nfu_pred_50;biotype=pre-miRNA;gene=mir-185;geneid=nfu_pred_50_mir-185;coords=scaffold00341:28270-28350

scaffold00341 nfu_mirna_prediction mature_miRNA 28280 28302 . + . ID=nfu_pred_50;biotype=5'-mature-miRNA;gene=mir-185;geneid=nfu_pred_50_mir-185;coords=scaffold00341:28280-28302

scaffold00341 nfu_mirna_prediction mature_miRNA 28315 28336 . + . ID=nfu_pred_50;biotype=3'-mature-miRNA;gene=mir-185;geneid=nfu_pred_50_mir-185;coords=scaffold00341:28315-28336

scaffold00344 nfu_mirna_prediction pre_miRNA 94405 94492 . - . ID=nfu_pred_51;biotype=pre-miRNA;gene=mir-187;geneid=nfu_pred_51_mir-187;coords=scaffold00344:94405-94492

scaffold00344 nfu_mirna_prediction mature_miRNA 94416 94438 . - . ID=nfu_pred_51;biotype=3'-mature-miRNA;gene=mir-187;geneid=nfu_pred_51_mir-187;coords=scaffold00344:94416-94438

scaffold00364 nfu_mirna_prediction pre_miRNA 198 276 . + . ID=nfu_pred_52;biotype=pre-miRNA;gene=mir-430;geneid=nfu_pred_52_mir-430;coords=scaffold00364:198-276

scaffold00364 nfu_mirna_prediction pre_miRNA 450 540 . + . ID=nfu_pred_54;biotype=pre-miRNA;gene=mir-430;geneid=nfu_pred_54_mir-430;coords=scaffold00364:450-540

scaffold00364 nfu_mirna_prediction mature_miRNA 471 491 . + . ID=nfu_pred_54;biotype=5'-mature-miRNA;gene=mir-430;geneid=nfu_pred_54_mir-430;coords=scaffold00364:471-491

scaffold00364 nfu_mirna_prediction mature_miRNA 508 529 . + . ID=nfu_pred_54;biotype=3'-mature-miRNA;gene=mir-430;geneid=nfu_pred_54_mir-430;coords=scaffold00364:508-529

scaffold00383 nfu_mirna_prediction pre_miRNA 75389 75467 . + . ID=nfu_pred_55;biotype=pre-miRNA;gene=;geneid=nfu_pred_55_;coords=scaffold00383:75389-75467

scaffold00383 nfu_mirna_prediction mature_miRNA 75436 75457 . + . ID=nfu_pred_55;biotype=3'-mature-miRNA;gene=;geneid=nfu_pred_55_;coords=scaffold00383:75436-75457

scaffold00389 nfu_mirna_prediction pre_miRNA 63910 64010 . - . ID=nfu_pred_56;biotype=pre-miRNA;gene=mir-605;geneid=nfu_pred_56_mir-605;coords=scaffold00389:63910-64010

scaffold00389 nfu_mirna_prediction mature_miRNA 63930 63950 . - . ID=nfu_pred_56;biotype=5'-mature-miRNA;gene=mir-605;geneid=nfu_pred_56_mir-605;coords=scaffold00389:63930-63950

scaffold00389 nfu_mirna_prediction mature_miRNA 63969 63990 . - . ID=nfu_pred_56;biotype=3'-mature-miRNA;gene=mir-605;geneid=nfu_pred_56_mir-605;coords=scaffold00389:63969-63990

scaffold00415 nfu_mirna_prediction pre_miRNA 71920 72007 . - . ID=nfu_pred_57;biotype=pre-miRNA;gene=mir-187;geneid=nfu_pred_57_mir-187;coords=scaffold00415:71920-72007

scaffold00415 nfu_mirna_prediction mature_miRNA 71972 71992 . - . ID=nfu_pred_57;biotype=5'-mature-miRNA;gene=mir-187;geneid=nfu_pred_57_mir-187;coords=scaffold00415:71972-71992

scaffold00415 nfu_mirna_prediction mature_miRNA 71932 71955 . - . ID=nfu_pred_57;biotype=3'-mature-miRNA;gene=mir-187;geneid=nfu_pred_57_mir-187;coords=scaffold00415:71932-71955

scaffold00423 nfu_mirna_prediction pre_miRNA 91310 91391 . - . ID=nfu_pred_58;biotype=pre-miRNA;gene=mir-3179;geneid=nfu_pred_58_mir-3179;coords=scaffold00423:91310-91391

scaffold00423 nfu_mirna_prediction mature_miRNA 91359 91380 . - . ID=nfu_pred_58;biotype=5'-mature-miRNA;gene=mir-3179;geneid=nfu_pred_58_mir-3179;coords=scaffold00423:91359-91380

scaffold00423 nfu_mirna_prediction mature_miRNA 91323 91344 . - . ID=nfu_pred_58;biotype=3'-mature-miRNA;gene=mir-3179;geneid=nfu_pred_58_mir-3179;coords=scaffold00423:91323-91344

scaffold00441 nfu_mirna_prediction pre_miRNA 17515 17620 . - . ID=nfu_pred_59;biotype=pre-miRNA;gene=mir-821;geneid=nfu_pred_59_mir-821;coords=scaffold00441:17515-17620

scaffold00459 nfu_mirna_prediction pre_miRNA 41461 41540 . + . ID=nfu_pred_60;biotype=pre-miRNA;gene=mir-22;geneid=nfu_pred_60_mir-22;coords=scaffold00459:41461-41540

scaffold00459 nfu_mirna_prediction mature_miRNA 41508 41529 . + . ID=nfu_pred_60;biotype=3'-mature-miRNA;gene=mir-22;geneid=nfu_pred_60_mir-22;coords=scaffold00459:41508-41529

scaffold00460 nfu_mirna_prediction pre_miRNA 65459 65537 . + . ID=nfu_pred_61;biotype=pre-miRNA;gene=mir-42;geneid=nfu_pred_61_mir-42;coords=scaffold00460:65459-65537

scaffold00460 nfu_mirna_prediction mature_miRNA 65469 65490 . + . ID=nfu_pred_61;biotype=5'-mature-miRNA;gene=mir-42;geneid=nfu_pred_61_mir-42;coords=scaffold00460:65469-65490

scaffold00460 nfu_mirna_prediction mature_miRNA 65503 65524 . + . ID=nfu_pred_61;biotype=3'-mature-miRNA;gene=mir-42;geneid=nfu_pred_61_mir-42;coords=scaffold00460:65503-65524

scaffold00462 nfu_mirna_prediction pre_miRNA 32815 32906 . + . ID=nfu_pred_823;biotype=pre-miRNA;gene=mir-153;geneid=nfu_pred_823_mir-153;coords=scaffold00462:32815-32906

scaffold00486 nfu_mirna_prediction pre_miRNA 11104 11176 . + . ID=nfu_pred_62;biotype=pre-miRNA;gene=mir-219;geneid=nfu_pred_62_mir-219;coords=scaffold00486:11104-11176

scaffold00486 nfu_mirna_prediction mature_miRNA 11151 11173 . + . ID=nfu_pred_62;biotype=3'-mature-miRNA;gene=mir-219;geneid=nfu_pred_62_mir-219;coords=scaffold00486:11151-11173

scaffold00587 nfu_mirna_prediction pre_miRNA 11208 11286 . - . ID=nfu_pred_64;biotype=pre-miRNA;gene=;geneid=nfu_pred_64_;coords=scaffold00587:11208-11286

scaffold00588 nfu_mirna_prediction pre_miRNA 14350 14430 . - . ID=nfu_pred_65;biotype=pre-miRNA;gene=mir-396;geneid=nfu_pred_65_mir-396;coords=scaffold00588:14350-14430

scaffold00588 nfu_mirna_prediction mature_miRNA 14404 14426 . - . ID=nfu_pred_65;biotype=5'-mature-miRNA;gene=mir-396;geneid=nfu_pred_65_mir-396;coords=scaffold00588:14404-14426

scaffold00588 nfu_mirna_prediction mature_miRNA 14364 14385 . - . ID=nfu_pred_65;biotype=3'-mature-miRNA;gene=mir-396;geneid=nfu_pred_65_mir-396;coords=scaffold00588:14364-14385

scaffold00594 nfu_mirna_prediction pre_miRNA 18999 19061 . + . ID=nfu_pred_66;biotype=pre-miRNA;gene=;geneid=nfu_pred_66_;coords=scaffold00594:18999-19061

scaffold00613 nfu_mirna_prediction pre_miRNA 47244 47338 . - . ID=nfu_pred_68;biotype=pre-miRNA;gene=mir-230;geneid=nfu_pred_68_mir-230;coords=scaffold00613:47244-47338

scaffold00613 nfu_mirna_prediction mature_miRNA 47308 47329 . - . ID=nfu_pred_68;biotype=5'-mature-miRNA;gene=mir-230;geneid=nfu_pred_68_mir-230;coords=scaffold00613:47308-47329

scaffold00613 nfu_mirna_prediction mature_miRNA 47267 47288 . - . ID=nfu_pred_68;biotype=3'-mature-miRNA;gene=mir-230;geneid=nfu_pred_68_mir-230;coords=scaffold00613:47267-47288

scaffold00614 nfu_mirna_prediction pre_miRNA 68923 69001 . + . ID=nfu_pred_69;biotype=pre-miRNA;gene=mir-1;geneid=nfu_pred_69_mir-1;coords=scaffold00614:68923-69001

scaffold00614 nfu_mirna_prediction mature_miRNA 68970 68991 . + . ID=nfu_pred_69;biotype=3'-mature-miRNA;gene=mir-1;geneid=nfu_pred_69_mir-1;coords=scaffold00614:68970-68991

scaffold00614 nfu_mirna_prediction pre_miRNA 69661 69749 . + . ID=nfu_pred_70;biotype=pre-miRNA;gene=mir-133;geneid=nfu_pred_70_mir-133;coords=scaffold00614:69661-69749

scaffold00614 nfu_mirna_prediction mature_miRNA 69713 69733 . + . ID=nfu_pred_70;biotype=3'-mature-miRNA;gene=mir-133;geneid=nfu_pred_70_mir-133;coords=scaffold00614:69713-69733

scaffold00655 nfu_mirna_prediction pre_miRNA 71987 72065 . - . ID=nfu_pred_72;biotype=pre-miRNA;gene=;geneid=nfu_pred_72_;coords=scaffold00655:71987-72065

scaffold00655 nfu_mirna_prediction mature_miRNA 72040 72061 . - . ID=nfu_pred_72;biotype=5'-mature-miRNA;gene=;geneid=nfu_pred_72_;coords=scaffold00655:72040-72061

scaffold00655 nfu_mirna_prediction mature_miRNA 72000 72019 . - . ID=nfu_pred_72;biotype=3'-mature-miRNA;gene=;geneid=nfu_pred_72_;coords=scaffold00655:72000-72019

scaffold00676 nfu_mirna_prediction pre_miRNA 43170 43226 . - . ID=nfu_pred_73;biotype=pre-miRNA;gene=mir-7641;geneid=nfu_pred_73_mir-7641;coords=scaffold00676:43170-43226

scaffold00682 nfu_mirna_prediction pre_miRNA 13252 13339 . - . ID=nfu_pred_74;biotype=pre-miRNA;gene=mir-33;geneid=nfu_pred_74_mir-33;coords=scaffold00682:13252-13339

scaffold00715 nfu_mirna_prediction pre_miRNA 30698 30782 . - . ID=nfu_pred_76;biotype=pre-miRNA;gene=mir-42;geneid=nfu_pred_76_mir-42;coords=scaffold00715:30698-30782

scaffold00715 nfu_mirna_prediction mature_miRNA 30756 30778 . - . ID=nfu_pred_76;biotype=5'-mature-miRNA;gene=mir-42;geneid=nfu_pred_76_mir-42;coords=scaffold00715:30756-30778

scaffold00715 nfu_mirna_prediction mature_miRNA 30716 30737 . - . ID=nfu_pred_76;biotype=3'-mature-miRNA;gene=mir-42;geneid=nfu_pred_76_mir-42;coords=scaffold00715:30716-30737

scaffold00715 nfu_mirna_prediction pre_miRNA 37113 37176 . + . ID=nfu_pred_77;biotype=pre-miRNA;gene=;geneid=nfu_pred_77_;coords=scaffold00715:37113-37176

scaffold00721 nfu_mirna_prediction pre_miRNA 50978 51050 . + . ID=nfu_pred_78;biotype=pre-miRNA;gene=mir-144;geneid=nfu_pred_78_mir-144;coords=scaffold00721:50978-51050

scaffold00721 nfu_mirna_prediction pre_miRNA 51374 51508 . + . ID=nfu_pred_79;biotype=pre-miRNA;gene=mir-33;geneid=nfu_pred_79_mir-33;coords=scaffold00721:51374-51508

scaffold00730 nfu_mirna_prediction pre_miRNA 53186 53264 . - . ID=nfu_pred_80;biotype=pre-miRNA;gene=mir-30;geneid=nfu_pred_80_mir-30;coords=scaffold00730:53186-53264

scaffold00730 nfu_mirna_prediction mature_miRNA 53233 53254 . - . ID=nfu_pred_80;biotype=5'-mature-miRNA;gene=mir-30;geneid=nfu_pred_80_mir-30;coords=scaffold00730:53233-53254

scaffold00730 nfu_mirna_prediction mature_miRNA 53192 53212 . - . ID=nfu_pred_80;biotype=3'-mature-miRNA;gene=mir-30;geneid=nfu_pred_80_mir-30;coords=scaffold00730:53192-53212

scaffold00730 nfu_mirna_prediction pre_miRNA 53393 53473 . - . ID=nfu_pred_81;biotype=pre-miRNA;gene=mir-30;geneid=nfu_pred_81_mir-30;coords=scaffold00730:53393-53473

scaffold00730 nfu_mirna_prediction mature_miRNA 53440 53463 . - . ID=nfu_pred_81;biotype=5'-mature-miRNA;gene=mir-30;geneid=nfu_pred_81_mir-30;coords=scaffold00730:53440-53463

scaffold00730 nfu_mirna_prediction mature_miRNA 53404 53425 . - . ID=nfu_pred_81;biotype=3'-mature-miRNA;gene=mir-30;geneid=nfu_pred_81_mir-30;coords=scaffold00730:53404-53425

scaffold00732 nfu_mirna_prediction pre_miRNA 27543 27608 . - . ID=nfu_pred_82;biotype=pre-miRNA;gene=let-7;geneid=nfu_pred_82_let-7;coords=scaffold00732:27543-27608

scaffold00732 nfu_mirna_prediction mature_miRNA 27580 27602 . - . ID=nfu_pred_82;biotype=5'-mature-miRNA;gene=let-7;geneid=nfu_pred_82_let-7;coords=scaffold00732:27580-27602

scaffold00795 nfu_mirna_prediction pre_miRNA 8968 9069 . - . ID=nfu_pred_83;biotype=pre-miRNA;gene=mir-358;geneid=nfu_pred_83_mir-358;coords=scaffold00795:8968-9069

scaffold00800 nfu_mirna_prediction pre_miRNA 14844 14922 . + . ID=nfu_pred_84;biotype=pre-miRNA;gene=mir-885;geneid=nfu_pred_84_mir-885;coords=scaffold00800:14844-14922

scaffold00800 nfu_mirna_prediction mature_miRNA 14854 14876 . + . ID=nfu_pred_84;biotype=5'-mature-miRNA;gene=mir-885;geneid=nfu_pred_84_mir-885;coords=scaffold00800:14854-14876

scaffold00800 nfu_mirna_prediction mature_miRNA 14889 14909 . + . ID=nfu_pred_84;biotype=3'-mature-miRNA;gene=mir-885;geneid=nfu_pred_84_mir-885;coords=scaffold00800:14889-14909

scaffold00836 nfu_mirna_prediction pre_miRNA 39804 39918 . + . ID=nfu_pred_85;biotype=pre-miRNA;gene=mir-455;geneid=nfu_pred_85_mir-455;coords=scaffold00836:39804-39918

scaffold00836 nfu_mirna_prediction mature_miRNA 39866 39887 . + . ID=nfu_pred_85;biotype=3'-mature-miRNA;gene=mir-455;geneid=nfu_pred_85_mir-455;coords=scaffold00836:39866-39887

scaffold00860 nfu_mirna_prediction pre_miRNA 5258 5339 . + . ID=nfu_pred_86;biotype=pre-miRNA;gene=mir-287;geneid=nfu_pred_86_mir-287;coords=scaffold00860:5258-5339

scaffold00888 nfu_mirna_prediction pre_miRNA 27515 27612 . - . ID=nfu_pred_90;biotype=pre-miRNA;gene=mir-6720;geneid=nfu_pred_90_mir-6720;coords=scaffold00888:27515-27612

scaffold00892 nfu_mirna_prediction pre_miRNA 39340 39429 . + . ID=nfu_pred_91;biotype=pre-miRNA;gene=mir-196;geneid=nfu_pred_91_mir-196;coords=scaffold00892:39340-39429

scaffold00892 nfu_mirna_prediction mature_miRNA 39358 39381 . + . ID=nfu_pred_91;biotype=5'-mature-miRNA;gene=mir-196;geneid=nfu_pred_91_mir-196;coords=scaffold00892:39358-39381

scaffold00892 nfu_mirna_prediction mature_miRNA 39394 39415 . + . ID=nfu_pred_91;biotype=3'-mature-miRNA;gene=mir-196;geneid=nfu_pred_91_mir-196;coords=scaffold00892:39394-39415

scaffold00898 nfu_mirna_prediction pre_miRNA 9631 9719 . - . ID=nfu_pred_92;biotype=pre-miRNA;gene=mir-458;geneid=nfu_pred_92_mir-458;coords=scaffold00898:9631-9719

scaffold00950 nfu_mirna_prediction pre_miRNA 28788 28868 . + . ID=nfu_pred_93;biotype=pre-miRNA;gene=mir-4683;geneid=nfu_pred_93_mir-4683;coords=scaffold00950:28788-28868

scaffold00954 nfu_mirna_prediction pre_miRNA 12429 12507 . + . ID=nfu_pred_94;biotype=pre-miRNA;gene=;geneid=nfu_pred_94_;coords=scaffold00954:12429-12507

scaffold00954 nfu_mirna_prediction mature_miRNA 12476 12497 . + . ID=nfu_pred_94;biotype=3'-mature-miRNA;gene=;geneid=nfu_pred_94_;coords=scaffold00954:12476-12497

scaffold00980 nfu_mirna_prediction pre_miRNA 22015 22095 . + . ID=nfu_pred_95;biotype=pre-miRNA;gene=;geneid=nfu_pred_95_;coords=scaffold00980:22015-22095

scaffold00980 nfu_mirna_prediction mature_miRNA 22026 22047 . + . ID=nfu_pred_95;biotype=5'-mature-miRNA;gene=;geneid=nfu_pred_95_;coords=scaffold00980:22026-22047

scaffold00980 nfu_mirna_prediction mature_miRNA 22063 22084 . + . ID=nfu_pred_95;biotype=3'-mature-miRNA;gene=;geneid=nfu_pred_95_;coords=scaffold00980:22063-22084

scaffold00989 nfu_mirna_prediction pre_miRNA 2287 2345 . + . ID=nfu_pred_96;biotype=pre-miRNA;gene=mir-7641;geneid=nfu_pred_96_mir-7641;coords=scaffold00989:2287-2345

scaffold01015 nfu_mirna_prediction pre_miRNA 48174 48230 . + . ID=nfu_pred_97;biotype=pre-miRNA;gene=mir-7641;geneid=nfu_pred_97_mir-7641;coords=scaffold01015:48174-48230

scaffold01015 nfu_mirna_prediction mature_miRNA 48179 48199 . + . ID=nfu_pred_97;biotype=5'-mature-miRNA;gene=mir-7641;geneid=nfu_pred_97_mir-7641;coords=scaffold01015:48179-48199

scaffold01015 nfu_mirna_prediction mature_miRNA 48207 48227 . + . ID=nfu_pred_97;biotype=3'-mature-miRNA;gene=mir-7641;geneid=nfu_pred_97_mir-7641;coords=scaffold01015:48207-48227

scaffold01082 nfu_mirna_prediction pre_miRNA 7332 7403 . + . ID=nfu_pred_98;biotype=pre-miRNA;gene=mir-144;geneid=nfu_pred_98_mir-144;coords=scaffold01082:7332-7403

scaffold01094 nfu_mirna_prediction pre_miRNA 22958 23044 . - . ID=nfu_pred_99;biotype=pre-miRNA;gene=mir-122a/mir-3591;geneid=nfu_pred_99_mir-122a/mir-3591;coords=scaffold01094:22958-23044

scaffold01141 nfu_mirna_prediction pre_miRNA 11975 12053 . - . ID=nfu_pred_100;biotype=pre-miRNA;gene=mir-101;geneid=nfu_pred_100_mir-101;coords=scaffold01141:11975-12053

scaffold01141 nfu_mirna_prediction mature_miRNA 11985 12006 . - . ID=nfu_pred_100;biotype=3'-mature-miRNA;gene=mir-101;geneid=nfu_pred_100_mir-101;coords=scaffold01141:11985-12006

scaffold01172 nfu_mirna_prediction pre_miRNA 10697 10775 . + . ID=nfu_pred_101;biotype=pre-miRNA;gene=;geneid=nfu_pred_101_;coords=scaffold01172:10697-10775

scaffold01172 nfu_mirna_prediction mature_miRNA 10706 10728 . + . ID=nfu_pred_101;biotype=5'-mature-miRNA;gene=;geneid=nfu_pred_101_;coords=scaffold01172:10706-10728

scaffold01172 nfu_mirna_prediction mature_miRNA 10744 10766 . + . ID=nfu_pred_101;biotype=3'-mature-miRNA;gene=;geneid=nfu_pred_101_;coords=scaffold01172:10744-10766

scaffold01177 nfu_mirna_prediction pre_miRNA 43424 43486 . + . ID=nfu_pred_102;biotype=pre-miRNA;gene=;geneid=nfu_pred_102_;coords=scaffold01177:43424-43486

scaffold01194 nfu_mirna_prediction pre_miRNA 38646 38722 . - . ID=nfu_pred_104;biotype=pre-miRNA;gene=mir-96a;geneid=nfu_pred_104_mir-96a;coords=scaffold01194:38646-38722

scaffold01210 nfu_mirna_prediction pre_miRNA 29934 30003 . - . ID=nfu_pred_105;biotype=pre-miRNA;gene=mir-3187;geneid=nfu_pred_105_mir-3187;coords=scaffold01210:29934-30003

scaffold01223 nfu_mirna_prediction pre_miRNA 9808 9894 . - . ID=nfu_pred_106;biotype=pre-miRNA;gene=mir-34;geneid=nfu_pred_106_mir-34;coords=scaffold01223:9808-9894

scaffold01223 nfu_mirna_prediction mature_miRNA 9824 9845 . - . ID=nfu_pred_106;biotype=3'-mature-miRNA;gene=mir-34;geneid=nfu_pred_106_mir-34;coords=scaffold01223:9824-9845

scaffold01224 nfu_mirna_prediction pre_miRNA 6689 6767 . + . ID=nfu_pred_107;biotype=pre-miRNA;gene=mir-939;geneid=nfu_pred_107_mir-939;coords=scaffold01224:6689-6767

scaffold01224 nfu_mirna_prediction mature_miRNA 6697 6721 . + . ID=nfu_pred_107;biotype=5'-mature-miRNA;gene=mir-939;geneid=nfu_pred_107_mir-939;coords=scaffold01224:6697-6721

scaffold01224 nfu_mirna_prediction mature_miRNA 6736 6757 . + . ID=nfu_pred_107;biotype=3'-mature-miRNA;gene=mir-939;geneid=nfu_pred_107_mir-939;coords=scaffold01224:6736-6757

scaffold01281 nfu_mirna_prediction pre_miRNA 6327 6426 . - . ID=nfu_pred_108;biotype=pre-miRNA;gene=mir-506;geneid=nfu_pred_108_mir-506;coords=scaffold01281:6327-6426

scaffold01281 nfu_mirna_prediction mature_miRNA 6392 6402 . - . ID=nfu_pred_108;biotype=5'-mature-miRNA;gene=mir-506;geneid=nfu_pred_108_mir-506;coords=scaffold01281:6392-6402

scaffold01281 nfu_mirna_prediction mature_miRNA 6447 6467 . - . ID=nfu_pred_108;biotype=3'-mature-miRNA;gene=mir-506;geneid=nfu_pred_108_mir-506;coords=scaffold01281:6447-6467

scaffold01293 nfu_mirna_prediction pre_miRNA 36109 36165 . + . ID=nfu_pred_109;biotype=pre-miRNA;gene=mir-7641;geneid=nfu_pred_109_mir-7641;coords=scaffold01293:36109-36165

scaffold01306 nfu_mirna_prediction pre_miRNA 22325 22407 . + . ID=nfu_pred_112;biotype=pre-miRNA;gene=mir-609;geneid=nfu_pred_112_mir-609;coords=scaffold01306:22325-22407

scaffold01306 nfu_mirna_prediction mature_miRNA 22335 22356 . + . ID=nfu_pred_112;biotype=5'-mature-miRNA;gene=mir-609;geneid=nfu_pred_112_mir-609;coords=scaffold01306:22335-22356

scaffold01306 nfu_mirna_prediction mature_miRNA 22378 22399 . + . ID=nfu_pred_112;biotype=3'-mature-miRNA;gene=mir-609;geneid=nfu_pred_112_mir-609;coords=scaffold01306:22378-22399

scaffold01309 nfu_mirna_prediction pre_miRNA 4481 4589 . - . ID=nfu_pred_113;biotype=pre-miRNA;gene=mir-33;geneid=nfu_pred_113_mir-33;coords=scaffold01309:4481-4589

scaffold01336 nfu_mirna_prediction pre_miRNA 36047 36144 . - . ID=nfu_pred_114;biotype=pre-miRNA;gene=mir-203;geneid=nfu_pred_114_mir-203;coords=scaffold01336:36047-36144

scaffold01363 nfu_mirna_prediction pre_miRNA 18888 18983 . + . ID=nfu_pred_115;biotype=pre-miRNA;gene=mir-287;geneid=nfu_pred_115_mir-287;coords=scaffold01363:18888-18983

scaffold01455 nfu_mirna_prediction pre_miRNA 9989 10076 . + . ID=nfu_pred_116;biotype=pre-miRNA;gene=mir-187;geneid=nfu_pred_116_mir-187;coords=scaffold01455:9989-10076

scaffold01455 nfu_mirna_prediction mature_miRNA 9996 10012 . + . ID=nfu_pred_116;biotype=5'-mature-miRNA;gene=mir-187;geneid=nfu_pred_116_mir-187;coords=scaffold01455:9996-10012

scaffold01455 nfu_mirna_prediction mature_miRNA 10034 10055 . + . ID=nfu_pred_116;biotype=3'-mature-miRNA;gene=mir-187;geneid=nfu_pred_116_mir-187;coords=scaffold01455:10034-10055

scaffold01489 nfu_mirna_prediction pre_miRNA 8029 8111 . + . ID=nfu_pred_117;biotype=pre-miRNA;gene=;geneid=nfu_pred_117_;coords=scaffold01489:8029-8111

scaffold01489 nfu_mirna_prediction mature_miRNA 8039 8062 . + . ID=nfu_pred_117;biotype=5'-mature-miRNA;gene=;geneid=nfu_pred_117_;coords=scaffold01489:8039-8062

scaffold01489 nfu_mirna_prediction mature_miRNA 8078 8099 . + . ID=nfu_pred_117;biotype=3'-mature-miRNA;gene=;geneid=nfu_pred_117_;coords=scaffold01489:8078-8099

scaffold01593 nfu_mirna_prediction pre_miRNA 29585 29669 . + . ID=nfu_pred_118;biotype=pre-miRNA;gene=;geneid=nfu_pred_118_;coords=scaffold01593:29585-29669

scaffold01596 nfu_mirna_prediction pre_miRNA 480 561 . - . ID=nfu_pred_119;biotype=pre-miRNA;gene=mir-122;geneid=nfu_pred_119_mir-122;coords=scaffold01596:480-561

scaffold01596 nfu_mirna_prediction mature_miRNA 533 555 . - . ID=nfu_pred_119;biotype=5'-mature-miRNA;gene=mir-122;geneid=nfu_pred_119_mir-122;coords=scaffold01596:533-555

scaffold01596 nfu_mirna_prediction mature_miRNA 493 515 . - . ID=nfu_pred_119;biotype=3'-mature-miRNA;gene=mir-122;geneid=nfu_pred_119_mir-122;coords=scaffold01596:493-515

scaffold01715 nfu_mirna_prediction pre_miRNA 9533 9629 . + . ID=nfu_pred_121;biotype=pre-miRNA;gene=mir-223;geneid=nfu_pred_121_mir-223;coords=scaffold01715:9533-9629

scaffold01829 nfu_mirna_prediction pre_miRNA 275 404 . + . ID=nfu_pred_122;biotype=pre-miRNA;gene=mir-458;geneid=nfu_pred_122_mir-458;coords=scaffold01829:275-404

scaffold01878 nfu_mirna_prediction pre_miRNA 6420 6517 . + . ID=nfu_pred_123;biotype=pre-miRNA;gene=mir-672;geneid=nfu_pred_123_mir-672;coords=scaffold01878:6420-6517

scaffold02021 nfu_mirna_prediction pre_miRNA 17346 17417 . - . ID=nfu_pred_125;biotype=pre-miRNA;gene=;geneid=nfu_pred_125_;coords=scaffold02021:17346-17417

scaffold02021 nfu_mirna_prediction mature_miRNA 17352 17373 . - . ID=nfu_pred_125;biotype=3'-mature-miRNA;gene=;geneid=nfu_pred_125_;coords=scaffold02021:17352-17373

scaffold02021 nfu_mirna_prediction mature_miRNA 17392 17412 . - . ID=nfu_pred_125;biotype=5'-mature-miRNA;gene=;geneid=nfu_pred_125_;coords=scaffold02021:17392-17412

scaffold02091 nfu_mirna_prediction pre_miRNA 16939 17017 . + . ID=nfu_pred_126;biotype=pre-miRNA;gene=mir-676;geneid=nfu_pred_126_mir-676;coords=scaffold02091:16939-17017

scaffold02105 nfu_mirna_prediction pre_miRNA 10549 10633 . + . ID=nfu_pred_127;biotype=pre-miRNA;gene=mir-148;geneid=nfu_pred_127_mir-148;coords=scaffold02105:10549-10633

scaffold02117 nfu_mirna_prediction pre_miRNA 13972 14073 . + . ID=nfu_pred_128;biotype=pre-miRNA;gene=mir-811;geneid=nfu_pred_128_mir-811;coords=scaffold02117:13972-14073

scaffold02117 nfu_mirna_prediction mature_miRNA 13995 14016 . + . ID=nfu_pred_128;biotype=5'-mature-miRNA;gene=mir-811;geneid=nfu_pred_128_mir-811;coords=scaffold02117:13995-14016

scaffold02117 nfu_mirna_prediction mature_miRNA 14030 14051 . + . ID=nfu_pred_128;biotype=3'-mature-miRNA;gene=mir-811;geneid=nfu_pred_128_mir-811;coords=scaffold02117:14030-14051

scaffold02167 nfu_mirna_prediction pre_miRNA 312 401 . - . ID=nfu_pred_129;biotype=pre-miRNA;gene=mir-133;geneid=nfu_pred_129_mir-133;coords=scaffold02167:312-401

scaffold02167 nfu_mirna_prediction mature_miRNA 326 348 . - . ID=nfu_pred_129;biotype=3'-mature-miRNA;gene=mir-133;geneid=nfu_pred_129_mir-133;coords=scaffold02167:326-348

scaffold02167 nfu_mirna_prediction pre_miRNA 9661 9740 . - . ID=nfu_pred_130;biotype=pre-miRNA;gene=mir-1;geneid=nfu_pred_130_mir-1;coords=scaffold02167:9661-9740

scaffold02167 nfu_mirna_prediction mature_miRNA 9671 9692 . - . ID=nfu_pred_130;biotype=3'-mature-miRNA;gene=mir-1;geneid=nfu_pred_130_mir-1;coords=scaffold02167:9671-9692

scaffold02575 nfu_mirna_prediction pre_miRNA 406 489 . - . ID=nfu_pred_133;biotype=pre-miRNA;gene=mir-221;geneid=nfu_pred_133_mir-221;coords=scaffold02575:406-489

scaffold02575 nfu_mirna_prediction mature_miRNA 416 438 . - . ID=nfu_pred_133;biotype=3'-mature-miRNA;gene=mir-221;geneid=nfu_pred_133_mir-221;coords=scaffold02575:416-438

scaffold02575 nfu_mirna_prediction pre_miRNA 661 743 . - . ID=nfu_pred_134;biotype=pre-miRNA;gene=mir-221;geneid=nfu_pred_134_mir-221;coords=scaffold02575:661-743

scaffold02575 nfu_mirna_prediction mature_miRNA 671 694 . - . ID=nfu_pred_134;biotype=3'-mature-miRNA;gene=mir-221;geneid=nfu_pred_134_mir-221;coords=scaffold02575:671-694

scaffold02844 nfu_mirna_prediction pre_miRNA 5654 5758 . - . ID=nfu_pred_139;biotype=pre-miRNA;gene=mir-576;geneid=nfu_pred_139_mir-576;coords=scaffold02844:5654-5758

scaffold02844 nfu_mirna_prediction mature_miRNA 5664 5685 . - . ID=nfu_pred_139;biotype=3'-mature-miRNA;gene=mir-576;geneid=nfu_pred_139_mir-576;coords=scaffold02844:5664-5685

scaffold03269 nfu_mirna_prediction pre_miRNA 4164 4268 . - . ID=nfu_pred_142;biotype=pre-miRNA;gene=mir-576;geneid=nfu_pred_142_mir-576;coords=scaffold03269:4164-4268

scaffold03269 nfu_mirna_prediction mature_miRNA 4174 4195 . - . ID=nfu_pred_142;biotype=3'-mature-miRNA;gene=mir-576;geneid=nfu_pred_142_mir-576;coords=scaffold03269:4174-4195

scaffold03726 nfu_mirna_prediction pre_miRNA 32 130 . - . ID=nfu_pred_144;biotype=pre-miRNA;gene=mir-430;geneid=nfu_pred_144_mir-430;coords=scaffold03726:32-130

scaffold03726 nfu_mirna_prediction mature_miRNA 51 72 . - . ID=nfu_pred_144;biotype=3'-mature-miRNA;gene=mir-430;geneid=nfu_pred_144_mir-430;coords=scaffold03726:51-72

scaffold03726 nfu_mirna_prediction pre_miRNA 555 654 . - . ID=nfu_pred_145;biotype=pre-miRNA;gene=mir-430;geneid=nfu_pred_145_mir-430;coords=scaffold03726:555-654

scaffold03726 nfu_mirna_prediction mature_miRNA 574 595 . - . ID=nfu_pred_145;biotype=3'-mature-miRNA;gene=mir-430;geneid=nfu_pred_145_mir-430;coords=scaffold03726:574-595

scaffold03727 nfu_mirna_prediction pre_miRNA 2740 2851 . + . ID=nfu_pred_146;biotype=pre-miRNA;gene=;geneid=nfu_pred_146_;coords=scaffold03727:2740-2851

scaffold03727 nfu_mirna_prediction mature_miRNA 2812 2833 . + . ID=nfu_pred_146;biotype=5'-mature-miRNA;gene=;geneid=nfu_pred_146_;coords=scaffold03727:2812-2833

scaffold03727 nfu_mirna_prediction mature_miRNA 2749 2770 . + . ID=nfu_pred_146;biotype=3'-mature-miRNA;gene=;geneid=nfu_pred_146_;coords=scaffold03727:2749-2770

scaffold03789 nfu_mirna_prediction pre_miRNA 1024 1116 . + . ID=nfu_pred_147;biotype=pre-miRNA;gene=mir-458;geneid=nfu_pred_147_mir-458;coords=scaffold03789:1024-1116

scaffold03789 nfu_mirna_prediction pre_miRNA 2671 2763 . + . ID=nfu_pred_148;biotype=pre-miRNA;gene=mir-458;geneid=nfu_pred_148_mir-458;coords=scaffold03789:2671-2763

scaffold04459 nfu_mirna_prediction pre_miRNA 1024 1119 . + . ID=nfu_pred_149;biotype=pre-miRNA;gene=mir-598;geneid=nfu_pred_149_mir-598;coords=scaffold04459:1024-1119

scaffold05327 nfu_mirna_prediction pre_miRNA 1429 1542 . + . ID=nfu_pred_150;biotype=pre-miRNA;gene=mir-789;geneid=nfu_pred_150_mir-789;coords=scaffold05327:1429-1542

scaffold05327 nfu_mirna_prediction mature_miRNA 1443 1466 . + . ID=nfu_pred_150;biotype=5'-mature-miRNA;gene=mir-789;geneid=nfu_pred_150_mir-789;coords=scaffold05327:1443-1466

scaffold05848 nfu_mirna_prediction pre_miRNA 464 526 . + . ID=nfu_pred_151;biotype=pre-miRNA;gene=;geneid=nfu_pred_151_;coords=scaffold05848:464-526

sgr01 nfu_mirna_prediction pre_miRNA 435269 435361 . + . ID=nfu_pred_152;biotype=pre-miRNA;gene=mir-338;geneid=nfu_pred_152_mir-338;coords=sgr01:435269-435361

sgr01 nfu_mirna_prediction mature_miRNA 435288 435311 . + . ID=nfu_pred_152;biotype=5'-mature-miRNA;gene=mir-338;geneid=nfu_pred_152_mir-338;coords=sgr01:435288-435311

sgr01 nfu_mirna_prediction mature_miRNA 435323 435344 . + . ID=nfu_pred_152;biotype=3'-mature-miRNA;gene=mir-338;geneid=nfu_pred_152_mir-338;coords=sgr01:435323-435344

sgr01 nfu_mirna_prediction pre_miRNA 958619 958721 . + . ID=nfu_pred_153;biotype=pre-miRNA;gene=mir-22;geneid=nfu_pred_153_mir-22;coords=sgr01:958619-958721

sgr01 nfu_mirna_prediction mature_miRNA 958641 958661 . + . ID=nfu_pred_153;biotype=5'-mature-miRNA;gene=mir-22;geneid=nfu_pred_153_mir-22;coords=sgr01:958641-958661

sgr01 nfu_mirna_prediction mature_miRNA 958677 958698 . + . ID=nfu_pred_153;biotype=3'-mature-miRNA;gene=mir-22;geneid=nfu_pred_153_mir-22;coords=sgr01:958677-958698

sgr01 nfu_mirna_prediction pre_miRNA 1702739 1702835 . - . ID=nfu_pred_154;biotype=pre-miRNA;gene=mir-722;geneid=nfu_pred_154_mir-722;coords=sgr01:1702739-1702835

sgr01 nfu_mirna_prediction mature_miRNA 1702775 1702796 . - . ID=nfu_pred_154;biotype=3'-mature-miRNA;gene=mir-722;geneid=nfu_pred_154_mir-722;coords=sgr01:1702775-1702796

sgr01 nfu_mirna_prediction mature_miRNA 1702752 1702773 . - . ID=nfu_pred_154;biotype=5'-mature-miRNA;gene=mir-722;geneid=nfu_pred_154_mir-722;coords=sgr01:1702752-1702773

sgr01 nfu_mirna_prediction pre_miRNA 1953846 1953944 . - . ID=nfu_pred_155;biotype=pre-miRNA;gene=mir-199;geneid=nfu_pred_155_mir-199;coords=sgr01:1953846-1953944

sgr01 nfu_mirna_prediction mature_miRNA 1953902 1953924 . - . ID=nfu_pred_155;biotype=5'-mature-miRNA;gene=mir-199;geneid=nfu_pred_155_mir-199;coords=sgr01:1953902-1953924

sgr01 nfu_mirna_prediction mature_miRNA 1953864 1953885 . - . ID=nfu_pred_155;biotype=3'-mature-miRNA;gene=mir-199;geneid=nfu_pred_155_mir-199;coords=sgr01:1953864-1953885

sgr01 nfu_mirna_prediction pre_miRNA 3822139 3822237 . + . ID=nfu_pred_156;biotype=pre-miRNA;gene=mir-552;geneid=nfu_pred_156_mir-552;coords=sgr01:3822139-3822237

sgr01 nfu_mirna_prediction mature_miRNA 3822161 3822181 . + . ID=nfu_pred_156;biotype=5'-mature-miRNA;gene=mir-552;geneid=nfu_pred_156_mir-552;coords=sgr01:3822161-3822181

sgr01 nfu_mirna_prediction mature_miRNA 3822200 3822221 . + . ID=nfu_pred_156;biotype=3'-mature-miRNA;gene=mir-552;geneid=nfu_pred_156_mir-552;coords=sgr01:3822200-3822221

sgr01 nfu_mirna_prediction pre_miRNA 7102580 7102660 . - . ID=nfu_pred_158;biotype=pre-miRNA;gene=mir-4683;geneid=nfu_pred_158_mir-4683;coords=sgr01:7102580-7102660

sgr01 nfu_mirna_prediction pre_miRNA 11052262 11052344 . + . ID=nfu_pred_159;biotype=pre-miRNA;gene=;geneid=nfu_pred_159_;coords=sgr01:11052262-11052344

sgr01 nfu_mirna_prediction mature_miRNA 11052273 11052295 . + . ID=nfu_pred_159;biotype=5'-mature-miRNA;gene=;geneid=nfu_pred_159_;coords=sgr01:11052273-11052295

sgr01 nfu_mirna_prediction pre_miRNA 18251103 18251232 . + . ID=nfu_pred_160;biotype=pre-miRNA;gene=mir-458;geneid=nfu_pred_160_mir-458;coords=sgr01:18251103-18251232

sgr01 nfu_mirna_prediction pre_miRNA 27733614 27733698 . + . ID=nfu_pred_161;biotype=pre-miRNA;gene=mir-BART7;geneid=nfu_pred_161_mir-BART7;coords=sgr01:27733614-27733698

sgr01 nfu_mirna_prediction mature_miRNA 27733624 27733645 . + . ID=nfu_pred_161;biotype=5'-mature-miRNA;gene=mir-BART7;geneid=nfu_pred_161_mir-BART7;coords=sgr01:27733624-27733645

sgr01 nfu_mirna_prediction mature_miRNA 27733663 27733685 . + . ID=nfu_pred_161;biotype=3'-mature-miRNA;gene=mir-BART7;geneid=nfu_pred_161_mir-BART7;coords=sgr01:27733663-27733685

sgr01 nfu_mirna_prediction pre_miRNA 30894458 30894557 . - . ID=nfu_pred_823;biotype=pre-miRNA;gene=mir-7;geneid=nfu_pred_823_mir-7;coords=sgr01:30894458-30894557

sgr01 nfu_mirna_prediction pre_miRNA 33308337 33308406 . + . ID=nfu_pred_162;biotype=pre-miRNA;gene=;geneid=nfu_pred_162_;coords=sgr01:33308337-33308406

sgr01 nfu_mirna_prediction mature_miRNA 33308347 33308369 . + . ID=nfu_pred_162;biotype=5'-mature-miRNA;gene=;geneid=nfu_pred_162_;coords=sgr01:33308347-33308369

sgr01 nfu_mirna_prediction mature_miRNA 33308383 33308405 . + . ID=nfu_pred_162;biotype=3'-mature-miRNA;gene=;geneid=nfu_pred_162_;coords=sgr01:33308383-33308405

sgr01 nfu_mirna_prediction pre_miRNA 33674963 33675057 . - . ID=nfu_pred_163;biotype=pre-miRNA;gene=mir-1444;geneid=nfu_pred_163_mir-1444;coords=sgr01:33674963-33675057

sgr01 nfu_mirna_prediction mature_miRNA 33675022 33675041 . - . ID=nfu_pred_163;biotype=5'-mature-miRNA;gene=mir-1444;geneid=nfu_pred_163_mir-1444;coords=sgr01:33675022-33675041

sgr01 nfu_mirna_prediction mature_miRNA 33674977 33674998 . - . ID=nfu_pred_163;biotype=3'-mature-miRNA;gene=mir-1444;geneid=nfu_pred_163_mir-1444;coords=sgr01:33674977-33674998

sgr01 nfu_mirna_prediction pre_miRNA 35197009 35197093 . - . ID=nfu_pred_164;biotype=pre-miRNA;gene=mir-625;geneid=nfu_pred_164_mir-625;coords=sgr01:35197009-35197093

sgr01 nfu_mirna_prediction mature_miRNA 35197022 35197043 . - . ID=nfu_pred_164;biotype=3'-mature-miRNA;gene=mir-625;geneid=nfu_pred_164_mir-625;coords=sgr01:35197022-35197043

sgr01 nfu_mirna_prediction pre_miRNA 43903864 43903980 . - . ID=nfu_pred_165;biotype=pre-miRNA;gene=mir-83;geneid=nfu_pred_165_mir-83;coords=sgr01:43903864-43903980

sgr01 nfu_mirna_prediction mature_miRNA 43903874 43903895 . - . ID=nfu_pred_165;biotype=3'-mature-miRNA;gene=mir-83;geneid=nfu_pred_165_mir-83;coords=sgr01:43903874-43903895

sgr01 nfu_mirna_prediction pre_miRNA 46246905 46246983 . - . ID=nfu_pred_166;biotype=pre-miRNA;gene=mir-92;geneid=nfu_pred_166_mir-92;coords=sgr01:46246905-46246983

sgr01 nfu_mirna_prediction mature_miRNA 46246915 46246935 . - . ID=nfu_pred_166;biotype=3'-mature-miRNA;gene=mir-92;geneid=nfu_pred_166_mir-92;coords=sgr01:46246915-46246935

sgr01 nfu_mirna_prediction pre_miRNA 47093740 47093810 . - . ID=nfu_pred_167;biotype=pre-miRNA;gene=mir-1905;geneid=nfu_pred_167_mir-1905;coords=sgr01:47093740-47093810

sgr01 nfu_mirna_prediction pre_miRNA 48042723 48042810 . - . ID=nfu_pred_168;biotype=pre-miRNA;gene=mir-7;geneid=nfu_pred_168_mir-7;coords=sgr01:48042723-48042810

sgr01 nfu_mirna_prediction mature_miRNA 48042774 48042796 . - . ID=nfu_pred_168;biotype=5'-mature-miRNA;gene=mir-7;geneid=nfu_pred_168_mir-7;coords=sgr01:48042774-48042796

sgr01 nfu_mirna_prediction pre_miRNA 50910838 50910900 . + . ID=nfu_pred_169;biotype=pre-miRNA;gene=;geneid=nfu_pred_169_;coords=sgr01:50910838-50910900

sgr01 nfu_mirna_prediction pre_miRNA 54083521 54083599 . + . ID=nfu_pred_170;biotype=pre-miRNA;gene=mir-9;geneid=nfu_pred_170_mir-9;coords=sgr01:54083521-54083599

sgr01 nfu_mirna_prediction mature_miRNA 54083531 54083553 . + . ID=nfu_pred_170;biotype=5'-mature-miRNA;gene=mir-9;geneid=nfu_pred_170_mir-9;coords=sgr01:54083531-54083553

sgr01 nfu_mirna_prediction mature_miRNA 54083570 54083591 . + . ID=nfu_pred_170;biotype=3'-mature-miRNA;gene=mir-9;geneid=nfu_pred_170_mir-9;coords=sgr01:54083570-54083591

sgr01 nfu_mirna_prediction pre_miRNA 59955147 59955229 . + . ID=nfu_pred_171;biotype=pre-miRNA;gene=mir-944;geneid=nfu_pred_171_mir-944;coords=sgr01:59955147-59955229

sgr01 nfu_mirna_prediction mature_miRNA 59955158 59955180 . + . ID=nfu_pred_171;biotype=5'-mature-miRNA;gene=mir-944;geneid=nfu_pred_171_mir-944;coords=sgr01:59955158-59955180

sgr01 nfu_mirna_prediction mature_miRNA 59955196 59955217 . + . ID=nfu_pred_171;biotype=3'-mature-miRNA;gene=mir-944;geneid=nfu_pred_171_mir-944;coords=sgr01:59955196-59955217

sgr01 nfu_mirna_prediction pre_miRNA 60174602 60174690 . - . ID=nfu_pred_172;biotype=pre-miRNA;gene=mir-944;geneid=nfu_pred_172_mir-944;coords=sgr01:60174602-60174690

sgr01 nfu_mirna_prediction pre_miRNA 61789919 61789997 . + . ID=nfu_pred_173;biotype=pre-miRNA;gene=;geneid=nfu_pred_173_;coords=sgr01:61789919-61789997

sgr01 nfu_mirna_prediction mature_miRNA 61789929 61789950 . + . ID=nfu_pred_173;biotype=5'-mature-miRNA;gene=;geneid=nfu_pred_173_;coords=sgr01:61789929-61789950

sgr01 nfu_mirna_prediction mature_miRNA 61789964 61789984 . + . ID=nfu_pred_173;biotype=3'-mature-miRNA;gene=;geneid=nfu_pred_173_;coords=sgr01:61789964-61789984

sgr01 nfu_mirna_prediction pre_miRNA 66626187 66626278 . - . ID=nfu_pred_176;biotype=pre-miRNA;gene=mir-190;geneid=nfu_pred_176_mir-190;coords=sgr01:66626187-66626278

sgr01 nfu_mirna_prediction pre_miRNA 68818822 68818924 . + . ID=nfu_pred_177;biotype=pre-miRNA;gene=mir-2189;geneid=nfu_pred_177_mir-2189;coords=sgr01:68818822-68818924

sgr02 nfu_mirna_prediction pre_miRNA 387664 387757 . + . ID=nfu_pred_178;biotype=pre-miRNA;gene=mir-458;geneid=nfu_pred_178_mir-458;coords=sgr02:387664-387757

sgr02 nfu_mirna_prediction pre_miRNA 387796 387889 . + . ID=nfu_pred_179;biotype=pre-miRNA;gene=mir-458;geneid=nfu_pred_179_mir-458;coords=sgr02:387796-387889

sgr02 nfu_mirna_prediction pre_miRNA 565010 565110 . + . ID=nfu_pred_180;biotype=pre-miRNA;gene=mir-455;geneid=nfu_pred_180_mir-455;coords=sgr02:565010-565110

sgr02 nfu_mirna_prediction mature_miRNA 565032 565053 . + . ID=nfu_pred_180;biotype=5'-mature-miRNA;gene=mir-455;geneid=nfu_pred_180_mir-455;coords=sgr02:565032-565053

sgr02 nfu_mirna_prediction mature_miRNA 565069 565090 . + . ID=nfu_pred_180;biotype=3'-mature-miRNA;gene=mir-455;geneid=nfu_pred_180_mir-455;coords=sgr02:565069-565090

sgr02 nfu_mirna_prediction pre_miRNA 737566 737627 . + . ID=nfu_pred_181;biotype=pre-miRNA;gene=mir-122a/mir-3591;geneid=nfu_pred_181_mir-122a/mir-3591;coords=sgr02:737566-737627

sgr02 nfu_mirna_prediction pre_miRNA 3579081 3579210 . + . ID=nfu_pred_182;biotype=pre-miRNA;gene=mir-458;geneid=nfu_pred_182_mir-458;coords=sgr02:3579081-3579210

sgr02 nfu_mirna_prediction pre_miRNA 3854141 3854219 . - . ID=nfu_pred_183;biotype=pre-miRNA;gene=mir-770;geneid=nfu_pred_183_mir-770;coords=sgr02:3854141-3854219

sgr02 nfu_mirna_prediction mature_miRNA 3854188 3854209 . - . ID=nfu_pred_183;biotype=5'-mature-miRNA;gene=mir-770;geneid=nfu_pred_183_mir-770;coords=sgr02:3854188-3854209

sgr02 nfu_mirna_prediction pre_miRNA 6782297 6782391 . + . ID=nfu_pred_184;biotype=pre-miRNA;gene=mir-50;geneid=nfu_pred_184_mir-50;coords=sgr02:6782297-6782391

sgr02 nfu_mirna_prediction mature_miRNA 6782314 6782336 . + . ID=nfu_pred_184;biotype=5'-mature-miRNA;gene=mir-50;geneid=nfu_pred_184_mir-50;coords=sgr02:6782314-6782336

sgr02 nfu_mirna_prediction mature_miRNA 6782350 6782371 . + . ID=nfu_pred_184;biotype=3'-mature-miRNA;gene=mir-50;geneid=nfu_pred_184_mir-50;coords=sgr02:6782350-6782371

sgr02 nfu_mirna_prediction pre_miRNA 7497841 7497919 . + . ID=nfu_pred_185;biotype=pre-miRNA;gene=;geneid=nfu_pred_185_;coords=sgr02:7497841-7497919

sgr02 nfu_mirna_prediction mature_miRNA 7497851 7497872 . + . ID=nfu_pred_185;biotype=5'-mature-miRNA;gene=;geneid=nfu_pred_185_;coords=sgr02:7497851-7497872

sgr02 nfu_mirna_prediction pre_miRNA 7800902 7800990 . + . ID=nfu_pred_186;biotype=pre-miRNA;gene=mir-624;geneid=nfu_pred_186_mir-624;coords=sgr02:7800902-7800990

sgr02 nfu_mirna_prediction pre_miRNA 7800902 7800990 . + . ID=nfu_pred_186;biotype=pre-miRNA;gene=mir-624;geneid=nfu_pred_186_mir-624;coords=sgr02:7800902-7800990

sgr02 nfu_mirna_prediction mature_miRNA 7800923 7800944 . + . ID=nfu_pred_186;biotype=5'-mature-miRNA;gene=mir-624;geneid=nfu_pred_186_mir-624;coords=sgr02:7800923-7800944

sgr02 nfu_mirna_prediction mature_miRNA 7800962 7800983 . + . ID=nfu_pred_186;biotype=3'-mature-miRNA;gene=mir-624;geneid=nfu_pred_186_mir-624;coords=sgr02:7800962-7800983

sgr02 nfu_mirna_prediction pre_miRNA 8274233 8274324 . - . ID=nfu_pred_825;biotype=pre-miRNA;gene=mir-15;geneid=nfu_pred_825_mir-15;coords=sgr02:8274233-8274324

sgr02 nfu_mirna_prediction pre_miRNA 9928382 9928467 . - . ID=nfu_pred_187;biotype=pre-miRNA;gene=mir-338;geneid=nfu_pred_187_mir-338;coords=sgr02:9928382-9928467

sgr02 nfu_mirna_prediction pre_miRNA 11057516 11057596 . + . ID=nfu_pred_188;biotype=pre-miRNA;gene=mir-21;geneid=nfu_pred_188_mir-21;coords=sgr02:11057516-11057596

sgr02 nfu_mirna_prediction mature_miRNA 11057526 11057548 . + . ID=nfu_pred_188;biotype=5'-mature-miRNA;gene=mir-21;geneid=nfu_pred_188_mir-21;coords=sgr02:11057526-11057548

sgr02 nfu_mirna_prediction mature_miRNA 11057565 11057586 . + . ID=nfu_pred_188;biotype=3'-mature-miRNA;gene=mir-21;geneid=nfu_pred_188_mir-21;coords=sgr02:11057565-11057586

sgr02 nfu_mirna_prediction pre_miRNA 11300034 11300110 . - . ID=nfu_pred_189;biotype=pre-miRNA;gene=lin-4;geneid=nfu_pred_189_lin-4;coords=sgr02:11300034-11300110

sgr02 nfu_mirna_prediction mature_miRNA 11300079 11300100 . - . ID=nfu_pred_189;biotype=5'-mature-miRNA;gene=lin-4;geneid=nfu_pred_189_lin-4;coords=sgr02:11300079-11300100

sgr02 nfu_mirna_prediction mature_miRNA 11300038 11300059 . - . ID=nfu_pred_189;biotype=3'-mature-miRNA;gene=lin-4;geneid=nfu_pred_189_lin-4;coords=sgr02:11300038-11300059

sgr02 nfu_mirna_prediction pre_miRNA 11307098 11307173 . - . ID=nfu_pred_190;biotype=pre-miRNA;gene=let-7;geneid=nfu_pred_190_let-7;coords=sgr02:11307098-11307173

sgr02 nfu_mirna_prediction mature_miRNA 11307146 11307167 . - . ID=nfu_pred_190;biotype=5'-mature-miRNA;gene=let-7;geneid=nfu_pred_190_let-7;coords=sgr02:11307146-11307167

sgr02 nfu_mirna_prediction mature_miRNA 11307101 11307122 . - . ID=nfu_pred_190;biotype=3'-mature-miRNA;gene=let-7;geneid=nfu_pred_190_let-7;coords=sgr02:11307101-11307122

sgr02 nfu_mirna_prediction pre_miRNA 11307394 11307470 . - . ID=nfu_pred_191;biotype=pre-miRNA;gene=mir-10;geneid=nfu_pred_191_mir-10;coords=sgr02:11307394-11307470

sgr02 nfu_mirna_prediction mature_miRNA 11307339 11307360 . - . ID=nfu_pred_191;biotype=5'-mature-miRNA;gene=mir-10;geneid=nfu_pred_191_mir-10;coords=sgr02:11307339-11307360

sgr02 nfu_mirna_prediction mature_miRNA 11307403 11307423 . - . ID=nfu_pred_191;biotype=3'-mature-miRNA;gene=mir-10;geneid=nfu_pred_191_mir-10;coords=sgr02:11307403-11307423

sgr02 nfu_mirna_prediction pre_miRNA 11309062 11309146 . - . ID=nfu_pred_192;biotype=pre-miRNA;gene=mir-190;geneid=nfu_pred_192_mir-190;coords=sgr02:11309062-11309146

sgr02 nfu_mirna_prediction pre_miRNA 11907361 11907444 . - . ID=nfu_pred_826;biotype=pre-miRNA;gene=mir-9;geneid=nfu_pred_826_mir-9;coords=sgr02:11907361-11907444

sgr02 nfu_mirna_prediction pre_miRNA 15500711 15500802 . - . ID=nfu_pred_194;biotype=pre-miRNA;gene=mir-145;geneid=nfu_pred_194_mir-145;coords=sgr02:15500711-15500802

sgr02 nfu_mirna_prediction mature_miRNA 15500730 15500750 . - . ID=nfu_pred_194;biotype=5'-mature-miRNA;gene=mir-145;geneid=nfu_pred_194_mir-145;coords=sgr02:15500730-15500750

sgr02 nfu_mirna_prediction mature_miRNA 15500767 15500789 . - . ID=nfu_pred_194;biotype=3'-mature-miRNA;gene=mir-145;geneid=nfu_pred_194_mir-145;coords=sgr02:15500767-15500789

sgr02 nfu_mirna_prediction pre_miRNA 16252787 16252867 . - . ID=nfu_pred_196;biotype=pre-miRNA;gene=;geneid=nfu_pred_196_;coords=sgr02:16252787-16252867

sgr02 nfu_mirna_prediction mature_miRNA 16252835 16252852 . - . ID=nfu_pred_196;biotype=5'-mature-miRNA;gene=;geneid=nfu_pred_196_;coords=sgr02:16252835-16252852

sgr02 nfu_mirna_prediction mature_miRNA 16252797 16252818 . - . ID=nfu_pred_196;biotype=3'-mature-miRNA;gene=;geneid=nfu_pred_196_;coords=sgr02:16252797-16252818

sgr02 nfu_mirna_prediction pre_miRNA 16253537 16253613 . - . ID=nfu_pred_197;biotype=pre-miRNA;gene=lin-4;geneid=nfu_pred_197_lin-4;coords=sgr02:16253537-16253613

sgr02 nfu_mirna_prediction mature_miRNA 16253581 16253603 . - . ID=nfu_pred_197;biotype=5'-mature-miRNA;gene=lin-4;geneid=nfu_pred_197_lin-4;coords=sgr02:16253581-16253603

sgr02 nfu_mirna_prediction mature_miRNA 16253543 16253565 . - . ID=nfu_pred_197;biotype=3'-mature-miRNA;gene=lin-4;geneid=nfu_pred_197_lin-4;coords=sgr02:16253543-16253565

sgr02 nfu_mirna_prediction pre_miRNA 16257306 16257385 . - . ID=nfu_pred_198;biotype=pre-miRNA;gene=let-7;geneid=nfu_pred_198_let-7;coords=sgr02:16257306-16257385

sgr02 nfu_mirna_prediction mature_miRNA 16257355 16257376 . - . ID=nfu_pred_198;biotype=5'-mature-miRNA;gene=let-7;geneid=nfu_pred_198_let-7;coords=sgr02:16257355-16257376

sgr02 nfu_mirna_prediction mature_miRNA 16257310 16257331 . - . ID=nfu_pred_198;biotype=3'-mature-miRNA;gene=let-7;geneid=nfu_pred_198_let-7;coords=sgr02:16257310-16257331

sgr02 nfu_mirna_prediction pre_miRNA 16257740 16257818 . - . ID=nfu_pred_199;biotype=pre-miRNA;gene=mir-10;geneid=nfu_pred_199_mir-10;coords=sgr02:16257740-16257818

sgr02 nfu_mirna_prediction mature_miRNA 16257786 16257808 . - . ID=nfu_pred_199;biotype=3'-mature-miRNA;gene=mir-10;geneid=nfu_pred_199_mir-10;coords=sgr02:16257786-16257808

sgr02 nfu_mirna_prediction mature_miRNA 16257752 16257773 . - . ID=nfu_pred_199;biotype=5'-mature-miRNA;gene=mir-10;geneid=nfu_pred_199_mir-10;coords=sgr02:16257752-16257773

sgr02 nfu_mirna_prediction pre_miRNA 17042962 17043044 . - . ID=nfu_pred_200;biotype=pre-miRNA;gene=mir-2985;geneid=nfu_pred_200_mir-2985;coords=sgr02:17042962-17043044

sgr02 nfu_mirna_prediction mature_miRNA 17042972 17042993 . - . ID=nfu_pred_200;biotype=3'-mature-miRNA;gene=mir-2985;geneid=nfu_pred_200_mir-2985;coords=sgr02:17042972-17042993

sgr02 nfu_mirna_prediction pre_miRNA 18298574 18298688 . - . ID=nfu_pred_201;biotype=pre-miRNA;gene=mir-223;geneid=nfu_pred_201_mir-223;coords=sgr02:18298574-18298688

sgr02 nfu_mirna_prediction mature_miRNA 18298636 18298660 . - . ID=nfu_pred_201;biotype=3'-mature-miRNA;gene=mir-223;geneid=nfu_pred_201_mir-223;coords=sgr02:18298636-18298660

sgr02 nfu_mirna_prediction mature_miRNA 18298600 18298621 . - . ID=nfu_pred_201;biotype=5'-mature-miRNA;gene=mir-223;geneid=nfu_pred_201_mir-223;coords=sgr02:18298600-18298621

sgr02 nfu_mirna_prediction pre_miRNA 21738494 21738568 . + . ID=nfu_pred_202;biotype=pre-miRNA;gene=;geneid=nfu_pred_202_;coords=sgr02:21738494-21738568

sgr02 nfu_mirna_prediction mature_miRNA 21738540 21738561 . + . ID=nfu_pred_202;biotype=5'-mature-miRNA;gene=;geneid=nfu_pred_202_;coords=sgr02:21738540-21738561

sgr02 nfu_mirna_prediction mature_miRNA 21738499 21738521 . + . ID=nfu_pred_202;biotype=3'-mature-miRNA;gene=;geneid=nfu_pred_202_;coords=sgr02:21738499-21738521

sgr02 nfu_mirna_prediction pre_miRNA 22806520 22806609 . - . ID=nfu_pred_203;biotype=pre-miRNA;gene=mir-609;geneid=nfu_pred_203_mir-609;coords=sgr02:22806520-22806609

sgr02 nfu_mirna_prediction pre_miRNA 22870528 22870636 . - . ID=nfu_pred_204;biotype=pre-miRNA;gene=mir-192;geneid=nfu_pred_204_mir-192;coords=sgr02:22870528-22870636

sgr02 nfu_mirna_prediction mature_miRNA 22870592 22870613 . - . ID=nfu_pred_204;biotype=5'-mature-miRNA;gene=mir-192;geneid=nfu_pred_204_mir-192;coords=sgr02:22870592-22870613

sgr02 nfu_mirna_prediction pre_miRNA 22870707 22870790 . - . ID=nfu_pred_205;biotype=pre-miRNA;gene=mir-194;geneid=nfu_pred_205_mir-194;coords=sgr02:22870707-22870790

sgr02 nfu_mirna_prediction mature_miRNA 22870754 22870775 . - . ID=nfu_pred_205;biotype=5'-mature-miRNA;gene=mir-194;geneid=nfu_pred_205_mir-194;coords=sgr02:22870754-22870775

sgr02 nfu_mirna_prediction pre_miRNA 24698334 24698428 . + . ID=nfu_pred_206;biotype=pre-miRNA;gene=mir-672;geneid=nfu_pred_206_mir-672;coords=sgr02:24698334-24698428

sgr02 nfu_mirna_prediction pre_miRNA 30159146 30159250 . + . ID=nfu_pred_207;biotype=pre-miRNA;gene=mir-3661;geneid=nfu_pred_207_mir-3661;coords=sgr02:30159146-30159250

sgr02 nfu_mirna_prediction pre_miRNA 30511425 30511534 . + . ID=nfu_pred_208;biotype=pre-miRNA;gene=mir-455;geneid=nfu_pred_208_mir-455;coords=sgr02:30511425-30511534

sgr02 nfu_mirna_prediction mature_miRNA 30511446 30511467 . + . ID=nfu_pred_208;biotype=5'-mature-miRNA;gene=mir-455;geneid=nfu_pred_208_mir-455;coords=sgr02:30511446-30511467

sgr02 nfu_mirna_prediction mature_miRNA 30511481 30511501 . + . ID=nfu_pred_208;biotype=3'-mature-miRNA;gene=mir-455;geneid=nfu_pred_208_mir-455;coords=sgr02:30511481-30511501

sgr02 nfu_mirna_prediction pre_miRNA 30512413 30512512 . - . ID=nfu_pred_209;biotype=pre-miRNA;gene=mir-132b;geneid=nfu_pred_209_mir-132b;coords=sgr02:30512413-30512512

sgr02 nfu_mirna_prediction mature_miRNA 30512471 30512492 . - . ID=nfu_pred_209;biotype=3'-mature-miRNA;gene=mir-132b;geneid=nfu_pred_209_mir-132b;coords=sgr02:30512471-30512492

sgr02 nfu_mirna_prediction mature_miRNA 30512431 30512452 . - . ID=nfu_pred_209;biotype=5'-mature-miRNA;gene=mir-132b;geneid=nfu_pred_209_mir-132b;coords=sgr02:30512431-30512452

sgr02 nfu_mirna_prediction pre_miRNA 30512852 30512946 . - . ID=nfu_pred_210;biotype=pre-miRNA;gene=mir-132;geneid=nfu_pred_210_mir-132;coords=sgr02:30512852-30512946

sgr02 nfu_mirna_prediction mature_miRNA 30512909 30512930 . - . ID=nfu_pred_210;biotype=5'-mature-miRNA;gene=mir-132;geneid=nfu_pred_210_mir-132;coords=sgr02:30512909-30512930

sgr02 nfu_mirna_prediction mature_miRNA 30512867 30512885 . - . ID=nfu_pred_210;biotype=3'-mature-miRNA;gene=mir-132;geneid=nfu_pred_210_mir-132;coords=sgr02:30512867-30512885

sgr02 nfu_mirna_prediction pre_miRNA 31174683 31174770 . + . ID=nfu_pred_211;biotype=pre-miRNA;gene=mir-22;geneid=nfu_pred_211_mir-22;coords=sgr02:31174683-31174770

sgr02 nfu_mirna_prediction mature_miRNA 31174697 31174717 . + . ID=nfu_pred_211;biotype=5'-mature-miRNA;gene=mir-22;geneid=nfu_pred_211_mir-22;coords=sgr02:31174697-31174717

sgr02 nfu_mirna_prediction mature_miRNA 31174738 31174758 . + . ID=nfu_pred_211;biotype=3'-mature-miRNA;gene=mir-22;geneid=nfu_pred_211_mir-22;coords=sgr02:31174738-31174758

sgr02 nfu_mirna_prediction pre_miRNA 32612291 32612367 . - . ID=nfu_pred_212;biotype=pre-miRNA;gene=mir-21;geneid=nfu_pred_212_mir-21;coords=sgr02:32612291-32612367

sgr02 nfu_mirna_prediction mature_miRNA 32612339 32612360 . - . ID=nfu_pred_212;biotype=5'-mature-miRNA;gene=mir-21;geneid=nfu_pred_212_mir-21;coords=sgr02:32612339-32612360

sgr02 nfu_mirna_prediction mature_miRNA 32612299 32612321 . - . ID=nfu_pred_212;biotype=3'-mature-miRNA;gene=mir-21;geneid=nfu_pred_212_mir-21;coords=sgr02:32612299-32612321

sgr02 nfu_mirna_prediction pre_miRNA 32935553 32935665 . - . ID=nfu_pred_213;biotype=pre-miRNA;gene=mir-214/mir-3120;geneid=nfu_pred_213_mir-214/mir-3120;coords=sgr02:32935553-32935665

sgr02 nfu_mirna_prediction pre_miRNA 35351829 35351909 . + . ID=nfu_pred_214;biotype=pre-miRNA;gene=mir-939;geneid=nfu_pred_214_mir-939;coords=sgr02:35351829-35351909

sgr02 nfu_mirna_prediction mature_miRNA 35351877 35351898 . + . ID=nfu_pred_214;biotype=3'-mature-miRNA;gene=mir-939;geneid=nfu_pred_214_mir-939;coords=sgr02:35351877-35351898

sgr02 nfu_mirna_prediction pre_miRNA 37123377 37123457 . - . ID=nfu_pred_215;biotype=pre-miRNA;gene=;geneid=nfu_pred_215_;coords=sgr02:37123377-37123457

sgr02 nfu_mirna_prediction pre_miRNA 37123519 37123629 . - . ID=nfu_pred_216;biotype=pre-miRNA;gene=mir-142;geneid=nfu_pred_216_mir-142;coords=sgr02:37123519-37123629

sgr02 nfu_mirna_prediction mature_miRNA 37123582 37123603 . - . ID=nfu_pred_216;biotype=5'-mature-miRNA;gene=mir-142;geneid=nfu_pred_216_mir-142;coords=sgr02:37123582-37123603

sgr02 nfu_mirna_prediction mature_miRNA 37123544 37123566 . - . ID=nfu_pred_216;biotype=3'-mature-miRNA;gene=mir-142;geneid=nfu_pred_216_mir-142;coords=sgr02:37123544-37123566

sgr02 nfu_mirna_prediction pre_miRNA 37527479 37527564 . + . ID=nfu_pred_217;biotype=pre-miRNA;gene=mir-144;geneid=nfu_pred_217_mir-144;coords=sgr02:37527479-37527564

sgr02 nfu_mirna_prediction mature_miRNA 37527492 37527515 . + . ID=nfu_pred_217;biotype=5'-mature-miRNA;gene=mir-144;geneid=nfu_pred_217_mir-144;coords=sgr02:37527492-37527515

sgr02 nfu_mirna_prediction mature_miRNA 37527530 37527550 . + . ID=nfu_pred_217;biotype=3'-mature-miRNA;gene=mir-144;geneid=nfu_pred_217_mir-144;coords=sgr02:37527530-37527550

sgr02 nfu_mirna_prediction pre_miRNA 37527607 37527680 . + . ID=nfu_pred_218;biotype=pre-miRNA;gene=mir-451;geneid=nfu_pred_218_mir-451;coords=sgr02:37527607-37527680

sgr02 nfu_mirna_prediction mature_miRNA 37527623 37527645 . + . ID=nfu_pred_218;biotype=5'-mature-miRNA;gene=mir-451;geneid=nfu_pred_218_mir-451;coords=sgr02:37527623-37527645

sgr02 nfu_mirna_prediction pre_miRNA 41744628 41744708 . + . ID=nfu_pred_219;biotype=pre-miRNA;gene=;geneid=nfu_pred_219_;coords=sgr02:41744628-41744708

sgr02 nfu_mirna_prediction mature_miRNA 41744638 41744659 . + . ID=nfu_pred_219;biotype=5'-mature-miRNA;gene=;geneid=nfu_pred_219_;coords=sgr02:41744638-41744659

sgr02 nfu_mirna_prediction mature_miRNA 41744676 41744698 . + . ID=nfu_pred_219;biotype=3'-mature-miRNA;gene=;geneid=nfu_pred_219_;coords=sgr02:41744676-41744698

sgr02 nfu_mirna_prediction pre_miRNA 44218028 44218121 . + . ID=nfu_pred_220;biotype=pre-miRNA;gene=mir-642;geneid=nfu_pred_220_mir-642;coords=sgr02:44218028-44218121

sgr02 nfu_mirna_prediction mature_miRNA 44218075 44218096 . + . ID=nfu_pred_220;biotype=3'-mature-miRNA;gene=mir-642;geneid=nfu_pred_220_mir-642;coords=sgr02:44218075-44218096

sgr02 nfu_mirna_prediction pre_miRNA 45879608 45879693 . + . ID=nfu_pred_222;biotype=pre-miRNA;gene=mir-2985;geneid=nfu_pred_222_mir-2985;coords=sgr02:45879608-45879693

sgr02 nfu_mirna_prediction pre_miRNA 47627662 47627747 . + . ID=nfu_pred_223;biotype=pre-miRNA;gene=mir-33;geneid=nfu_pred_223_mir-33;coords=sgr02:47627662-47627747

sgr03 nfu_mirna_prediction pre_miRNA 248311 248394 . + . ID=nfu_pred_224;biotype=pre-miRNA;gene=mir-548;geneid=nfu_pred_224_mir-548;coords=sgr03:248311-248394

sgr03 nfu_mirna_prediction mature_miRNA 248361 248382 . + . ID=nfu_pred_224;biotype=3'-mature-miRNA;gene=mir-548;geneid=nfu_pred_224_mir-548;coords=sgr03:248361-248382

sgr03 nfu_mirna_prediction pre_miRNA 1866226 1866306 . + . ID=nfu_pred_225;biotype=pre-miRNA;gene=mir-138;geneid=nfu_pred_225_mir-138;coords=sgr03:1866226-1866306

sgr03 nfu_mirna_prediction mature_miRNA 1866236 1866258 . + . ID=nfu_pred_225;biotype=5'-mature-miRNA;gene=mir-138;geneid=nfu_pred_225_mir-138;coords=sgr03:1866236-1866258

sgr03 nfu_mirna_prediction mature_miRNA 1866276 1866297 . + . ID=nfu_pred_225;biotype=3'-mature-miRNA;gene=mir-138;geneid=nfu_pred_225_mir-138;coords=sgr03:1866276-1866297

sgr03 nfu_mirna_prediction pre_miRNA 8520773 8520870 . + . ID=nfu_pred_227;biotype=pre-miRNA;gene=mir-489;geneid=nfu_pred_227_mir-489;coords=sgr03:8520773-8520870

sgr03 nfu_mirna_prediction mature_miRNA 8520791 8520812 . + . ID=nfu_pred_227;biotype=5'-mature-miRNA;gene=mir-489;geneid=nfu_pred_227_mir-489;coords=sgr03:8520791-8520812

sgr03 nfu_mirna_prediction mature_miRNA 8520830 8520852 . + . ID=nfu_pred_227;biotype=3'-mature-miRNA;gene=mir-489;geneid=nfu_pred_227_mir-489;coords=sgr03:8520830-8520852

sgr03 nfu_mirna_prediction pre_miRNA 10059233 10059326 . + . ID=nfu_pred_228;biotype=pre-miRNA;gene=mir-642;geneid=nfu_pred_228_mir-642;coords=sgr03:10059233-10059326

sgr03 nfu_mirna_prediction mature_miRNA 10059282 10059303 . + . ID=nfu_pred_228;biotype=3'-mature-miRNA;gene=mir-642;geneid=nfu_pred_228_mir-642;coords=sgr03:10059282-10059303

sgr03 nfu_mirna_prediction pre_miRNA 11106946 11107035 . - . ID=nfu_pred_229;biotype=pre-miRNA;gene=mir-196;geneid=nfu_pred_229_mir-196;coords=sgr03:11106946-11107035

sgr03 nfu_mirna_prediction mature_miRNA 11106996 11107017 . - . ID=nfu_pred_229;biotype=5'-mature-miRNA;gene=mir-196;geneid=nfu_pred_229_mir-196;coords=sgr03:11106996-11107017

sgr03 nfu_mirna_prediction mature_miRNA 11106962 11106982 . - . ID=nfu_pred_229;biotype=3'-mature-miRNA;gene=mir-196;geneid=nfu_pred_229_mir-196;coords=sgr03:11106962-11106982

sgr03 nfu_mirna_prediction pre_miRNA 14947294 14947347 . + . ID=nfu_pred_230;biotype=pre-miRNA;gene=mir-669a;geneid=nfu_pred_230_mir-669a;coords=sgr03:14947294-14947347

sgr03 nfu_mirna_prediction pre_miRNA 18773198 18773254 . + . ID=nfu_pred_231;biotype=pre-miRNA;gene=mir-7641;geneid=nfu_pred_231_mir-7641;coords=sgr03:18773198-18773254

sgr03 nfu_mirna_prediction pre_miRNA 18797529 18797641 . - . ID=nfu_pred_232;biotype=pre-miRNA;gene=mir-214/mir-3120;geneid=nfu_pred_232_mir-214/mir-3120;coords=sgr03:18797529-18797641

sgr03 nfu_mirna_prediction pre_miRNA 18801583 18801695 . - . ID=nfu_pred_233;biotype=pre-miRNA;gene=mir-214/mir-3120;geneid=nfu_pred_233_mir-214/mir-3120;coords=sgr03:18801583-18801695

sgr03 nfu_mirna_prediction pre_miRNA 20175958 20176044 . + . ID=nfu_pred_234;biotype=pre-miRNA;gene=mir-153;geneid=nfu_pred_234_mir-153;coords=sgr03:20175958-20176044

sgr03 nfu_mirna_prediction mature_miRNA 20175971 20175993 . + . ID=nfu_pred_234;biotype=5'-mature-miRNA;gene=mir-153;geneid=nfu_pred_234_mir-153;coords=sgr03:20175971-20175993

sgr03 nfu_mirna_prediction mature_miRNA 20176010 20176031 . + . ID=nfu_pred_234;biotype=3'-mature-miRNA;gene=mir-153;geneid=nfu_pred_234_mir-153;coords=sgr03:20176010-20176031

sgr03 nfu_mirna_prediction pre_miRNA 21046952 21047032 . + . ID=nfu_pred_235;biotype=pre-miRNA;gene=mir-4683;geneid=nfu_pred_235_mir-4683;coords=sgr03:21046952-21047032

sgr03 nfu_mirna_prediction pre_miRNA 23156858 23156934 . - . ID=nfu_pred_237;biotype=pre-miRNA;gene=;geneid=nfu_pred_237_;coords=sgr03:23156858-23156934

sgr03 nfu_mirna_prediction mature_miRNA 23156868 23156888 . - . ID=nfu_pred_237;biotype=3'-mature-miRNA;gene=;geneid=nfu_pred_237_;coords=sgr03:23156868-23156888

sgr03 nfu_mirna_prediction pre_miRNA 25720086 25720151 . + . ID=nfu_pred_238;biotype=pre-miRNA;gene=;geneid=nfu_pred_238_;coords=sgr03:25720086-25720151

sgr03 nfu_mirna_prediction mature_miRNA 25720094 25720115 . + . ID=nfu_pred_238;biotype=5'-mature-miRNA;gene=;geneid=nfu_pred_238_;coords=sgr03:25720094-25720115

sgr03 nfu_mirna_prediction mature_miRNA 25720127 25720150 . + . ID=nfu_pred_238;biotype=3'-mature-miRNA;gene=;geneid=nfu_pred_238_;coords=sgr03:25720127-25720150

sgr03 nfu_mirna_prediction pre_miRNA 29288397 29288465 . - . ID=nfu_pred_239;biotype=pre-miRNA;gene=mir-24;geneid=nfu_pred_239_mir-24;coords=sgr03:29288397-29288465

sgr03 nfu_mirna_prediction mature_miRNA 29288439 29288459 . - . ID=nfu_pred_239;biotype=5'-mature-miRNA;gene=mir-24;geneid=nfu_pred_239_mir-24;coords=sgr03:29288439-29288459

sgr03 nfu_mirna_prediction mature_miRNA 29288401 29288421 . - . ID=nfu_pred_239;biotype=3'-mature-miRNA;gene=mir-24;geneid=nfu_pred_239_mir-24;coords=sgr03:29288401-29288421

sgr03 nfu_mirna_prediction pre_miRNA 29288911 29289009 . - . ID=nfu_pred_240;biotype=pre-miRNA;gene=mir-27;geneid=nfu_pred_240_mir-27;coords=sgr03:29288911-29289009

sgr03 nfu_mirna_prediction mature_miRNA 29288929 29288950 . - . ID=nfu_pred_240;biotype=3'-mature-miRNA;gene=mir-27;geneid=nfu_pred_240_mir-27;coords=sgr03:29288929-29288950

sgr03 nfu_mirna_prediction pre_miRNA 31902689 31902787 . + . ID=nfu_pred_241;biotype=pre-miRNA;gene=mir-23;geneid=nfu_pred_241_mir-23;coords=sgr03:31902689-31902787

sgr03 nfu_mirna_prediction mature_miRNA 31902747 31902768 . + . ID=nfu_pred_241;biotype=3'-mature-miRNA;gene=mir-23;geneid=nfu_pred_241_mir-23;coords=sgr03:31902747-31902768

sgr03 nfu_mirna_prediction pre_miRNA 31904671 31904768 . + . ID=nfu_pred_242;biotype=pre-miRNA;gene=mir-27;geneid=nfu_pred_242_mir-27;coords=sgr03:31904671-31904768

sgr03 nfu_mirna_prediction mature_miRNA 31904728 31904750 . + . ID=nfu_pred_242;biotype=3'-mature-miRNA;gene=mir-27;geneid=nfu_pred_242_mir-27;coords=sgr03:31904728-31904750

sgr03 nfu_mirna_prediction pre_miRNA 31908159 31908239 . + . ID=nfu_pred_243;biotype=pre-miRNA;gene=mir-24;geneid=nfu_pred_243_mir-24;coords=sgr03:31908159-31908239

sgr03 nfu_mirna_prediction mature_miRNA 31908206 31908226 . + . ID=nfu_pred_243;biotype=3'-mature-miRNA;gene=mir-24;geneid=nfu_pred_243_mir-24;coords=sgr03:31908206-31908226

sgr03 nfu_mirna_prediction pre_miRNA 32325457 32325537 . + . ID=nfu_pred_244;biotype=pre-miRNA;gene=mir-1;geneid=nfu_pred_244_mir-1;coords=sgr03:32325457-32325537

sgr03 nfu_mirna_prediction mature_miRNA 32325506 32325527 . + . ID=nfu_pred_244;biotype=3'-mature-miRNA;gene=mir-1;geneid=nfu_pred_244_mir-1;coords=sgr03:32325506-32325527

sgr03 nfu_mirna_prediction pre_miRNA 32328734 32328820 . + . ID=nfu_pred_245;biotype=pre-miRNA;gene=mir-133;geneid=nfu_pred_245_mir-133;coords=sgr03:32328734-32328820

sgr03 nfu_mirna_prediction mature_miRNA 32328784 32328806 . + . ID=nfu_pred_245;biotype=3'-mature-miRNA;gene=mir-133;geneid=nfu_pred_245_mir-133;coords=sgr03:32328784-32328806

sgr03 nfu_mirna_prediction pre_miRNA 33171215 33171298 . + . ID=nfu_pred_246;biotype=pre-miRNA;gene=mir-460;geneid=nfu_pred_246_mir-460;coords=sgr03:33171215-33171298

sgr03 nfu_mirna_prediction mature_miRNA 33171228 33171248 . + . ID=nfu_pred_246;biotype=5'-mature-miRNA;gene=mir-460;geneid=nfu_pred_246_mir-460;coords=sgr03:33171228-33171248

sgr03 nfu_mirna_prediction mature_miRNA 33171265 33171286 . + . ID=nfu_pred_246;biotype=3'-mature-miRNA;gene=mir-460;geneid=nfu_pred_246_mir-460;coords=sgr03:33171265-33171286

sgr03 nfu_mirna_prediction pre_miRNA 33954276 33954358 . - . ID=nfu_pred_247;biotype=pre-miRNA;gene=mir-548;geneid=nfu_pred_247_mir-548;coords=sgr03:33954276-33954358

sgr03 nfu_mirna_prediction mature_miRNA 33954321 33954342 . - . ID=nfu_pred_247;biotype=5'-mature-miRNA;gene=mir-548;geneid=nfu_pred_247_mir-548;coords=sgr03:33954321-33954342

sgr03 nfu_mirna_prediction mature_miRNA 33954283 33954305 . - . ID=nfu_pred_247;biotype=3'-mature-miRNA;gene=mir-548;geneid=nfu_pred_247_mir-548;coords=sgr03:33954283-33954305

sgr03 nfu_mirna_prediction pre_miRNA 34749361 34749504 . - . ID=nfu_pred_248;biotype=pre-miRNA;gene=mir-8994;geneid=nfu_pred_248_mir-8994;coords=sgr03:34749361-34749504

sgr03 nfu_mirna_prediction pre_miRNA 35127088 35127185 . + . ID=nfu_pred_249;biotype=pre-miRNA;gene=mir-6720;geneid=nfu_pred_249_mir-6720;coords=sgr03:35127088-35127185

sgr03 nfu_mirna_prediction pre_miRNA 35133176 35133273 . + . ID=nfu_pred_250;biotype=pre-miRNA;gene=mir-6720;geneid=nfu_pred_250_mir-6720;coords=sgr03:35133176-35133273

sgr03 nfu_mirna_prediction pre_miRNA 35136792 35136889 . + . ID=nfu_pred_251;biotype=pre-miRNA;gene=mir-6720;geneid=nfu_pred_251_mir-6720;coords=sgr03:35136792-35136889

sgr03 nfu_mirna_prediction pre_miRNA 36009052 36009095 . - . ID=nfu_pred_252;biotype=pre-miRNA;gene=mir-7549;geneid=nfu_pred_252_mir-7549;coords=sgr03:36009052-36009095

sgr03 nfu_mirna_prediction pre_miRNA 36458339 36458444 . + . ID=nfu_pred_253;biotype=pre-miRNA;gene=mir-6698;geneid=nfu_pred_253_mir-6698;coords=sgr03:36458339-36458444

sgr03 nfu_mirna_prediction pre_miRNA 36554680 36554773 . - . ID=nfu_pred_254;biotype=pre-miRNA;gene=mir-338;geneid=nfu_pred_254_mir-338;coords=sgr03:36554680-36554773

sgr03 nfu_mirna_prediction mature_miRNA 36554735 36554756 . - . ID=nfu_pred_254;biotype=5'-mature-miRNA;gene=mir-338;geneid=nfu_pred_254_mir-338;coords=sgr03:36554735-36554756

sgr03 nfu_mirna_prediction pre_miRNA 41166518 41166600 . - . ID=nfu_pred_255;biotype=pre-miRNA;gene=mir-181;geneid=nfu_pred_255_mir-181;coords=sgr03:41166518-41166600

sgr03 nfu_mirna_prediction mature_miRNA 41166567 41166590 . - . ID=nfu_pred_255;biotype=5'-mature-miRNA;gene=mir-181;geneid=nfu_pred_255_mir-181;coords=sgr03:41166567-41166590

sgr03 nfu_mirna_prediction pre_miRNA 41166724 41166840 . - . ID=nfu_pred_256;biotype=pre-miRNA;gene=mir-181;geneid=nfu_pred_256_mir-181;coords=sgr03:41166724-41166840

sgr03 nfu_mirna_prediction mature_miRNA 41166785 41166807 . - . ID=nfu_pred_256;biotype=5'-mature-miRNA;gene=mir-181;geneid=nfu_pred_256_mir-181;coords=sgr03:41166785-41166807

sgr03 nfu_mirna_prediction mature_miRNA 41166743 41166765 . - . ID=nfu_pred_256;biotype=3'-mature-miRNA;gene=mir-181;geneid=nfu_pred_256_mir-181;coords=sgr03:41166743-41166765

sgr03 nfu_mirna_prediction pre_miRNA 41513309 41513388 . + . ID=nfu_pred_257;biotype=pre-miRNA;gene=mir-9;geneid=nfu_pred_257_mir-9;coords=sgr03:41513309-41513388

sgr03 nfu_mirna_prediction mature_miRNA 41513320 41513342 . + . ID=nfu_pred_257;biotype=5'-mature-miRNA;gene=mir-9;geneid=nfu_pred_257_mir-9;coords=sgr03:41513320-41513342

sgr03 nfu_mirna_prediction mature_miRNA 41513355 41513376 . + . ID=nfu_pred_257;biotype=3'-mature-miRNA;gene=mir-9;geneid=nfu_pred_257_mir-9;coords=sgr03:41513355-41513376

sgr03 nfu_mirna_prediction pre_miRNA 41706471 41706558 . + . ID=nfu_pred_258;biotype=pre-miRNA;gene=mir-263;geneid=nfu_pred_258_mir-263;coords=sgr03:41706471-41706558

sgr03 nfu_mirna_prediction mature_miRNA 41706485 41706508 . + . ID=nfu_pred_258;biotype=5'-mature-miRNA;gene=mir-263;geneid=nfu_pred_258_mir-263;coords=sgr03:41706485-41706508

sgr03 nfu_mirna_prediction mature_miRNA 41706525 41706546 . + . ID=nfu_pred_258;biotype=3'-mature-miRNA;gene=mir-263;geneid=nfu_pred_258_mir-263;coords=sgr03:41706525-41706546

sgr03 nfu_mirna_prediction pre_miRNA 41785309 41785395 . - . ID=nfu_pred_259;biotype=pre-miRNA;gene=mir-7;geneid=nfu_pred_259_mir-7;coords=sgr03:41785309-41785395

sgr03 nfu_mirna_prediction mature_miRNA 41785321 41785342 . - . ID=nfu_pred_259;biotype=5'-mature-miRNA;gene=mir-7;geneid=nfu_pred_259_mir-7;coords=sgr03:41785321-41785342

sgr03 nfu_mirna_prediction mature_miRNA 41785359 41785381 . - . ID=nfu_pred_259;biotype=3'-mature-miRNA;gene=mir-7;geneid=nfu_pred_259_mir-7;coords=sgr03:41785359-41785381

sgr03 nfu_mirna_prediction pre_miRNA 42340399 42340503 . - . ID=nfu_pred_260;biotype=pre-miRNA;gene=mir-48;geneid=nfu_pred_260_mir-48;coords=sgr03:42340399-42340503

sgr03 nfu_mirna_prediction mature_miRNA 42340423 42340444 . - . ID=nfu_pred_260;biotype=3'-mature-miRNA;gene=mir-48;geneid=nfu_pred_260_mir-48;coords=sgr03:42340423-42340444

sgr03 nfu_mirna_prediction pre_miRNA 46010663 46010749 . + . ID=nfu_pred_261;biotype=pre-miRNA;gene=mir-665;geneid=nfu_pred_261_mir-665;coords=sgr03:46010663-46010749

sgr03 nfu_mirna_prediction mature_miRNA 46010673 46010695 . + . ID=nfu_pred_261;biotype=5'-mature-miRNA;gene=mir-665;geneid=nfu_pred_261_mir-665;coords=sgr03:46010673-46010695

sgr03 nfu_mirna_prediction mature_miRNA 46010713 46010735 . + . ID=nfu_pred_261;biotype=3'-mature-miRNA;gene=mir-665;geneid=nfu_pred_261_mir-665;coords=sgr03:46010713-46010735

sgr03 nfu_mirna_prediction pre_miRNA 49916501 49916579 . + . ID=nfu_pred_263;biotype=pre-miRNA;gene=mir-26;geneid=nfu_pred_263_mir-26;coords=sgr03:49916501-49916579

sgr03 nfu_mirna_prediction mature_miRNA 49916511 49916532 . + . ID=nfu_pred_263;biotype=5'-mature-miRNA;gene=mir-26;geneid=nfu_pred_263_mir-26;coords=sgr03:49916511-49916532

sgr03 nfu_mirna_prediction pre_miRNA 51442557 51442635 . - . ID=nfu_pred_265;biotype=pre-miRNA;gene=lsy-6;geneid=nfu_pred_265_lsy-6;coords=sgr03:51442557-51442635

sgr03 nfu_mirna_prediction mature_miRNA 51442603 51442625 . - . ID=nfu_pred_265;biotype=5'-mature-miRNA;gene=lsy-6;geneid=nfu_pred_265_lsy-6;coords=sgr03:51442603-51442625

sgr03 nfu_mirna_prediction mature_miRNA 51442564 51442587 . - . ID=nfu_pred_265;biotype=3'-mature-miRNA;gene=lsy-6;geneid=nfu_pred_265_lsy-6;coords=sgr03:51442564-51442587

sgr03 nfu_mirna_prediction pre_miRNA 51452619 51452716 . - . ID=nfu_pred_266;biotype=pre-miRNA;gene=mir-727;geneid=nfu_pred_266_mir-727;coords=sgr03:51452619-51452716

sgr03 nfu_mirna_prediction mature_miRNA 51452677 51452698 . - . ID=nfu_pred_266;biotype=5'-mature-miRNA;gene=mir-727;geneid=nfu_pred_266_mir-727;coords=sgr03:51452677-51452698

sgr03 nfu_mirna_prediction mature_miRNA 51452634 51452655 . - . ID=nfu_pred_266;biotype=3'-mature-miRNA;gene=mir-727;geneid=nfu_pred_266_mir-727;coords=sgr03:51452634-51452655

sgr03 nfu_mirna_prediction pre_miRNA 52870861 52870939 . - . ID=nfu_pred_267;biotype=pre-miRNA;gene=mir-944;geneid=nfu_pred_267_mir-944;coords=sgr03:52870861-52870939

sgr03 nfu_mirna_prediction mature_miRNA 52870865 52870885 . - . ID=nfu_pred_267;biotype=3'-mature-miRNA;gene=mir-944;geneid=nfu_pred_267_mir-944;coords=sgr03:52870865-52870885

sgr03 nfu_mirna_prediction mature_miRNA 52870912 52870933 . - . ID=nfu_pred_267;biotype=5'-mature-miRNA;gene=mir-944;geneid=nfu_pred_267_mir-944;coords=sgr03:52870912-52870933

sgr03 nfu_mirna_prediction pre_miRNA 53791309 53791389 . - . ID=nfu_pred_268;biotype=pre-miRNA;gene=mir-124;geneid=nfu_pred_268_mir-124;coords=sgr03:53791309-53791389

sgr03 nfu_mirna_prediction mature_miRNA 53791320 53791341 . - . ID=nfu_pred_268;biotype=3'-mature-miRNA;gene=mir-124;geneid=nfu_pred_268_mir-124;coords=sgr03:53791320-53791341

sgr04 nfu_mirna_prediction pre_miRNA 446156 446264 . + . ID=nfu_pred_269;biotype=pre-miRNA;gene=;geneid=nfu_pred_269_;coords=sgr04:446156-446264

sgr04 nfu_mirna_prediction mature_miRNA 446181 446202 . + . ID=nfu_pred_269;biotype=5'-mature-miRNA;gene=;geneid=nfu_pred_269_;coords=sgr04:446181-446202

sgr04 nfu_mirna_prediction mature_miRNA 446221 446242 . + . ID=nfu_pred_269;biotype=3'-mature-miRNA;gene=;geneid=nfu_pred_269_;coords=sgr04:446221-446242

sgr04 nfu_mirna_prediction pre_miRNA 709012 709092 . - . ID=nfu_pred_270;biotype=pre-miRNA;gene=mir-181;geneid=nfu_pred_270_mir-181;coords=sgr04:709012-709092

sgr04 nfu_mirna_prediction mature_miRNA 709060 709082 . - . ID=nfu_pred_270;biotype=5'-mature-miRNA;gene=mir-181;geneid=nfu_pred_270_mir-181;coords=sgr04:709060-709082

sgr04 nfu_mirna_prediction pre_miRNA 712524 712634 . - . ID=nfu_pred_271;biotype=pre-miRNA;gene=mir-181;geneid=nfu_pred_271_mir-181;coords=sgr04:712524-712634

sgr04 nfu_mirna_prediction mature_miRNA 712581 712603 . - . ID=nfu_pred_271;biotype=5'-mature-miRNA;gene=mir-3570;geneid=nfu_pred_271_mir-3570;coords=sgr04:712581-712603

sgr04 nfu_mirna_prediction pre_miRNA 1949048 1949134 . - . ID=nfu_pred_272;biotype=pre-miRNA;gene=mir-150;geneid=nfu_pred_272_mir-150;coords=sgr04:1949048-1949134

sgr04 nfu_mirna_prediction mature_miRNA 1949101 1949123 . - . ID=nfu_pred_272;biotype=5'-mature-miRNA;gene=mir-150;geneid=nfu_pred_272_mir-150;coords=sgr04:1949101-1949123

sgr04 nfu_mirna_prediction mature_miRNA 1949064 1949086 . - . ID=nfu_pred_272;biotype=3'-mature-miRNA;gene=mir-150;geneid=nfu_pred_272_mir-150;coords=sgr04:1949064-1949086

sgr04 nfu_mirna_prediction pre_miRNA 2272841 2272924 . + . ID=nfu_pred_273;biotype=pre-miRNA;gene=mir-995;geneid=nfu_pred_273_mir-995;coords=sgr04:2272841-2272924

sgr04 nfu_mirna_prediction mature_miRNA 2272853 2272874 . + . ID=nfu_pred_273;biotype=5'-mature-miRNA;gene=mir-995;geneid=nfu_pred_273_mir-995;coords=sgr04:2272853-2272874

sgr04 nfu_mirna_prediction mature_miRNA 2272891 2272911 . + . ID=nfu_pred_273;biotype=3'-mature-miRNA;gene=mir-995;geneid=nfu_pred_273_mir-995;coords=sgr04:2272891-2272911

sgr04 nfu_mirna_prediction pre_miRNA 3126658 3126736 . - . ID=nfu_pred_274;biotype=pre-miRNA;gene=mir-46;geneid=nfu_pred_274_mir-46;coords=sgr04:3126658-3126736

sgr04 nfu_mirna_prediction mature_miRNA 3126671 3126690 . - . ID=nfu_pred_274;biotype=3'-mature-miRNA;gene=mir-46;geneid=nfu_pred_274_mir-46;coords=sgr04:3126671-3126690

sgr04 nfu_mirna_prediction pre_miRNA 4912701 4912781 . + . ID=nfu_pred_275;biotype=pre-miRNA;gene=mir-396;geneid=nfu_pred_275_mir-396;coords=sgr04:4912701-4912781

sgr04 nfu_mirna_prediction mature_miRNA 4912707 4912728 . + . ID=nfu_pred_275;biotype=5'-mature-miRNA;gene=mir-396;geneid=nfu_pred_275_mir-396;coords=sgr04:4912707-4912728

sgr04 nfu_mirna_prediction mature_miRNA 4912748 4912769 . + . ID=nfu_pred_275;biotype=3'-mature-miRNA;gene=mir-396;geneid=nfu_pred_275_mir-396;coords=sgr04:4912748-4912769

sgr04 nfu_mirna_prediction pre_miRNA 5867206 5867303 . - . ID=nfu_pred_276;biotype=pre-miRNA;gene=mir-6720;geneid=nfu_pred_276_mir-6720;coords=sgr04:5867206-5867303

sgr04 nfu_mirna_prediction pre_miRNA 9983798 9983876 . + . ID=nfu_pred_278;biotype=pre-miRNA;gene=;geneid=nfu_pred_278_;coords=sgr04:9983798-9983876

sgr04 nfu_mirna_prediction mature_miRNA 9983808 9983829 . + . ID=nfu_pred_278;biotype=5'-mature-miRNA;gene=;geneid=nfu_pred_278_;coords=sgr04:9983808-9983829

sgr04 nfu_mirna_prediction mature_miRNA 9983835 9983856 . + . ID=nfu_pred_278;biotype=3'-mature-miRNA;gene=;geneid=nfu_pred_278_;coords=sgr04:9983835-9983856

sgr04 nfu_mirna_prediction pre_miRNA 10366126 10366216 . + . ID=nfu_pred_279;biotype=pre-miRNA;gene=mir-651;geneid=nfu_pred_279_mir-651;coords=sgr04:10366126-10366216

sgr04 nfu_mirna_prediction mature_miRNA 10366175 10366195 . + . ID=nfu_pred_279;biotype=3'-mature-miRNA;gene=mir-651;geneid=nfu_pred_279_mir-651;coords=sgr04:10366175-10366195

sgr04 nfu_mirna_prediction pre_miRNA 12773233 12773327 . + . ID=nfu_pred_280;biotype=pre-miRNA;gene=mir-56;geneid=nfu_pred_280_mir-56;coords=sgr04:12773233-12773327

sgr04 nfu_mirna_prediction mature_miRNA 12773250 12773272 . + . ID=nfu_pred_280;biotype=5'-mature-miRNA;gene=mir-56;geneid=nfu_pred_280_mir-56;coords=sgr04:12773250-12773272

sgr04 nfu_mirna_prediction mature_miRNA 12773291 12773311 . + . ID=nfu_pred_280;biotype=3'-mature-miRNA;gene=mir-56;geneid=nfu_pred_280_mir-56;coords=sgr04:12773291-12773311

sgr04 nfu_mirna_prediction pre_miRNA 13388714 13388794 . - . ID=nfu_pred_281;biotype=pre-miRNA;gene=mir-10;geneid=nfu_pred_281_mir-10;coords=sgr04:13388714-13388794

sgr04 nfu_mirna_prediction mature_miRNA 13388762 13388784 . - . ID=nfu_pred_281;biotype=5'-mature-miRNA;gene=mir-10;geneid=nfu_pred_281_mir-10;coords=sgr04:13388762-13388784

sgr04 nfu_mirna_prediction pre_miRNA 14989239 14989336 . + . ID=nfu_pred_283;biotype=pre-miRNA;gene=mir-6720;geneid=nfu_pred_283_mir-6720;coords=sgr04:14989239-14989336

sgr04 nfu_mirna_prediction pre_miRNA 16206430 16206510 . - . ID=nfu_pred_284;biotype=pre-miRNA;gene=mir-103;geneid=nfu_pred_284_mir-103;coords=sgr04:16206430-16206510

sgr04 nfu_mirna_prediction mature_miRNA 16206440 16206462 . - . ID=nfu_pred_284;biotype=3'-mature-miRNA;gene=mir-103;geneid=nfu_pred_284_mir-103;coords=sgr04:16206440-16206462

sgr04 nfu_mirna_prediction pre_miRNA 22520815 22520881 . - . ID=nfu_pred_285;biotype=pre-miRNA;gene=mir-3187;geneid=nfu_pred_285_mir-3187;coords=sgr04:22520815-22520881

sgr04 nfu_mirna_prediction pre_miRNA 23590592 23590654 . + . ID=nfu_pred_286;biotype=pre-miRNA;gene=;geneid=nfu_pred_286_;coords=sgr04:23590592-23590654

sgr04 nfu_mirna_prediction mature_miRNA 25531524 25531545 . + . ID=nfu_pred_287;biotype=5'-mature-miRNA;gene=mir-25;geneid=nfu_pred_287_mir-25;coords=sgr04:25531524-25531545

sgr04 nfu_mirna_prediction mature_miRNA 25531560 25531582 . + . ID=nfu_pred_287;biotype=3'-mature-miRNA;gene=mir-25;geneid=nfu_pred_287_mir-25;coords=sgr04:25531560-25531582

sgr04 nfu_mirna_prediction pre_miRNA 27732224 27732330 . - . ID=nfu_pred_288;biotype=pre-miRNA;gene=;geneid=nfu_pred_288_;coords=sgr04:27732224-27732330

sgr04 nfu_mirna_prediction mature_miRNA 27732288 27732308 . - . ID=nfu_pred_288;biotype=5'-mature-miRNA;gene=;geneid=nfu_pred_288_;coords=sgr04:27732288-27732308

sgr04 nfu_mirna_prediction mature_miRNA 27732248 27732269 . - . ID=nfu_pred_288;biotype=3'-mature-miRNA;gene=;geneid=nfu_pred_288_;coords=sgr04:27732248-27732269

sgr04 nfu_mirna_prediction pre_miRNA 28067209 28067290 . - . ID=nfu_pred_289;biotype=pre-miRNA;gene=mir-124;geneid=nfu_pred_289_mir-124;coords=sgr04:28067209-28067290

sgr04 nfu_mirna_prediction mature_miRNA 28067220 28067241 . - . ID=nfu_pred_289;biotype=3'-mature-miRNA;gene=mir-124;geneid=nfu_pred_289_mir-124;coords=sgr04:28067220-28067241

sgr04 nfu_mirna_prediction pre_miRNA 28404990 28405069 . + . ID=nfu_pred_290;biotype=pre-miRNA;gene=mir-1444;geneid=nfu_pred_290_mir-1444;coords=sgr04:28404990-28405069

sgr04 nfu_mirna_prediction mature_miRNA 28405038 28405058 . + . ID=nfu_pred_290;biotype=3'-mature-miRNA;gene=mir-1444;geneid=nfu_pred_290_mir-1444;coords=sgr04:28405038-28405058

sgr04 nfu_mirna_prediction pre_miRNA 32342989 32343090 . + . ID=nfu_pred_292;biotype=pre-miRNA;gene=mir-203;geneid=nfu_pred_292_mir-203;coords=sgr04:32342989-32343090

sgr04 nfu_mirna_prediction mature_miRNA 32343009 32343030 . + . ID=nfu_pred_292;biotype=5'-mature-miRNA;gene=mir-203;geneid=nfu_pred_292_mir-203;coords=sgr04:32343009-32343030

sgr04 nfu_mirna_prediction mature_miRNA 32343047 32343068 . + . ID=nfu_pred_292;biotype=3'-mature-miRNA;gene=mir-203;geneid=nfu_pred_292_mir-203;coords=sgr04:32343047-32343068

sgr04 nfu_mirna_prediction pre_miRNA 37157342 37157439 . + . ID=nfu_pred_293;biotype=pre-miRNA;gene=mir-6720;geneid=nfu_pred_293_mir-6720;coords=sgr04:37157342-37157439

sgr05 nfu_mirna_prediction pre_miRNA 355339 355436 . + . ID=nfu_pred_294;biotype=pre-miRNA;gene=mir-580;geneid=nfu_pred_294_mir-580;coords=sgr05:355339-355436

sgr05 nfu_mirna_prediction mature_miRNA 355395 355417 . + . ID=nfu_pred_294;biotype=3'-mature-miRNA;gene=mir-580;geneid=nfu_pred_294_mir-580;coords=sgr05:355395-355417

sgr05 nfu_mirna_prediction pre_miRNA 2235950 2236020 . + . ID=nfu_pred_296;biotype=pre-miRNA;gene=mir-158;geneid=nfu_pred_296_mir-158;coords=sgr05:2235950-2236020

sgr05 nfu_mirna_prediction mature_miRNA 2235959 2235979 . + . ID=nfu_pred_296;biotype=5'-mature-miRNA;gene=mir-158;geneid=nfu_pred_296_mir-158;coords=sgr05:2235959-2235979

sgr05 nfu_mirna_prediction mature_miRNA 2235986 2236006 . + . ID=nfu_pred_296;biotype=3'-mature-miRNA;gene=mir-158;geneid=nfu_pred_296_mir-158;coords=sgr05:2235986-2236006

sgr05 nfu_mirna_prediction pre_miRNA 4492434 4492477 . + . ID=nfu_pred_302;biotype=pre-miRNA;gene=mir-7549;geneid=nfu_pred_302_mir-7549;coords=sgr05:4492434-4492477

sgr05 nfu_mirna_prediction pre_miRNA 4710723 4710799 . + . ID=nfu_pred_303;biotype=pre-miRNA;gene=;geneid=nfu_pred_303_;coords=sgr05:4710723-4710799

sgr05 nfu_mirna_prediction mature_miRNA 4710733 4710753 . + . ID=nfu_pred_303;biotype=5'-mature-miRNA;gene=;geneid=nfu_pred_303_;coords=sgr05:4710733-4710753

sgr05 nfu_mirna_prediction mature_miRNA 4710769 4710789 . + . ID=nfu_pred_303;biotype=3'-mature-miRNA;gene=;geneid=nfu_pred_303_;coords=sgr05:4710769-4710789

sgr05 nfu_mirna_prediction pre_miRNA 5480987 5481065 . - . ID=nfu_pred_304;biotype=pre-miRNA;gene=mir-1027;geneid=nfu_pred_304_mir-1027;coords=sgr05:5480987-5481065

sgr05 nfu_mirna_prediction mature_miRNA 5480997 5481017 . - . ID=nfu_pred_304;biotype=5'-mature-miRNA;gene=mir-1027;geneid=nfu_pred_304_mir-1027;coords=sgr05:5480997-5481017

sgr05 nfu_mirna_prediction mature_miRNA 5481034 5481055 . - . ID=nfu_pred_304;biotype=3'-mature-miRNA;gene=mir-1027;geneid=nfu_pred_304_mir-1027;coords=sgr05:5481034-5481055

sgr05 nfu_mirna_prediction pre_miRNA 6107602 6107698 . + . ID=nfu_pred_827;biotype=pre-miRNA;gene=mir-15;geneid=nfu_pred_827_mir-15;coords=sgr05:6107602-6107698

sgr05 nfu_mirna_prediction pre_miRNA 6265267 6265380 . + . ID=nfu_pred_305;biotype=pre-miRNA;gene=mir-142;geneid=nfu_pred_305_mir-142;coords=sgr05:6265267-6265380

sgr05 nfu_mirna_prediction mature_miRNA 6265294 6265314 . + . ID=nfu_pred_305;biotype=5'-mature-miRNA;gene=mir-142;geneid=nfu_pred_305_mir-142;coords=sgr05:6265294-6265314

sgr05 nfu_mirna_prediction mature_miRNA 6265331 6265353 . + . ID=nfu_pred_305;biotype=3'-mature-miRNA;gene=mir-142;geneid=nfu_pred_305_mir-142;coords=sgr05:6265331-6265353

sgr05 nfu_mirna_prediction pre_miRNA 8889086 8889167 . - . ID=nfu_pred_307;biotype=pre-miRNA;gene=mir-16;geneid=nfu_pred_307_mir-16;coords=sgr05:8889086-8889167

sgr05 nfu_mirna_prediction mature_miRNA 8889136 8889152 . - . ID=nfu_pred_307;biotype=5'-mature-miRNA;gene=mir-16;geneid=nfu_pred_307_mir-16;coords=sgr05:8889136-8889152

sgr05 nfu_mirna_prediction pre_miRNA 8889291 8889377 . - . ID=nfu_pred_308;biotype=pre-miRNA;gene=mir-15;geneid=nfu_pred_308_mir-15;coords=sgr05:8889291-8889377

sgr05 nfu_mirna_prediction mature_miRNA 8889342 8889363 . - . ID=nfu_pred_308;biotype=5'-mature-miRNA;gene=mir-15;geneid=nfu_pred_308_mir-15;coords=sgr05:8889342-8889363

sgr05 nfu_mirna_prediction pre_miRNA 14378256 14378350 . + . ID=nfu_pred_310;biotype=pre-miRNA;gene=mir-132;geneid=nfu_pred_310_mir-132;coords=sgr05:14378256-14378350

sgr05 nfu_mirna_prediction mature_miRNA 14378271 14378293 . + . ID=nfu_pred_310;biotype=5'-mature-miRNA;gene=mir-132;geneid=nfu_pred_310_mir-132;coords=sgr05:14378271-14378293

sgr05 nfu_mirna_prediction mature_miRNA 14378317 14378337 . + . ID=nfu_pred_310;biotype=3'-mature-miRNA;gene=mir-132;geneid=nfu_pred_310_mir-132;coords=sgr05:14378317-14378337

sgr05 nfu_mirna_prediction pre_miRNA 14378548 14378648 . + . ID=nfu_pred_311;biotype=pre-miRNA;gene=mir-132;geneid=nfu_pred_311_mir-132;coords=sgr05:14378548-14378648

sgr05 nfu_mirna_prediction mature_miRNA 14378568 14378589 . + . ID=nfu_pred_311;biotype=5'-mature-miRNA;gene=mir-132;geneid=nfu_pred_311_mir-132;coords=sgr05:14378568-14378589

sgr05 nfu_mirna_prediction mature_miRNA 14378609 14378630 . + . ID=nfu_pred_311;biotype=3'-mature-miRNA;gene=mir-132;geneid=nfu_pred_311_mir-132;coords=sgr05:14378609-14378630

sgr05 nfu_mirna_prediction pre_miRNA 14425725 14425781 . + . ID=nfu_pred_312;biotype=pre-miRNA;gene=mir-7641;geneid=nfu_pred_312_mir-7641;coords=sgr05:14425725-14425781

sgr05 nfu_mirna_prediction pre_miRNA 16606086 16606191 . + . ID=nfu_pred_313;biotype=pre-miRNA;gene=;geneid=nfu_pred_313_;coords=sgr05:16606086-16606191

sgr05 nfu_mirna_prediction mature_miRNA 16606146 16606167 . + . ID=nfu_pred_313;biotype=3'-mature-miRNA;gene=;geneid=nfu_pred_313_;coords=sgr05:16606146-16606167

sgr05 nfu_mirna_prediction pre_miRNA 17566427 17566527 . + . ID=nfu_pred_314;biotype=pre-miRNA;gene=mir-187;geneid=nfu_pred_314_mir-187;coords=sgr05:17566427-17566527

sgr05 nfu_mirna_prediction mature_miRNA 17566444 17566465 . + . ID=nfu_pred_314;biotype=5'-mature-miRNA;gene=mir-187;geneid=nfu_pred_314_mir-187;coords=sgr05:17566444-17566465

sgr05 nfu_mirna_prediction mature_miRNA 17566485 17566506 . + . ID=nfu_pred_314;biotype=3'-mature-miRNA;gene=mir-187;geneid=nfu_pred_314_mir-187;coords=sgr05:17566485-17566506

sgr05 nfu_mirna_prediction pre_miRNA 26513387 26513468 . + . ID=nfu_pred_315;biotype=pre-miRNA;gene=mir-194;geneid=nfu_pred_315_mir-194;coords=sgr05:26513387-26513468

sgr05 nfu_mirna_prediction mature_miRNA 26513400 26513421 . + . ID=nfu_pred_315;biotype=5'-mature-miRNA;gene=mir-194;geneid=nfu_pred_315_mir-194;coords=sgr05:26513400-26513421

sgr05 nfu_mirna_prediction mature_miRNA 26513433 26513455 . + . ID=nfu_pred_315;biotype=3'-mature-miRNA;gene=mir-194;geneid=nfu_pred_315_mir-194;coords=sgr05:26513433-26513455

sgr05 nfu_mirna_prediction pre_miRNA 27289476 27289563 . - . ID=nfu_pred_316;biotype=pre-miRNA;gene=mir-22;geneid=nfu_pred_316_mir-22;coords=sgr05:27289476-27289563

sgr05 nfu_mirna_prediction mature_miRNA 27289487 27289508 . - . ID=nfu_pred_316;biotype=3'-mature-miRNA;gene=mir-22;geneid=nfu_pred_316_mir-22;coords=sgr05:27289487-27289508

sgr05 nfu_mirna_prediction pre_miRNA 30181713 30181797 . + . ID=nfu_pred_317;biotype=pre-miRNA;gene=mir-583;geneid=nfu_pred_317_mir-583;coords=sgr05:30181713-30181797

sgr05 nfu_mirna_prediction mature_miRNA 30181729 30181750 . + . ID=nfu_pred_317;biotype=5'-mature-miRNA;gene=mir-583;geneid=nfu_pred_317_mir-583;coords=sgr05:30181729-30181750

sgr05 nfu_mirna_prediction mature_miRNA 30181765 30181785 . + . ID=nfu_pred_317;biotype=3'-mature-miRNA;gene=mir-583;geneid=nfu_pred_317_mir-583;coords=sgr05:30181765-30181785

sgr05 nfu_mirna_prediction pre_miRNA 32268824 32268939 . + . ID=nfu_pred_318;biotype=pre-miRNA;gene=mir-139;geneid=nfu_pred_318_mir-139;coords=sgr05:32268824-32268939

sgr05 nfu_mirna_prediction mature_miRNA 32268845 32268867 . + . ID=nfu_pred_318;biotype=5'-mature-miRNA;gene=mir-139;geneid=nfu_pred_318_mir-139;coords=sgr05:32268845-32268867

sgr05 nfu_mirna_prediction mature_miRNA 32268884 32268905 . + . ID=nfu_pred_318;biotype=3'-mature-miRNA;gene=mir-139;geneid=nfu_pred_318_mir-139;coords=sgr05:32268884-32268905

sgr05 nfu_mirna_prediction pre_miRNA 32491064 32491142 . - . ID=nfu_pred_319;biotype=pre-miRNA;gene=;geneid=nfu_pred_319_;coords=sgr05:32491064-32491142

sgr05 nfu_mirna_prediction mature_miRNA 32491111 32491132 . - . ID=nfu_pred_319;biotype=5'-mature-miRNA;gene=;geneid=nfu_pred_319_;coords=sgr05:32491111-32491132

sgr05 nfu_mirna_prediction mature_miRNA 32491075 32491097 . - . ID=nfu_pred_319;biotype=3'-mature-miRNA;gene=;geneid=nfu_pred_319_;coords=sgr05:32491075-32491097

sgr05 nfu_mirna_prediction pre_miRNA 36356590 36356686 . - . ID=nfu_pred_320;biotype=pre-miRNA;gene=mir-132;geneid=nfu_pred_320_mir-132;coords=sgr05:36356590-36356686

sgr05 nfu_mirna_prediction pre_miRNA 37377235 37377317 . - . ID=nfu_pred_321;biotype=pre-miRNA;gene=mir-21;geneid=nfu_pred_321_mir-21;coords=sgr05:37377235-37377317

sgr05 nfu_mirna_prediction mature_miRNA 37377245 37377267 . - . ID=nfu_pred_321;biotype=5'-mature-miRNA;gene=mir-21;geneid=nfu_pred_321_mir-21;coords=sgr05:37377245-37377267

sgr05 nfu_mirna_prediction mature_miRNA 37377284 37377306 . - . ID=nfu_pred_321;biotype=3'-mature-miRNA;gene=mir-21;geneid=nfu_pred_321_mir-21;coords=sgr05:37377284-37377306

sgr05 nfu_mirna_prediction pre_miRNA 39312111 39312191 . - . ID=nfu_pred_322;biotype=pre-miRNA;gene=mir-396;geneid=nfu_pred_322_mir-396;coords=sgr05:39312111-39312191

sgr05 nfu_mirna_prediction mature_miRNA 39312166 39312187 . - . ID=nfu_pred_322;biotype=5'-mature-miRNA;gene=mir-396;geneid=nfu_pred_322_mir-396;coords=sgr05:39312166-39312187

sgr05 nfu_mirna_prediction mature_miRNA 39312124 39312145 . - . ID=nfu_pred_322;biotype=3'-mature-miRNA;gene=mir-396;geneid=nfu_pred_322_mir-396;coords=sgr05:39312124-39312145

sgr05 nfu_mirna_prediction pre_miRNA 44211808 44211886 . + . ID=nfu_pred_323;biotype=pre-miRNA;gene=mir-10;geneid=nfu_pred_323_mir-10;coords=sgr05:44211808-44211886

sgr05 nfu_mirna_prediction mature_miRNA 44211818 44211839 . + . ID=nfu_pred_323;biotype=5'-mature-miRNA;gene=mir-10;geneid=nfu_pred_323_mir-10;coords=sgr05:44211818-44211839

sgr05 nfu_mirna_prediction mature_miRNA 44211852 44211874 . + . ID=nfu_pred_323;biotype=3'-mature-miRNA;gene=mir-10;geneid=nfu_pred_323_mir-10;coords=sgr05:44211852-44211874

sgr05 nfu_mirna_prediction pre_miRNA 44213383 44213476 . + . ID=nfu_pred_324;biotype=pre-miRNA;gene=let-7;geneid=nfu_pred_324_let-7;coords=sgr05:44213383-44213476

sgr05 nfu_mirna_prediction mature_miRNA 44213401 44213422 . + . ID=nfu_pred_324;biotype=5'-mature-miRNA;gene=let-7;geneid=nfu_pred_324_let-7;coords=sgr05:44213401-44213422

sgr05 nfu_mirna_prediction mature_miRNA 44213445 44213466 . + . ID=nfu_pred_324;biotype=3'-mature-miRNA;gene=let-7;geneid=nfu_pred_324_let-7;coords=sgr05:44213445-44213466

sgr05 nfu_mirna_prediction pre_miRNA 44218927 44219003 . + . ID=nfu_pred_325;biotype=pre-miRNA;gene=lin-4;geneid=nfu_pred_325_lin-4;coords=sgr05:44218927-44219003

sgr05 nfu_mirna_prediction mature_miRNA 44218937 44218958 . + . ID=nfu_pred_325;biotype=5'-mature-miRNA;gene=lin-4;geneid=nfu_pred_325_lin-4;coords=sgr05:44218937-44218958

sgr05 nfu_mirna_prediction pre_miRNA 46897704 46897760 . + . ID=nfu_pred_326;biotype=pre-miRNA;gene=mir-7641;geneid=nfu_pred_326_mir-7641;coords=sgr05:46897704-46897760

sgr05 nfu_mirna_prediction pre_miRNA 47747945 47748038 . + . ID=nfu_pred_327;biotype=pre-miRNA;gene=mir-158;geneid=nfu_pred_327_mir-158;coords=sgr05:47747945-47748038

sgr05 nfu_mirna_prediction mature_miRNA 47747959 47747981 . + . ID=nfu_pred_327;biotype=5'-mature-miRNA;gene=mir-158;geneid=nfu_pred_327_mir-158;coords=sgr05:47747959-47747981

sgr05 nfu_mirna_prediction mature_miRNA 47747997 47748018 . + . ID=nfu_pred_327;biotype=3'-mature-miRNA;gene=mir-158;geneid=nfu_pred_327_mir-158;coords=sgr05:47747997-47748018

sgr05 nfu_mirna_prediction pre_miRNA 49045148 49045238 . - . ID=nfu_pred_328;biotype=pre-miRNA;gene=mir-786;geneid=nfu_pred_328_mir-786;coords=sgr05:49045148-49045238

sgr05 nfu_mirna_prediction mature_miRNA 49045203 49045223 . - . ID=nfu_pred_328;biotype=5'-mature-miRNA;gene=mir-786;geneid=nfu_pred_328_mir-786;coords=sgr05:49045203-49045223

sgr05 nfu_mirna_prediction mature_miRNA 49045159 49045181 . - . ID=nfu_pred_328;biotype=3'-mature-miRNA;gene=mir-786;geneid=nfu_pred_328_mir-786;coords=sgr05:49045159-49045181

sgr05 nfu_mirna_prediction pre_miRNA 50124580 50124717 . + . ID=nfu_pred_827;biotype=pre-miRNA;gene=mir-219;geneid=nfu_pred_827_mir-219;coords=sgr05:50124580-50124717

sgr05 nfu_mirna_prediction pre_miRNA 53630713 53630791 . - . ID=nfu_pred_329;biotype=pre-miRNA;gene=mir-101;geneid=nfu_pred_329_mir-101;coords=sgr05:53630713-53630791

sgr05 nfu_mirna_prediction mature_miRNA 53630723 53630744 . - . ID=nfu_pred_329;biotype=3'-mature-miRNA;gene=mir-101;geneid=nfu_pred_329_mir-101;coords=sgr05:53630723-53630744

sgr05 nfu_mirna_prediction pre_miRNA 54839677 54839755 . - . ID=nfu_pred_330;biotype=pre-miRNA;gene=mir-939;geneid=nfu_pred_330_mir-939;coords=sgr05:54839677-54839755

sgr05 nfu_mirna_prediction mature_miRNA 54839697 54839718 . - . ID=nfu_pred_330;biotype=5'-mature-miRNA;gene=mir-939;geneid=nfu_pred_330_mir-939;coords=sgr05:54839697-54839718

sgr05 nfu_mirna_prediction mature_miRNA 54839726 54839747 . - . ID=nfu_pred_330;biotype=3'-mature-miRNA;gene=mir-939;geneid=nfu_pred_330_mir-939;coords=sgr05:54839726-54839747

sgr05 nfu_mirna_prediction pre_miRNA 55923120 55923199 . + . ID=nfu_pred_331;biotype=pre-miRNA;gene=mir-221;geneid=nfu_pred_331_mir-221;coords=sgr05:55923120-55923199

sgr05 nfu_mirna_prediction mature_miRNA 55923130 55923151 . + . ID=nfu_pred_331;biotype=5'-mature-miRNA;gene=mir-221;geneid=nfu_pred_331_mir-221;coords=sgr05:55923130-55923151

sgr05 nfu_mirna_prediction mature_miRNA 55923168 55923190 . + . ID=nfu_pred_331;biotype=3'-mature-miRNA;gene=mir-221;geneid=nfu_pred_331_mir-221;coords=sgr05:55923168-55923190

sgr05 nfu_mirna_prediction pre_miRNA 55923262 55923342 . + . ID=nfu_pred_332;biotype=pre-miRNA;gene=mir-221;geneid=nfu_pred_332_mir-221/mir-222;coords=sgr05:55923262-55923342

sgr05 nfu_mirna_prediction mature_miRNA 55923262 55923292 . + . ID=nfu_pred_332;biotype=5'-mature-miRNA;gene=mir-221;geneid=nfu_pred_332_mir-221;coords=sgr05:55923262-55923292

sgr05 nfu_mirna_prediction mature_miRNA 55923310 55923332 . + . ID=nfu_pred_332;biotype=3'-mature-miRNA;gene=mir-221;geneid=nfu_pred_332_mir-221;coords=sgr05:55923310-55923332

sgr05 nfu_mirna_prediction pre_miRNA 56352606 56352712 . - . ID=nfu_pred_333;biotype=pre-miRNA;gene=mir-143;geneid=nfu_pred_333_mir-143;coords=sgr05:56352606-56352712

sgr05 nfu_mirna_prediction mature_miRNA 56352663 56352685 . - . ID=nfu_pred_333;biotype=5'-mature-miRNA;gene=mir-143;geneid=nfu_pred_333_mir-143;coords=sgr05:56352663-56352685

sgr05 nfu_mirna_prediction mature_miRNA 56352629 56352650 . - . ID=nfu_pred_333;biotype=3'-mature-miRNA;gene=mir-143;geneid=nfu_pred_333_mir-143;coords=sgr05:56352629-56352650

sgr05 nfu_mirna_prediction pre_miRNA 56934597 56934691 . + . ID=nfu_pred_829;biotype=pre-miRNA;gene=mir-133;geneid=nfu_pred_829_mir-133;coords=sgr05:56934597-56934691

sgr05 nfu_mirna_prediction pre_miRNA 59120487 59120587 . - . ID=nfu_pred_334;biotype=pre-miRNA;gene=mir-205;geneid=nfu_pred_334_mir-205;coords=sgr05:59120487-59120587

sgr05 nfu_mirna_prediction mature_miRNA 59120538 59120560 . - . ID=nfu_pred_334;biotype=5'-mature-miRNA;gene=mir-205;geneid=nfu_pred_334_mir-205;coords=sgr05:59120538-59120560

sgr05 nfu_mirna_prediction pre_miRNA 63189345 63189434 . - . ID=nfu_pred_335;biotype=pre-miRNA;gene=mir-128;geneid=nfu_pred_335_mir-128;coords=sgr05:63189345-63189434

sgr05 nfu_mirna_prediction mature_miRNA 63189399 63189420 . - . ID=nfu_pred_335;biotype=5'-mature-miRNA;gene=mir-128;geneid=nfu_pred_335_mir-128;coords=sgr05:63189399-63189420

sgr05 nfu_mirna_prediction mature_miRNA 63189363 63189383 . - . ID=nfu_pred_335;biotype=3'-mature-miRNA;gene=mir-128;geneid=nfu_pred_335_mir-128;coords=sgr05:63189363-63189383

sgr05 nfu_mirna_prediction pre_miRNA 65760833 65760912 . + . ID=nfu_pred_336;biotype=pre-miRNA;gene=mir-326;geneid=nfu_pred_336_mir-326;coords=sgr05:65760833-65760912

sgr05 nfu_mirna_prediction mature_miRNA 65760844 65760865 . + . ID=nfu_pred_336;biotype=5'-mature-miRNA;gene=mir-326;geneid=nfu_pred_336_mir-326;coords=sgr05:65760844-65760865

sgr05 nfu_mirna_prediction mature_miRNA 65760880 65760901 . + . ID=nfu_pred_336;biotype=3'-mature-miRNA;gene=mir-326;geneid=nfu_pred_336_mir-326;coords=sgr05:65760880-65760901

sgr05 nfu_mirna_prediction pre_miRNA 67041765 67041856 . + . ID=nfu_pred_815;biotype=pre-miRNA;gene=mir-196;geneid=nfu_pred_815_mir-196;coords=sgr05:67041765-67041856

sgr06 nfu_mirna_prediction pre_miRNA 2170805 2170925 . + . ID=nfu_pred_337;biotype=pre-miRNA;gene=mir-8915;geneid=nfu_pred_337_mir-8915;coords=sgr06:2170805-2170925

sgr06 nfu_mirna_prediction pre_miRNA 2187009 2187129 . + . ID=nfu_pred_339;biotype=pre-miRNA;gene=mir-8915;geneid=nfu_pred_339_mir-8915;coords=sgr06:2187009-2187129

sgr06 nfu_mirna_prediction pre_miRNA 2277902 2278005 . + . ID=nfu_pred_830;biotype=pre-miRNA;gene=mir-375;geneid=nfu_pred_830_mir-375;coords=sgr06:2277902-2278005

sgr06 nfu_mirna_prediction pre_miRNA 3015959 3016079 . + . ID=nfu_pred_341;biotype=pre-miRNA;gene=mir-8915;geneid=nfu_pred_341_mir-8915;coords=sgr06:3015959-3016079

sgr06 nfu_mirna_prediction pre_miRNA 6577207 6577350 . - . ID=nfu_pred_342;biotype=pre-miRNA;gene=mir-8994;geneid=nfu_pred_342_mir-8994;coords=sgr06:6577207-6577350

sgr06 nfu_mirna_prediction pre_miRNA 12320079 12320150 . + . ID=nfu_pred_343;biotype=pre-miRNA;gene=mir-722;geneid=nfu_pred_343_mir-722;coords=sgr06:12320079-12320150

sgr06 nfu_mirna_prediction pre_miRNA 13862367 13862464 . + . ID=nfu_pred_344;biotype=pre-miRNA;gene=mir-580;geneid=nfu_pred_344_mir-580;coords=sgr06:13862367-13862464

sgr06 nfu_mirna_prediction pre_miRNA 15446218 15446316 . + . ID=nfu_pred_345;biotype=pre-miRNA;gene=mir-6720;geneid=nfu_pred_345_mir-6720;coords=sgr06:15446218-15446316

sgr06 nfu_mirna_prediction pre_miRNA 17302747 17302789 . + . ID=nfu_pred_346;biotype=pre-miRNA;gene=mir-430;geneid=nfu_pred_346_mir-430;coords=sgr06:17302747-17302789

sgr06 nfu_mirna_prediction pre_miRNA 24599878 24599956 . + . ID=nfu_pred_347;biotype=pre-miRNA;gene=;geneid=nfu_pred_347_;coords=sgr06:24599878-24599956

sgr06 nfu_mirna_prediction pre_miRNA 24705088 24705179 . + . ID=nfu_pred_348;biotype=pre-miRNA;gene=mir-821;geneid=nfu_pred_348_mir-821;coords=sgr06:24705088-24705179

sgr06 nfu_mirna_prediction pre_miRNA 28007106 28007188 . - . ID=nfu_pred_349;biotype=pre-miRNA;gene=mir-46;geneid=nfu_pred_349_mir-46;coords=sgr06:28007106-28007188

sgr06 nfu_mirna_prediction mature_miRNA 28007152 28007174 . - . ID=nfu_pred_349;biotype=5'-mature-miRNA;gene=mir-46;geneid=nfu_pred_349_mir-46;coords=sgr06:28007152-28007174

sgr06 nfu_mirna_prediction mature_miRNA 28007116 28007139 . - . ID=nfu_pred_349;biotype=3'-mature-miRNA;gene=mir-46;geneid=nfu_pred_349_mir-46;coords=sgr06:28007116-28007139

sgr06 nfu_mirna_prediction pre_miRNA 29317259 29317376 . + . ID=nfu_pred_350;biotype=pre-miRNA;gene=mir-459;geneid=nfu_pred_350_mir-459;coords=sgr06:29317259-29317376

sgr06 nfu_mirna_prediction pre_miRNA 29552088 29552172 . - . ID=nfu_pred_351;biotype=pre-miRNA;gene=;geneid=nfu_pred_351_;coords=sgr06:29552088-29552172

sgr06 nfu_mirna_prediction mature_miRNA 29552092 29552112 . - . ID=nfu_pred_351;biotype=3'-mature-miRNA;gene=;geneid=nfu_pred_351_;coords=sgr06:29552092-29552112

sgr06 nfu_mirna_prediction pre_miRNA 32169549 32169641 . + . ID=nfu_pred_352;biotype=pre-miRNA;gene=mir-609;geneid=nfu_pred_352_mir-609;coords=sgr06:32169549-32169641

sgr06 nfu_mirna_prediction mature_miRNA 32169565 32169587 . + . ID=nfu_pred_352;biotype=5'-mature-miRNA;gene=mir-609;geneid=nfu_pred_352_mir-609;coords=sgr06:32169565-32169587

sgr06 nfu_mirna_prediction mature_miRNA 32169610 32169632 . + . ID=nfu_pred_352;biotype=3'-mature-miRNA;gene=mir-609;geneid=nfu_pred_352_mir-609;coords=sgr06:32169610-32169632

sgr06 nfu_mirna_prediction pre_miRNA 32969031 32969122 . + . ID=nfu_pred_353;biotype=pre-miRNA;gene=mir-821;geneid=nfu_pred_353_mir-821;coords=sgr06:32969031-32969122

sgr06 nfu_mirna_prediction mature_miRNA 32969046 32969066 . + . ID=nfu_pred_353;biotype=5'-mature-miRNA;gene=mir-821;geneid=nfu_pred_353_mir-821;coords=sgr06:32969046-32969066

sgr06 nfu_mirna_prediction mature_miRNA 32969091 32969111 . + . ID=nfu_pred_353;biotype=3'-mature-miRNA;gene=mir-821;geneid=nfu_pred_353_mir-821;coords=sgr06:32969091-32969111

sgr06 nfu_mirna_prediction pre_miRNA 37672992 37673097 . - . ID=nfu_pred_354;biotype=pre-miRNA;gene=mir-122a/mir-3591;geneid=nfu_pred_354_mir-122a/mir-3591;coords=sgr06:37672992-37673097

sgr06 nfu_mirna_prediction pre_miRNA 48112752 48112853 . - . ID=nfu_pred_356;biotype=pre-miRNA;gene=mir-101;geneid=nfu_pred_356_mir-101;coords=sgr06:48112752-48112853

sgr06 nfu_mirna_prediction mature_miRNA 48112808 48112830 . - . ID=nfu_pred_356;biotype=5'-mature-miRNA;gene=mir-101;geneid=nfu_pred_356_mir-101;coords=sgr06:48112808-48112830

sgr06 nfu_mirna_prediction mature_miRNA 48112773 48112795 . - . ID=nfu_pred_356;biotype=3'-mature-miRNA;gene=mir-101;geneid=nfu_pred_356_mir-101;coords=sgr06:48112773-48112795

sgr06 nfu_mirna_prediction pre_miRNA 48705661 48705741 . + . ID=nfu_pred_357;biotype=pre-miRNA;gene=mir-219;geneid=nfu_pred_357_mir-219;coords=sgr06:48705661-48705741

sgr06 nfu_mirna_prediction mature_miRNA 48705669 48705690 . + . ID=nfu_pred_357;biotype=5'-mature-miRNA;gene=mir-219;geneid=nfu_pred_357_mir-219;coords=sgr06:48705669-48705690

sgr06 nfu_mirna_prediction mature_miRNA 48705715 48705736 . + . ID=nfu_pred_357;biotype=3'-mature-miRNA;gene=mir-219;geneid=nfu_pred_357_mir-219;coords=sgr06:48705715-48705736

sgr06 nfu_mirna_prediction pre_miRNA 50420262 50420340 . + . ID=nfu_pred_358;biotype=pre-miRNA;gene=mir-130;geneid=nfu_pred_358_mir-130;coords=sgr06:50420262-50420340

sgr06 nfu_mirna_prediction mature_miRNA 50420309 50420330 . + . ID=nfu_pred_358;biotype=3'-mature-miRNA;gene=mir-130;geneid=nfu_pred_358_mir-130;coords=sgr06:50420309-50420330

sgr06 nfu_mirna_prediction pre_miRNA 50420539 50420623 . + . ID=nfu_pred_359;biotype=pre-miRNA;gene=mir-301;geneid=nfu_pred_359_mir-301;coords=sgr06:50420539-50420623

sgr06 nfu_mirna_prediction mature_miRNA 50420550 50420571 . + . ID=nfu_pred_359;biotype=5'-mature-miRNA;gene=mir-301;geneid=nfu_pred_359_mir-301;coords=sgr06:50420550-50420571

sgr06 nfu_mirna_prediction mature_miRNA 50420588 50420610 . + . ID=nfu_pred_359;biotype=3'-mature-miRNA;gene=mir-301;geneid=nfu_pred_359_mir-301;coords=sgr06:50420588-50420610

sgr06 nfu_mirna_prediction pre_miRNA 50524286 50524379 . + . ID=nfu_pred_360;biotype=pre-miRNA;gene=mir-218;geneid=nfu_pred_360_mir-218;coords=sgr06:50524286-50524379

sgr06 nfu_mirna_prediction mature_miRNA 50524299 50524319 . + . ID=nfu_pred_360;biotype=5'-mature-miRNA;gene=mir-218;geneid=nfu_pred_360_mir-218;coords=sgr06:50524299-50524319

sgr06 nfu_mirna_prediction pre_miRNA 55224639 55224738 . - . ID=nfu_pred_361;biotype=pre-miRNA;gene=mir-199;geneid=nfu_pred_361_mir-199;coords=sgr06:55224639-55224738

sgr06 nfu_mirna_prediction mature_miRNA 55224696 55224718 . - . ID=nfu_pred_361;biotype=5'-mature-miRNA;gene=mir-199;geneid=nfu_pred_361_mir-199;coords=sgr06:55224696-55224718

sgr06 nfu_mirna_prediction mature_miRNA 55224659 55224680 . - . ID=nfu_pred_361;biotype=3'-mature-miRNA;gene=mir-199;geneid=nfu_pred_361_mir-199;coords=sgr06:55224659-55224680

sgr06 nfu_mirna_prediction pre_miRNA 55269621 55269706 . - . ID=nfu_pred_362;biotype=pre-miRNA;gene=mir-592;geneid=nfu_pred_362_mir-592;coords=sgr06:55269621-55269706

sgr06 nfu_mirna_prediction mature_miRNA 55269674 55269695 . - . ID=nfu_pred_362;biotype=5'-mature-miRNA;gene=mir-592;geneid=nfu_pred_362_mir-592;coords=sgr06:55269674-55269695

sgr06 nfu_mirna_prediction pre_miRNA 59721245 59721328 . + . ID=nfu_pred_363;biotype=pre-miRNA;gene=mir-122;geneid=nfu_pred_363_mir-122;coords=sgr06:59721245-59721328

sgr06 nfu_mirna_prediction mature_miRNA 59721258 59721280 . + . ID=nfu_pred_363;biotype=5'-mature-miRNA;gene=mir-122;geneid=nfu_pred_363_mir-122;coords=sgr06:59721258-59721280

sgr06 nfu_mirna_prediction mature_miRNA 59721290 59721310 . + . ID=nfu_pred_363;biotype=3'-mature-miRNA;gene=mir-122;geneid=nfu_pred_363_mir-122;coords=sgr06:59721290-59721310

sgr06 nfu_mirna_prediction pre_miRNA 62526550 62526650 . + . ID=nfu_pred_364;biotype=pre-miRNA;gene=mir-455;geneid=nfu_pred_364_mir-455;coords=sgr06:62526550-62526650

sgr06 nfu_mirna_prediction mature_miRNA 62526572 62526593 . + . ID=nfu_pred_364;biotype=5'-mature-miRNA;gene=mir-455;geneid=nfu_pred_364_mir-455;coords=sgr06:62526572-62526593

sgr06 nfu_mirna_prediction mature_miRNA 62526609 62526630 . + . ID=nfu_pred_364;biotype=3'-mature-miRNA;gene=mir-455;geneid=nfu_pred_364_mir-455;coords=sgr06:62526609-62526630

sgr06 nfu_mirna_prediction pre_miRNA 64167078 64167177 . + . ID=nfu_pred_366;biotype=pre-miRNA;gene=mir-576;geneid=nfu_pred_366_mir-576;coords=sgr06:64167078-64167177

sgr06 nfu_mirna_prediction mature_miRNA 64167094 64167125 . + . ID=nfu_pred_366;biotype=5'-mature-miRNA;gene=mir-576;geneid=nfu_pred_366_mir-576;coords=sgr06:64167094-64167125

sgr06 nfu_mirna_prediction mature_miRNA 64167136 64167158 . + . ID=nfu_pred_366;biotype=3'-mature-miRNA;gene=mir-576;geneid=nfu_pred_366_mir-576;coords=sgr06:64167136-64167158

sgr06 nfu_mirna_prediction pre_miRNA 64728450 64728553 . + . ID=nfu_pred_367;biotype=pre-miRNA;gene=mir-811;geneid=nfu_pred_367_mir-811;coords=sgr06:64728450-64728553

sgr06 nfu_mirna_prediction mature_miRNA 64728510 64728531 . + . ID=nfu_pred_367;biotype=3'-mature-miRNA;gene=mir-811;geneid=nfu_pred_367_mir-811;coords=sgr06:64728510-64728531

sgr06 nfu_mirna_prediction pre_miRNA 68644857 68644962 . + . ID=nfu_pred_368;biotype=pre-miRNA;gene=;geneid=nfu_pred_368_;coords=sgr06:68644857-68644962

sgr06 nfu_mirna_prediction mature_miRNA 68644874 68644896 . + . ID=nfu_pred_368;biotype=5'-mature-miRNA;gene=;geneid=nfu_pred_368_;coords=sgr06:68644874-68644896

sgr06 nfu_mirna_prediction mature_miRNA 68644917 68644938 . + . ID=nfu_pred_368;biotype=3'-mature-miRNA;gene=;geneid=nfu_pred_368_;coords=sgr06:68644917-68644938

sgr06 nfu_mirna_prediction pre_miRNA 68651841 68651925 . + . ID=nfu_pred_369;biotype=pre-miRNA;gene=;geneid=nfu_pred_369_;coords=sgr06:68651841-68651925

sgr06 nfu_mirna_prediction pre_miRNA 69298198 69298303 . - . ID=nfu_pred_370;biotype=pre-miRNA;gene=mir-214/mir-3120;geneid=nfu_pred_370_mir-214/mir-3120;coords=sgr06:69298198-69298303

sgr06 nfu_mirna_prediction pre_miRNA 69299769 69299866 . - . ID=nfu_pred_371;biotype=pre-miRNA;gene=mir-6720;geneid=nfu_pred_371_mir-6720;coords=sgr06:69299769-69299866

sgr06 nfu_mirna_prediction pre_miRNA 72693275 72693378 . - . ID=nfu_pred_372;biotype=pre-miRNA;gene=mir-126;geneid=nfu_pred_372_mir-126;coords=sgr06:72693275-72693378

sgr06 nfu_mirna_prediction mature_miRNA 72693338 72693358 . - . ID=nfu_pred_372;biotype=5'-mature-miRNA;gene=mir-126;geneid=nfu_pred_372_mir-126;coords=sgr06:72693338-72693358

sgr06 nfu_mirna_prediction mature_miRNA 72693300 72693321 . - . ID=nfu_pred_372;biotype=3'-mature-miRNA;gene=mir-126;geneid=nfu_pred_372_mir-126;coords=sgr06:72693300-72693321

sgr06 nfu_mirna_prediction pre_miRNA 72716638 72716743 . - . ID=nfu_pred_373;biotype=pre-miRNA;gene=;geneid=nfu_pred_373_;coords=sgr06:72716638-72716743

sgr06 nfu_mirna_prediction mature_miRNA 72716663 72716684 . - . ID=nfu_pred_373;biotype=3'-mature-miRNA;gene=;geneid=nfu_pred_373_;coords=sgr06:72716663-72716684

sgr06 nfu_mirna_prediction pre_miRNA 73388299 73388397 . - . ID=nfu_pred_374;biotype=pre-miRNA;gene=mir-807;geneid=nfu_pred_374_mir-807;coords=sgr06:73388299-73388397

sgr06 nfu_mirna_prediction mature_miRNA 73388356 73388377 . - . ID=nfu_pred_374;biotype=5'-mature-miRNA;gene=mir-807;geneid=nfu_pred_374_mir-807;coords=sgr06:73388356-73388377

sgr06 nfu_mirna_prediction pre_miRNA 73919137 73919231 . - . ID=nfu_pred_375;biotype=pre-miRNA;gene=mir-607;geneid=nfu_pred_375_mir-607;coords=sgr06:73919137-73919231

sgr06 nfu_mirna_prediction mature_miRNA 73919191 73919213 . - . ID=nfu_pred_375;biotype=5'-mature-miRNA;gene=mir-607;geneid=nfu_pred_375_mir-607;coords=sgr06:73919191-73919213

sgr06 nfu_mirna_prediction mature_miRNA 73919151 73919173 . - . ID=nfu_pred_375;biotype=3'-mature-miRNA;gene=mir-607;geneid=nfu_pred_375_mir-607;coords=sgr06:73919151-73919173

sgr06 nfu_mirna_prediction pre_miRNA 73920179 73920255 . + . ID=nfu_pred_376;biotype=pre-miRNA;gene=mir-197;geneid=nfu_pred_376_mir-197;coords=sgr06:73920179-73920255

sgr06 nfu_mirna_prediction mature_miRNA 73920186 73920207 . + . ID=nfu_pred_376;biotype=5'-mature-miRNA;gene=mir-197;geneid=nfu_pred_376_mir-197;coords=sgr06:73920186-73920207

sgr06 nfu_mirna_prediction mature_miRNA 73920225 73920246 . + . ID=nfu_pred_376;biotype=3'-mature-miRNA;gene=mir-197;geneid=nfu_pred_376_mir-197;coords=sgr06:73920225-73920246

sgr06 nfu_mirna_prediction pre_miRNA 74142319 74142402 . + . ID=nfu_pred_377;biotype=pre-miRNA;gene=mir-460;geneid=nfu_pred_377_mir-460;coords=sgr06:74142319-74142402

sgr06 nfu_mirna_prediction pre_miRNA 74142599 74142682 . + . ID=nfu_pred_378;biotype=pre-miRNA;gene=mir-460;geneid=nfu_pred_378_mir-460;coords=sgr06:74142599-74142682

sgr06 nfu_mirna_prediction pre_miRNA 74360820 74360900 . + . ID=nfu_pred_379;biotype=pre-miRNA;gene=mir-1027;geneid=nfu_pred_379_mir-1027;coords=sgr06:74360820-74360900

sgr06 nfu_mirna_prediction mature_miRNA 74360834 74360855 . + . ID=nfu_pred_379;biotype=5'-mature-miRNA;gene=mir-1027;geneid=nfu_pred_379_mir-1027;coords=sgr06:74360834-74360855

sgr06 nfu_mirna_prediction pre_miRNA 78787253 78787333 . + . ID=nfu_pred_380;biotype=pre-miRNA;gene=mir-631;geneid=nfu_pred_380_mir-631;coords=sgr06:78787253-78787333

sgr06 nfu_mirna_prediction mature_miRNA 78787263 78787284 . + . ID=nfu_pred_380;biotype=5'-mature-miRNA;gene=mir-631;geneid=nfu_pred_380_mir-631;coords=sgr06:78787263-78787284

sgr06 nfu_mirna_prediction mature_miRNA 78787296 78787318 . + . ID=nfu_pred_380;biotype=3'-mature-miRNA;gene=mir-631;geneid=nfu_pred_380_mir-631;coords=sgr06:78787296-78787318

sgr06 nfu_mirna_prediction pre_miRNA 80266627 80266718 . - . ID=nfu_pred_381;biotype=pre-miRNA;gene=;geneid=nfu_pred_381_;coords=sgr06:80266627-80266718

sgr06 nfu_mirna_prediction pre_miRNA 82081482 82081624 . + . ID=nfu_pred_382;biotype=pre-miRNA;gene=mir-811;geneid=nfu_pred_382_mir-811;coords=sgr06:82081482-82081624

sgr06 nfu_mirna_prediction mature_miRNA 82081527 82081548 . + . ID=nfu_pred_382;biotype=5'-mature-miRNA;gene=mir-811;geneid=nfu_pred_382_mir-811;coords=sgr06:82081527-82081548

sgr06 nfu_mirna_prediction mature_miRNA 82081564 82081585 . + . ID=nfu_pred_382;biotype=3'-mature-miRNA;gene=mir-811;geneid=nfu_pred_382_mir-811;coords=sgr06:82081564-82081585

sgr06 nfu_mirna_prediction pre_miRNA 82550604 82550688 . + . ID=nfu_pred_383;biotype=pre-miRNA;gene=mir-599;geneid=nfu_pred_383_mir-599;coords=sgr06:82550604-82550688

sgr06 nfu_mirna_prediction mature_miRNA 82550622 82550642 . + . ID=nfu_pred_383;biotype=5'-mature-miRNA;gene=mir-599;geneid=nfu_pred_383_mir-599;coords=sgr06:82550622-82550642

sgr06 nfu_mirna_prediction mature_miRNA 82550656 82550676 . + . ID=nfu_pred_383;biotype=3'-mature-miRNA;gene=mir-599;geneid=nfu_pred_383_mir-599;coords=sgr06:82550656-82550676

sgr06 nfu_mirna_prediction pre_miRNA 86860851 86860931 . - . ID=nfu_pred_384;biotype=pre-miRNA;gene=mir-122;geneid=nfu_pred_384_mir-122;coords=sgr06:86860851-86860931

sgr06 nfu_mirna_prediction mature_miRNA 86860863 86860884 . - . ID=nfu_pred_384;biotype=5'-mature-miRNA;gene=mir-122;geneid=nfu_pred_384_mir-122;coords=sgr06:86860863-86860884

sgr06 nfu_mirna_prediction mature_miRNA 86860899 86860921 . - . ID=nfu_pred_384;biotype=3'-mature-miRNA;gene=mir-122;geneid=nfu_pred_384_mir-122;coords=sgr06:86860899-86860921

sgr07 nfu_mirna_prediction pre_miRNA 522317 522407 . - . ID=nfu_pred_386;biotype=pre-miRNA;gene=mir-672;geneid=nfu_pred_386_mir-672;coords=sgr07:522317-522407

sgr07 nfu_mirna_prediction pre_miRNA 1371923 1372010 . + . ID=nfu_pred_387;biotype=pre-miRNA;gene=mir-187;geneid=nfu_pred_387_mir-187;coords=sgr07:1371923-1372010

sgr07 nfu_mirna_prediction mature_miRNA 1371938 1371958 . + . ID=nfu_pred_387;biotype=5'-mature-miRNA;gene=mir-187;geneid=nfu_pred_387_mir-187;coords=sgr07:1371938-1371958

sgr07 nfu_mirna_prediction mature_miRNA 1371977 1371998 . + . ID=nfu_pred_387;biotype=3'-mature-miRNA;gene=mir-187;geneid=nfu_pred_387_mir-187;coords=sgr07:1371977-1371998

sgr07 nfu_mirna_prediction pre_miRNA 1620127 1620228 . + . ID=nfu_pred_388;biotype=pre-miRNA;gene=mir-728;geneid=nfu_pred_388_mir-728;coords=sgr07:1620127-1620228

sgr07 nfu_mirna_prediction pre_miRNA 1757749 1757827 . - . ID=nfu_pred_389;biotype=pre-miRNA;gene=;geneid=nfu_pred_389_;coords=sgr07:1757749-1757827

sgr07 nfu_mirna_prediction mature_miRNA 1757757 1757777 . - . ID=nfu_pred_389;biotype=3'-mature-miRNA;gene=;geneid=nfu_pred_389_;coords=sgr07:1757757-1757777

sgr07 nfu_mirna_prediction pre_miRNA 2639793 2639868 . - . ID=nfu_pred_390;biotype=pre-miRNA;gene=mir-19;geneid=nfu_pred_390_mir-19;coords=sgr07:2639793-2639868

sgr07 nfu_mirna_prediction mature_miRNA 2639800 2639822 . - . ID=nfu_pred_390;biotype=3'-mature-miRNA;gene=mir-19;geneid=nfu_pred_390_mir-19;coords=sgr07:2639800-2639822

sgr07 nfu_mirna_prediction pre_miRNA 2639897 2639977 . - . ID=nfu_pred_391;biotype=pre-miRNA;gene=mir-17;geneid=nfu_pred_391_mir-17;coords=sgr07:2639897-2639977

sgr07 nfu_mirna_prediction mature_miRNA 2639945 2639966 . - . ID=nfu_pred_391;biotype=5'-mature-miRNA;gene=mir-17;geneid=nfu_pred_391_mir-17;coords=sgr07:2639945-2639966

sgr07 nfu_mirna_prediction mature_miRNA 2639912 2639933 . - . ID=nfu_pred_391;biotype=3'-mature-miRNA;gene=mir-17;geneid=nfu_pred_391_mir-17;coords=sgr07:2639912-2639933

sgr07 nfu_mirna_prediction pre_miRNA 2640188 2640285 . - . ID=nfu_pred_392;biotype=pre-miRNA;gene=mir-186;geneid=nfu_pred_392_mir-186;coords=sgr07:2640188-2640285

sgr07 nfu_mirna_prediction mature_miRNA 2640246 2640266 . - . ID=nfu_pred_392;biotype=5'-mature-miRNA;gene=mir-186;geneid=nfu_pred_392_mir-186;coords=sgr07:2640246-2640266

sgr07 nfu_mirna_prediction mature_miRNA 2640204 2640225 . - . ID=nfu_pred_392;biotype=3'-mature-miRNA;gene=mir-186;geneid=nfu_pred_392_mir-186;coords=sgr07:2640204-2640225

sgr07 nfu_mirna_prediction pre_miRNA 3075400 3075486 . + . ID=nfu_pred_393;biotype=pre-miRNA;gene=mir-581;geneid=nfu_pred_393_mir-581;coords=sgr07:3075400-3075486

sgr07 nfu_mirna_prediction mature_miRNA 3075455 3075476 . + . ID=nfu_pred_393;biotype=3'-mature-miRNA;gene=mir-581;geneid=nfu_pred_393_mir-581;coords=sgr07:3075455-3075476

sgr07 nfu_mirna_prediction pre_miRNA 8795005 8795085 . + . ID=nfu_pred_394;biotype=pre-miRNA;gene=MIR396;geneid=nfu_pred_394_MIR396;coords=sgr07:8795005-8795085

sgr07 nfu_mirna_prediction mature_miRNA 8795009 8795030 . + . ID=nfu_pred_394;biotype=5'-mature-miRNA;gene=MIR396;geneid=nfu_pred_394_MIR396;coords=sgr07:8795009-8795030

sgr07 nfu_mirna_prediction mature_miRNA 8795050 8795071 . + . ID=nfu_pred_394;biotype=3'-mature-miRNA;gene=MIR396;geneid=nfu_pred_394_MIR396;coords=sgr07:8795050-8795071

sgr07 nfu_mirna_prediction pre_miRNA 11407535 11407640 . + . ID=nfu_pred_395;biotype=pre-miRNA;gene=mir-143;geneid=nfu_pred_395_mir-143;coords=sgr07:11407535-11407640

sgr07 nfu_mirna_prediction mature_miRNA 11407561 11407582 . + . ID=nfu_pred_395;biotype=5'-mature-miRNA;gene=mir-143;geneid=nfu_pred_395_mir-143;coords=sgr07:11407561-11407582

sgr07 nfu_mirna_prediction mature_miRNA 11407595 11407615 . + . ID=nfu_pred_395;biotype=3'-mature-miRNA;gene=mir-143;geneid=nfu_pred_395_mir-143;coords=sgr07:11407595-11407615

sgr07 nfu_mirna_prediction pre_miRNA 11407735 11407843 . + . ID=nfu_pred_396;biotype=pre-miRNA;gene=mir-145;geneid=nfu_pred_396_mir-145;coords=sgr07:11407735-11407843

sgr07 nfu_mirna_prediction mature_miRNA 11407764 11407786 . + . ID=nfu_pred_396;biotype=5'-mature-miRNA;gene=mir-145;geneid=nfu_pred_396_mir-145;coords=sgr07:11407764-11407786

sgr07 nfu_mirna_prediction mature_miRNA 11407802 11407823 . + . ID=nfu_pred_396;biotype=3'-mature-miRNA;gene=mir-145;geneid=nfu_pred_396_mir-145;coords=sgr07:11407802-11407823

sgr07 nfu_mirna_prediction pre_miRNA 11846655 11846717 . + . ID=nfu_pred_397;biotype=pre-miRNA;gene=;geneid=nfu_pred_397_;coords=sgr07:11846655-11846717

sgr07 nfu_mirna_prediction pre_miRNA 12892735 12892879 . + . ID=nfu_pred_398;biotype=pre-miRNA;gene=mir-8852;geneid=nfu_pred_398_mir-8852;coords=sgr07:12892735-12892879

sgr07 nfu_mirna_prediction pre_miRNA 13619556 13619697 . + . ID=nfu_pred_399;biotype=pre-miRNA;gene=mir-455;geneid=nfu_pred_399_mir-455;coords=sgr07:13619556-13619697

sgr07 nfu_mirna_prediction pre_miRNA 16105357 16105413 . - . ID=nfu_pred_400;biotype=pre-miRNA;gene=mir-7641;geneid=nfu_pred_400_mir-7641;coords=sgr07:16105357-16105413

sgr07 nfu_mirna_prediction pre_miRNA 19915295 19915377 . - . ID=nfu_pred_401;biotype=pre-miRNA;gene=mir-2985;geneid=nfu_pred_401_mir-2985;coords=sgr07:19915295-19915377

sgr07 nfu_mirna_prediction mature_miRNA 19915305 19915326 . - . ID=nfu_pred_401;biotype=3'-mature-miRNA;gene=mir-2985;geneid=nfu_pred_401_mir-2985;coords=sgr07:19915305-19915326

sgr07 nfu_mirna_prediction pre_miRNA 25921744 25921826 . + . ID=nfu_pred_403;biotype=pre-miRNA;gene=mir-1265;geneid=nfu_pred_403_mir-1265;coords=sgr07:25921744-25921826

sgr07 nfu_mirna_prediction mature_miRNA 25921756 25921778 . + . ID=nfu_pred_403;biotype=5'-mature-miRNA;gene=mir-1265;geneid=nfu_pred_403_mir-1265;coords=sgr07:25921756-25921778

sgr07 nfu_mirna_prediction mature_miRNA 25921792 25921813 . + . ID=nfu_pred_403;biotype=3'-mature-miRNA;gene=mir-1265;geneid=nfu_pred_403_mir-1265;coords=sgr07:25921792-25921813

sgr07 nfu_mirna_prediction pre_miRNA 26940551 26940653 . - . ID=nfu_pred_404;biotype=pre-miRNA;gene=;geneid=nfu_pred_404_;coords=sgr07:26940551-26940653

sgr07 nfu_mirna_prediction mature_miRNA 26940616 26940638 . - . ID=nfu_pred_404;biotype=5'-mature-miRNA;gene=;geneid=nfu_pred_404_;coords=sgr07:26940616-26940638

sgr07 nfu_mirna_prediction mature_miRNA 26940572 26940594 . - . ID=nfu_pred_404;biotype=3'-mature-miRNA;gene=;geneid=nfu_pred_404_;coords=sgr07:26940572-26940594

sgr07 nfu_mirna_prediction pre_miRNA 33516887 33516972 . - . ID=nfu_pred_407;biotype=pre-miRNA;gene=mir-194;geneid=nfu_pred_407_mir-194;coords=sgr07:33516887-33516972

sgr07 nfu_mirna_prediction mature_miRNA 33516935 33516957 . - . ID=nfu_pred_407;biotype=5'-mature-miRNA;gene=mir-194;geneid=nfu_pred_407_mir-194;coords=sgr07:33516935-33516957

sgr07 nfu_mirna_prediction mature_miRNA 33516903 33516924 . - . ID=nfu_pred_407;biotype=3'-mature-miRNA;gene=mir-194;geneid=nfu_pred_407_mir-194;coords=sgr07:33516903-33516924

sgr07 nfu_mirna_prediction pre_miRNA 36125518 36125619 . + . ID=nfu_pred_408;biotype=pre-miRNA;gene=mir-820;geneid=nfu_pred_408_mir-820;coords=sgr07:36125518-36125619

sgr07 nfu_mirna_prediction mature_miRNA 36125537 36125558 . + . ID=nfu_pred_408;biotype=5'-mature-miRNA;gene=mir-820;geneid=nfu_pred_408_mir-820;coords=sgr07:36125537-36125558

sgr07 nfu_mirna_prediction mature_miRNA 36125575 36125596 . + . ID=nfu_pred_408;biotype=3'-mature-miRNA;gene=mir-820;geneid=nfu_pred_408_mir-820;coords=sgr07:36125575-36125596

sgr07 nfu_mirna_prediction pre_miRNA 42467549 42467629 . - . ID=nfu_pred_409;biotype=pre-miRNA;gene=mir-103;geneid=nfu_pred_409_mir-103;coords=sgr07:42467549-42467629

sgr07 nfu_mirna_prediction mature_miRNA 42467559 42467581 . - . ID=nfu_pred_409;biotype=3'-mature-miRNA;gene=mir-103;geneid=nfu_pred_409_mir-103;coords=sgr07:42467559-42467581

sgr07 nfu_mirna_prediction pre_miRNA 43293809 43293887 . - . ID=nfu_pred_410;biotype=pre-miRNA;gene=mir-25;geneid=nfu_pred_410_mir-25;coords=sgr07:43293809-43293887

sgr07 nfu_mirna_prediction mature_miRNA 43293819 43293840 . - . ID=nfu_pred_410;biotype=5'-mature-miRNA;gene=mir-25;geneid=nfu_pred_410_mir-25;coords=sgr07:43293819-43293840

sgr07 nfu_mirna_prediction mature_miRNA 43293856 43293878 . - . ID=nfu_pred_410;biotype=3'-mature-miRNA;gene=mir-25;geneid=nfu_pred_410_mir-25;coords=sgr07:43293856-43293878

sgr07 nfu_mirna_prediction pre_miRNA 43293980 43294058 . - . ID=nfu_pred_411;biotype=pre-miRNA;gene=mir-19;geneid=nfu_pred_411_mir-19;coords=sgr07:43293980-43294058

sgr07 nfu_mirna_prediction mature_miRNA 43293990 43294011 . - . ID=nfu_pred_411;biotype=3'-mature-miRNA;gene=mir-19;geneid=nfu_pred_411_mir-19;coords=sgr07:43293990-43294011

sgr07 nfu_mirna_prediction pre_miRNA 43294200 43294280 . - . ID=nfu_pred_412;biotype=pre-miRNA;gene=mir-17;geneid=nfu_pred_412_mir-17;coords=sgr07:43294200-43294280

sgr07 nfu_mirna_prediction mature_miRNA 43294213 43294234 . - . ID=nfu_pred_412;biotype=5'-mature-miRNA;gene=mir-17;geneid=nfu_pred_412_mir-17;coords=sgr07:43294213-43294234

sgr07 nfu_mirna_prediction mature_miRNA 43294249 43294270 . - . ID=nfu_pred_412;biotype=3'-mature-miRNA;gene=mir-17;geneid=nfu_pred_412_mir-17;coords=sgr07:43294249-43294270

sgr07 nfu_mirna_prediction pre_miRNA 43294334 43294414 . - . ID=nfu_pred_413;biotype=pre-miRNA;gene=mir-17;geneid=nfu_pred_413_mir-17;coords=sgr07:43294334-43294414

sgr07 nfu_mirna_prediction mature_miRNA 43294382 43294404 . - . ID=nfu_pred_413;biotype=5'-mature-miRNA;gene=mir-17;geneid=nfu_pred_413_mir-17;coords=sgr07:43294382-43294404

sgr07 nfu_mirna_prediction pre_miRNA 43945257 43945371 . - . ID=nfu_pred_414;biotype=pre-miRNA;gene=mir-218;geneid=nfu_pred_414_mir-218;coords=sgr07:43945257-43945371

sgr07 nfu_mirna_prediction mature_miRNA 43945322 43945343 . - . ID=nfu_pred_414;biotype=5'-mature-miRNA;gene=mir-218;geneid=nfu_pred_414_mir-218;coords=sgr07:43945322-43945343

sgr07 nfu_mirna_prediction mature_miRNA 43945281 43945300 . - . ID=nfu_pred_414;biotype=3'-mature-miRNA;gene=mir-218;geneid=nfu_pred_414_mir-218;coords=sgr07:43945281-43945300

sgr07 nfu_mirna_prediction pre_miRNA 45562279 45562355 . + . ID=nfu_pred_415;biotype=pre-miRNA;gene=;geneid=nfu_pred_415_;coords=sgr07:45562279-45562355

sgr07 nfu_mirna_prediction pre_miRNA 47824658 47824740 . + . ID=nfu_pred_416;biotype=pre-miRNA;gene=mir-2985;geneid=nfu_pred_416_mir-2985;coords=sgr07:47824658-47824740

sgr07 nfu_mirna_prediction mature_miRNA 47824709 47824729 . + . ID=nfu_pred_416;biotype=3'-mature-miRNA;gene=mir-2985;geneid=nfu_pred_416_mir-2985;coords=sgr07:47824709-47824729

sgr07 nfu_mirna_prediction pre_miRNA 50477957 50478041 . + . ID=nfu_pred_417;biotype=pre-miRNA;gene=mir-599;geneid=nfu_pred_417_mir-599;coords=sgr07:50477957-50478041

sgr07 nfu_mirna_prediction mature_miRNA 50477975 50477995 . + . ID=nfu_pred_417;biotype=5'-mature-miRNA;gene=mir-599;geneid=nfu_pred_417_mir-599;coords=sgr07:50477975-50477995

sgr07 nfu_mirna_prediction mature_miRNA 50478009 50478029 . + . ID=nfu_pred_417;biotype=3'-mature-miRNA;gene=mir-599;geneid=nfu_pred_417_mir-599;coords=sgr07:50478009-50478029

sgr07 nfu_mirna_prediction pre_miRNA 51603761 51603848 . + . ID=nfu_pred_831;biotype=pre-miRNA;gene=mir-30;geneid=nfu_pred_831_mir-30;coords=sgr07:51603761-51603848

sgr07 nfu_mirna_prediction pre_miRNA 53186041 53186175 . + . ID=nfu_pred_418;biotype=pre-miRNA;gene=mir-214/mir-3120;geneid=nfu_pred_418_mir-214/mir-3120;coords=sgr07:53186041-53186175

sgr07 nfu_mirna_prediction pre_miRNA 53992331 53992426 . - . ID=nfu_pred_419;biotype=pre-miRNA;gene=mir-458;geneid=nfu_pred_419_mir-458;coords=sgr07:53992331-53992426

sgr07 nfu_mirna_prediction mature_miRNA 53992349 53992370 . - . ID=nfu_pred_419;biotype=3'-mature-miRNA;gene=mir-458;geneid=nfu_pred_419_mir-458;coords=sgr07:53992349-53992370

sgr07 nfu_mirna_prediction pre_miRNA 54004132 54004234 . - . ID=nfu_pred_420;biotype=pre-miRNA;gene=;geneid=nfu_pred_420_;coords=sgr07:54004132-54004234

sgr07 nfu_mirna_prediction mature_miRNA 54004192 54004212 . - . ID=nfu_pred_420;biotype=5'-mature-miRNA;gene=;geneid=nfu_pred_420_;coords=sgr07:54004192-54004212

sgr07 nfu_mirna_prediction mature_miRNA 54004149 54004171 . - . ID=nfu_pred_420;biotype=3'-mature-miRNA;gene=;geneid=nfu_pred_420_;coords=sgr07:54004149-54004171

sgr07 nfu_mirna_prediction pre_miRNA 58331290 58331384 . - . ID=nfu_pred_816;biotype=pre-miRNA;gene=mir-200;geneid=nfu_pred_816_mir-200;coords=sgr07:58331290-58331384

sgr07 nfu_mirna_prediction pre_miRNA 61407874 61407984 . - . ID=nfu_pred_422;biotype=pre-miRNA;gene=mir-29;geneid=nfu_pred_422_mir-29;coords=sgr07:61407874-61407984

sgr07 nfu_mirna_prediction mature_miRNA 61407938 61407959 . - . ID=nfu_pred_422;biotype=5'-mature-miRNA;gene=mir-29;geneid=nfu_pred_422_mir-29;coords=sgr07:61407938-61407959

sgr07 nfu_mirna_prediction mature_miRNA 61407899 61407920 . - . ID=nfu_pred_422;biotype=3'-mature-miRNA;gene=mir-29;geneid=nfu_pred_422_mir-29;coords=sgr07:61407899-61407920

sgr07 nfu_mirna_prediction pre_miRNA 61408015 61408121 . - . ID=nfu_pred_423;biotype=pre-miRNA;gene=mir-29;geneid=nfu_pred_423_mir-29;coords=sgr07:61408015-61408121

sgr07 nfu_mirna_prediction mature_miRNA 61408039 61408060 . - . ID=nfu_pred_423;biotype=5'-mature-miRNA;gene=mir-29;geneid=nfu_pred_423_mir-29;coords=sgr07:61408039-61408060

sgr07 nfu_mirna_prediction mature_miRNA 61408077 61408098 . - . ID=nfu_pred_423;biotype=3'-mature-miRNA;gene=mir-29;geneid=nfu_pred_423_mir-29;coords=sgr07:61408077-61408098

sgr07 nfu_mirna_prediction pre_miRNA 67382778 67382863 . + . ID=nfu_pred_424;biotype=pre-miRNA;gene=mir-455;geneid=nfu_pred_424_mir-455;coords=sgr07:67382778-67382863

sgr07 nfu_mirna_prediction mature_miRNA 67382828 67382849 . + . ID=nfu_pred_424;biotype=3'-mature-miRNA;gene=mir-455;geneid=nfu_pred_424_mir-455;coords=sgr07:67382828-67382849

sgr07 nfu_mirna_prediction pre_miRNA 70120153 70120273 . + . ID=nfu_pred_425;biotype=pre-miRNA;gene=mir-8915;geneid=nfu_pred_425_mir-8915;coords=sgr07:70120153-70120273

sgr07 nfu_mirna_prediction pre_miRNA 71376960 71377022 . + . ID=nfu_pred_427;biotype=pre-miRNA;gene=;geneid=nfu_pred_427_;coords=sgr07:71376960-71377022

sgr07 nfu_mirna_prediction mature_miRNA 71376962 71376983 . + . ID=nfu_pred_427;biotype=3'-mature-miRNA;gene=;geneid=nfu_pred_427_;coords=sgr07:71376962-71376983

sgr07 nfu_mirna_prediction pre_miRNA 72073967 72074057 . - . ID=nfu_pred_428;biotype=pre-miRNA;gene=let-7;geneid=nfu_pred_428_let-7;coords=sgr07:72073967-72074057

sgr07 nfu_mirna_prediction mature_miRNA 72073970 72073991 . - . ID=nfu_pred_428;biotype=5'-mature-miRNA;gene=let-7;geneid=nfu_pred_428_let-7;coords=sgr07:72073970-72073991

sgr07 nfu_mirna_prediction mature_miRNA 72074030 72074051 . - . ID=nfu_pred_428;biotype=3'-mature-miRNA;gene=let-7;geneid=nfu_pred_428_let-7;coords=sgr07:72074030-72074051

sgr07 nfu_mirna_prediction pre_miRNA 72340755 72340845 . + . ID=nfu_pred_429;biotype=pre-miRNA;gene=let-7;geneid=nfu_pred_429_let-7;coords=sgr07:72340755-72340845

sgr07 nfu_mirna_prediction mature_miRNA 72340762 72340786 . + . ID=nfu_pred_429;biotype=5'-mature-miRNA;gene=let-7;geneid=nfu_pred_429_let-7;coords=sgr07:72340762-72340786

sgr07 nfu_mirna_prediction mature_miRNA 72340817 72340839 . + . ID=nfu_pred_429;biotype=3'-mature-miRNA;gene=let-7;geneid=nfu_pred_429_let-7;coords=sgr07:72340817-72340839

sgr07 nfu_mirna_prediction pre_miRNA 72341585 72341680 . + . ID=nfu_pred_430;biotype=pre-miRNA;gene=let-7;geneid=nfu_pred_430_let-7;coords=sgr07:72341585-72341680

sgr07 nfu_mirna_prediction mature_miRNA 72341596 72341617 . + . ID=nfu_pred_430;biotype=5'-mature-miRNA;gene=let-7;geneid=nfu_pred_430_let-7;coords=sgr07:72341596-72341617

sgr07 nfu_mirna_prediction mature_miRNA 72341640 72341661 . + . ID=nfu_pred_430;biotype=3'-mature-miRNA;gene=let-7;geneid=nfu_pred_430_let-7;coords=sgr07:72341640-72341661

sgr07 nfu_mirna_prediction pre_miRNA 78175591 78175684 . + . ID=nfu_pred_431;biotype=pre-miRNA;gene=mir-135;geneid=nfu_pred_431_mir-135;coords=sgr07:78175591-78175684

sgr07 nfu_mirna_prediction mature_miRNA 78175608 78175629 . + . ID=nfu_pred_431;biotype=5'-mature-miRNA;gene=mir-135;geneid=nfu_pred_431_mir-135;coords=sgr07:78175608-78175629

sgr07 nfu_mirna_prediction mature_miRNA 78175646 78175665 . + . ID=nfu_pred_431;biotype=3'-mature-miRNA;gene=mir-135;geneid=nfu_pred_431_mir-135;coords=sgr07:78175646-78175665

sgr07 nfu_mirna_prediction pre_miRNA 78698835 78698996 . - . ID=nfu_pred_432;biotype=pre-miRNA;gene=mir-811;geneid=nfu_pred_432_mir-811;coords=sgr07:78698835-78698996

sgr07 nfu_mirna_prediction mature_miRNA 78698926 78698947 . - . ID=nfu_pred_432;biotype=5'-mature-miRNA;gene=mir-811;geneid=nfu_pred_432_mir-811;coords=sgr07:78698926-78698947

sgr07 nfu_mirna_prediction mature_miRNA 78698889 78698909 . - . ID=nfu_pred_432;biotype=3'-mature-miRNA;gene=mir-811;geneid=nfu_pred_432_mir-811;coords=sgr07:78698889-78698909

sgr07 nfu_mirna_prediction pre_miRNA 79465544 79465649 . + . ID=nfu_pred_433;biotype=pre-miRNA;gene=mir-183;geneid=nfu_pred_433_mir-183;coords=sgr07:79465544-79465649

sgr07 nfu_mirna_prediction mature_miRNA 79465566 79465587 . + . ID=nfu_pred_433;biotype=5'-mature-miRNA;gene=mir-183;geneid=nfu_pred_433_mir-183;coords=sgr07:79465566-79465587

sgr07 nfu_mirna_prediction pre_miRNA 79465883 79465974 . + . ID=nfu_pred_434;biotype=pre-miRNA;gene=mir-96a;geneid=nfu_pred_434_mir-96a;coords=sgr07:79465883-79465974

sgr07 nfu_mirna_prediction mature_miRNA 79465898 79465920 . + . ID=nfu_pred_434;biotype=5'-mature-miRNA;gene=mir-96a;geneid=nfu_pred_434_mir-96a;coords=sgr07:79465898-79465920

sgr07 nfu_mirna_prediction mature_miRNA 79465938 79465959 . + . ID=nfu_pred_434;biotype=3'-mature-miRNA;gene=mir-96a;geneid=nfu_pred_434_mir-96a;coords=sgr07:79465938-79465959

sgr07 nfu_mirna_prediction pre_miRNA 79466239 79466323 . + . ID=nfu_pred_435;biotype=pre-miRNA;gene=mir-182;geneid=nfu_pred_435_mir-182;coords=sgr07:79466239-79466323

sgr07 nfu_mirna_prediction mature_miRNA 79466249 79466271 . + . ID=nfu_pred_435;biotype=5'-mature-miRNA;gene=mir-182;geneid=nfu_pred_435_mir-182;coords=sgr07:79466249-79466271

sgr07 nfu_mirna_prediction pre_miRNA 82688967 82689061 . + . ID=nfu_pred_436;biotype=pre-miRNA;gene=mir-129;geneid=nfu_pred_436_mir-129;coords=sgr07:82688967-82689061

sgr07 nfu_mirna_prediction mature_miRNA 82688984 82689004 . + . ID=nfu_pred_436;biotype=5'-mature-miRNA;gene=mir-129;geneid=nfu_pred_436_mir-129;coords=sgr07:82688984-82689004

sgr07 nfu_mirna_prediction mature_miRNA 82689027 82689048 . + . ID=nfu_pred_436;biotype=3'-mature-miRNA;gene=mir-129;geneid=nfu_pred_436_mir-129;coords=sgr07:82689027-82689048

sgr07 nfu_mirna_prediction pre_miRNA 83740076 83740178 . - . ID=nfu_pred_437;biotype=pre-miRNA;gene=mir-458;geneid=nfu_pred_437_mir-458;coords=sgr07:83740076-83740178

sgr07 nfu_mirna_prediction pre_miRNA 84887668 84887761 . - . ID=nfu_pred_438;biotype=pre-miRNA;gene=mir-577;geneid=nfu_pred_438_mir-577;coords=sgr07:84887668-84887761

sgr07 nfu_mirna_prediction mature_miRNA 84887731 84887752 . - . ID=nfu_pred_438;biotype=5'-mature-miRNA;gene=mir-577;geneid=nfu_pred_438_mir-577;coords=sgr07:84887731-84887752

sgr07 nfu_mirna_prediction mature_miRNA 84887687 84887707 . - . ID=nfu_pred_438;biotype=3'-mature-miRNA;gene=mir-577;geneid=nfu_pred_438_mir-577;coords=sgr07:84887687-84887707

sgr07 nfu_mirna_prediction pre_miRNA 95909306 95909369 . - . ID=nfu_pred_439;biotype=pre-miRNA;gene=;geneid=nfu_pred_439_;coords=sgr07:95909306-95909369

sgr07 nfu_mirna_prediction mature_miRNA 95909306 95909327 . - . ID=nfu_pred_439;biotype=5'-mature-miRNA;gene=;geneid=nfu_pred_439_;coords=sgr07:95909306-95909327

sgr07 nfu_mirna_prediction mature_miRNA 95909347 95909368 . - . ID=nfu_pred_439;biotype=3'-mature-miRNA;gene=;geneid=nfu_pred_439_;coords=sgr07:95909347-95909368

sgr08 nfu_mirna_prediction pre_miRNA 1255522 1255600 . - . ID=nfu_pred_440;biotype=pre-miRNA;gene=mir-92;geneid=nfu_pred_440_mir-92;coords=sgr08:1255522-1255600

sgr08 nfu_mirna_prediction mature_miRNA 1255570 1255591 . - . ID=nfu_pred_440;biotype=5'-mature-miRNA;gene=mir-92;geneid=nfu_pred_440_mir-92;coords=sgr08:1255570-1255591

sgr08 nfu_mirna_prediction mature_miRNA 1255533 1255554 . - . ID=nfu_pred_440;biotype=3'-mature-miRNA;gene=mir-92;geneid=nfu_pred_440_mir-92;coords=sgr08:1255533-1255554

sgr08 nfu_mirna_prediction pre_miRNA 2323534 2323620 . + . ID=nfu_pred_441;biotype=pre-miRNA;gene=mir-216;geneid=nfu_pred_441_mir-216;coords=sgr08:2323534-2323620

sgr08 nfu_mirna_prediction mature_miRNA 2323549 2323570 . + . ID=nfu_pred_441;biotype=5'-mature-miRNA;gene=mir-216;geneid=nfu_pred_441_mir-216;coords=sgr08:2323549-2323570

sgr08 nfu_mirna_prediction mature_miRNA 2323581 2323603 . + . ID=nfu_pred_441;biotype=3'-mature-miRNA;gene=mir-216;geneid=nfu_pred_441_mir-216;coords=sgr08:2323581-2323603

sgr08 nfu_mirna_prediction pre_miRNA 2323720 2323807 . + . ID=nfu_pred_442;biotype=pre-miRNA;gene=mir-216;geneid=nfu_pred_442_mir-216;coords=sgr08:2323720-2323807

sgr08 nfu_mirna_prediction mature_miRNA 2323735 2323756 . + . ID=nfu_pred_442;biotype=5'-mature-miRNA;gene=mir-216;geneid=nfu_pred_442_mir-216;coords=sgr08:2323735-2323756

sgr08 nfu_mirna_prediction mature_miRNA 2323769 2323789 . + . ID=nfu_pred_442;biotype=3'-mature-miRNA;gene=mir-216;geneid=nfu_pred_442_mir-216;coords=sgr08:2323769-2323789

sgr08 nfu_mirna_prediction pre_miRNA 2324050 2324170 . + . ID=nfu_pred_443;biotype=pre-miRNA;gene=mir-217;geneid=nfu_pred_443_mir-217;coords=sgr08:2324050-2324170

sgr08 nfu_mirna_prediction mature_miRNA 2324081 2324102 . + . ID=nfu_pred_443;biotype=5'-mature-miRNA;gene=mir-217;geneid=nfu_pred_443_mir-217;coords=sgr08:2324081-2324102

sgr08 nfu_mirna_prediction pre_miRNA 3602521 3602637 . + . ID=nfu_pred_444;biotype=pre-miRNA;gene=mir-8094;geneid=nfu_pred_444_mir-8094;coords=sgr08:3602521-3602637

sgr08 nfu_mirna_prediction pre_miRNA 5252749 5252834 . + . ID=nfu_pred_445;biotype=pre-miRNA;gene=mir-599;geneid=nfu_pred_445_mir-599;coords=sgr08:5252749-5252834

sgr08 nfu_mirna_prediction mature_miRNA 5252759 5252781 . + . ID=nfu_pred_445;biotype=5'-mature-miRNA;gene=mir-599;geneid=nfu_pred_445_mir-599;coords=sgr08:5252759-5252781

sgr08 nfu_mirna_prediction mature_miRNA 5252795 5252815 . + . ID=nfu_pred_445;biotype=3'-mature-miRNA;gene=mir-599;geneid=nfu_pred_445_mir-599;coords=sgr08:5252795-5252815

sgr08 nfu_mirna_prediction pre_miRNA 7461252 7461337 . - . ID=nfu_pred_446;biotype=pre-miRNA;gene=mir-101;geneid=nfu_pred_446_mir-101;coords=sgr08:7461252-7461337

sgr08 nfu_mirna_prediction mature_miRNA 7461300 7461320 . - . ID=nfu_pred_446;biotype=5'-mature-miRNA;gene=mir-101;geneid=nfu_pred_446_mir-101;coords=sgr08:7461300-7461320

sgr08 nfu_mirna_prediction mature_miRNA 7461265 7461285 . - . ID=nfu_pred_446;biotype=3'-mature-miRNA;gene=mir-101;geneid=nfu_pred_446_mir-101;coords=sgr08:7461265-7461285

sgr08 nfu_mirna_prediction pre_miRNA 11033034 11033131 . - . ID=nfu_pred_448;biotype=pre-miRNA;gene=mir-137;geneid=nfu_pred_448_mir-137;coords=sgr08:11033034-11033131

sgr08 nfu_mirna_prediction mature_miRNA 11033052 11033073 . - . ID=nfu_pred_448;biotype=3'-mature-miRNA;gene=mir-137;geneid=nfu_pred_448_mir-137;coords=sgr08:11033052-11033073

sgr08 nfu_mirna_prediction pre_miRNA 11523311 11523420 . - . ID=nfu_pred_449;biotype=pre-miRNA;gene=mir-365;geneid=nfu_pred_449_mir-365;coords=sgr08:11523311-11523420

sgr08 nfu_mirna_prediction mature_miRNA 11523376 11523397 . - . ID=nfu_pred_449;biotype=5'-mature-miRNA;gene=mir-365;geneid=nfu_pred_449_mir-365;coords=sgr08:11523376-11523397

sgr08 nfu_mirna_prediction mature_miRNA 11523334 11523355 . - . ID=nfu_pred_449;biotype=3'-mature-miRNA;gene=mir-365;geneid=nfu_pred_449_mir-365;coords=sgr08:11523334-11523355

sgr08 nfu_mirna_prediction pre_miRNA 11525759 11525845 . - . ID=nfu_pred_450;biotype=pre-miRNA;gene=mir-193;geneid=nfu_pred_450_mir-193;coords=sgr08:11525759-11525845

sgr08 nfu_mirna_prediction mature_miRNA 11525814 11525835 . - . ID=nfu_pred_450;biotype=5'-mature-miRNA;gene=mir-193;geneid=nfu_pred_450_mir-193;coords=sgr08:11525814-11525835

sgr08 nfu_mirna_prediction mature_miRNA 11525778 11525799 . - . ID=nfu_pred_450;biotype=3'-mature-miRNA;gene=mir-193;geneid=nfu_pred_450_mir-193;coords=sgr08:11525778-11525799

sgr08 nfu_mirna_prediction pre_miRNA 11835718 11835828 . + . ID=nfu_pred_451;biotype=pre-miRNA;gene=mir-722;geneid=nfu_pred_451_mir-722;coords=sgr08:11835718-11835828

sgr08 nfu_mirna_prediction pre_miRNA 16313730 16313847 . - . ID=nfu_pred_452;biotype=pre-miRNA;gene=mir-218;geneid=nfu_pred_452_mir-218;coords=sgr08:16313730-16313847

sgr08 nfu_mirna_prediction mature_miRNA 16313798 16313819 . - . ID=nfu_pred_452;biotype=5'-mature-miRNA;gene=mir-218;geneid=nfu_pred_452_mir-218;coords=sgr08:16313798-16313819

sgr08 nfu_mirna_prediction mature_miRNA 16313757 16313777 . - . ID=nfu_pred_452;biotype=3'-mature-miRNA;gene=mir-218;geneid=nfu_pred_452_mir-218;coords=sgr08:16313757-16313777

sgr08 nfu_mirna_prediction pre_miRNA 20865172 20865253 . - . ID=nfu_pred_453;biotype=pre-miRNA;gene=mir-7147;geneid=nfu_pred_453_mir-7147;coords=sgr08:20865172-20865253

sgr08 nfu_mirna_prediction mature_miRNA 20865219 20865240 . - . ID=nfu_pred_453;biotype=5'-mature-miRNA;gene=mir-7147;geneid=nfu_pred_453_mir-7147;coords=sgr08:20865219-20865240

sgr08 nfu_mirna_prediction mature_miRNA 20865183 20865203 . - . ID=nfu_pred_453;biotype=3'-mature-miRNA;gene=mir-7147;geneid=nfu_pred_453_mir-7147;coords=sgr08:20865183-20865203

sgr08 nfu_mirna_prediction pre_miRNA 20932697 20932812 . + . ID=nfu_pred_454;biotype=pre-miRNA;gene=mir-811;geneid=nfu_pred_454_mir-811;coords=sgr08:20932697-20932812

sgr08 nfu_mirna_prediction mature_miRNA 20932720 20932744 . + . ID=nfu_pred_454;biotype=5'-mature-miRNA;gene=mir-811;geneid=nfu_pred_454_mir-811;coords=sgr08:20932720-20932744

sgr08 nfu_mirna_prediction mature_miRNA 20932779 20932800 . + . ID=nfu_pred_454;biotype=3'-mature-miRNA;gene=mir-811;geneid=nfu_pred_454_mir-811;coords=sgr08:20932779-20932800

sgr08 nfu_mirna_prediction pre_miRNA 25589549 25589648 . - . ID=nfu_pred_455;biotype=pre-miRNA;gene=mir-137;geneid=nfu_pred_455_mir-137;coords=sgr08:25589549-25589648

sgr08 nfu_mirna_prediction mature_miRNA 25589608 25589629 . - . ID=nfu_pred_455;biotype=5'-mature-miRNA;gene=mir-137;geneid=nfu_pred_455_mir-137;coords=sgr08:25589608-25589629

sgr08 nfu_mirna_prediction mature_miRNA 25589669 25589691 . - . ID=nfu_pred_455;biotype=3'-mature-miRNA;gene=mir-137;geneid=nfu_pred_455_mir-137;coords=sgr08:25589669-25589691

sgr08 nfu_mirna_prediction pre_miRNA 28927149 28927229 . - . ID=nfu_pred_456;biotype=pre-miRNA;gene=mir-4683;geneid=nfu_pred_456_mir-4683;coords=sgr08:28927149-28927229

sgr08 nfu_mirna_prediction pre_miRNA 35572401 35572477 . - . ID=nfu_pred_457;biotype=pre-miRNA;gene=;geneid=nfu_pred_457_;coords=sgr08:35572401-35572477

sgr08 nfu_mirna_prediction mature_miRNA 35572411 35572432 . - . ID=nfu_pred_457;biotype=3'-mature-miRNA;gene=;geneid=nfu_pred_457_;coords=sgr08:35572411-35572432

sgr08 nfu_mirna_prediction pre_miRNA 42122477 42122555 . - . ID=nfu_pred_458;biotype=pre-miRNA;gene=mir-26;geneid=nfu_pred_458_mir-26;coords=sgr08:42122477-42122555

sgr08 nfu_mirna_prediction mature_miRNA 42122524 42122545 . - . ID=nfu_pred_458;biotype=5'-mature-miRNA;gene=mir-26;geneid=nfu_pred_458_mir-26;coords=sgr08:42122524-42122545

sgr08 nfu_mirna_prediction pre_miRNA 43344500 43344579 . + . ID=nfu_pred_459;biotype=pre-miRNA;gene=mir-124;geneid=nfu_pred_459_mir-124;coords=sgr08:43344500-43344579

sgr08 nfu_mirna_prediction mature_miRNA 43344511 43344532 . + . ID=nfu_pred_459;biotype=5'-mature-miRNA;gene=mir-124;geneid=nfu_pred_459_mir-124;coords=sgr08:43344511-43344532

sgr08 nfu_mirna_prediction mature_miRNA 43344548 43344568 . + . ID=nfu_pred_459;biotype=3'-mature-miRNA;gene=mir-124;geneid=nfu_pred_459_mir-124;coords=sgr08:43344548-43344568

sgr08 nfu_mirna_prediction pre_miRNA 45694865 45694960 . + . ID=nfu_pred_460;biotype=pre-miRNA;gene=;geneid=nfu_pred_460_;coords=sgr08:45694865-45694960

sgr08 nfu_mirna_prediction mature_miRNA 45694886 45694906 . + . ID=nfu_pred_460;biotype=5'-mature-miRNA;gene=;geneid=nfu_pred_460_;coords=sgr08:45694886-45694906

sgr08 nfu_mirna_prediction mature_miRNA 45694917 45694939 . + . ID=nfu_pred_460;biotype=3'-mature-miRNA;gene=;geneid=nfu_pred_460_;coords=sgr08:45694917-45694939

sgr08 nfu_mirna_prediction pre_miRNA 51698393 51698474 . - . ID=nfu_pred_461;biotype=pre-miRNA;gene=mir-728;geneid=nfu_pred_461_mir-728;coords=sgr08:51698393-51698474

sgr08 nfu_mirna_prediction mature_miRNA 51698441 51698464 . - . ID=nfu_pred_461;biotype=5'-mature-miRNA;gene=mir-728;geneid=nfu_pred_461_mir-728;coords=sgr08:51698441-51698464

sgr08 nfu_mirna_prediction mature_miRNA 51698403 51698424 . - . ID=nfu_pred_461;biotype=3'-mature-miRNA;gene=mir-728;geneid=nfu_pred_461_mir-728;coords=sgr08:51698403-51698424

sgr08 nfu_mirna_prediction pre_miRNA 51704613 51704710 . - . ID=nfu_pred_462;biotype=pre-miRNA;gene=mir-477;geneid=nfu_pred_462_mir-477;coords=sgr08:51704613-51704710

sgr08 nfu_mirna_prediction mature_miRNA 51704673 51704695 . - . ID=nfu_pred_462;biotype=5'-mature-miRNA;gene=mir-477;geneid=nfu_pred_462_mir-477;coords=sgr08:51704673-51704695

sgr08 nfu_mirna_prediction mature_miRNA 51704633 51704653 . - . ID=nfu_pred_462;biotype=3'-mature-miRNA;gene=mir-477;geneid=nfu_pred_462_mir-477;coords=sgr08:51704633-51704653

sgr08 nfu_mirna_prediction pre_miRNA 55315318 55315376 . + . ID=nfu_pred_463;biotype=pre-miRNA;gene=mir-2;geneid=nfu_pred_463_mir-2;coords=sgr08:55315318-55315376

sgr08 nfu_mirna_prediction pre_miRNA 55945745 55945825 . + . ID=nfu_pred_464;biotype=pre-miRNA;gene=mir-4683;geneid=nfu_pred_464_mir-4683;coords=sgr08:55945745-55945825

sgr09 nfu_mirna_prediction pre_miRNA 2122394 2122483 . - . ID=nfu_pred_465;biotype=pre-miRNA;gene=mir-802;geneid=nfu_pred_465_mir-802;coords=sgr09:2122394-2122483

sgr09 nfu_mirna_prediction pre_miRNA 3552876 3552954 . + . ID=nfu_pred_466;biotype=pre-miRNA;gene=;geneid=nfu_pred_466_;coords=sgr09:3552876-3552954

sgr09 nfu_mirna_prediction pre_miRNA 8794528 8794625 . + . ID=nfu_pred_467;biotype=pre-miRNA;gene=mir-642;geneid=nfu_pred_467_mir-642;coords=sgr09:8794528-8794625

sgr09 nfu_mirna_prediction mature_miRNA 8794556 8794578 . + . ID=nfu_pred_467;biotype=5'-mature-miRNA;gene=mir-642;geneid=nfu_pred_467_mir-642;coords=sgr09:8794556-8794578

sgr09 nfu_mirna_prediction pre_miRNA 9269244 9269324 . - . ID=nfu_pred_468;biotype=pre-miRNA;gene=mir-396;geneid=nfu_pred_468_mir-396;coords=sgr09:9269244-9269324

sgr09 nfu_mirna_prediction mature_miRNA 9269299 9269320 . - . ID=nfu_pred_468;biotype=5'-mature-miRNA;gene=mir-396;geneid=nfu_pred_468_mir-396;coords=sgr09:9269299-9269320

sgr09 nfu_mirna_prediction mature_miRNA 9269258 9269279 . - . ID=nfu_pred_468;biotype=3'-mature-miRNA;gene=mir-396;geneid=nfu_pred_468_mir-396;coords=sgr09:9269258-9269279

sgr09 nfu_mirna_prediction pre_miRNA 11516901 11516967 . + . ID=nfu_pred_469;biotype=pre-miRNA;gene=;geneid=nfu_pred_469_;coords=sgr09:11516901-11516967

sgr09 nfu_mirna_prediction pre_miRNA 14159715 14159860 . + . ID=nfu_pred_470;biotype=pre-miRNA;gene=mir-821;geneid=nfu_pred_470_mir-821;coords=sgr09:14159715-14159860

sgr09 nfu_mirna_prediction pre_miRNA 14297259 14297361 . + . ID=nfu_pred_471;biotype=pre-miRNA;gene=mir-396;geneid=nfu_pred_471_mir-396;coords=sgr09:14297259-14297361

sgr09 nfu_mirna_prediction mature_miRNA 14297278 14297298 . + . ID=nfu_pred_471;biotype=5'-mature-miRNA;gene=mir-396;geneid=nfu_pred_471_mir-396;coords=sgr09:14297278-14297298

sgr09 nfu_mirna_prediction mature_miRNA 14297319 14297340 . + . ID=nfu_pred_471;biotype=3'-mature-miRNA;gene=mir-396;geneid=nfu_pred_471_mir-396;coords=sgr09:14297319-14297340

sgr09 nfu_mirna_prediction pre_miRNA 18334640 18334732 . + . ID=nfu_pred_472;biotype=pre-miRNA;gene=mir-821;geneid=nfu_pred_472_mir-821;coords=sgr09:18334640-18334732

sgr09 nfu_mirna_prediction mature_miRNA 18334692 18334713 . + . ID=nfu_pred_472;biotype=3'-mature-miRNA;gene=mir-821;geneid=nfu_pred_472_mir-821;coords=sgr09:18334692-18334713

sgr09 nfu_mirna_prediction pre_miRNA 19920713 19920802 . - . ID=nfu_pred_473;biotype=pre-miRNA;gene=mir-820;geneid=nfu_pred_473_mir-820;coords=sgr09:19920713-19920802

sgr09 nfu_mirna_prediction mature_miRNA 19920771 19920792 . - . ID=nfu_pred_473;biotype=5'-mature-miRNA;gene=mir-820;geneid=nfu_pred_473_mir-820;coords=sgr09:19920771-19920792

sgr09 nfu_mirna_prediction mature_miRNA 19920733 19920753 . - . ID=nfu_pred_473;biotype=3'-mature-miRNA;gene=mir-820;geneid=nfu_pred_473_mir-820;coords=sgr09:19920733-19920753

sgr09 nfu_mirna_prediction pre_miRNA 20083606 20083662 . + . ID=nfu_pred_474;biotype=pre-miRNA;gene=mir-7641;geneid=nfu_pred_474_mir-7641;coords=sgr09:20083606-20083662

sgr09 nfu_mirna_prediction pre_miRNA 20473466 20473568 . - . ID=nfu_pred_475;biotype=pre-miRNA;gene=mir-205;geneid=nfu_pred_475_mir-205;coords=sgr09:20473466-20473568

sgr09 nfu_mirna_prediction mature_miRNA 20473520 20473541 . - . ID=nfu_pred_475;biotype=5'-mature-miRNA;gene=mir-205;geneid=nfu_pred_475_mir-205;coords=sgr09:20473520-20473541

sgr09 nfu_mirna_prediction pre_miRNA 20717794 20717900 . + . ID=nfu_pred_476;biotype=pre-miRNA;gene=mir-29;geneid=nfu_pred_476_mir-29;coords=sgr09:20717794-20717900

sgr09 nfu_mirna_prediction mature_miRNA 20717816 20717837 . + . ID=nfu_pred_476;biotype=5'-mature-miRNA;gene=mir-29;geneid=nfu_pred_476_mir-29;coords=sgr09:20717816-20717837

sgr09 nfu_mirna_prediction mature_miRNA 20717855 20717877 . + . ID=nfu_pred_476;biotype=3'-mature-miRNA;gene=mir-29;geneid=nfu_pred_476_mir-29;coords=sgr09:20717855-20717877

sgr09 nfu_mirna_prediction pre_miRNA 20718002 20718109 . + . ID=nfu_pred_477;biotype=pre-miRNA;gene=mir-29;geneid=nfu_pred_477_mir-29;coords=sgr09:20718002-20718109

sgr09 nfu_mirna_prediction mature_miRNA 20718028 20718049 . + . ID=nfu_pred_477;biotype=5'-mature-miRNA;gene=mir-29;geneid=nfu_pred_477_mir-29;coords=sgr09:20718028-20718049

sgr09 nfu_mirna_prediction mature_miRNA 20718064 20718085 . + . ID=nfu_pred_477;biotype=3'-mature-miRNA;gene=mir-29;geneid=nfu_pred_477_mir-29;coords=sgr09:20718064-20718085

sgr09 nfu_mirna_prediction pre_miRNA 22483551 22483641 . + . ID=nfu_pred_478;biotype=pre-miRNA;gene=let-7;geneid=nfu_pred_478_let-7;coords=sgr09:22483551-22483641

sgr09 nfu_mirna_prediction mature_miRNA 22483562 22483583 . + . ID=nfu_pred_478;biotype=5'-mature-miRNA;gene=let-7;geneid=nfu_pred_478_let-7;coords=sgr09:22483562-22483583

sgr09 nfu_mirna_prediction mature_miRNA 22483609 22483630 . + . ID=nfu_pred_478;biotype=3'-mature-miRNA;gene=let-7;geneid=nfu_pred_478_let-7;coords=sgr09:22483609-22483630

sgr09 nfu_mirna_prediction pre_miRNA 22483776 22483871 . + . ID=nfu_pred_479;biotype=pre-miRNA;gene=let-7;geneid=nfu_pred_479_let-7;coords=sgr09:22483776-22483871

sgr09 nfu_mirna_prediction mature_miRNA 22483787 22483808 . + . ID=nfu_pred_479;biotype=5'-mature-miRNA;gene=let-7;geneid=nfu_pred_479_let-7;coords=sgr09:22483787-22483808

sgr09 nfu_mirna_prediction mature_miRNA 22483842 22483863 . + . ID=nfu_pred_479;biotype=3'-mature-miRNA;gene=let-7;geneid=nfu_pred_479_let-7;coords=sgr09:22483842-22483863

sgr09 nfu_mirna_prediction pre_miRNA 28363524 28363619 . - . ID=nfu_pred_480;biotype=pre-miRNA;gene=mir-3596;geneid=nfu_pred_480_mir-3596;coords=sgr09:28363524-28363619

sgr09 nfu_mirna_prediction mature_miRNA 28363588 28363609 . - . ID=nfu_pred_480;biotype=5'-mature-miRNA;gene=mir-3596;geneid=nfu_pred_480_mir-3596;coords=sgr09:28363588-28363609

sgr09 nfu_mirna_prediction mature_miRNA 28363531 28363552 . - . ID=nfu_pred_480;biotype=3'-mature-miRNA;gene=mir-3596;geneid=nfu_pred_480_mir-3596;coords=sgr09:28363531-28363552

sgr09 nfu_mirna_prediction pre_miRNA 29092164 29092255 . - . ID=nfu_pred_481;biotype=pre-miRNA;gene=mir-549;geneid=nfu_pred_481_mir-549;coords=sgr09:29092164-29092255

sgr09 nfu_mirna_prediction mature_miRNA 29092212 29092232 . - . ID=nfu_pred_481;biotype=5'-mature-miRNA;gene=mir-549;geneid=nfu_pred_481_mir-549;coords=sgr09:29092212-29092232

sgr09 nfu_mirna_prediction pre_miRNA 31012506 31012586 . - . ID=nfu_pred_482;biotype=pre-miRNA;gene=mir-396;geneid=nfu_pred_482_mir-396;coords=sgr09:31012506-31012586

sgr09 nfu_mirna_prediction mature_miRNA 31012561 31012582 . - . ID=nfu_pred_482;biotype=5'-mature-miRNA;gene=mir-396;geneid=nfu_pred_482_mir-396;coords=sgr09:31012561-31012582

sgr09 nfu_mirna_prediction mature_miRNA 31012520 31012541 . - . ID=nfu_pred_482;biotype=3'-mature-miRNA;gene=mir-396;geneid=nfu_pred_482_mir-396;coords=sgr09:31012520-31012541

sgr09 nfu_mirna_prediction pre_miRNA 35356045 35356144 . + . ID=nfu_pred_483;biotype=pre-miRNA;gene=mir-576;geneid=nfu_pred_483_mir-576;coords=sgr09:35356045-35356144

sgr09 nfu_mirna_prediction mature_miRNA 35356060 35356080 . + . ID=nfu_pred_483;biotype=5'-mature-miRNA;gene=mir-576;geneid=nfu_pred_483_mir-576;coords=sgr09:35356060-35356080

sgr09 nfu_mirna_prediction pre_miRNA 38791100 38791174 . - . ID=nfu_pred_484;biotype=pre-miRNA;gene=;geneid=nfu_pred_484_;coords=sgr09:38791100-38791174

sgr09 nfu_mirna_prediction mature_miRNA 38791149 38791171 . - . ID=nfu_pred_484;biotype=5'-mature-miRNA;gene=;geneid=nfu_pred_484_;coords=sgr09:38791149-38791171

sgr09 nfu_mirna_prediction mature_miRNA 38791110 38791131 . - . ID=nfu_pred_484;biotype=3'-mature-miRNA;gene=;geneid=nfu_pred_484_;coords=sgr09:38791110-38791131

sgr09 nfu_mirna_prediction pre_miRNA 42331453 42331518 . + . ID=nfu_pred_485;biotype=pre-miRNA;gene=mir-724;geneid=nfu_pred_485_mir-724;coords=sgr09:42331453-42331518

sgr09 nfu_mirna_prediction mature_miRNA 42331457 42331477 . + . ID=nfu_pred_485;biotype=5'-mature-miRNA;gene=mir-724;geneid=nfu_pred_485_mir-724;coords=sgr09:42331457-42331477

sgr09 nfu_mirna_prediction mature_miRNA 42331494 42331515 . + . ID=nfu_pred_485;biotype=3'-mature-miRNA;gene=mir-724;geneid=nfu_pred_485_mir-724;coords=sgr09:42331494-42331515

sgr09 nfu_mirna_prediction pre_miRNA 43634223 43634332 . + . ID=nfu_pred_486;biotype=pre-miRNA;gene=mir-101;geneid=nfu_pred_486_mir-101;coords=sgr09:43634223-43634332

sgr09 nfu_mirna_prediction pre_miRNA 46051566 46051695 . + . ID=nfu_pred_487;biotype=pre-miRNA;gene=mir-408;geneid=nfu_pred_487_mir-408;coords=sgr09:46051566-46051695

sgr09 nfu_mirna_prediction pre_miRNA 46757120 46757200 . + . ID=nfu_pred_488;biotype=pre-miRNA;gene=;geneid=nfu_pred_488_;coords=sgr09:46757120-46757200

sgr09 nfu_mirna_prediction pre_miRNA 48679174 48679267 . + . ID=nfu_pred_817;biotype=pre-miRNA;gene=mir-150;geneid=nfu_pred_817_mir-150;coords=sgr09:48679174-48679267

sgr09 nfu_mirna_prediction pre_miRNA 53704606 53704684 . - . ID=nfu_pred_489;biotype=pre-miRNA;gene=mir-643;geneid=nfu_pred_489_mir-643;coords=sgr09:53704606-53704684

sgr09 nfu_mirna_prediction mature_miRNA 53704612 53704635 . - . ID=nfu_pred_489;biotype=3'-mature-miRNA;gene=mir-643;geneid=nfu_pred_489_mir-643;coords=sgr09:53704612-53704635

sgr09 nfu_mirna_prediction pre_miRNA 55661410 55661509 . + . ID=nfu_pred_490;biotype=pre-miRNA;gene=mir-476;geneid=nfu_pred_490_mir-476;coords=sgr09:55661410-55661509

sgr09 nfu_mirna_prediction mature_miRNA 55661470 55661490 . + . ID=nfu_pred_490;biotype=3'-mature-miRNA;gene=mir-476;geneid=nfu_pred_490_mir-476;coords=sgr09:55661470-55661490

sgr09 nfu_mirna_prediction pre_miRNA 55751180 55751273 . + . ID=nfu_pred_491;biotype=pre-miRNA;gene=;geneid=nfu_pred_491_;coords=sgr09:55751180-55751273

sgr09 nfu_mirna_prediction mature_miRNA 55751200 55751221 . + . ID=nfu_pred_491;biotype=5'-mature-miRNA;gene=;geneid=nfu_pred_491_;coords=sgr09:55751200-55751221

sgr09 nfu_mirna_prediction pre_miRNA 55926017 55926134 . + . ID=nfu_pred_492;biotype=pre-miRNA;gene=mir-499;geneid=nfu_pred_492_mir-499;coords=sgr09:55926017-55926134

sgr09 nfu_mirna_prediction mature_miRNA 55926048 55926069 . + . ID=nfu_pred_492;biotype=5'-mature-miRNA;gene=mir-499;geneid=nfu_pred_492_mir-499;coords=sgr09:55926048-55926069

sgr09 nfu_mirna_prediction mature_miRNA 55926085 55926106 . + . ID=nfu_pred_492;biotype=3'-mature-miRNA;gene=mir-499;geneid=nfu_pred_492_mir-499;coords=sgr09:55926085-55926106

sgr10 nfu_mirna_prediction pre_miRNA 375684 375799 . + . ID=nfu_pred_493;biotype=pre-miRNA;gene=mir-181;geneid=nfu_pred_493_mir-181;coords=sgr10:375684-375799

sgr10 nfu_mirna_prediction mature_miRNA 375717 375738 . + . ID=nfu_pred_493;biotype=5'-mature-miRNA;gene=mir-181;geneid=nfu_pred_493_mir-181;coords=sgr10:375717-375738

sgr10 nfu_mirna_prediction mature_miRNA 375755 375775 . + . ID=nfu_pred_493;biotype=3'-mature-miRNA;gene=mir-181;geneid=nfu_pred_493_mir-181;coords=sgr10:375755-375775

sgr10 nfu_mirna_prediction pre_miRNA 375853 375933 . + . ID=nfu_pred_494;biotype=pre-miRNA;gene=mir-181;geneid=nfu_pred_494_mir-181;coords=sgr10:375853-375933

sgr10 nfu_mirna_prediction mature_miRNA 375863 375884 . + . ID=nfu_pred_494;biotype=5'-mature-miRNA;gene=mir-181;geneid=nfu_pred_494_mir-181;coords=sgr10:375863-375884

sgr10 nfu_mirna_prediction pre_miRNA 701270 701322 . - . ID=nfu_pred_495;biotype=pre-miRNA;gene=mir-669;geneid=nfu_pred_495_mir-669;coords=sgr10:701270-701322

sgr10 nfu_mirna_prediction pre_miRNA 2990066 2990148 . - . ID=nfu_pred_496;biotype=pre-miRNA;gene=mir-605;geneid=nfu_pred_496_mir-605;coords=sgr10:2990066-2990148

sgr10 nfu_mirna_prediction mature_miRNA 2990118 2990138 . - . ID=nfu_pred_496;biotype=5'-mature-miRNA;gene=mir-605;geneid=nfu_pred_496_mir-605;coords=sgr10:2990118-2990138

sgr10 nfu_mirna_prediction mature_miRNA 2990082 2990103 . - . ID=nfu_pred_496;biotype=3'-mature-miRNA;gene=mir-605;geneid=nfu_pred_496_mir-605;coords=sgr10:2990082-2990103

sgr10 nfu_mirna_prediction pre_miRNA 6707060 6707148 . + . ID=nfu_pred_498;biotype=pre-miRNA;gene=mir-3618;geneid=nfu_pred_498_mir-3618;coords=sgr10:6707060-6707148

sgr10 nfu_mirna_prediction mature_miRNA 6707077 6707099 . + . ID=nfu_pred_498;biotype=5'-mature-miRNA;gene=mir-3618;geneid=nfu_pred_498_mir-3618;coords=sgr10:6707077-6707099

sgr10 nfu_mirna_prediction pre_miRNA 6707337 6707423 . + . ID=nfu_pred_499;biotype=pre-miRNA;gene=mir-1306;geneid=nfu_pred_499_mir-1306;coords=sgr10:6707337-6707423

sgr10 nfu_mirna_prediction mature_miRNA 6707352 6707373 . + . ID=nfu_pred_499;biotype=5'-mature-miRNA;gene=mir-1306;geneid=nfu_pred_499_mir-1306;coords=sgr10:6707352-6707373

sgr10 nfu_mirna_prediction mature_miRNA 6707393 6707413 . + . ID=nfu_pred_499;biotype=3'-mature-miRNA;gene=mir-1306;geneid=nfu_pred_499_mir-1306;coords=sgr10:6707393-6707413

sgr10 nfu_mirna_prediction pre_miRNA 10396468 10396548 . - . ID=nfu_pred_500;biotype=pre-miRNA;gene=mir-124;geneid=nfu_pred_500_mir-124;coords=sgr10:10396468-10396548

sgr10 nfu_mirna_prediction mature_miRNA 10396479 10396500 . - . ID=nfu_pred_500;biotype=3'-mature-miRNA;gene=mir-124;geneid=nfu_pred_500_mir-124;coords=sgr10:10396479-10396500

sgr10 nfu_mirna_prediction pre_miRNA 12207271 12207349 . + . ID=nfu_pred_501;biotype=pre-miRNA;gene=mir-458;geneid=nfu_pred_501_mir-458;coords=sgr10:12207271-12207349

sgr10 nfu_mirna_prediction mature_miRNA 12207319 12207340 . + . ID=nfu_pred_501;biotype=3'-mature-miRNA;gene=mir-458;geneid=nfu_pred_501_mir-458;coords=sgr10:12207319-12207340

sgr10 nfu_mirna_prediction pre_miRNA 17209178 17209286 . + . ID=nfu_pred_502;biotype=pre-miRNA;gene=mir-204;geneid=nfu_pred_502_mir-204;coords=sgr10:17209178-17209286

sgr10 nfu_mirna_prediction mature_miRNA 17209204 17209225 . + . ID=nfu_pred_502;biotype=5'-mature-miRNA;gene=mir-204;geneid=nfu_pred_502_mir-204;coords=sgr10:17209204-17209225

sgr10 nfu_mirna_prediction mature_miRNA 17209243 17209263 . + . ID=nfu_pred_502;biotype=3'-mature-miRNA;gene=mir-204;geneid=nfu_pred_502_mir-204;coords=sgr10:17209243-17209263

sgr10 nfu_mirna_prediction pre_miRNA 19463006 19463084 . + . ID=nfu_pred_503;biotype=pre-miRNA;gene=;geneid=nfu_pred_503_;coords=sgr10:19463006-19463084

sgr10 nfu_mirna_prediction mature_miRNA 19463019 19463041 . + . ID=nfu_pred_503;biotype=5'-mature-miRNA;gene=;geneid=nfu_pred_503_;coords=sgr10:19463019-19463041

sgr10 nfu_mirna_prediction mature_miRNA 19463053 19463074 . + . ID=nfu_pred_503;biotype=3'-mature-miRNA;gene=;geneid=nfu_pred_503_;coords=sgr10:19463053-19463074

sgr10 nfu_mirna_prediction pre_miRNA 19463588 19463688 . + . ID=nfu_pred_504;biotype=pre-miRNA;gene=mir-9;geneid=nfu_pred_504_mir-9;coords=sgr10:19463588-19463688

sgr10 nfu_mirna_prediction mature_miRNA 19463603 19463624 . + . ID=nfu_pred_504;biotype=5'-mature-miRNA;gene=mir-9;geneid=nfu_pred_504_mir-9;coords=sgr10:19463603-19463624

sgr10 nfu_mirna_prediction mature_miRNA 19463640 19463661 . + . ID=nfu_pred_504;biotype=3'-mature-miRNA;gene=mir-9;geneid=nfu_pred_504_mir-9;coords=sgr10:19463640-19463661

sgr10 nfu_mirna_prediction pre_miRNA 21547922 21548009 . + . ID=nfu_pred_505;biotype=pre-miRNA;gene=mir-22;geneid=nfu_pred_505_mir-22;coords=sgr10:21547922-21548009

sgr10 nfu_mirna_prediction mature_miRNA 21547936 21547956 . + . ID=nfu_pred_505;biotype=5'-mature-miRNA;gene=mir-22;geneid=nfu_pred_505_mir-22;coords=sgr10:21547936-21547956

sgr10 nfu_mirna_prediction mature_miRNA 21547977 21547998 . + . ID=nfu_pred_505;biotype=3'-mature-miRNA;gene=mir-22;geneid=nfu_pred_505_mir-22;coords=sgr10:21547977-21547998

sgr10 nfu_mirna_prediction pre_miRNA 22706091 22706185 . - . ID=nfu_pred_506;biotype=pre-miRNA;gene=mir-50;geneid=nfu_pred_506_mir-50;coords=sgr10:22706091-22706185

sgr10 nfu_mirna_prediction mature_miRNA 22706144 22706167 . - . ID=nfu_pred_506;biotype=5'-mature-miRNA;gene=mir-50;geneid=nfu_pred_506_mir-50;coords=sgr10:22706144-22706167

sgr10 nfu_mirna_prediction mature_miRNA 22706108 22706129 . - . ID=nfu_pred_506;biotype=3'-mature-miRNA;gene=mir-50;geneid=nfu_pred_506_mir-50;coords=sgr10:22706108-22706129

sgr10 nfu_mirna_prediction pre_miRNA 22735255 22735353 . - . ID=nfu_pred_507;biotype=pre-miRNA;gene=mir-299;geneid=nfu_pred_507_mir-299;coords=sgr10:22735255-22735353

sgr10 nfu_mirna_prediction mature_miRNA 22735312 22735334 . - . ID=nfu_pred_507;biotype=5'-mature-miRNA;gene=mir-299;geneid=nfu_pred_507_mir-299;coords=sgr10:22735312-22735334

sgr10 nfu_mirna_prediction mature_miRNA 22735275 22735297 . - . ID=nfu_pred_507;biotype=3'-mature-miRNA;gene=mir-299;geneid=nfu_pred_507_mir-299;coords=sgr10:22735275-22735297

sgr10 nfu_mirna_prediction pre_miRNA 23555714 23555811 . - . ID=nfu_pred_508;biotype=pre-miRNA;gene=mir-6720;geneid=nfu_pred_508_mir-6720;coords=sgr10:23555714-23555811

sgr10 nfu_mirna_prediction pre_miRNA 23638688 23638769 . + . ID=nfu_pred_509;biotype=pre-miRNA;gene=mir-669;geneid=nfu_pred_509_mir-669;coords=sgr10:23638688-23638769

sgr10 nfu_mirna_prediction pre_miRNA 25627945 25628034 . + . ID=nfu_pred_510;biotype=pre-miRNA;gene=mir-7;geneid=nfu_pred_510_mir-7;coords=sgr10:25627945-25628034

sgr10 nfu_mirna_prediction mature_miRNA 25627959 25627981 . + . ID=nfu_pred_510;biotype=5'-mature-miRNA;gene=mir-7;geneid=nfu_pred_510_mir-7;coords=sgr10:25627959-25627981

sgr10 nfu_mirna_prediction mature_miRNA 25628001 25628021 . + . ID=nfu_pred_510;biotype=3'-mature-miRNA;gene=mir-7;geneid=nfu_pred_510_mir-7;coords=sgr10:25628001-25628021

sgr10 nfu_mirna_prediction pre_miRNA 28510343 28510440 . + . ID=nfu_pred_511;biotype=pre-miRNA;gene=mir-6720;geneid=nfu_pred_511_mir-6720;coords=sgr10:28510343-28510440

sgr10 nfu_mirna_prediction pre_miRNA 28851687 28851782 . + . ID=nfu_pred_512;biotype=pre-miRNA;gene=mir-23;geneid=nfu_pred_512_mir-23;coords=sgr10:28851687-28851782

sgr10 nfu_mirna_prediction mature_miRNA 28851704 28851725 . + . ID=nfu_pred_512;biotype=5'-mature-miRNA;gene=mir-23;geneid=nfu_pred_512_mir-23;coords=sgr10:28851704-28851725

sgr10 nfu_mirna_prediction mature_miRNA 28851742 28851763 . + . ID=nfu_pred_512;biotype=3'-mature-miRNA;gene=mir-23;geneid=nfu_pred_512_mir-23;coords=sgr10:28851742-28851763

sgr10 nfu_mirna_prediction pre_miRNA 28851987 28852084 . + . ID=nfu_pred_513;biotype=pre-miRNA;gene=mir-27;geneid=nfu_pred_513_mir-27;coords=sgr10:28851987-28852084

sgr10 nfu_mirna_prediction mature_miRNA 28852007 28852028 . + . ID=nfu_pred_513;biotype=5'-mature-miRNA;gene=mir-27;geneid=nfu_pred_513_mir-27;coords=sgr10:28852007-28852028

sgr10 nfu_mirna_prediction mature_miRNA 28852049 28852069 . + . ID=nfu_pred_513;biotype=3'-mature-miRNA;gene=mir-27;geneid=nfu_pred_513_mir-27;coords=sgr10:28852049-28852069

sgr10 nfu_mirna_prediction pre_miRNA 28857818 28857889 . + . ID=nfu_pred_514;biotype=pre-miRNA;gene=mir-24;geneid=nfu_pred_514_mir-24;coords=sgr10:28857818-28857889

sgr10 nfu_mirna_prediction mature_miRNA 28857823 28857843 . + . ID=nfu_pred_514;biotype=5'-mature-miRNA;gene=mir-24;geneid=nfu_pred_514_mir-24;coords=sgr10:28857823-28857843

sgr10 nfu_mirna_prediction mature_miRNA 28857860 28857881 . + . ID=nfu_pred_514;biotype=3'-mature-miRNA;gene=mir-24;geneid=nfu_pred_514_mir-24;coords=sgr10:28857860-28857881

sgr10 nfu_mirna_prediction pre_miRNA 35331610 35331688 . - . ID=nfu_pred_516;biotype=pre-miRNA;gene=mir-449;geneid=nfu_pred_516_mir-449;coords=sgr10:35331610-35331688

sgr10 nfu_mirna_prediction mature_miRNA 35331657 35331678 . - . ID=nfu_pred_516;biotype=5'-mature-miRNA;gene=mir-449;geneid=nfu_pred_516_mir-449;coords=sgr10:35331657-35331678

sgr10 nfu_mirna_prediction mature_miRNA 35331620 35331641 . - . ID=nfu_pred_516;biotype=3'-mature-miRNA;gene=mir-449;geneid=nfu_pred_516_mir-449;coords=sgr10:35331620-35331641

sgr10 nfu_mirna_prediction pre_miRNA 35331751 35331838 . - . ID=nfu_pred_517;biotype=pre-miRNA;gene=mir-449;geneid=nfu_pred_517_mir-449;coords=sgr10:35331751-35331838

sgr11 nfu_mirna_prediction pre_miRNA 83026 83123 . + . ID=nfu_pred_518;biotype=pre-miRNA;gene=mir-155;geneid=nfu_pred_518_mir-155;coords=sgr11:83026-83123

sgr11 nfu_mirna_prediction mature_miRNA 83045 83066 . + . ID=nfu_pred_518;biotype=5'-mature-miRNA;gene=mir-155;geneid=nfu_pred_518_mir-155;coords=sgr11:83045-83066

sgr11 nfu_mirna_prediction pre_miRNA 104766 104842 . + . ID=nfu_pred_519;biotype=pre-miRNA;gene=mir-17;geneid=nfu_pred_519_mir-17;coords=sgr11:104766-104842

sgr11 nfu_mirna_prediction mature_miRNA 104776 104797 . + . ID=nfu_pred_519;biotype=5'-mature-miRNA;gene=mir-17;geneid=nfu_pred_519_mir-17;coords=sgr11:104776-104797

sgr11 nfu_mirna_prediction mature_miRNA 104815 104835 . + . ID=nfu_pred_519;biotype=3'-mature-miRNA;gene=mir-17;geneid=nfu_pred_519_mir-17;coords=sgr11:104815-104835

sgr11 nfu_mirna_prediction pre_miRNA 104893 104973 . + . ID=nfu_pred_520;biotype=pre-miRNA;gene=mir-17;geneid=nfu_pred_520_mir-17;coords=sgr11:104893-104973

sgr11 nfu_mirna_prediction mature_miRNA 104903 104924 . + . ID=nfu_pred_520;biotype=5'-mature-miRNA;gene=mir-17;geneid=nfu_pred_520_mir-17;coords=sgr11:104903-104924

sgr11 nfu_mirna_prediction mature_miRNA 104944 104965 . + . ID=nfu_pred_520;biotype=3'-mature-miRNA;gene=mir-17;geneid=nfu_pred_520_mir-17;coords=sgr11:104944-104965

sgr11 nfu_mirna_prediction pre_miRNA 105031 105109 . + . ID=nfu_pred_521;biotype=pre-miRNA;gene=mir-19;geneid=nfu_pred_521_mir-19;coords=sgr11:105031-105109

sgr11 nfu_mirna_prediction mature_miRNA 105040 105061 . + . ID=nfu_pred_521;biotype=5'-mature-miRNA;gene=mir-19;geneid=nfu_pred_521_mir-19;coords=sgr11:105040-105061

sgr11 nfu_mirna_prediction mature_miRNA 105078 105099 . + . ID=nfu_pred_521;biotype=3'-mature-miRNA;gene=mir-19;geneid=nfu_pred_521_mir-19;coords=sgr11:105078-105099

sgr11 nfu_mirna_prediction pre_miRNA 105236 105312 . + . ID=nfu_pred_522;biotype=pre-miRNA;gene=mir-17;geneid=nfu_pred_522_mir-17;coords=sgr11:105236-105312

sgr11 nfu_mirna_prediction mature_miRNA 105246 105267 . + . ID=nfu_pred_522;biotype=5'-mature-miRNA;gene=mir-17;geneid=nfu_pred_522_mir-17;coords=sgr11:105246-105267

sgr11 nfu_mirna_prediction mature_miRNA 105284 105305 . + . ID=nfu_pred_522;biotype=3'-mature-miRNA;gene=mir-17;geneid=nfu_pred_522_mir-17;coords=sgr11:105284-105305

sgr11 nfu_mirna_prediction pre_miRNA 105368 105446 . + . ID=nfu_pred_523;biotype=pre-miRNA;gene=mir-19;geneid=nfu_pred_523_mir-19;coords=sgr11:105368-105446

sgr11 nfu_mirna_prediction mature_miRNA 105374 105395 . + . ID=nfu_pred_523;biotype=5'-mature-miRNA;gene=mir-19;geneid=nfu_pred_523_mir-19;coords=sgr11:105374-105395

sgr11 nfu_mirna_prediction mature_miRNA 105415 105436 . + . ID=nfu_pred_523;biotype=3'-mature-miRNA;gene=mir-19;geneid=nfu_pred_523_mir-19;coords=sgr11:105415-105436

sgr11 nfu_mirna_prediction pre_miRNA 105531 105609 . + . ID=nfu_pred_524;biotype=pre-miRNA;gene=mir-92;geneid=nfu_pred_524_mir-92;coords=sgr11:105531-105609

sgr11 nfu_mirna_prediction mature_miRNA 105545 105566 . + . ID=nfu_pred_524;biotype=5'-mature-miRNA;gene=mir-92;geneid=nfu_pred_524_mir-92;coords=sgr11:105545-105566

sgr11 nfu_mirna_prediction mature_miRNA 105578 105599 . + . ID=nfu_pred_524;biotype=3'-mature-miRNA;gene=mir-92;geneid=nfu_pred_524_mir-92;coords=sgr11:105578-105599

sgr11 nfu_mirna_prediction pre_miRNA 11184691 11184774 . + . ID=nfu_pred_525;biotype=pre-miRNA;gene=mir-605;geneid=nfu_pred_525_mir-605;coords=sgr11:11184691-11184774

sgr11 nfu_mirna_prediction mature_miRNA 11184700 11184722 . + . ID=nfu_pred_525;biotype=5'-mature-miRNA;gene=mir-605;geneid=nfu_pred_525_mir-605;coords=sgr11:11184700-11184722

sgr11 nfu_mirna_prediction mature_miRNA 11184738 11184760 . + . ID=nfu_pred_525;biotype=3'-mature-miRNA;gene=mir-605;geneid=nfu_pred_525_mir-605;coords=sgr11:11184738-11184760

sgr11 nfu_mirna_prediction pre_miRNA 11607674 11607780 . - . ID=nfu_pred_526;biotype=pre-miRNA;gene=mir-204;geneid=nfu_pred_526_mir-204;coords=sgr11:11607674-11607780

sgr11 nfu_mirna_prediction mature_miRNA 11607732 11607754 . - . ID=nfu_pred_526;biotype=5'-mature-miRNA;gene=mir-204;geneid=nfu_pred_526_mir-204;coords=sgr11:11607732-11607754

sgr11 nfu_mirna_prediction pre_miRNA 12194194 12194291 . + . ID=nfu_pred_527;biotype=pre-miRNA;gene=mir-6720;geneid=nfu_pred_527_mir-6720;coords=sgr11:12194194-12194291

sgr11 nfu_mirna_prediction pre_miRNA 12375840 12375937 . - . ID=nfu_pred_528;biotype=pre-miRNA;gene=mir-184;geneid=nfu_pred_528_mir-184;coords=sgr11:12375840-12375937

sgr11 nfu_mirna_prediction mature_miRNA 12375899 12375919 . - . ID=nfu_pred_528;biotype=5'-mature-miRNA;gene=mir-184;geneid=nfu_pred_528_mir-184;coords=sgr11:12375899-12375919

sgr11 nfu_mirna_prediction mature_miRNA 12375857 12375878 . - . ID=nfu_pred_528;biotype=3'-mature-miRNA;gene=mir-184;geneid=nfu_pred_528_mir-184;coords=sgr11:12375857-12375878

sgr11 nfu_mirna_prediction pre_miRNA 22199936 22200014 . - . ID=nfu_pred_529;biotype=pre-miRNA;gene=mir-9;geneid=nfu_pred_529_mir-9;coords=sgr11:22199936-22200014

sgr11 nfu_mirna_prediction mature_miRNA 22199982 22200004 . - . ID=nfu_pred_529;biotype=5'-mature-miRNA;gene=mir-9;geneid=nfu_pred_529_mir-9;coords=sgr11:22199982-22200004

sgr11 nfu_mirna_prediction mature_miRNA 22199945 22199965 . - . ID=nfu_pred_529;biotype=3'-mature-miRNA;gene=mir-9;geneid=nfu_pred_529_mir-9;coords=sgr11:22199945-22199965

sgr11 nfu_mirna_prediction pre_miRNA 22205219 22205297 . + . ID=nfu_pred_530;biotype=pre-miRNA;gene=;geneid=nfu_pred_530_;coords=sgr11:22205219-22205297

sgr11 nfu_mirna_prediction mature_miRNA 22205230 22205250 . + . ID=nfu_pred_530;biotype=5'-mature-miRNA;gene=;geneid=nfu_pred_530_;coords=sgr11:22205230-22205250

sgr11 nfu_mirna_prediction mature_miRNA 22205269 22205290 . + . ID=nfu_pred_530;biotype=3'-mature-miRNA;gene=;geneid=nfu_pred_530_;coords=sgr11:22205269-22205290

sgr11 nfu_mirna_prediction pre_miRNA 22575664 22575761 . + . ID=nfu_pred_531;biotype=pre-miRNA;gene=mir-6720;geneid=nfu_pred_531_mir-6720;coords=sgr11:22575664-22575761

sgr11 nfu_mirna_prediction pre_miRNA 25102905 25102981 . - . ID=nfu_pred_532;biotype=pre-miRNA;gene=;geneid=nfu_pred_532_;coords=sgr11:25102905-25102981

sgr11 nfu_mirna_prediction mature_miRNA 25102915 25102947 . - . ID=nfu_pred_532;biotype=3'-mature-miRNA;gene=;geneid=nfu_pred_532_;coords=sgr11:25102915-25102947

sgr11 nfu_mirna_prediction pre_miRNA 28378577 28378674 . + . ID=nfu_pred_533;biotype=pre-miRNA;gene=mir-765;geneid=nfu_pred_533_mir-765;coords=sgr11:28378577-28378674

sgr11 nfu_mirna_prediction mature_miRNA 28378632 28378654 . + . ID=nfu_pred_533;biotype=3'-mature-miRNA;gene=mir-765;geneid=nfu_pred_533_mir-765;coords=sgr11:28378632-28378654

sgr11 nfu_mirna_prediction pre_miRNA 38188069 38188151 . - . ID=nfu_pred_534;biotype=pre-miRNA;gene=mir-147;geneid=nfu_pred_534_mir-147;coords=sgr11:38188069-38188151

sgr11 nfu_mirna_prediction pre_miRNA 43606751 43606829 . + . ID=nfu_pred_535;biotype=pre-miRNA;gene=;geneid=nfu_pred_535_;coords=sgr11:43606751-43606829

sgr11 nfu_mirna_prediction mature_miRNA 43606798 43606818 . + . ID=nfu_pred_535;biotype=3'-mature-miRNA;gene=;geneid=nfu_pred_535_;coords=sgr11:43606798-43606818

sgr11 nfu_mirna_prediction pre_miRNA 46037487 46037588 . - . ID=nfu_pred_536;biotype=pre-miRNA;gene=mir-1338;geneid=nfu_pred_536_mir-1338;coords=sgr11:46037487-46037588

sgr11 nfu_mirna_prediction pre_miRNA 53194836 53194920 . + . ID=nfu_pred_537;biotype=pre-miRNA;gene=mir-662;geneid=nfu_pred_537_mir-662;coords=sgr11:53194836-53194920

sgr11 nfu_mirna_prediction mature_miRNA 53194884 53194905 . + . ID=nfu_pred_537;biotype=3'-mature-miRNA;gene=mir-662;geneid=nfu_pred_537_mir-662;coords=sgr11:53194884-53194905

sgr11 nfu_mirna_prediction pre_miRNA 60700718 60700815 . - . ID=nfu_pred_538;biotype=pre-miRNA;gene=mir-357;geneid=nfu_pred_538_mir-357;coords=sgr11:60700718-60700815

sgr11 nfu_mirna_prediction mature_miRNA 60700738 60700759 . - . ID=nfu_pred_538;biotype=3'-mature-miRNA;gene=mir-357;geneid=nfu_pred_538_mir-357;coords=sgr11:60700738-60700759

sgr12 nfu_mirna_prediction pre_miRNA 4325345 4325479 . - . ID=nfu_pred_539;biotype=pre-miRNA;gene=mir-2184;geneid=nfu_pred_539_mir-2184;coords=sgr12:4325345-4325479

sgr12 nfu_mirna_prediction pre_miRNA 5795795 5795916 . + . ID=nfu_pred_540;biotype=pre-miRNA;gene=mir-31;geneid=nfu_pred_540_mir-31;coords=sgr12:5795795-5795916

sgr12 nfu_mirna_prediction pre_miRNA 7130341 7130446 . - . ID=nfu_pred_541;biotype=pre-miRNA;gene=mir-29;geneid=nfu_pred_541_mir-29;coords=sgr12:7130341-7130446

sgr12 nfu_mirna_prediction mature_miRNA 7130362 7130383 . - . ID=nfu_pred_541;biotype=3'-mature-miRNA;gene=mir-29;geneid=nfu_pred_541_mir-29;coords=sgr12:7130362-7130383

sgr12 nfu_mirna_prediction pre_miRNA 7130574 7130654 . - . ID=nfu_pred_542;biotype=pre-miRNA;gene=mir-29;geneid=nfu_pred_542_mir-29;coords=sgr12:7130574-7130654

sgr12 nfu_mirna_prediction mature_miRNA 7130622 7130645 . - . ID=nfu_pred_542;biotype=5'-mature-miRNA;gene=mir-29;geneid=nfu_pred_542_mir-29;coords=sgr12:7130622-7130645

sgr12 nfu_mirna_prediction mature_miRNA 7130585 7130606 . - . ID=nfu_pred_542;biotype=3'-mature-miRNA;gene=mir-29;geneid=nfu_pred_542_mir-29;coords=sgr12:7130585-7130606

sgr12 nfu_mirna_prediction pre_miRNA 7961993 7962102 . + . ID=nfu_pred_543;biotype=pre-miRNA;gene=mir-205;geneid=nfu_pred_543_mir-205;coords=sgr12:7961993-7962102

sgr12 nfu_mirna_prediction mature_miRNA 7962020 7962041 . + . ID=nfu_pred_543;biotype=5'-mature-miRNA;gene=mir-205;geneid=nfu_pred_543_mir-205;coords=sgr12:7962020-7962041

sgr12 nfu_mirna_prediction pre_miRNA 8808403 8808493 . + . ID=nfu_pred_544;biotype=pre-miRNA;gene=let-7;geneid=nfu_pred_544_let-7;coords=sgr12:8808403-8808493

sgr12 nfu_mirna_prediction mature_miRNA 8808413 8808434 . + . ID=nfu_pred_544;biotype=5'-mature-miRNA;gene=let-7;geneid=nfu_pred_544_let-7;coords=sgr12:8808413-8808434

sgr12 nfu_mirna_prediction mature_miRNA 8808462 8808484 . + . ID=nfu_pred_544;biotype=3'-mature-miRNA;gene=let-7;geneid=nfu_pred_544_let-7;coords=sgr12:8808462-8808484

sgr12 nfu_mirna_prediction pre_miRNA 8808777 8808880 . + . ID=nfu_pred_545;biotype=pre-miRNA;gene=mir-3596;geneid=nfu_pred_545_mir-3596;coords=sgr12:8808777-8808880

sgr12 nfu_mirna_prediction mature_miRNA 8808788 8808809 . + . ID=nfu_pred_545;biotype=5'-mature-miRNA;gene=mir-3596;geneid=nfu_pred_545_mir-3596;coords=sgr12:8808788-8808809

sgr12 nfu_mirna_prediction mature_miRNA 8808851 8808871 . + . ID=nfu_pred_545;biotype=3'-mature-miRNA;gene=mir-3596;geneid=nfu_pred_545_mir-3596;coords=sgr12:8808851-8808871

sgr12 nfu_mirna_prediction pre_miRNA 9234788 9234868 . + . ID=nfu_pred_546;biotype=pre-miRNA;gene=mir-8;geneid=nfu_pred_546_mir-8;coords=sgr12:9234788-9234868

sgr12 nfu_mirna_prediction mature_miRNA 9234836 9234857 . + . ID=nfu_pred_546;biotype=3'-mature-miRNA;gene=mir-8;geneid=nfu_pred_546_mir-8;coords=sgr12:9234836-9234857

sgr12 nfu_mirna_prediction pre_miRNA 9234968 9235047 . + . ID=nfu_pred_547;biotype=pre-miRNA;gene=mir-8;geneid=nfu_pred_547_mir-8;coords=sgr12:9234968-9235047

sgr12 nfu_mirna_prediction mature_miRNA 9234976 9234998 . + . ID=nfu_pred_547;biotype=5'-mature-miRNA;gene=mir-8;geneid=nfu_pred_547_mir-8;coords=sgr12:9234976-9234998

sgr12 nfu_mirna_prediction mature_miRNA 9235016 9235036 . + . ID=nfu_pred_547;biotype=3'-mature-miRNA;gene=mir-8;geneid=nfu_pred_547_mir-8;coords=sgr12:9235016-9235036

sgr12 nfu_mirna_prediction pre_miRNA 9236277 9236355 . + . ID=nfu_pred_548;biotype=pre-miRNA;gene=mir-8;geneid=nfu_pred_548_mir-8;coords=sgr12:9236277-9236355

sgr12 nfu_mirna_prediction mature_miRNA 9236324 9236344 . + . ID=nfu_pred_548;biotype=3'-mature-miRNA;gene=mir-8;geneid=nfu_pred_548_mir-8;coords=sgr12:9236324-9236344

sgr12 nfu_mirna_prediction pre_miRNA 9236475 9236550 . + . ID=nfu_pred_549;biotype=pre-miRNA;gene=mir-7149;geneid=nfu_pred_549_mir-7149;coords=sgr12:9236475-9236550

sgr12 nfu_mirna_prediction mature_miRNA 9236481 9236501 . + . ID=nfu_pred_549;biotype=5'-mature-miRNA;gene=mir-7149;geneid=nfu_pred_549_mir-7149;coords=sgr12:9236481-9236501

sgr12 nfu_mirna_prediction pre_miRNA 11386361 11386455 . - . ID=nfu_pred_550;biotype=pre-miRNA;gene=mir-563;geneid=nfu_pred_550_mir-563;coords=sgr12:11386361-11386455

sgr12 nfu_mirna_prediction mature_miRNA 11386429 11386450 . - . ID=nfu_pred_550;biotype=5'-mature-miRNA;gene=mir-563;geneid=nfu_pred_550_mir-563;coords=sgr12:11386429-11386450

sgr12 nfu_mirna_prediction pre_miRNA 11494168 11494262 . + . ID=nfu_pred_551;biotype=pre-miRNA;gene=mir-135;geneid=nfu_pred_551_mir-135;coords=sgr12:11494168-11494262

sgr12 nfu_mirna_prediction mature_miRNA 11494185 11494206 . + . ID=nfu_pred_551;biotype=5'-mature-miRNA;gene=mir-135;geneid=nfu_pred_551_mir-135;coords=sgr12:11494185-11494206

sgr12 nfu_mirna_prediction mature_miRNA 11494224 11494244 . + . ID=nfu_pred_551;biotype=3'-mature-miRNA;gene=mir-135;geneid=nfu_pred_551_mir-135;coords=sgr12:11494224-11494244

sgr12 nfu_mirna_prediction pre_miRNA 11494514 11494592 . + . ID=nfu_pred_552;biotype=pre-miRNA;gene=;geneid=nfu_pred_552_;coords=sgr12:11494514-11494592

sgr12 nfu_mirna_prediction mature_miRNA 11494525 11494546 . + . ID=nfu_pred_552;biotype=5'-mature-miRNA;gene=;geneid=nfu_pred_552_;coords=sgr12:11494525-11494546

sgr12 nfu_mirna_prediction mature_miRNA 11494561 11494581 . + . ID=nfu_pred_552;biotype=3'-mature-miRNA;gene=;geneid=nfu_pred_552_;coords=sgr12:11494561-11494581

sgr12 nfu_mirna_prediction pre_miRNA 11555224 11555323 . + . ID=nfu_pred_553;biotype=pre-miRNA;gene=mir-337;geneid=nfu_pred_553_mir-337;coords=sgr12:11555224-11555323

sgr12 nfu_mirna_prediction mature_miRNA 11555237 11555259 . + . ID=nfu_pred_553;biotype=5'-mature-miRNA;gene=mir-337;geneid=nfu_pred_553_mir-337;coords=sgr12:11555237-11555259

sgr12 nfu_mirna_prediction pre_miRNA 11555563 11555660 . + . ID=nfu_pred_554;biotype=pre-miRNA;gene=mir-425;geneid=nfu_pred_554_mir-425;coords=sgr12:11555563-11555660

sgr12 nfu_mirna_prediction mature_miRNA 11555581 11555603 . + . ID=nfu_pred_554;biotype=5'-mature-miRNA;gene=mir-425;geneid=nfu_pred_554_mir-425;coords=sgr12:11555581-11555603

sgr12 nfu_mirna_prediction pre_miRNA 16909239 16909328 . - . ID=nfu_pred_555;biotype=pre-miRNA;gene=mir-9861;geneid=nfu_pred_555_mir-9861;coords=sgr12:16909239-16909328

sgr12 nfu_mirna_prediction pre_miRNA 17679958 17680045 . - . ID=nfu_pred_556;biotype=pre-miRNA;gene=mir-34;geneid=nfu_pred_556_mir-34;coords=sgr12:17679958-17680045

sgr12 nfu_mirna_prediction mature_miRNA 17680010 17680032 . - . ID=nfu_pred_556;biotype=5'-mature-miRNA;gene=mir-34;geneid=nfu_pred_556_mir-34;coords=sgr12:17680010-17680032

sgr12 nfu_mirna_prediction mature_miRNA 17679970 17679990 . - . ID=nfu_pred_556;biotype=3'-mature-miRNA;gene=mir-34;geneid=nfu_pred_556_mir-34;coords=sgr12:17679970-17679990

sgr12 nfu_mirna_prediction pre_miRNA 17700382 17700462 . + . ID=nfu_pred_557;biotype=pre-miRNA;gene=;geneid=nfu_pred_557_;coords=sgr12:17700382-17700462

sgr12 nfu_mirna_prediction mature_miRNA 17700392 17700413 . + . ID=nfu_pred_557;biotype=5'-mature-miRNA;gene=;geneid=nfu_pred_557_;coords=sgr12:17700392-17700413

sgr12 nfu_mirna_prediction mature_miRNA 17700439 17700459 . + . ID=nfu_pred_557;biotype=3'-mature-miRNA;gene=;geneid=nfu_pred_557_;coords=sgr12:17700439-17700459

sgr12 nfu_mirna_prediction pre_miRNA 18688271 18688330 . - . ID=nfu_pred_558;biotype=pre-miRNA;gene=mir-724;geneid=nfu_pred_558_mir-724;coords=sgr12:18688271-18688330

sgr12 nfu_mirna_prediction mature_miRNA 18688309 18688330 . - . ID=nfu_pred_558;biotype=5'-mature-miRNA;gene=mir-724;geneid=nfu_pred_558_mir-724;coords=sgr12:18688309-18688330

sgr12 nfu_mirna_prediction mature_miRNA 18688272 18688293 . - . ID=nfu_pred_558;biotype=3'-mature-miRNA;gene=mir-724;geneid=nfu_pred_558_mir-724;coords=sgr12:18688272-18688293

sgr12 nfu_mirna_prediction pre_miRNA 19555840 19555928 . + . ID=nfu_pred_559;biotype=pre-miRNA;gene=let-7;geneid=nfu_pred_559_let-7;coords=sgr12:19555840-19555928

sgr12 nfu_mirna_prediction mature_miRNA 19555848 19555869 . + . ID=nfu_pred_559;biotype=5'-mature-miRNA;gene=let-7;geneid=nfu_pred_559_let-7;coords=sgr12:19555848-19555869

sgr12 nfu_mirna_prediction mature_miRNA 19555898 19555918 . + . ID=nfu_pred_559;biotype=3'-mature-miRNA;gene=let-7;geneid=nfu_pred_559_let-7;coords=sgr12:19555898-19555918

sgr12 nfu_mirna_prediction pre_miRNA 19556353 19556453 . + . ID=nfu_pred_560;biotype=pre-miRNA;gene=let-7;geneid=nfu_pred_560_let-7;coords=sgr12:19556353-19556453

sgr12 nfu_mirna_prediction mature_miRNA 19556364 19556384 . + . ID=nfu_pred_560;biotype=5'-mature-miRNA;gene=let-7;geneid=nfu_pred_560_let-7;coords=sgr12:19556364-19556384

sgr12 nfu_mirna_prediction mature_miRNA 19556423 19556445 . + . ID=nfu_pred_560;biotype=3'-mature-miRNA;gene=let-7;geneid=nfu_pred_560_let-7;coords=sgr12:19556423-19556445

sgr12 nfu_mirna_prediction pre_miRNA 20272281 20272359 . + . ID=nfu_pred_561;biotype=pre-miRNA;gene=mir-203;geneid=nfu_pred_561_mir-203;coords=sgr12:20272281-20272359

sgr12 nfu_mirna_prediction mature_miRNA 20272291 20272312 . + . ID=nfu_pred_561;biotype=5'-mature-miRNA;gene=mir-203;geneid=nfu_pred_561_mir-203;coords=sgr12:20272291-20272312

sgr12 nfu_mirna_prediction mature_miRNA 20272328 20272349 . + . ID=nfu_pred_561;biotype=3'-mature-miRNA;gene=mir-203;geneid=nfu_pred_561_mir-203;coords=sgr12:20272328-20272349

sgr12 nfu_mirna_prediction pre_miRNA 20633013 20633106 . + . ID=nfu_pred_562;biotype=pre-miRNA;gene=mir-196;geneid=nfu_pred_562_mir-196;coords=sgr12:20633013-20633106

sgr12 nfu_mirna_prediction mature_miRNA 20633034 20633055 . + . ID=nfu_pred_562;biotype=5'-mature-miRNA;gene=mir-196;geneid=nfu_pred_562_mir-196;coords=sgr12:20633034-20633055

sgr12 nfu_mirna_prediction pre_miRNA 20664585 20664663 . + . ID=nfu_pred_563;biotype=pre-miRNA;gene=mir-10;geneid=nfu_pred_563_mir-10;coords=sgr12:20664585-20664663

sgr12 nfu_mirna_prediction mature_miRNA 20664595 20664616 . + . ID=nfu_pred_563;biotype=5'-mature-miRNA;gene=mir-10;geneid=nfu_pred_563_mir-10;coords=sgr12:20664595-20664616

sgr12 nfu_mirna_prediction pre_miRNA 20665391 20665465 . + . ID=nfu_pred_564;biotype=pre-miRNA;gene=mir-10;geneid=nfu_pred_564_mir-10;coords=sgr12:20665391-20665465

sgr12 nfu_mirna_prediction mature_miRNA 20665399 20665420 . + . ID=nfu_pred_564;biotype=5'-mature-miRNA;gene=mir-10;geneid=nfu_pred_564_mir-10;coords=sgr12:20665399-20665420

sgr12 nfu_mirna_prediction pre_miRNA 21936327 21936420 . + . ID=nfu_pred_565;biotype=pre-miRNA;gene=let-7;geneid=nfu_pred_565_let-7;coords=sgr12:21936327-21936420

sgr12 nfu_mirna_prediction mature_miRNA 21936339 21936360 . + . ID=nfu_pred_565;biotype=5'-mature-miRNA;gene=let-7;geneid=nfu_pred_565_let-7;coords=sgr12:21936339-21936360

sgr12 nfu_mirna_prediction mature_miRNA 21936390 21936410 . + . ID=nfu_pred_565;biotype=3'-mature-miRNA;gene=let-7;geneid=nfu_pred_565_let-7;coords=sgr12:21936390-21936410

sgr12 nfu_mirna_prediction pre_miRNA 21936579 21936677 . + . ID=nfu_pred_566;biotype=pre-miRNA;gene=let-7;geneid=nfu_pred_566_let-7;coords=sgr12:21936579-21936677

sgr12 nfu_mirna_prediction mature_miRNA 21936591 21936611 . + . ID=nfu_pred_566;biotype=5'-mature-miRNA;gene=let-7;geneid=nfu_pred_566_let-7;coords=sgr12:21936591-21936611

sgr12 nfu_mirna_prediction mature_miRNA 21936648 21936670 . + . ID=nfu_pred_566;biotype=3'-mature-miRNA;gene=let-7;geneid=nfu_pred_566_let-7;coords=sgr12:21936648-21936670

sgr12 nfu_mirna_prediction pre_miRNA 25597805 25597904 . + . ID=nfu_pred_569;biotype=pre-miRNA;gene=mir-1388;geneid=nfu_pred_569_mir-1388;coords=sgr12:25597805-25597904

sgr12 nfu_mirna_prediction mature_miRNA 25597828 25597849 . + . ID=nfu_pred_569;biotype=5'-mature-miRNA;gene=mir-1388;geneid=nfu_pred_569_mir-1388;coords=sgr12:25597828-25597849

sgr12 nfu_mirna_prediction mature_miRNA 25597865 25597886 . + . ID=nfu_pred_569;biotype=3'-mature-miRNA;gene=mir-1388;geneid=nfu_pred_569_mir-1388;coords=sgr12:25597865-25597886

sgr12 nfu_mirna_prediction pre_miRNA 25864340 25864421 . - . ID=nfu_pred_570;biotype=pre-miRNA;gene=mir-124;geneid=nfu_pred_570_mir-124;coords=sgr12:25864340-25864421

sgr12 nfu_mirna_prediction mature_miRNA 25864387 25864408 . - . ID=nfu_pred_570;biotype=5'-mature-miRNA;gene=mir-124;geneid=nfu_pred_570_mir-124;coords=sgr12:25864387-25864408

sgr12 nfu_mirna_prediction mature_miRNA 25864351 25864371 . - . ID=nfu_pred_570;biotype=3'-mature-miRNA;gene=mir-124;geneid=nfu_pred_570_mir-124;coords=sgr12:25864351-25864371

sgr12 nfu_mirna_prediction pre_miRNA 28220261 28220339 . - . ID=nfu_pred_571;biotype=pre-miRNA;gene=mir-26;geneid=nfu_pred_571_mir-26;coords=sgr12:28220261-28220339

sgr12 nfu_mirna_prediction mature_miRNA 28220308 28220329 . - . ID=nfu_pred_571;biotype=5'-mature-miRNA;gene=mir-26;geneid=nfu_pred_571_mir-26;coords=sgr12:28220308-28220329

sgr12 nfu_mirna_prediction pre_miRNA 33293729 33293785 . - . ID=nfu_pred_572;biotype=pre-miRNA;gene=mir-7641;geneid=nfu_pred_572_mir-7641;coords=sgr12:33293729-33293785

sgr12 nfu_mirna_prediction mature_miRNA 33293756 33293777 . - . ID=nfu_pred_572;biotype=5'-mature-miRNA;gene=mir-7641;geneid=nfu_pred_572_mir-7641;coords=sgr12:33293756-33293777

sgr12 nfu_mirna_prediction pre_miRNA 36843604 36843682 . - . ID=nfu_pred_573;biotype=pre-miRNA;gene=mir-BART15;geneid=nfu_pred_573_mir-BART15;coords=sgr12:36843604-36843682

sgr12 nfu_mirna_prediction mature_miRNA 36843651 36843672 . - . ID=nfu_pred_573;biotype=5'-mature-miRNA;gene=mir-BART15;geneid=nfu_pred_573_mir-BART15;coords=sgr12:36843651-36843672

sgr12 nfu_mirna_prediction mature_miRNA 36843615 36843636 . - . ID=nfu_pred_573;biotype=3'-mature-miRNA;gene=mir-BART15;geneid=nfu_pred_573_mir-BART15;coords=sgr12:36843615-36843636

sgr12 nfu_mirna_prediction pre_miRNA 37172470 37172540 . - . ID=nfu_pred_574;biotype=pre-miRNA;gene=;geneid=nfu_pred_574_;coords=sgr12:37172470-37172540

sgr12 nfu_mirna_prediction pre_miRNA 38626531 38626613 . - . ID=nfu_pred_575;biotype=pre-miRNA;gene=mir-548;geneid=nfu_pred_575_mir-548;coords=sgr12:38626531-38626613

sgr12 nfu_mirna_prediction mature_miRNA 38626583 38626603 . - . ID=nfu_pred_575;biotype=5'-mature-miRNA;gene=mir-548;geneid=nfu_pred_575_mir-548;coords=sgr12:38626583-38626603

sgr12 nfu_mirna_prediction mature_miRNA 38626545 38626565 . - . ID=nfu_pred_575;biotype=3'-mature-miRNA;gene=mir-548;geneid=nfu_pred_575_mir-548;coords=sgr12:38626545-38626565

sgr12 nfu_mirna_prediction pre_miRNA 39958223 39958305 . - . ID=nfu_pred_818;biotype=pre-miRNA;gene=mir-150;geneid=nfu_pred_818_mir-150;coords=sgr12:39958223-39958305

sgr12 nfu_mirna_prediction pre_miRNA 40962757 40962820 . - . ID=nfu_pred_576;biotype=pre-miRNA;gene=mir-4468;geneid=nfu_pred_576_mir-4468;coords=sgr12:40962757-40962820

sgr13 nfu_mirna_prediction pre_miRNA 1601031 1601137 . - . ID=nfu_pred_577;biotype=pre-miRNA;gene=mir-872;geneid=nfu_pred_577_mir-872;coords=sgr13:1601031-1601137

sgr13 nfu_mirna_prediction mature_miRNA 1601111 1601131 . - . ID=nfu_pred_577;biotype=5'-mature-miRNA;gene=mir-872;geneid=nfu_pred_577_mir-872;coords=sgr13:1601111-1601131

sgr13 nfu_mirna_prediction mature_miRNA 1601070 1601092 . - . ID=nfu_pred_577;biotype=3'-mature-miRNA;gene=mir-872;geneid=nfu_pred_577_mir-872;coords=sgr13:1601070-1601092

sgr13 nfu_mirna_prediction pre_miRNA 5126881 5127003 . - . ID=nfu_pred_578;biotype=pre-miRNA;gene=mir-455;geneid=nfu_pred_578_mir-455;coords=sgr13:5126881-5127003

sgr13 nfu_mirna_prediction mature_miRNA 5126912 5126932 . - . ID=nfu_pred_578;biotype=3'-mature-miRNA;gene=mir-455;geneid=nfu_pred_578_mir-455;coords=sgr13:5126912-5126932

sgr13 nfu_mirna_prediction pre_miRNA 6091366 6091464 . + . ID=nfu_pred_579;biotype=pre-miRNA;gene=mir-576;geneid=nfu_pred_579_mir-576;coords=sgr13:6091366-6091464

sgr13 nfu_mirna_prediction mature_miRNA 6091413 6091434 . + . ID=nfu_pred_579;biotype=3'-mature-miRNA;gene=mir-576;geneid=nfu_pred_579_mir-576;coords=sgr13:6091413-6091434

sgr13 nfu_mirna_prediction pre_miRNA 7284990 7285070 . + . ID=nfu_pred_580;biotype=pre-miRNA;gene=;geneid=nfu_pred_580_;coords=sgr13:7284990-7285070

sgr13 nfu_mirna_prediction mature_miRNA 7285045 7285065 . + . ID=nfu_pred_580;biotype=3'-mature-miRNA;gene=;geneid=nfu_pred_580_;coords=sgr13:7285045-7285065

sgr13 nfu_mirna_prediction pre_miRNA 11158338 11158428 . + . ID=nfu_pred_581;biotype=pre-miRNA;gene=let-7;geneid=nfu_pred_581_let-7;coords=sgr13:11158338-11158428

sgr13 nfu_mirna_prediction mature_miRNA 11158349 11158370 . + . ID=nfu_pred_581;biotype=5'-mature-miRNA;gene=let-7;geneid=nfu_pred_581_let-7;coords=sgr13:11158349-11158370

sgr13 nfu_mirna_prediction mature_miRNA 11158399 11158419 . + . ID=nfu_pred_581;biotype=3'-mature-miRNA;gene=let-7;geneid=nfu_pred_581_let-7;coords=sgr13:11158399-11158419

sgr13 nfu_mirna_prediction pre_miRNA 11160222 11160317 . + . ID=nfu_pred_582;biotype=pre-miRNA;gene=let-7;geneid=nfu_pred_582_let-7;coords=sgr13:11160222-11160317

sgr13 nfu_mirna_prediction mature_miRNA 11160233 11160253 . + . ID=nfu_pred_582;biotype=5'-mature-miRNA;gene=let-7;geneid=nfu_pred_582_let-7;coords=sgr13:11160233-11160253

sgr13 nfu_mirna_prediction mature_miRNA 11160288 11160310 . + . ID=nfu_pred_582;biotype=3'-mature-miRNA;gene=let-7;geneid=nfu_pred_582_let-7;coords=sgr13:11160288-11160310

sgr13 nfu_mirna_prediction pre_miRNA 12911226 12911304 . + . ID=nfu_pred_583;biotype=pre-miRNA;gene=mir-7641;geneid=nfu_pred_583_mir-7641;coords=sgr13:12911226-12911304

sgr13 nfu_mirna_prediction mature_miRNA 12911234 12911255 . + . ID=nfu_pred_583;biotype=5'-mature-miRNA;gene=mir-7641;geneid=nfu_pred_583_mir-7641;coords=sgr13:12911234-12911255

sgr13 nfu_mirna_prediction pre_miRNA 12938317 12938419 . - . ID=nfu_pred_584;biotype=pre-miRNA;gene=mir-29;geneid=nfu_pred_584_mir-29;coords=sgr13:12938317-12938419

sgr13 nfu_mirna_prediction mature_miRNA 12938339 12938359 . - . ID=nfu_pred_584;biotype=3'-mature-miRNA;gene=mir-29;geneid=nfu_pred_584_mir-29;coords=sgr13:12938339-12938359

sgr13 nfu_mirna_prediction pre_miRNA 14201073 14201155 . - . ID=nfu_pred_586;biotype=pre-miRNA;gene=;geneid=nfu_pred_586_;coords=sgr13:14201073-14201155

sgr13 nfu_mirna_prediction mature_miRNA 14201119 14201142 . - . ID=nfu_pred_586;biotype=5'-mature-miRNA;gene=;geneid=nfu_pred_586_;coords=sgr13:14201119-14201142

sgr13 nfu_mirna_prediction pre_miRNA 14202462 14202544 . - . ID=nfu_pred_592;biotype=pre-miRNA;gene=mir-716;geneid=nfu_pred_592_mir-716;coords=sgr13:14202462-14202544

sgr13 nfu_mirna_prediction mature_miRNA 14202475 14202495 . - . ID=nfu_pred_592;biotype=3'-mature-miRNA;gene=mir-716;geneid=nfu_pred_592_mir-716;coords=sgr13:14202475-14202495

sgr13 nfu_mirna_prediction pre_miRNA 19552470 19552549 . - . ID=nfu_pred_593;biotype=pre-miRNA;gene=mir-2192;geneid=nfu_pred_593_mir-2192;coords=sgr13:19552470-19552549

sgr13 nfu_mirna_prediction pre_miRNA 22164499 22164604 . - . ID=nfu_pred_594;biotype=pre-miRNA;gene=mir-204;geneid=nfu_pred_594_mir-204;coords=sgr13:22164499-22164604

sgr13 nfu_mirna_prediction mature_miRNA 22164557 22164578 . - . ID=nfu_pred_594;biotype=5'-mature-miRNA;gene=mir-204;geneid=nfu_pred_594_mir-204;coords=sgr13:22164557-22164578

sgr13 nfu_mirna_prediction pre_miRNA 22385105 22385202 . - . ID=nfu_pred_595;biotype=pre-miRNA;gene=mir-6720;geneid=nfu_pred_595_mir-6720;coords=sgr13:22385105-22385202

sgr13 nfu_mirna_prediction pre_miRNA 23186944 23187030 . + . ID=nfu_pred_596;biotype=pre-miRNA;gene=mir-190;geneid=nfu_pred_596_mir-190;coords=sgr13:23186944-23187030

sgr13 nfu_mirna_prediction mature_miRNA 23186958 23186978 . + . ID=nfu_pred_596;biotype=5'-mature-miRNA;gene=mir-190;geneid=nfu_pred_596_mir-190;coords=sgr13:23186958-23186978

sgr13 nfu_mirna_prediction mature_miRNA 23186995 23187025 . + . ID=nfu_pred_596;biotype=3'-mature-miRNA;gene=mir-190;geneid=nfu_pred_596_mir-190;coords=sgr13:23186995-23187025

sgr13 nfu_mirna_prediction pre_miRNA 23632489 23632586 . + . ID=nfu_pred_597;biotype=pre-miRNA;gene=mir-184;geneid=nfu_pred_597_mir-184;coords=sgr13:23632489-23632586

sgr13 nfu_mirna_prediction mature_miRNA 23632509 23632530 . + . ID=nfu_pred_597;biotype=5'-mature-miRNA;gene=mir-184;geneid=nfu_pred_597_mir-184;coords=sgr13:23632509-23632530

sgr13 nfu_mirna_prediction mature_miRNA 23632549 23632570 . + . ID=nfu_pred_597;biotype=3'-mature-miRNA;gene=mir-184;geneid=nfu_pred_597_mir-184;coords=sgr13:23632549-23632570

sgr13 nfu_mirna_prediction pre_miRNA 26784283 26784377 . + . ID=nfu_pred_598;biotype=pre-miRNA;gene=mir-811;geneid=nfu_pred_598_mir-811;coords=sgr13:26784283-26784377

sgr13 nfu_mirna_prediction mature_miRNA 26784337 26784357 . + . ID=nfu_pred_598;biotype=3'-mature-miRNA;gene=mir-811;geneid=nfu_pred_598_mir-811;coords=sgr13:26784337-26784357

sgr13 nfu_mirna_prediction pre_miRNA 27366403 27366494 . + . ID=nfu_pred_599;biotype=pre-miRNA;gene=mir-129;geneid=nfu_pred_599_mir-129;coords=sgr13:27366403-27366494

sgr13 nfu_mirna_prediction mature_miRNA 27366420 27366440 . + . ID=nfu_pred_599;biotype=5'-mature-miRNA;gene=mir-129;geneid=nfu_pred_599_mir-129;coords=sgr13:27366420-27366440

sgr13 nfu_mirna_prediction mature_miRNA 27366460 27366481 . + . ID=nfu_pred_599;biotype=3'-mature-miRNA;gene=mir-129;geneid=nfu_pred_599_mir-129;coords=sgr13:27366460-27366481

sgr13 nfu_mirna_prediction pre_miRNA 29189816 29189902 . - . ID=nfu_pred_600;biotype=pre-miRNA;gene=mir-182;geneid=nfu_pred_600_mir-182;coords=sgr13:29189816-29189902

sgr13 nfu_mirna_prediction mature_miRNA 29189870 29189891 . - . ID=nfu_pred_600;biotype=5'-mature-miRNA;gene=mir-182;geneid=nfu_pred_600_mir-182;coords=sgr13:29189870-29189891

sgr13 nfu_mirna_prediction pre_miRNA 29190121 29190207 . - . ID=nfu_pred_601;biotype=pre-miRNA;gene=mir-96;geneid=nfu_pred_601_mir-96;coords=sgr13:29190121-29190207

sgr13 nfu_mirna_prediction mature_miRNA 29190171 29190192 . - . ID=nfu_pred_601;biotype=5'-mature-miRNA;gene=mir-96;geneid=nfu_pred_601_mir-96;coords=sgr13:29190171-29190192

sgr13 nfu_mirna_prediction pre_miRNA 29190250 29190358 . - . ID=nfu_pred_602;biotype=pre-miRNA;gene=mir-183;geneid=nfu_pred_602_mir-183;coords=sgr13:29190250-29190358

sgr13 nfu_mirna_prediction mature_miRNA 29190311 29190333 . - . ID=nfu_pred_602;biotype=5'-mature-miRNA;gene=mir-183;geneid=nfu_pred_602_mir-183;coords=sgr13:29190311-29190333

sgr13 nfu_mirna_prediction pre_miRNA 29672206 29672300 . + . ID=nfu_pred_603;biotype=pre-miRNA;gene=mir-135;geneid=nfu_pred_603_mir-135;coords=sgr13:29672206-29672300

sgr13 nfu_mirna_prediction mature_miRNA 29672223 29672244 . + . ID=nfu_pred_603;biotype=5'-mature-miRNA;gene=mir-135;geneid=nfu_pred_603_mir-135;coords=sgr13:29672223-29672244

sgr13 nfu_mirna_prediction pre_miRNA 30029207 30029285 . + . ID=nfu_pred_604;biotype=pre-miRNA;gene=mir-9;geneid=nfu_pred_604_mir-9;coords=sgr13:30029207-30029285

sgr13 nfu_mirna_prediction mature_miRNA 30029217 30029239 . + . ID=nfu_pred_604;biotype=5'-mature-miRNA;gene=mir-9;geneid=nfu_pred_604_mir-9;coords=sgr13:30029217-30029239

sgr13 nfu_mirna_prediction mature_miRNA 30029256 30029276 . + . ID=nfu_pred_604;biotype=3'-mature-miRNA;gene=mir-9;geneid=nfu_pred_604_mir-9;coords=sgr13:30029256-30029276

sgr13 nfu_mirna_prediction pre_miRNA 32180343 32180440 . + . ID=nfu_pred_605;biotype=pre-miRNA;gene=mir-210;geneid=nfu_pred_605_mir-210;coords=sgr13:32180343-32180440

sgr13 nfu_mirna_prediction mature_miRNA 32180358 32180378 . + . ID=nfu_pred_605;biotype=5'-mature-miRNA;gene=mir-210;geneid=nfu_pred_605_mir-210;coords=sgr13:32180358-32180378

sgr13 nfu_mirna_prediction mature_miRNA 32180397 32180418 . + . ID=nfu_pred_605;biotype=3'-mature-miRNA;gene=mir-210;geneid=nfu_pred_605_mir-210;coords=sgr13:32180397-32180418

sgr13 nfu_mirna_prediction pre_miRNA 32569833 32569919 . + . ID=nfu_pred_819;biotype=pre-miRNA;gene=mir-193;geneid=nfu_pred_819_mir-193;coords=sgr13:32569833-32569919

sgr13 nfu_mirna_prediction pre_miRNA 34175322 34175365 . - . ID=nfu_pred_606;biotype=pre-miRNA;gene=mir-7549;geneid=nfu_pred_606_mir-7549;coords=sgr13:34175322-34175365

sgr13 nfu_mirna_prediction pre_miRNA 36974788 36974894 . + . ID=nfu_pred_608;biotype=pre-miRNA;gene=mir-728;geneid=nfu_pred_608_mir-728;coords=sgr13:36974788-36974894

sgr13 nfu_mirna_prediction pre_miRNA 38013091 38013177 . + . ID=nfu_pred_609;biotype=pre-miRNA;gene=mir-934;geneid=nfu_pred_609_mir-934;coords=sgr13:38013091-38013177

sgr13 nfu_mirna_prediction mature_miRNA 38013107 38013128 . + . ID=nfu_pred_609;biotype=5'-mature-miRNA;gene=mir-934;geneid=nfu_pred_609_mir-934;coords=sgr13:38013107-38013128

sgr13 nfu_mirna_prediction mature_miRNA 38013141 38013161 . + . ID=nfu_pred_609;biotype=3'-mature-miRNA;gene=mir-934;geneid=nfu_pred_609_mir-934;coords=sgr13:38013141-38013161

sgr13 nfu_mirna_prediction pre_miRNA 38729815 38729912 . + . ID=nfu_pred_610;biotype=pre-miRNA;gene=mir-6720;geneid=nfu_pred_610_mir-6720;coords=sgr13:38729815-38729912

sgr13 nfu_mirna_prediction pre_miRNA 39449373 39449461 . + . ID=nfu_pred_611;biotype=pre-miRNA;gene=mir-7;geneid=nfu_pred_611_mir-7;coords=sgr13:39449373-39449461

sgr13 nfu_mirna_prediction mature_miRNA 39449387 39449407 . + . ID=nfu_pred_611;biotype=5'-mature-miRNA;gene=mir-7;geneid=nfu_pred_611_mir-7;coords=sgr13:39449387-39449407

sgr13 nfu_mirna_prediction mature_miRNA 39449428 39449448 . + . ID=nfu_pred_611;biotype=3'-mature-miRNA;gene=mir-7;geneid=nfu_pred_611_mir-7;coords=sgr13:39449428-39449448

sgr13 nfu_mirna_prediction pre_miRNA 42403770 42403848 . + . ID=nfu_pred_612;biotype=pre-miRNA;gene=;geneid=nfu_pred_612_;coords=sgr13:42403770-42403848

sgr13 nfu_mirna_prediction pre_miRNA 42820226 42820324 . + . ID=nfu_pred_613;biotype=pre-miRNA;gene=mir-140;geneid=nfu_pred_613_mir-140;coords=sgr13:42820226-42820324

sgr13 nfu_mirna_prediction mature_miRNA 42820247 42820267 . + . ID=nfu_pred_613;biotype=5'-mature-miRNA;gene=mir-140;geneid=nfu_pred_613_mir-140;coords=sgr13:42820247-42820267

sgr13 nfu_mirna_prediction mature_miRNA 42820287 42820308 . + . ID=nfu_pred_613;biotype=3'-mature-miRNA;gene=mir-140;geneid=nfu_pred_613_mir-140;coords=sgr13:42820287-42820308

sgr13 nfu_mirna_prediction pre_miRNA 43008294 43008403 . + . ID=nfu_pred_614;biotype=pre-miRNA;gene=mir-6698;geneid=nfu_pred_614_mir-6698;coords=sgr13:43008294-43008403

sgr13 nfu_mirna_prediction pre_miRNA 43228044 43228133 . - . ID=nfu_pred_615;biotype=pre-miRNA;gene=mir-577;geneid=nfu_pred_615_mir-577;coords=sgr13:43228044-43228133

sgr13 nfu_mirna_prediction pre_miRNA 43353821 43353905 . + . ID=nfu_pred_616;biotype=pre-miRNA;gene=mir-138;geneid=nfu_pred_616_mir-138;coords=sgr13:43353821-43353905

sgr13 nfu_mirna_prediction mature_miRNA 43353831 43353853 . + . ID=nfu_pred_616;biotype=5'-mature-miRNA;gene=mir-138;geneid=nfu_pred_616_mir-138;coords=sgr13:43353831-43353853

sgr13 nfu_mirna_prediction mature_miRNA 43353878 43353898 . + . ID=nfu_pred_616;biotype=3'-mature-miRNA;gene=mir-138;geneid=nfu_pred_616_mir-138;coords=sgr13:43353878-43353898

sgr13 nfu_mirna_prediction pre_miRNA 43487086 43487162 . + . ID=nfu_pred_617;biotype=pre-miRNA;gene=;geneid=nfu_pred_617_;coords=sgr13:43487086-43487162

sgr13 nfu_mirna_prediction mature_miRNA 43487091 43487112 . + . ID=nfu_pred_617;biotype=5'-mature-miRNA;gene=;geneid=nfu_pred_617_;coords=sgr13:43487091-43487112

sgr13 nfu_mirna_prediction mature_miRNA 43487133 43487152 . + . ID=nfu_pred_617;biotype=3'-mature-miRNA;gene=;geneid=nfu_pred_617_;coords=sgr13:43487133-43487152

sgr14 nfu_mirna_prediction pre_miRNA 2042323 2042431 . - . ID=nfu_pred_618;biotype=pre-miRNA;gene=mir-187;geneid=nfu_pred_618_mir-187;coords=sgr14:2042323-2042431

sgr14 nfu_mirna_prediction mature_miRNA 2042339 2042361 . - . ID=nfu_pred_618;biotype=5'-mature-miRNA;gene=mir-187;geneid=nfu_pred_618_mir-187;coords=sgr14:2042339-2042361

sgr14 nfu_mirna_prediction mature_miRNA 2042401 2042420 . - . ID=nfu_pred_618;biotype=3'-mature-miRNA;gene=mir-187;geneid=nfu_pred_618_mir-187;coords=sgr14:2042401-2042420

sgr14 nfu_mirna_prediction pre_miRNA 2566903 2566981 . - . ID=nfu_pred_619;biotype=pre-miRNA;gene=;geneid=nfu_pred_619_;coords=sgr14:2566903-2566981

sgr14 nfu_mirna_prediction mature_miRNA 2566950 2566970 . - . ID=nfu_pred_619;biotype=5'-mature-miRNA;gene=;geneid=nfu_pred_619_;coords=sgr14:2566950-2566970

sgr14 nfu_mirna_prediction mature_miRNA 2566913 2566934 . - . ID=nfu_pred_619;biotype=3'-mature-miRNA;gene=;geneid=nfu_pred_619_;coords=sgr14:2566913-2566934

sgr14 nfu_mirna_prediction pre_miRNA 6397661 6397745 . + . ID=nfu_pred_621;biotype=pre-miRNA;gene=mir-149;geneid=nfu_pred_621_mir-149;coords=sgr14:6397661-6397745

sgr14 nfu_mirna_prediction mature_miRNA 6397675 6397695 . + . ID=nfu_pred_621;biotype=5'-mature-miRNA;gene=mir-149;geneid=nfu_pred_621_mir-149;coords=sgr14:6397675-6397695

sgr14 nfu_mirna_prediction pre_miRNA 11893617 11893695 . - . ID=nfu_pred_622;biotype=pre-miRNA;gene=mir-9;geneid=nfu_pred_622_mir-9;coords=sgr14:11893617-11893695

sgr14 nfu_mirna_prediction mature_miRNA 11893625 11893645 . - . ID=nfu_pred_622;biotype=5'-mature-miRNA;gene=mir-9;geneid=nfu_pred_622_mir-9;coords=sgr14:11893625-11893645

sgr14 nfu_mirna_prediction mature_miRNA 11893665 11893685 . - . ID=nfu_pred_622;biotype=3'-mature-miRNA;gene=mir-9;geneid=nfu_pred_622_mir-9;coords=sgr14:11893665-11893685

sgr14 nfu_mirna_prediction pre_miRNA 11948185 11948284 . + . ID=nfu_pred_623;biotype=pre-miRNA;gene=mir-642;geneid=nfu_pred_623_mir-642;coords=sgr14:11948185-11948284

sgr14 nfu_mirna_prediction mature_miRNA 11948200 11948220 . + . ID=nfu_pred_623;biotype=5'-mature-miRNA;gene=mir-642;geneid=nfu_pred_623_mir-642;coords=sgr14:11948200-11948220

sgr14 nfu_mirna_prediction mature_miRNA 11948237 11948257 . + . ID=nfu_pred_623;biotype=3'-mature-miRNA;gene=mir-642;geneid=nfu_pred_623_mir-642;coords=sgr14:11948237-11948257

sgr14 nfu_mirna_prediction pre_miRNA 11969115 11969214 . - . ID=nfu_pred_624;biotype=pre-miRNA;gene=mir-396;geneid=nfu_pred_624_mir-396;coords=sgr14:11969115-11969214

sgr14 nfu_mirna_prediction mature_miRNA 11969174 11969195 . - . ID=nfu_pred_624;biotype=5'-mature-miRNA;gene=mir-396;geneid=nfu_pred_624_mir-396;coords=sgr14:11969174-11969195

sgr14 nfu_mirna_prediction mature_miRNA 11969130 11969151 . - . ID=nfu_pred_624;biotype=3'-mature-miRNA;gene=mir-396;geneid=nfu_pred_624_mir-396;coords=sgr14:11969130-11969151

sgr14 nfu_mirna_prediction pre_miRNA 11973951 11974027 . - . ID=nfu_pred_625;biotype=pre-miRNA;gene=lin-4;geneid=nfu_pred_625_lin-4;coords=sgr14:11973951-11974027

sgr14 nfu_mirna_prediction mature_miRNA 11973960 11973980 . - . ID=nfu_pred_625;biotype=5'-mature-miRNA;gene=lin-4;geneid=nfu_pred_625_lin-4;coords=sgr14:11973960-11973980

sgr14 nfu_mirna_prediction mature_miRNA 11973996 11974012 . - . ID=nfu_pred_625;biotype=3'-mature-miRNA;gene=lin-4;geneid=nfu_pred_625_lin-4;coords=sgr14:11973996-11974012

sgr14 nfu_mirna_prediction pre_miRNA 11977550 11977638 . - . ID=nfu_pred_626;biotype=pre-miRNA;gene=let-7;geneid=nfu_pred_626_let-7;coords=sgr14:11977550-11977638

sgr14 nfu_mirna_prediction mature_miRNA 11977608 11977628 . - . ID=nfu_pred_626;biotype=5'-mature-miRNA;gene=let-7;geneid=nfu_pred_626_let-7;coords=sgr14:11977608-11977628

sgr14 nfu_mirna_prediction pre_miRNA 14749748 14749826 . - . ID=nfu_pred_627;biotype=pre-miRNA;gene=mir-821;geneid=nfu_pred_627_mir-821;coords=sgr14:14749748-14749826

sgr14 nfu_mirna_prediction mature_miRNA 14749755 14749778 . - . ID=nfu_pred_627;biotype=3'-mature-miRNA;gene=mir-821;geneid=nfu_pred_627_mir-821;coords=sgr14:14749755-14749778

sgr14 nfu_mirna_prediction pre_miRNA 17747536 17747613 . + . ID=nfu_pred_628;biotype=pre-miRNA;gene=mir-574;geneid=nfu_pred_628_mir-574;coords=sgr14:17747536-17747613

sgr14 nfu_mirna_prediction mature_miRNA 17747583 17747604 . + . ID=nfu_pred_628;biotype=3'-mature-miRNA;gene=mir-574;geneid=nfu_pred_628_mir-574;coords=sgr14:17747583-17747604

sgr14 nfu_mirna_prediction pre_miRNA 18351772 18351854 . - . ID=nfu_pred_630;biotype=pre-miRNA;gene=mir-30;geneid=nfu_pred_630_mir-30;coords=sgr14:18351772-18351854

sgr14 nfu_mirna_prediction mature_miRNA 18351822 18351844 . - . ID=nfu_pred_630;biotype=5'-mature-miRNA;gene=mir-30;geneid=nfu_pred_630_mir-30;coords=sgr14:18351822-18351844

sgr14 nfu_mirna_prediction mature_miRNA 18351785 18351805 . - . ID=nfu_pred_630;biotype=3'-mature-miRNA;gene=mir-30;geneid=nfu_pred_630_mir-30;coords=sgr14:18351785-18351805

sgr14 nfu_mirna_prediction pre_miRNA 18352032 18352112 . - . ID=nfu_pred_631;biotype=pre-miRNA;gene=mir-30;geneid=nfu_pred_631_mir-30;coords=sgr14:18352032-18352112

sgr14 nfu_mirna_prediction mature_miRNA 18352079 18352102 . - . ID=nfu_pred_631;biotype=5'-mature-miRNA;gene=mir-30;geneid=nfu_pred_631_mir-30;coords=sgr14:18352079-18352102

sgr14 nfu_mirna_prediction mature_miRNA 18352038 18352059 . - . ID=nfu_pred_631;biotype=3'-mature-miRNA;gene=mir-30;geneid=nfu_pred_631_mir-30;coords=sgr14:18352038-18352059

sgr14 nfu_mirna_prediction pre_miRNA 18728169 18728259 . + . ID=nfu_pred_632;biotype=pre-miRNA;gene=mir-128;geneid=nfu_pred_632_mir-128;coords=sgr14:18728169-18728259

sgr14 nfu_mirna_prediction mature_miRNA 18728183 18728203 . + . ID=nfu_pred_632;biotype=5'-mature-miRNA;gene=mir-128;geneid=nfu_pred_632_mir-128;coords=sgr14:18728183-18728203

sgr14 nfu_mirna_prediction mature_miRNA 18728221 18728241 . + . ID=nfu_pred_632;biotype=3'-mature-miRNA;gene=mir-128;geneid=nfu_pred_632_mir-128;coords=sgr14:18728221-18728241

sgr14 nfu_mirna_prediction pre_miRNA 19690762 19690844 . - . ID=nfu_pred_633;biotype=pre-miRNA;gene=mir-934;geneid=nfu_pred_633_mir-934;coords=sgr14:19690762-19690844

sgr14 nfu_mirna_prediction mature_miRNA 19690815 19690835 . - . ID=nfu_pred_633;biotype=5'-mature-miRNA;gene=mir-934;geneid=nfu_pred_633_mir-934;coords=sgr14:19690815-19690835

sgr14 nfu_mirna_prediction pre_miRNA 19974079 19974157 . + . ID=nfu_pred_634;biotype=pre-miRNA;gene=mir-219;geneid=nfu_pred_634_mir-219;coords=sgr14:19974079-19974157

sgr14 nfu_mirna_prediction mature_miRNA 19974089 19974110 . + . ID=nfu_pred_634;biotype=5'-mature-miRNA;gene=mir-219;geneid=nfu_pred_634_mir-219;coords=sgr14:19974089-19974110

sgr14 nfu_mirna_prediction mature_miRNA 19974132 19974153 . + . ID=nfu_pred_634;biotype=3'-mature-miRNA;gene=mir-219;geneid=nfu_pred_634_mir-219;coords=sgr14:19974132-19974153

sgr14 nfu_mirna_prediction pre_miRNA 21815903 21816015 . - . ID=nfu_pred_635;biotype=pre-miRNA;gene=mir-187;geneid=nfu_pred_635_mir-187;coords=sgr14:21815903-21816015

sgr14 nfu_mirna_prediction mature_miRNA 21815970 21815990 . - . ID=nfu_pred_635;biotype=5'-mature-miRNA;gene=mir-187;geneid=nfu_pred_635_mir-187;coords=sgr14:21815970-21815990

sgr14 nfu_mirna_prediction mature_miRNA 21815930 21815951 . - . ID=nfu_pred_635;biotype=3'-mature-miRNA;gene=mir-187;geneid=nfu_pred_635_mir-187;coords=sgr14:21815930-21815951

sgr14 nfu_mirna_prediction pre_miRNA 23428000 23428090 . + . ID=nfu_pred_637;biotype=pre-miRNA;gene=mir-934;geneid=nfu_pred_637_mir-934;coords=sgr14:23428000-23428090

sgr14 nfu_mirna_prediction mature_miRNA 23428052 23428072 . + . ID=nfu_pred_637;biotype=3'-mature-miRNA;gene=mir-934;geneid=nfu_pred_637_mir-934;coords=sgr14:23428052-23428072

sgr14 nfu_mirna_prediction pre_miRNA 23492076 23492158 . - . ID=nfu_pred_638;biotype=pre-miRNA;gene=;geneid=nfu_pred_638_;coords=sgr14:23492076-23492158

sgr14 nfu_mirna_prediction pre_miRNA 23912591 23912671 . - . ID=nfu_pred_639;biotype=pre-miRNA;gene=mir-423;geneid=nfu_pred_639_mir-423;coords=sgr14:23912591-23912671

sgr14 nfu_mirna_prediction mature_miRNA 23912640 23912661 . - . ID=nfu_pred_639;biotype=5'-mature-miRNA;gene=mir-423;geneid=nfu_pred_639_mir-423;coords=sgr14:23912640-23912661

sgr14 nfu_mirna_prediction mature_miRNA 23912595 23912615 . - . ID=nfu_pred_639;biotype=3'-mature-miRNA;gene=mir-423;geneid=nfu_pred_639_mir-423;coords=sgr14:23912595-23912615

sgr15 nfu_mirna_prediction pre_miRNA 4368234 4368312 . + . ID=nfu_pred_640;biotype=pre-miRNA;gene=;geneid=nfu_pred_640_;coords=sgr15:4368234-4368312

sgr15 nfu_mirna_prediction mature_miRNA 4368282 4368302 . + . ID=nfu_pred_640;biotype=3'-mature-miRNA;gene=;geneid=nfu_pred_640_;coords=sgr15:4368282-4368302

sgr15 nfu_mirna_prediction pre_miRNA 4447885 4447974 . + . ID=nfu_pred_641;biotype=pre-miRNA;gene=mir-586;geneid=nfu_pred_641_mir-586;coords=sgr15:4447885-4447974

sgr15 nfu_mirna_prediction pre_miRNA 4468029 4468115 . + . ID=nfu_pred_642;biotype=pre-miRNA;gene=mir-942;geneid=nfu_pred_642_mir-942;coords=sgr15:4468029-4468115

sgr15 nfu_mirna_prediction pre_miRNA 4632006 4632118 . + . ID=nfu_pred_643;biotype=pre-miRNA;gene=mir-458;geneid=nfu_pred_643_mir-458;coords=sgr15:4632006-4632118

sgr15 nfu_mirna_prediction pre_miRNA 10937149 10937252 . + . ID=nfu_pred_644;biotype=pre-miRNA;gene=mir-456;geneid=nfu_pred_644_mir-456;coords=sgr15:10937149-10937252

sgr15 nfu_mirna_prediction mature_miRNA 10937211 10937231 . + . ID=nfu_pred_644;biotype=3'-mature-miRNA;gene=mir-456;geneid=nfu_pred_644_mir-456;coords=sgr15:10937211-10937231

sgr15 nfu_mirna_prediction pre_miRNA 13535492 13535592 . + . ID=nfu_pred_645;biotype=pre-miRNA;gene=mir-146;geneid=nfu_pred_645_mir-146;coords=sgr15:13535492-13535592

sgr15 nfu_mirna_prediction mature_miRNA 13535510 13535531 . + . ID=nfu_pred_645;biotype=5'-mature-miRNA;gene=mir-146;geneid=nfu_pred_645_mir-146;coords=sgr15:13535510-13535531

sgr15 nfu_mirna_prediction mature_miRNA 13535550 13535570 . + . ID=nfu_pred_645;biotype=3'-mature-miRNA;gene=mir-146;geneid=nfu_pred_645_mir-146;coords=sgr15:13535550-13535570

sgr15 nfu_mirna_prediction pre_miRNA 16574755 16574847 . + . ID=nfu_pred_646;biotype=pre-miRNA;gene=mir-216;geneid=nfu_pred_646_mir-216;coords=sgr15:16574755-16574847

sgr15 nfu_mirna_prediction mature_miRNA 16574770 16574791 . + . ID=nfu_pred_646;biotype=5'-mature-miRNA;gene=mir-216;geneid=nfu_pred_646_mir-216;coords=sgr15:16574770-16574791

sgr15 nfu_mirna_prediction mature_miRNA 16574809 16574830 . + . ID=nfu_pred_646;biotype=3'-mature-miRNA;gene=mir-216;geneid=nfu_pred_646_mir-216;coords=sgr15:16574809-16574830

sgr15 nfu_mirna_prediction pre_miRNA 16575350 16575438 . + . ID=nfu_pred_647;biotype=pre-miRNA;gene=mir-216;geneid=nfu_pred_647_mir-216;coords=sgr15:16575350-16575438

sgr15 nfu_mirna_prediction mature_miRNA 16575365 16575385 . + . ID=nfu_pred_647;biotype=5'-mature-miRNA;gene=mir-216;geneid=nfu_pred_647_mir-216;coords=sgr15:16575365-16575385

sgr15 nfu_mirna_prediction mature_miRNA 16575405 16575426 . + . ID=nfu_pred_647;biotype=3'-mature-miRNA;gene=mir-216;geneid=nfu_pred_647_mir-216;coords=sgr15:16575405-16575426

sgr15 nfu_mirna_prediction pre_miRNA 16576360 16576467 . + . ID=nfu_pred_648;biotype=pre-miRNA;gene=mir-217;geneid=nfu_pred_648_mir-217;coords=sgr15:16576360-16576467

sgr15 nfu_mirna_prediction mature_miRNA 16576392 16576414 . + . ID=nfu_pred_648;biotype=5'-mature-miRNA;gene=mir-217;geneid=nfu_pred_648_mir-217;coords=sgr15:16576392-16576414

sgr15 nfu_mirna_prediction mature_miRNA 16576429 16576450 . + . ID=nfu_pred_648;biotype=3'-mature-miRNA;gene=mir-217;geneid=nfu_pred_648_mir-217;coords=sgr15:16576429-16576450

sgr15 nfu_mirna_prediction pre_miRNA 20421378 20421475 . + . ID=nfu_pred_649;biotype=pre-miRNA;gene=mir-6720;geneid=nfu_pred_649_mir-6720;coords=sgr15:20421378-20421475

sgr15 nfu_mirna_prediction pre_miRNA 23820174 23820272 . + . ID=nfu_pred_650;biotype=pre-miRNA;gene=mir-202;geneid=nfu_pred_650_mir-202;coords=sgr15:23820174-23820272

sgr15 nfu_mirna_prediction mature_miRNA 23820192 23820212 . + . ID=nfu_pred_650;biotype=5'-mature-miRNA;gene=mir-202;geneid=nfu_pred_650_mir-202;coords=sgr15:23820192-23820212

sgr15 nfu_mirna_prediction pre_miRNA 27348903 27349014 . - . ID=nfu_pred_651;biotype=pre-miRNA;gene=mir-2184;geneid=nfu_pred_651_mir-2184;coords=sgr15:27348903-27349014

sgr15 nfu_mirna_prediction pre_miRNA 27534719 27534803 . - . ID=nfu_pred_652;biotype=pre-miRNA;gene=mir-471;geneid=nfu_pred_652_mir-471;coords=sgr15:27534719-27534803

sgr15 nfu_mirna_prediction mature_miRNA 27534771 27534792 . - . ID=nfu_pred_652;biotype=5'-mature-miRNA;gene=mir-471;geneid=nfu_pred_652_mir-471;coords=sgr15:27534771-27534792

sgr15 nfu_mirna_prediction mature_miRNA 27534733 27534754 . - . ID=nfu_pred_652;biotype=3'-mature-miRNA;gene=mir-471;geneid=nfu_pred_652_mir-471;coords=sgr15:27534733-27534754

sgr15 nfu_mirna_prediction pre_miRNA 28070537 28070650 . - . ID=nfu_pred_653;biotype=pre-miRNA;gene=mir-458;geneid=nfu_pred_653_mir-458;coords=sgr15:28070537-28070650

sgr15 nfu_mirna_prediction pre_miRNA 28537090 28537167 . - . ID=nfu_pred_654;biotype=pre-miRNA;gene=mir-103;geneid=nfu_pred_654_mir-103;coords=sgr15:28537090-28537167

sgr15 nfu_mirna_prediction mature_miRNA 28537100 28537120 . - . ID=nfu_pred_654;biotype=3'-mature-miRNA;gene=mir-103;geneid=nfu_pred_654_mir-103;coords=sgr15:28537100-28537120

sgr15 nfu_mirna_prediction pre_miRNA 29878044 29878124 . + . ID=nfu_pred_655;biotype=pre-miRNA;gene=mir-10;geneid=nfu_pred_655_mir-10;coords=sgr15:29878044-29878124

sgr15 nfu_mirna_prediction mature_miRNA 29878054 29878075 . + . ID=nfu_pred_655;biotype=5'-mature-miRNA;gene=mir-10;geneid=nfu_pred_655_mir-10;coords=sgr15:29878054-29878075

sgr15 nfu_mirna_prediction pre_miRNA 31812617 31812710 . - . ID=nfu_pred_656;biotype=pre-miRNA;gene=mir-218;geneid=nfu_pred_656_mir-218;coords=sgr15:31812617-31812710

sgr15 nfu_mirna_prediction mature_miRNA 31812674 31812694 . - . ID=nfu_pred_656;biotype=5'-mature-miRNA;gene=mir-218;geneid=nfu_pred_656_mir-218;coords=sgr15:31812674-31812694

sgr15 nfu_mirna_prediction pre_miRNA 32891923 32892026 . + . ID=nfu_pred_657;biotype=pre-miRNA;gene=mir-374;geneid=nfu_pred_657_mir-374;coords=sgr15:32891923-32892026

sgr15 nfu_mirna_prediction mature_miRNA 32891944 32891965 . + . ID=nfu_pred_657;biotype=5'-mature-miRNA;gene=mir-374;geneid=nfu_pred_657_mir-374;coords=sgr15:32891944-32891965

sgr15 nfu_mirna_prediction mature_miRNA 32891983 32892003 . + . ID=nfu_pred_657;biotype=3'-mature-miRNA;gene=mir-374;geneid=nfu_pred_657_mir-374;coords=sgr15:32891983-32892003

sgr15 nfu_mirna_prediction pre_miRNA 34529176 34529253 . - . ID=nfu_pred_658;biotype=pre-miRNA;gene=mir-1444;geneid=nfu_pred_658_mir-1444;coords=sgr15:34529176-34529253

sgr15 nfu_mirna_prediction pre_miRNA 41172350 41172444 . - . ID=nfu_pred_659;biotype=pre-miRNA;gene=mir-573;geneid=nfu_pred_659_mir-573;coords=sgr15:41172350-41172444

sgr15 nfu_mirna_prediction mature_miRNA 41172371 41172392 . - . ID=nfu_pred_659;biotype=3'-mature-miRNA;gene=mir-573;geneid=nfu_pred_659_mir-573;coords=sgr15:41172371-41172392

sgr15 nfu_mirna_prediction pre_miRNA 43800668 43800762 . + . ID=nfu_pred_660;biotype=pre-miRNA;gene=mir-811;geneid=nfu_pred_660_mir-811;coords=sgr15:43800668-43800762

sgr15 nfu_mirna_prediction mature_miRNA 43800689 43800710 . + . ID=nfu_pred_660;biotype=5'-mature-miRNA;gene=mir-811;geneid=nfu_pred_660_mir-811;coords=sgr15:43800689-43800710

sgr15 nfu_mirna_prediction mature_miRNA 43800722 43800743 . + . ID=nfu_pred_660;biotype=3'-mature-miRNA;gene=mir-811;geneid=nfu_pred_660_mir-811;coords=sgr15:43800722-43800743

sgr15 nfu_mirna_prediction pre_miRNA 43819830 43819954 . - . ID=nfu_pred_661;biotype=pre-miRNA;gene=mir-458;geneid=nfu_pred_661_mir-458;coords=sgr15:43819830-43819954

sgr15 nfu_mirna_prediction mature_miRNA 43819853 43819874 . - . ID=nfu_pred_661;biotype=3'-mature-miRNA;gene=mir-458;geneid=nfu_pred_661_mir-458;coords=sgr15:43819853-43819874

sgr15 nfu_mirna_prediction pre_miRNA 44153638 44153720 . - . ID=nfu_pred_662;biotype=pre-miRNA;gene=mir-122;geneid=nfu_pred_662_mir-122;coords=sgr15:44153638-44153720

sgr15 nfu_mirna_prediction mature_miRNA 44153691 44153712 . - . ID=nfu_pred_662;biotype=5'-mature-miRNA;gene=mir-122;geneid=nfu_pred_662_mir-122;coords=sgr15:44153691-44153712

sgr15 nfu_mirna_prediction mature_miRNA 44153653 44153673 . - . ID=nfu_pred_662;biotype=3'-mature-miRNA;gene=mir-122;geneid=nfu_pred_662_mir-122;coords=sgr15:44153653-44153673

sgr15 nfu_mirna_prediction pre_miRNA 47579553 47579634 . + . ID=nfu_pred_663;biotype=pre-miRNA;gene=mir-328;geneid=nfu_pred_663_mir-328;coords=sgr15:47579553-47579634

sgr15 nfu_mirna_prediction mature_miRNA 47579568 47579588 . + . ID=nfu_pred_663;biotype=5'-mature-miRNA;gene=mir-328;geneid=nfu_pred_663_mir-328;coords=sgr15:47579568-47579588

sgr15 nfu_mirna_prediction mature_miRNA 47579599 47579619 . + . ID=nfu_pred_663;biotype=3'-mature-miRNA;gene=mir-328;geneid=nfu_pred_663_mir-328;coords=sgr15:47579599-47579619

sgr15 nfu_mirna_prediction pre_miRNA 50361299 50361399 . - . ID=nfu_pred_664;biotype=pre-miRNA;gene=mir-7132;geneid=nfu_pred_664_mir-7132;coords=sgr15:50361299-50361399

sgr15 nfu_mirna_prediction mature_miRNA 50361357 50361377 . - . ID=nfu_pred_664;biotype=5'-mature-miRNA;gene=mir-7132;geneid=nfu_pred_664_mir-7132;coords=sgr15:50361357-50361377

sgr15 nfu_mirna_prediction pre_miRNA 53071504 53071625 . - . ID=nfu_pred_665;biotype=pre-miRNA;gene=mir-455;geneid=nfu_pred_665_mir-455;coords=sgr15:53071504-53071625

sgr15 nfu_mirna_prediction pre_miRNA 54542740 54542824 . + . ID=nfu_pred_666;biotype=pre-miRNA;gene=;geneid=nfu_pred_666_;coords=sgr15:54542740-54542824

sgr15 nfu_mirna_prediction mature_miRNA 54542743 54542761 . + . ID=nfu_pred_666;biotype=5'-mature-miRNA;gene=;geneid=nfu_pred_666_;coords=sgr15:54542743-54542761

sgr15 nfu_mirna_prediction mature_miRNA 54542793 54542814 . + . ID=nfu_pred_666;biotype=3'-mature-miRNA;gene=;geneid=nfu_pred_666_;coords=sgr15:54542793-54542814

sgr15 nfu_mirna_prediction pre_miRNA 54578035 54578129 . - . ID=nfu_pred_667;biotype=pre-miRNA;gene=mir-27;geneid=nfu_pred_667_mir-27;coords=sgr15:54578035-54578129

sgr15 nfu_mirna_prediction mature_miRNA 54578088 54578110 . - . ID=nfu_pred_667;biotype=5'-mature-miRNA;gene=mir-27;geneid=nfu_pred_667_mir-27;coords=sgr15:54578088-54578110

sgr15 nfu_mirna_prediction mature_miRNA 54578051 54578070 . - . ID=nfu_pred_667;biotype=3'-mature-miRNA;gene=mir-27;geneid=nfu_pred_667_mir-27;coords=sgr15:54578051-54578070

sgr15 nfu_mirna_prediction pre_miRNA 54578205 54578303 . - . ID=nfu_pred_668;biotype=pre-miRNA;gene=mir-23;geneid=nfu_pred_668_mir-23;coords=sgr15:54578205-54578303

sgr15 nfu_mirna_prediction mature_miRNA 54578225 54578245 . - . ID=nfu_pred_668;biotype=3'-mature-miRNA;gene=mir-23;geneid=nfu_pred_668_mir-23;coords=sgr15:54578225-54578245

sgr15 nfu_mirna_prediction pre_miRNA 56795444 56795534 . - . ID=nfu_pred_669;biotype=pre-miRNA;gene=mir-33;geneid=nfu_pred_669_mir-33;coords=sgr15:56795444-56795534

sgr15 nfu_mirna_prediction mature_miRNA 56795499 56795519 . - . ID=nfu_pred_669;biotype=5'-mature-miRNA;gene=mir-33;geneid=nfu_pred_669_mir-33;coords=sgr15:56795499-56795519

sgr15 nfu_mirna_prediction mature_miRNA 56795458 56795478 . - . ID=nfu_pred_669;biotype=3'-mature-miRNA;gene=mir-33;geneid=nfu_pred_669_mir-33;coords=sgr15:56795458-56795478

sgr15 nfu_mirna_prediction pre_miRNA 57267305 57267405 . + . ID=nfu_pred_670;biotype=pre-miRNA;gene=mir-455;geneid=nfu_pred_670_mir-455;coords=sgr15:57267305-57267405

sgr15 nfu_mirna_prediction mature_miRNA 57267327 57267348 . + . ID=nfu_pred_670;biotype=5'-mature-miRNA;gene=mir-455;geneid=nfu_pred_670_mir-455;coords=sgr15:57267327-57267348

sgr15 nfu_mirna_prediction mature_miRNA 57267364 57267385 . + . ID=nfu_pred_670;biotype=3'-mature-miRNA;gene=mir-455;geneid=nfu_pred_670_mir-455;coords=sgr15:57267364-57267385

sgr15 nfu_mirna_prediction pre_miRNA 58239308 58239388 . - . ID=nfu_pred_671;biotype=pre-miRNA;gene=mir-396;geneid=nfu_pred_671_mir-396;coords=sgr15:58239308-58239388

sgr15 nfu_mirna_prediction mature_miRNA 58239361 58239382 . - . ID=nfu_pred_671;biotype=5'-mature-miRNA;gene=mir-396;geneid=nfu_pred_671_mir-396;coords=sgr15:58239361-58239382

sgr15 nfu_mirna_prediction pre_miRNA 64276135 64276220 . + . ID=nfu_pred_672;biotype=pre-miRNA;gene=mir-577;geneid=nfu_pred_672_mir-577;coords=sgr15:64276135-64276220

sgr15 nfu_mirna_prediction mature_miRNA 64276139 64276160 . + . ID=nfu_pred_672;biotype=5'-mature-miRNA;gene=mir-577;geneid=nfu_pred_672_mir-577;coords=sgr15:64276139-64276160

sgr15 nfu_mirna_prediction mature_miRNA 64276186 64276206 . + . ID=nfu_pred_672;biotype=3'-mature-miRNA;gene=mir-577;geneid=nfu_pred_672_mir-577;coords=sgr15:64276186-64276206

sgr15 nfu_mirna_prediction pre_miRNA 64955302 64955383 . - . ID=nfu_pred_673;biotype=pre-miRNA;gene=mir-942;geneid=nfu_pred_673_mir-942;coords=sgr15:64955302-64955383

sgr15 nfu_mirna_prediction mature_miRNA 64955351 64955372 . - . ID=nfu_pred_673;biotype=5'-mature-miRNA;gene=mir-942;geneid=nfu_pred_673_mir-942;coords=sgr15:64955351-64955372

sgr15 nfu_mirna_prediction mature_miRNA 64955313 64955334 . - . ID=nfu_pred_673;biotype=3'-mature-miRNA;gene=mir-942;geneid=nfu_pred_673_mir-942;coords=sgr15:64955313-64955334

sgr15 nfu_mirna_prediction pre_miRNA 67223106 67223162 . + . ID=nfu_pred_674;biotype=pre-miRNA;gene=mir-7641;geneid=nfu_pred_674_mir-7641;coords=sgr15:67223106-67223162

sgr15 nfu_mirna_prediction pre_miRNA 69804995 69805084 . - . ID=nfu_pred_675;biotype=pre-miRNA;gene=mir-33;geneid=nfu_pred_675_mir-33;coords=sgr15:69804995-69805084

sgr15 nfu_mirna_prediction mature_miRNA 69805048 69805068 . - . ID=nfu_pred_675;biotype=5'-mature-miRNA;gene=mir-33;geneid=nfu_pred_675_mir-33;coords=sgr15:69805048-69805068

sgr15 nfu_mirna_prediction mature_miRNA 69805010 69805030 . - . ID=nfu_pred_675;biotype=3'-mature-miRNA;gene=mir-33;geneid=nfu_pred_675_mir-33;coords=sgr15:69805010-69805030

sgr15 nfu_mirna_prediction pre_miRNA 71180875 71180972 . + . ID=nfu_pred_676;biotype=pre-miRNA;gene=mir-6720;geneid=nfu_pred_676_mir-6720;coords=sgr15:71180875-71180972

sgr15 nfu_mirna_prediction pre_miRNA 73684502 73684617 . - . ID=nfu_pred_678;biotype=pre-miRNA;gene=mir-8094;geneid=nfu_pred_678_mir-8094;coords=sgr15:73684502-73684617

sgr16 nfu_mirna_prediction pre_miRNA 279147 279225 . - . ID=nfu_pred_679;biotype=pre-miRNA;gene=mir-26;geneid=nfu_pred_679_mir-26;coords=sgr16:279147-279225

sgr16 nfu_mirna_prediction mature_miRNA 279194 279215 . - . ID=nfu_pred_679;biotype=5'-mature-miRNA;gene=mir-26;geneid=nfu_pred_679_mir-26;coords=sgr16:279194-279215

sgr16 nfu_mirna_prediction pre_miRNA 4353796 4353888 . + . ID=nfu_pred_680;biotype=pre-miRNA;gene=mir-228;geneid=nfu_pred_680_mir-228;coords=sgr16:4353796-4353888

sgr16 nfu_mirna_prediction mature_miRNA 4353815 4353836 . + . ID=nfu_pred_680;biotype=5'-mature-miRNA;gene=mir-228;geneid=nfu_pred_680_mir-228;coords=sgr16:4353815-4353836

sgr16 nfu_mirna_prediction mature_miRNA 4353854 4353875 . + . ID=nfu_pred_680;biotype=3'-mature-miRNA;gene=mir-228;geneid=nfu_pred_680_mir-228;coords=sgr16:4353854-4353875

sgr16 nfu_mirna_prediction pre_miRNA 10107324 10107433 . + . ID=nfu_pred_681;biotype=pre-miRNA;gene=mir-338;geneid=nfu_pred_681_mir-338;coords=sgr16:10107324-10107433

sgr16 nfu_mirna_prediction pre_miRNA 15491774 15491825 . - . ID=nfu_pred_682;biotype=pre-miRNA;gene=mir-7399;geneid=nfu_pred_682_mir-7399;coords=sgr16:15491774-15491825

sgr16 nfu_mirna_prediction pre_miRNA 15492600 15492711 . + . ID=nfu_pred_683;biotype=pre-miRNA;gene=mir-7133;geneid=nfu_pred_683_mir-7133;coords=sgr16:15492600-15492711

sgr16 nfu_mirna_prediction pre_miRNA 15572904 15572987 . - . ID=nfu_pred_684;biotype=pre-miRNA;gene=mir-934;geneid=nfu_pred_684_mir-934;coords=sgr16:15572904-15572987

sgr16 nfu_mirna_prediction mature_miRNA 15572955 15572974 . - . ID=nfu_pred_684;biotype=5'-mature-miRNA;gene=mir-934;geneid=nfu_pred_684_mir-934;coords=sgr16:15572955-15572974

sgr16 nfu_mirna_prediction mature_miRNA 15572916 15572936 . - . ID=nfu_pred_684;biotype=3'-mature-miRNA;gene=mir-934;geneid=nfu_pred_684_mir-934;coords=sgr16:15572916-15572936

sgr16 nfu_mirna_prediction pre_miRNA 16705229 16705322 . + . ID=nfu_pred_820;biotype=pre-miRNA;gene=mir-150;geneid=nfu_pred_820_mir-150;coords=sgr16:16705229-16705322

sgr16 nfu_mirna_prediction pre_miRNA 18925920 18926000 . - . ID=nfu_pred_685;biotype=pre-miRNA;gene=mir-4683;geneid=nfu_pred_685_mir-4683;coords=sgr16:18925920-18926000

sgr16 nfu_mirna_prediction pre_miRNA 22832801 22832877 . + . ID=nfu_pred_686;biotype=pre-miRNA;gene=mir-15;geneid=nfu_pred_686_mir-15;coords=sgr16:22832801-22832877

sgr16 nfu_mirna_prediction mature_miRNA 22832811 22832831 . + . ID=nfu_pred_686;biotype=5'-mature-miRNA;gene=mir-15;geneid=nfu_pred_686_mir-15;coords=sgr16:22832811-22832831

sgr16 nfu_mirna_prediction pre_miRNA 22832964 22833042 . + . ID=nfu_pred_687;biotype=pre-miRNA;gene=mir-16;geneid=nfu_pred_687_mir-16;coords=sgr16:22832964-22833042

sgr16 nfu_mirna_prediction mature_miRNA 22832974 22832995 . + . ID=nfu_pred_687;biotype=5'-mature-miRNA;gene=mir-16;geneid=nfu_pred_687_mir-16;coords=sgr16:22832974-22832995

sgr16 nfu_mirna_prediction mature_miRNA 22833014 22833035 . + . ID=nfu_pred_687;biotype=3'-mature-miRNA;gene=mir-16;geneid=nfu_pred_687_mir-16;coords=sgr16:22833014-22833035

sgr16 nfu_mirna_prediction pre_miRNA 23855680 23855758 . - . ID=nfu_pred_688;biotype=pre-miRNA;gene=;geneid=nfu_pred_688_;coords=sgr16:23855680-23855758

sgr16 nfu_mirna_prediction mature_miRNA 23855727 23855747 . - . ID=nfu_pred_688;biotype=5'-mature-miRNA;gene=;geneid=nfu_pred_688_;coords=sgr16:23855727-23855747

sgr16 nfu_mirna_prediction mature_miRNA 23855691 23855711 . - . ID=nfu_pred_688;biotype=3'-mature-miRNA;gene=;geneid=nfu_pred_688_;coords=sgr16:23855691-23855711

sgr16 nfu_mirna_prediction pre_miRNA 26994252 26994332 . + . ID=nfu_pred_689;biotype=pre-miRNA;gene=mir-17;geneid=nfu_pred_689_mir-17;coords=sgr16:26994252-26994332

sgr16 nfu_mirna_prediction mature_miRNA 26994262 26994282 . + . ID=nfu_pred_689;biotype=5'-mature-miRNA;gene=mir-17;geneid=nfu_pred_689_mir-17;coords=sgr16:26994262-26994282

sgr16 nfu_mirna_prediction mature_miRNA 26994299 26994320 . + . ID=nfu_pred_689;biotype=3'-mature-miRNA;gene=mir-17;geneid=nfu_pred_689_mir-17;coords=sgr16:26994299-26994320

sgr16 nfu_mirna_prediction pre_miRNA 26994545 26994621 . + . ID=nfu_pred_690;biotype=pre-miRNA;gene=mir-17;geneid=nfu_pred_690_mir-17;coords=sgr16:26994545-26994621

sgr16 nfu_mirna_prediction mature_miRNA 26994555 26994575 . + . ID=nfu_pred_690;biotype=5'-mature-miRNA;gene=mir-17;geneid=nfu_pred_690_mir-17;coords=sgr16:26994555-26994575

sgr16 nfu_mirna_prediction mature_miRNA 26994596 26994616 . + . ID=nfu_pred_690;biotype=3'-mature-miRNA;gene=mir-17;geneid=nfu_pred_690_mir-17;coords=sgr16:26994596-26994616

sgr16 nfu_mirna_prediction pre_miRNA 26994687 26994765 . + . ID=nfu_pred_691;biotype=pre-miRNA;gene=mir-19;geneid=nfu_pred_691_mir-19;coords=sgr16:26994687-26994765

sgr16 nfu_mirna_prediction mature_miRNA 26994697 26994718 . + . ID=nfu_pred_691;biotype=5'-mature-miRNA;gene=mir-19;geneid=nfu_pred_691_mir-19;coords=sgr16:26994697-26994718

sgr16 nfu_mirna_prediction mature_miRNA 26994734 26994756 . + . ID=nfu_pred_691;biotype=3'-mature-miRNA;gene=mir-19;geneid=nfu_pred_691_mir-19;coords=sgr16:26994734-26994756

sgr16 nfu_mirna_prediction pre_miRNA 26994820 26994900 . + . ID=nfu_pred_692;biotype=pre-miRNA;gene=mir-17;geneid=nfu_pred_692_mir-17;coords=sgr16:26994820-26994900

sgr16 nfu_mirna_prediction mature_miRNA 26994830 26994852 . + . ID=nfu_pred_692;biotype=5'-mature-miRNA;gene=mir-17;geneid=nfu_pred_692_mir-17;coords=sgr16:26994830-26994852

sgr16 nfu_mirna_prediction mature_miRNA 26994867 26994889 . + . ID=nfu_pred_692;biotype=3'-mature-miRNA;gene=mir-17;geneid=nfu_pred_692_mir-17;coords=sgr16:26994867-26994889

sgr16 nfu_mirna_prediction pre_miRNA 26994945 26995023 . + . ID=nfu_pred_693;biotype=pre-miRNA;gene=mir-19;geneid=nfu_pred_693_mir-19;coords=sgr16:26994945-26995023

sgr16 nfu_mirna_prediction mature_miRNA 26994954 26994976 . + . ID=nfu_pred_693;biotype=5'-mature-miRNA;gene=mir-19;geneid=nfu_pred_693_mir-19;coords=sgr16:26994954-26994976

sgr16 nfu_mirna_prediction mature_miRNA 26994992 26995013 . + . ID=nfu_pred_693;biotype=3'-mature-miRNA;gene=mir-19;geneid=nfu_pred_693_mir-19;coords=sgr16:26994992-26995013

sgr16 nfu_mirna_prediction pre_miRNA 26995050 26995128 . + . ID=nfu_pred_694;biotype=pre-miRNA;gene=mir-92;geneid=nfu_pred_694_mir-92;coords=sgr16:26995050-26995128

sgr16 nfu_mirna_prediction mature_miRNA 26995097 26995117 . + . ID=nfu_pred_694;biotype=3'-mature-miRNA;gene=mir-92;geneid=nfu_pred_694_mir-92;coords=sgr16:26995097-26995117

sgr16 nfu_mirna_prediction pre_miRNA 28133964 28134054 . - . ID=nfu_pred_695;biotype=pre-miRNA;gene=mir-148;geneid=nfu_pred_695_mir-148;coords=sgr16:28133964-28134054

sgr16 nfu_mirna_prediction mature_miRNA 28134015 28134037 . - . ID=nfu_pred_695;biotype=5'-mature-miRNA;gene=mir-148;geneid=nfu_pred_695_mir-148;coords=sgr16:28134015-28134037

sgr16 nfu_mirna_prediction mature_miRNA 28133980 28134001 . - . ID=nfu_pred_695;biotype=3'-mature-miRNA;gene=mir-148;geneid=nfu_pred_695_mir-148;coords=sgr16:28133980-28134001

sgr16 nfu_mirna_prediction pre_miRNA 28339320 28339398 . - . ID=nfu_pred_696;biotype=pre-miRNA;gene=mir-10;geneid=nfu_pred_696_mir-10;coords=sgr16:28339320-28339398

sgr16 nfu_mirna_prediction mature_miRNA 28339367 28339388 . - . ID=nfu_pred_696;biotype=5'-mature-miRNA;gene=mir-10;geneid=nfu_pred_696_mir-10;coords=sgr16:28339367-28339388

sgr16 nfu_mirna_prediction pre_miRNA 32786377 32786485 . - . ID=nfu_pred_697;biotype=pre-miRNA;gene=mir-360;geneid=nfu_pred_697_mir-360;coords=sgr16:32786377-32786485

sgr16 nfu_mirna_prediction mature_miRNA 32786438 32786458 . - . ID=nfu_pred_697;biotype=5'-mature-miRNA;gene=mir-360;geneid=nfu_pred_697_mir-360;coords=sgr16:32786438-32786458

sgr16 nfu_mirna_prediction mature_miRNA 32786400 32786422 . - . ID=nfu_pred_697;biotype=3'-mature-miRNA;gene=mir-360;geneid=nfu_pred_697_mir-360;coords=sgr16:32786400-32786422

sgr16 nfu_mirna_prediction pre_miRNA 34288667 34288748 . - . ID=nfu_pred_698;biotype=pre-miRNA;gene=mir-423;geneid=nfu_pred_698_mir-423;coords=sgr16:34288667-34288748

sgr16 nfu_mirna_prediction mature_miRNA 34288715 34288737 . - . ID=nfu_pred_698;biotype=5'-mature-miRNA;gene=mir-423;geneid=nfu_pred_698_mir-423;coords=sgr16:34288715-34288737

sgr16 nfu_mirna_prediction mature_miRNA 34288684 34288705 . - . ID=nfu_pred_698;biotype=3'-mature-miRNA;gene=mir-423;geneid=nfu_pred_698_mir-423;coords=sgr16:34288684-34288705

sgr16 nfu_mirna_prediction pre_miRNA 35270388 35270473 . - . ID=nfu_pred_699;biotype=pre-miRNA;gene=mir-153;geneid=nfu_pred_699_mir-153;coords=sgr16:35270388-35270473

sgr16 nfu_mirna_prediction mature_miRNA 35270439 35270460 . - . ID=nfu_pred_699;biotype=5'-mature-miRNA;gene=mir-153;geneid=nfu_pred_699_mir-153;coords=sgr16:35270439-35270460

sgr16 nfu_mirna_prediction mature_miRNA 35270401 35270422 . - . ID=nfu_pred_699;biotype=3'-mature-miRNA;gene=mir-153;geneid=nfu_pred_699_mir-153;coords=sgr16:35270401-35270422

sgr16 nfu_mirna_prediction pre_miRNA 36942471 36942549 . + . ID=nfu_pred_700;biotype=pre-miRNA;gene=mir-375;geneid=nfu_pred_700_mir-375;coords=sgr16:36942471-36942549

sgr16 nfu_mirna_prediction mature_miRNA 36942482 36942502 . + . ID=nfu_pred_700;biotype=5'-mature-miRNA;gene=mir-375;geneid=nfu_pred_700_mir-375;coords=sgr16:36942482-36942502

sgr16 nfu_mirna_prediction mature_miRNA 36942518 36942538 . + . ID=nfu_pred_700;biotype=3'-mature-miRNA;gene=mir-375;geneid=nfu_pred_700_mir-375;coords=sgr16:36942518-36942538

sgr16 nfu_mirna_prediction pre_miRNA 36964593 36964685 . + . ID=nfu_pred_701;biotype=pre-miRNA;gene=mir-883;geneid=nfu_pred_701_mir-883;coords=sgr16:36964593-36964685

sgr16 nfu_mirna_prediction mature_miRNA 36964611 36964631 . + . ID=nfu_pred_701;biotype=5'-mature-miRNA;gene=mir-883;geneid=nfu_pred_701_mir-883;coords=sgr16:36964611-36964631

sgr16 nfu_mirna_prediction mature_miRNA 36964648 36964668 . + . ID=nfu_pred_701;biotype=3'-mature-miRNA;gene=mir-883;geneid=nfu_pred_701_mir-883;coords=sgr16:36964648-36964668

sgr16 nfu_mirna_prediction pre_miRNA 40103049 40103129 . - . ID=nfu_pred_702;biotype=pre-miRNA;gene=mir-4683;geneid=nfu_pred_702_mir-4683;coords=sgr16:40103049-40103129

sgr17 nfu_mirna_prediction pre_miRNA 4111312 4111406 . - . ID=nfu_pred_703;biotype=pre-miRNA;gene=mir-563;geneid=nfu_pred_703_mir-563;coords=sgr17:4111312-4111406

sgr17 nfu_mirna_prediction mature_miRNA 4111370 4111394 . - . ID=nfu_pred_703;biotype=5'-mature-miRNA;gene=mir-563;geneid=nfu_pred_703_mir-563;coords=sgr17:4111370-4111394

sgr17 nfu_mirna_prediction pre_miRNA 6677914 6678040 . - . ID=nfu_pred_704;biotype=pre-miRNA;gene=mir-2189;geneid=nfu_pred_704_mir-2189;coords=sgr17:6677914-6678040

sgr17 nfu_mirna_prediction pre_miRNA 10525207 10525289 . + . ID=nfu_pred_705;biotype=pre-miRNA;gene=mir-728;geneid=nfu_pred_705_mir-728;coords=sgr17:10525207-10525289

sgr17 nfu_mirna_prediction pre_miRNA 10691931 10692005 . - . ID=nfu_pred_706;biotype=pre-miRNA;gene=;geneid=nfu_pred_706_;coords=sgr17:10691931-10692005

sgr17 nfu_mirna_prediction pre_miRNA 14862036 14862118 . - . ID=nfu_pred_708;biotype=pre-miRNA;gene=mir-460;geneid=nfu_pred_708_mir-460;coords=sgr17:14862036-14862118

sgr17 nfu_mirna_prediction mature_miRNA 14862087 14862108 . - . ID=nfu_pred_708;biotype=5'-mature-miRNA;gene=mir-460;geneid=nfu_pred_708_mir-460;coords=sgr17:14862087-14862108

sgr17 nfu_mirna_prediction mature_miRNA 14862048 14862069 . - . ID=nfu_pred_708;biotype=3'-mature-miRNA;gene=mir-460;geneid=nfu_pred_708_mir-460;coords=sgr17:14862048-14862069

sgr17 nfu_mirna_prediction pre_miRNA 17200478 17200543 . + . ID=nfu_pred_709;biotype=pre-miRNA;gene=mir-122a/mir-3591;geneid=nfu_pred_709_mir-122a/mir-3591;coords=sgr17:17200478-17200543

sgr17 nfu_mirna_prediction pre_miRNA 19060977 19061059 . + . ID=nfu_pred_710;biotype=pre-miRNA;gene=mir-577;geneid=nfu_pred_710_mir-577;coords=sgr17:19060977-19061059

sgr17 nfu_mirna_prediction mature_miRNA 19060998 19061018 . + . ID=nfu_pred_710;biotype=5'-mature-miRNA;gene=mir-577;geneid=nfu_pred_710_mir-577;coords=sgr17:19060998-19061018

sgr17 nfu_mirna_prediction mature_miRNA 19061023 19061043 . + . ID=nfu_pred_710;biotype=3'-mature-miRNA;gene=mir-577;geneid=nfu_pred_710_mir-577;coords=sgr17:19061023-19061043

sgr17 nfu_mirna_prediction pre_miRNA 19733074 19733167 . + . ID=nfu_pred_711;biotype=pre-miRNA;gene=mir-340;geneid=nfu_pred_711_mir-340;coords=sgr17:19733074-19733167

sgr17 nfu_mirna_prediction mature_miRNA 19733131 19733152 . + . ID=nfu_pred_711;biotype=3'-mature-miRNA;gene=mir-340;geneid=nfu_pred_711_mir-340;coords=sgr17:19733131-19733152

sgr17 nfu_mirna_prediction pre_miRNA 20221532 20221612 . + . ID=nfu_pred_712;biotype=pre-miRNA;gene=mir-939;geneid=nfu_pred_712_mir-939;coords=sgr17:20221532-20221612

sgr17 nfu_mirna_prediction mature_miRNA 20221540 20221561 . + . ID=nfu_pred_712;biotype=5'-mature-miRNA;gene=mir-939;geneid=nfu_pred_712_mir-939;coords=sgr17:20221540-20221561

sgr17 nfu_mirna_prediction mature_miRNA 20221580 20221600 . + . ID=nfu_pred_712;biotype=3'-mature-miRNA;gene=mir-939;geneid=nfu_pred_712_mir-939;coords=sgr17:20221580-20221600

sgr17 nfu_mirna_prediction pre_miRNA 20790604 20790688 . - . ID=nfu_pred_713;biotype=pre-miRNA;gene=mir-31;geneid=nfu_pred_713_mir-31;coords=sgr17:20790604-20790688

sgr17 nfu_mirna_prediction mature_miRNA 20790654 20790675 . - . ID=nfu_pred_713;biotype=5'-mature-miRNA;gene=mir-31;geneid=nfu_pred_713_mir-31;coords=sgr17:20790654-20790675

sgr17 nfu_mirna_prediction mature_miRNA 20790615 20790636 . - . ID=nfu_pred_713;biotype=3'-mature-miRNA;gene=mir-31;geneid=nfu_pred_713_mir-31;coords=sgr17:20790615-20790636

sgr17 nfu_mirna_prediction pre_miRNA 23331806 23331871 . + . ID=nfu_pred_714;biotype=pre-miRNA;gene=mir-451;geneid=nfu_pred_714_mir-451;coords=sgr17:23331806-23331871

sgr17 nfu_mirna_prediction pre_miRNA 24410343 24410406 . + . ID=nfu_pred_715;biotype=pre-miRNA;gene=mir-144;geneid=nfu_pred_715_mir-144;coords=sgr17:24410343-24410406

sgr17 nfu_mirna_prediction pre_miRNA 25047476 25047552 . + . ID=nfu_pred_716;biotype=pre-miRNA;gene=mir-944;geneid=nfu_pred_716_mir-944;coords=sgr17:25047476-25047552

sgr17 nfu_mirna_prediction mature_miRNA 25047486 25047507 . + . ID=nfu_pred_716;biotype=5'-mature-miRNA;gene=mir-944;geneid=nfu_pred_716_mir-944;coords=sgr17:25047486-25047507

sgr17 nfu_mirna_prediction mature_miRNA 25047522 25047543 . + . ID=nfu_pred_716;biotype=3'-mature-miRNA;gene=mir-944;geneid=nfu_pred_716_mir-944;coords=sgr17:25047522-25047543

sgr17 nfu_mirna_prediction pre_miRNA 25615326 25615398 . - . ID=nfu_pred_717;biotype=pre-miRNA;gene=;geneid=nfu_pred_717_;coords=sgr17:25615326-25615398

sgr17 nfu_mirna_prediction mature_miRNA 25615336 25615356 . - . ID=nfu_pred_717;biotype=3'-mature-miRNA;gene=;geneid=nfu_pred_717_;coords=sgr17:25615336-25615356

sgr17 nfu_mirna_prediction pre_miRNA 25863407 25863500 . - . ID=nfu_pred_718;biotype=pre-miRNA;gene=;geneid=nfu_pred_718_;coords=sgr17:25863407-25863500

sgr17 nfu_mirna_prediction mature_miRNA 25863462 25863482 . - . ID=nfu_pred_718;biotype=5'-mature-miRNA;gene=;geneid=nfu_pred_718_;coords=sgr17:25863462-25863482

sgr17 nfu_mirna_prediction mature_miRNA 25863419 25863441 . - . ID=nfu_pred_718;biotype=3'-mature-miRNA;gene=;geneid=nfu_pred_718_;coords=sgr17:25863419-25863441

sgr17 nfu_mirna_prediction pre_miRNA 26180129 26180207 . - . ID=nfu_pred_719;biotype=pre-miRNA;gene=mir-26;geneid=nfu_pred_719_mir-26;coords=sgr17:26180129-26180207

sgr17 nfu_mirna_prediction mature_miRNA 26180176 26180197 . - . ID=nfu_pred_719;biotype=5'-mature-miRNA;gene=mir-26;geneid=nfu_pred_719_mir-26;coords=sgr17:26180176-26180197

sgr17 nfu_mirna_prediction pre_miRNA 33425078 33425180 . - . ID=nfu_pred_720;biotype=pre-miRNA;gene=mir-8915;geneid=nfu_pred_720_mir-8915;coords=sgr17:33425078-33425180

sgr17 nfu_mirna_prediction pre_miRNA 36486101 36486206 . - . ID=nfu_pred_721;biotype=pre-miRNA;gene=mir-607;geneid=nfu_pred_721_mir-607;coords=sgr17:36486101-36486206

sgr17 nfu_mirna_prediction mature_miRNA 36486161 36486182 . - . ID=nfu_pred_721;biotype=5'-mature-miRNA;gene=mir-607;geneid=nfu_pred_721_mir-607;coords=sgr17:36486161-36486182

sgr17 nfu_mirna_prediction mature_miRNA 36486121 36486142 . - . ID=nfu_pred_721;biotype=3'-mature-miRNA;gene=mir-607;geneid=nfu_pred_721_mir-607;coords=sgr17:36486121-36486142

sgr17 nfu_mirna_prediction pre_miRNA 39782248 39782412 . - . ID=nfu_pred_722;biotype=pre-miRNA;gene=mir-811;geneid=nfu_pred_722_mir-811;coords=sgr17:39782248-39782412

sgr17 nfu_mirna_prediction mature_miRNA 39782338 39782358 . - . ID=nfu_pred_722;biotype=5'-mature-miRNA;gene=mir-811;geneid=nfu_pred_722_mir-811;coords=sgr17:39782338-39782358

sgr17 nfu_mirna_prediction mature_miRNA 39782300 39782322 . - . ID=nfu_pred_722;biotype=3'-mature-miRNA;gene=mir-811;geneid=nfu_pred_722_mir-811;coords=sgr17:39782300-39782322

sgr17 nfu_mirna_prediction pre_miRNA 40227588 40227683 . + . ID=nfu_pred_723;biotype=pre-miRNA;gene=mir-1338;geneid=nfu_pred_723_mir-1338;coords=sgr17:40227588-40227683

sgr17 nfu_mirna_prediction pre_miRNA 47994862 47994956 . - . ID=nfu_pred_724;biotype=pre-miRNA;gene=mir-642;geneid=nfu_pred_724_mir-642;coords=sgr17:47994862-47994956

sgr17 nfu_mirna_prediction mature_miRNA 47994923 47994943 . - . ID=nfu_pred_724;biotype=5'-mature-miRNA;gene=mir-642;geneid=nfu_pred_724_mir-642;coords=sgr17:47994923-47994943

sgr17 nfu_mirna_prediction mature_miRNA 47994888 47994908 . - . ID=nfu_pred_724;biotype=3'-mature-miRNA;gene=mir-642;geneid=nfu_pred_724_mir-642;coords=sgr17:47994888-47994908

sgr17 nfu_mirna_prediction pre_miRNA 48814037 48814147 . - . ID=nfu_pred_725;biotype=pre-miRNA;gene=mir-142;geneid=nfu_pred_725_mir-142;coords=sgr17:48814037-48814147

sgr17 nfu_mirna_prediction mature_miRNA 48814099 48814120 . - . ID=nfu_pred_725;biotype=5'-mature-miRNA;gene=mir-142;geneid=nfu_pred_725_mir-142;coords=sgr17:48814099-48814120

sgr17 nfu_mirna_prediction mature_miRNA 48814059 48814081 . - . ID=nfu_pred_725;biotype=3'-mature-miRNA;gene=mir-142;geneid=nfu_pred_725_mir-142;coords=sgr17:48814059-48814081

sgr17 nfu_mirna_prediction pre_miRNA 53110065 53110141 . - . ID=nfu_pred_726;biotype=pre-miRNA;gene=;geneid=nfu_pred_726_;coords=sgr17:53110065-53110141

sgr17 nfu_mirna_prediction pre_miRNA 53200093 53200171 . - . ID=nfu_pred_727;biotype=pre-miRNA;gene=;geneid=nfu_pred_727_;coords=sgr17:53200093-53200171

sgr17 nfu_mirna_prediction mature_miRNA 53200103 53200123 . - . ID=nfu_pred_727;biotype=5'-mature-miRNA;gene=;geneid=nfu_pred_727_;coords=sgr17:53200103-53200123

sgr17 nfu_mirna_prediction mature_miRNA 53200140 53200161 . - . ID=nfu_pred_727;biotype=3'-mature-miRNA;gene=;geneid=nfu_pred_727_;coords=sgr17:53200140-53200161

sgr17 nfu_mirna_prediction pre_miRNA 53207618 53207721 . + . ID=nfu_pred_728;biotype=pre-miRNA;gene=mir-811;geneid=nfu_pred_728_mir-811;coords=sgr17:53207618-53207721

sgr17 nfu_mirna_prediction mature_miRNA 53207648 53207669 . + . ID=nfu_pred_728;biotype=5'-mature-miRNA;gene=mir-811;geneid=nfu_pred_728_mir-811;coords=sgr17:53207648-53207669

sgr17 nfu_mirna_prediction mature_miRNA 53207680 53207701 . + . ID=nfu_pred_728;biotype=3'-mature-miRNA;gene=mir-811;geneid=nfu_pred_728_mir-811;coords=sgr17:53207680-53207701

sgr17 nfu_mirna_prediction pre_miRNA 59325399 59325486 . - . ID=nfu_pred_729;biotype=pre-miRNA;gene=mir-187;geneid=nfu_pred_729_mir-187;coords=sgr17:59325399-59325486

sgr17 nfu_mirna_prediction mature_miRNA 59325411 59325432 . - . ID=nfu_pred_729;biotype=5'-mature-miRNA;gene=mir-187;geneid=nfu_pred_729_mir-187;coords=sgr17:59325411-59325432

sgr17 nfu_mirna_prediction mature_miRNA 59325451 59325471 . - . ID=nfu_pred_729;biotype=3'-mature-miRNA;gene=mir-187;geneid=nfu_pred_729_mir-187;coords=sgr17:59325451-59325471

sgr17 nfu_mirna_prediction pre_miRNA 59399607 59399688 . + . ID=nfu_pred_730;biotype=pre-miRNA;gene=mir-124;geneid=nfu_pred_730_mir-124;coords=sgr17:59399607-59399688

sgr17 nfu_mirna_prediction mature_miRNA 59399618 59399638 . + . ID=nfu_pred_730;biotype=5'-mature-miRNA;gene=mir-124;geneid=nfu_pred_730_mir-124;coords=sgr17:59399618-59399638

sgr17 nfu_mirna_prediction mature_miRNA 59399657 59399677 . + . ID=nfu_pred_730;biotype=3'-mature-miRNA;gene=mir-124;geneid=nfu_pred_730_mir-124;coords=sgr17:59399657-59399677

sgr17 nfu_mirna_prediction pre_miRNA 61154948 61155048 . + . ID=nfu_pred_731;biotype=pre-miRNA;gene=mir-203;geneid=nfu_pred_731_mir-203;coords=sgr17:61154948-61155048

sgr17 nfu_mirna_prediction mature_miRNA 61154970 61154991 . + . ID=nfu_pred_731;biotype=5'-mature-miRNA;gene=mir-203;geneid=nfu_pred_731_mir-203;coords=sgr17:61154970-61154991

sgr17 nfu_mirna_prediction mature_miRNA 61155008 61155028 . + . ID=nfu_pred_731;biotype=3'-mature-miRNA;gene=mir-203;geneid=nfu_pred_731_mir-203;coords=sgr17:61155008-61155028

sgr17 nfu_mirna_prediction pre_miRNA 62591296 62591390 . - . ID=nfu_pred_732;biotype=pre-miRNA;gene=mir-340;geneid=nfu_pred_732_mir-340;coords=sgr17:62591296-62591390

sgr17 nfu_mirna_prediction mature_miRNA 62591311 62591333 . - . ID=nfu_pred_732;biotype=3'-mature-miRNA;gene=mir-340;geneid=nfu_pred_732_mir-340;coords=sgr17:62591311-62591333

sgr17 nfu_mirna_prediction pre_miRNA 63912594 63912735 . + . ID=nfu_pred_733;biotype=pre-miRNA;gene=mir-821;geneid=nfu_pred_733_mir-821;coords=sgr17:63912594-63912735

sgr17 nfu_mirna_prediction mature_miRNA 63912624 63912644 . + . ID=nfu_pred_733;biotype=5'-mature-miRNA;gene=mir-821;geneid=nfu_pred_733_mir-821;coords=sgr17:63912624-63912644

sgr17 nfu_mirna_prediction pre_miRNA 65844696 65844785 . + . ID=nfu_pred_734;biotype=pre-miRNA;gene=mir-430;geneid=nfu_pred_734_mir-430;coords=sgr17:65844696-65844785

sgr17 nfu_mirna_prediction mature_miRNA 65844755 65844776 . + . ID=nfu_pred_734;biotype=3'-mature-miRNA;gene=mir-430;geneid=nfu_pred_734_mir-430;coords=sgr17:65844755-65844776

sgr17 nfu_mirna_prediction pre_miRNA 65845229 65845325 . + . ID=nfu_pred_735;biotype=pre-miRNA;gene=mir-430;geneid=nfu_pred_735_mir-430;coords=sgr17:65845229-65845325

sgr17 nfu_mirna_prediction mature_miRNA 65845285 65845306 . + . ID=nfu_pred_735;biotype=3'-mature-miRNA;gene=mir-430;geneid=nfu_pred_735_mir-430;coords=sgr17:65845285-65845306

sgr17 nfu_mirna_prediction pre_miRNA 66014809 66014866 . + . ID=nfu_pred_736;biotype=pre-miRNA;gene=mir-430;geneid=nfu_pred_736_mir-430;coords=sgr17:66014809-66014866

sgr17 nfu_mirna_prediction pre_miRNA 66014927 66015005 . + . ID=nfu_pred_821;biotype=pre-miRNA;gene=mir-430;geneid=nfu_pred_821_mir-430;coords=sgr17:66014927-66015005

sgr17 nfu_mirna_prediction pre_miRNA 66015050 66015139 . + . ID=nfu_pred_737;biotype=pre-miRNA;gene=mir-430;geneid=nfu_pred_737_mir-430;coords=sgr17:66015050-66015139

sgr17 nfu_mirna_prediction mature_miRNA 66015071 66015091 . + . ID=nfu_pred_737;biotype=5'-mature-miRNA;gene=mir-430;geneid=nfu_pred_737_mir-430;coords=sgr17:66015071-66015091

sgr17 nfu_mirna_prediction mature_miRNA 66015108 66015129 . + . ID=nfu_pred_737;biotype=3'-mature-miRNA;gene=mir-430;geneid=nfu_pred_737_mir-430;coords=sgr17:66015108-66015129

sgr17 nfu_mirna_prediction pre_miRNA 66015353 66015421 . + . ID=nfu_pred_738;biotype=pre-miRNA;gene=mir-430;geneid=nfu_pred_738_mir-430;coords=sgr17:66015353-66015421

sgr17 nfu_mirna_prediction mature_miRNA 66015361 66015380 . + . ID=nfu_pred_738;biotype=5'-mature-miRNA;gene=mir-430;geneid=nfu_pred_738_mir-430;coords=sgr17:66015361-66015380

sgr17 nfu_mirna_prediction mature_miRNA 66015393 66015414 . + . ID=nfu_pred_738;biotype=3'-mature-miRNA;gene=mir-430;geneid=nfu_pred_738_mir-430;coords=sgr17:66015393-66015414

sgr17 nfu_mirna_prediction pre_miRNA 66015447 66015547 . + . ID=nfu_pred_739;biotype=pre-miRNA;gene=mir-430;geneid=nfu_pred_739_mir-430;coords=sgr17:66015447-66015547

sgr17 nfu_mirna_prediction mature_miRNA 66015505 66015525 . + . ID=nfu_pred_739;biotype=3'-mature-miRNA;gene=mir-430;geneid=nfu_pred_739_mir-430;coords=sgr17:66015505-66015525

sgr17 nfu_mirna_prediction pre_miRNA 66015608 66015686 . + . ID=nfu_pred_740;biotype=pre-miRNA;gene=mir-430;geneid=nfu_pred_740_mir-430;coords=sgr17:66015608-66015686

sgr17 nfu_mirna_prediction mature_miRNA 66015655 66015676 . + . ID=nfu_pred_740;biotype=3'-mature-miRNA;gene=mir-430;geneid=nfu_pred_740_mir-430;coords=sgr17:66015655-66015676

sgr17 nfu_mirna_prediction pre_miRNA 66015733 66015823 . + . ID=nfu_pred_741;biotype=pre-miRNA;gene=mir-430;geneid=nfu_pred_741_mir-430;coords=sgr17:66015733-66015823

sgr17 nfu_mirna_prediction mature_miRNA 66015792 66015813 . + . ID=nfu_pred_741;biotype=3'-mature-miRNA;gene=mir-430;geneid=nfu_pred_741_mir-430;coords=sgr17:66015792-66015813

sgr17 nfu_mirna_prediction pre_miRNA 66016253 66016331 . + . ID=nfu_pred_742;biotype=pre-miRNA;gene=mir-430;geneid=nfu_pred_742_mir-430;coords=sgr17:66016253-66016331

sgr17 nfu_mirna_prediction pre_miRNA 66016380 66016470 . + . ID=nfu_pred_743;biotype=pre-miRNA;gene=mir-430;geneid=nfu_pred_743_mir-430;coords=sgr17:66016380-66016470

sgr17 nfu_mirna_prediction mature_miRNA 66016429 66016449 . + . ID=nfu_pred_743;biotype=3'-mature-miRNA;gene=mir-430;geneid=nfu_pred_743_mir-430;coords=sgr17:66016429-66016449

sgr17 nfu_mirna_prediction pre_miRNA 66016506 66016606 . + . ID=nfu_pred_744;biotype=pre-miRNA;gene=mir-430;geneid=nfu_pred_744_mir-430;coords=sgr17:66016506-66016606

sgr17 nfu_mirna_prediction mature_miRNA 66016527 66016547 . + . ID=nfu_pred_744;biotype=5'-mature-miRNA;gene=mir-430;geneid=nfu_pred_744_mir-430;coords=sgr17:66016527-66016547

sgr17 nfu_mirna_prediction mature_miRNA 66016564 66016585 . + . ID=nfu_pred_744;biotype=3'-mature-miRNA;gene=mir-430;geneid=nfu_pred_744_mir-430;coords=sgr17:66016564-66016585

sgr17 nfu_mirna_prediction pre_miRNA 66016809 66016877 . + . ID=nfu_pred_745;biotype=pre-miRNA;gene=mir-430;geneid=nfu_pred_745_mir-430;coords=sgr17:66016809-66016877

sgr17 nfu_mirna_prediction pre_miRNA 66016904 66017004 . + . ID=nfu_pred_746;biotype=pre-miRNA;gene=mir-430;geneid=nfu_pred_746_mir-430;coords=sgr17:66016904-66017004

sgr17 nfu_mirna_prediction mature_miRNA 66016962 66016982 . + . ID=nfu_pred_746;biotype=3'-mature-miRNA;gene=mir-430;geneid=nfu_pred_746_mir-430;coords=sgr17:66016962-66016982

sgr17 nfu_mirna_prediction pre_miRNA 66017065 66017143 . + . ID=nfu_pred_747;biotype=pre-miRNA;gene=mir-430;geneid=nfu_pred_747_mir-430;coords=sgr17:66017065-66017143

sgr17 nfu_mirna_prediction mature_miRNA 66017112 66017133 . + . ID=nfu_pred_747;biotype=3'-mature-miRNA;gene=mir-430;geneid=nfu_pred_747_mir-430;coords=sgr17:66017112-66017133

sgr17 nfu_mirna_prediction pre_miRNA 66017190 66017280 . + . ID=nfu_pred_748;biotype=pre-miRNA;gene=mir-430;geneid=nfu_pred_748_mir-430;coords=sgr17:66017190-66017280

sgr17 nfu_mirna_prediction mature_miRNA 66017249 66017270 . + . ID=nfu_pred_748;biotype=3'-mature-miRNA;gene=mir-430;geneid=nfu_pred_748_mir-430;coords=sgr17:66017249-66017270

sgr17 nfu_mirna_prediction pre_miRNA 66017709 66017787 . + . ID=nfu_pred_749;biotype=pre-miRNA;gene=mir-430;geneid=nfu_pred_749_mir-430;coords=sgr17:66017709-66017787

sgr17 nfu_mirna_prediction pre_miRNA 66017836 66017926 . + . ID=nfu_pred_750;biotype=pre-miRNA;gene=mir-430;geneid=nfu_pred_750_mir-430;coords=sgr17:66017836-66017926

sgr17 nfu_mirna_prediction mature_miRNA 66017885 66017905 . + . ID=nfu_pred_750;biotype=3'-mature-miRNA;gene=mir-430;geneid=nfu_pred_750_mir-430;coords=sgr17:66017885-66017905

sgr17 nfu_mirna_prediction pre_miRNA 66017962 66018062 . + . ID=nfu_pred_751;biotype=pre-miRNA;gene=mir-430;geneid=nfu_pred_751_mir-430;coords=sgr17:66017962-66018062

sgr17 nfu_mirna_prediction mature_miRNA 66018020 66018041 . + . ID=nfu_pred_751;biotype=3'-mature-miRNA;gene=mir-430;geneid=nfu_pred_751_mir-430;coords=sgr17:66018020-66018041

sgr17 nfu_mirna_prediction pre_miRNA 66018258 66018336 . + . ID=nfu_pred_752;biotype=pre-miRNA;gene=mir-430;geneid=nfu_pred_752_mir-430;coords=sgr17:66018258-66018336

sgr17 nfu_mirna_prediction mature_miRNA 66018273 66018292 . + . ID=nfu_pred_752;biotype=5'-mature-miRNA;gene=mir-430;geneid=nfu_pred_752_mir-430;coords=sgr17:66018273-66018292

sgr17 nfu_mirna_prediction mature_miRNA 66018305 66018326 . + . ID=nfu_pred_752;biotype=3'-mature-miRNA;gene=mir-430;geneid=nfu_pred_752_mir-430;coords=sgr17:66018305-66018326

sgr17 nfu_mirna_prediction pre_miRNA 66018359 66018459 . + . ID=nfu_pred_753;biotype=pre-miRNA;gene=mir-430;geneid=nfu_pred_753_mir-430;coords=sgr17:66018359-66018459

sgr17 nfu_mirna_prediction mature_miRNA 66018417 66018437 . + . ID=nfu_pred_753;biotype=3'-mature-miRNA;gene=mir-430;geneid=nfu_pred_753_mir-430;coords=sgr17:66018417-66018437

sgr17 nfu_mirna_prediction pre_miRNA 66018520 66018598 . + . ID=nfu_pred_754;biotype=pre-miRNA;gene=mir-430;geneid=nfu_pred_754_mir-430;coords=sgr17:66018520-66018598

sgr17 nfu_mirna_prediction mature_miRNA 66018567 66018588 . + . ID=nfu_pred_754;biotype=3'-mature-miRNA;gene=mir-430;geneid=nfu_pred_754_mir-430;coords=sgr17:66018567-66018588

sgr17 nfu_mirna_prediction pre_miRNA 66018716 66018806 . + . ID=nfu_pred_755;biotype=pre-miRNA;gene=mir-430;geneid=nfu_pred_755_mir-430;coords=sgr17:66018716-66018806

sgr17 nfu_mirna_prediction mature_miRNA 66018765 66018785 . + . ID=nfu_pred_755;biotype=3'-mature-miRNA;gene=mir-430;geneid=nfu_pred_755_mir-430;coords=sgr17:66018765-66018785

sgr17 nfu_mirna_prediction pre_miRNA 71733845 71733943 . + . ID=nfu_pred_756;biotype=pre-miRNA;gene=mir-BHRF1;geneid=nfu_pred_756_mir-BHRF1;coords=sgr17:71733845-71733943

sgr17 nfu_mirna_prediction mature_miRNA 71733867 71733887 . + . ID=nfu_pred_756;biotype=5'-mature-miRNA;gene=mir-BHRF1;geneid=nfu_pred_756_mir-BHRF1;coords=sgr17:71733867-71733887

sgr17 nfu_mirna_prediction mature_miRNA 71733902 71733923 . + . ID=nfu_pred_756;biotype=3'-mature-miRNA;gene=mir-BHRF1;geneid=nfu_pred_756_mir-BHRF1;coords=sgr17:71733902-71733923

sgr17 nfu_mirna_prediction pre_miRNA 71738259 71738350 . + . ID=nfu_pred_757;biotype=pre-miRNA;gene=mir-558;geneid=nfu_pred_757_mir-558;coords=sgr17:71738259-71738350

sgr17 nfu_mirna_prediction mature_miRNA 71738277 71738299 . + . ID=nfu_pred_757;biotype=5'-mature-miRNA;gene=mir-558;geneid=nfu_pred_757_mir-558;coords=sgr17:71738277-71738299

sgr18 nfu_mirna_prediction pre_miRNA 2136097 2136175 . + . ID=nfu_pred_758;biotype=pre-miRNA;gene=;geneid=nfu_pred_758_;coords=sgr18:2136097-2136175

sgr18 nfu_mirna_prediction mature_miRNA 2136144 2136164 . + . ID=nfu_pred_758;biotype=3'-mature-miRNA;gene=;geneid=nfu_pred_758_;coords=sgr18:2136144-2136164

sgr18 nfu_mirna_prediction pre_miRNA 2662282 2662338 . - . ID=nfu_pred_759;biotype=pre-miRNA;gene=mir-7641;geneid=nfu_pred_759_mir-7641;coords=sgr18:2662282-2662338

sgr18 nfu_mirna_prediction pre_miRNA 5424819 5424879 . - . ID=nfu_pred_761;biotype=pre-miRNA;gene=mir-722;geneid=nfu_pred_761_mir-722;coords=sgr18:5424819-5424879

sgr18 nfu_mirna_prediction pre_miRNA 5502789 5502918 . + . ID=nfu_pred_762;biotype=pre-miRNA;gene=mir-458;geneid=nfu_pred_762_mir-458;coords=sgr18:5502789-5502918

sgr18 nfu_mirna_prediction pre_miRNA 8767533 8767625 . + . ID=nfu_pred_763;biotype=pre-miRNA;gene=mir-345;geneid=nfu_pred_763_mir-345;coords=sgr18:8767533-8767625

sgr18 nfu_mirna_prediction mature_miRNA 8767562 8767582 . + . ID=nfu_pred_763;biotype=5'-mature-miRNA;gene=mir-345;geneid=nfu_pred_763_mir-345;coords=sgr18:8767562-8767582

sgr18 nfu_mirna_prediction mature_miRNA 8767594 8767614 . + . ID=nfu_pred_763;biotype=3'-mature-miRNA;gene=mir-345;geneid=nfu_pred_763_mir-345;coords=sgr18:8767594-8767614

sgr18 nfu_mirna_prediction pre_miRNA 9294094 9294188 . + . ID=nfu_pred_764;biotype=pre-miRNA;gene=mir-338;geneid=nfu_pred_764_mir-338;coords=sgr18:9294094-9294188

sgr18 nfu_mirna_prediction mature_miRNA 9294102 9294123 . + . ID=nfu_pred_764;biotype=5'-mature-miRNA;gene=mir-338;geneid=nfu_pred_764_mir-338;coords=sgr18:9294102-9294123

sgr18 nfu_mirna_prediction mature_miRNA 9294140 9294161 . + . ID=nfu_pred_764;biotype=3'-mature-miRNA;gene=mir-338;geneid=nfu_pred_764_mir-338;coords=sgr18:9294140-9294161

sgr18 nfu_mirna_prediction pre_miRNA 10688820 10688917 . + . ID=nfu_pred_765;biotype=pre-miRNA;gene=mir-676;geneid=nfu_pred_765_mir-676;coords=sgr18:10688820-10688917

sgr18 nfu_mirna_prediction mature_miRNA 10688839 10688860 . + . ID=nfu_pred_765;biotype=5'-mature-miRNA;gene=mir-676;geneid=nfu_pred_765_mir-676;coords=sgr18:10688839-10688860

sgr18 nfu_mirna_prediction mature_miRNA 10688880 10688901 . + . ID=nfu_pred_765;biotype=3'-mature-miRNA;gene=mir-676;geneid=nfu_pred_765_mir-676;coords=sgr18:10688880-10688901

sgr18 nfu_mirna_prediction pre_miRNA 11564930 11564979 . + . ID=nfu_pred_766;biotype=pre-miRNA;gene=mir-7399;geneid=nfu_pred_766_mir-7399;coords=sgr18:11564930-11564979

sgr18 nfu_mirna_prediction pre_miRNA 12476464 12476542 . - . ID=nfu_pred_767;biotype=pre-miRNA;gene=mir-202;geneid=nfu_pred_767_mir-202;coords=sgr18:12476464-12476542

sgr18 nfu_mirna_prediction mature_miRNA 12476474 12476495 . - . ID=nfu_pred_767;biotype=3'-mature-miRNA;gene=mir-202;geneid=nfu_pred_767_mir-202;coords=sgr18:12476474-12476495

sgr18 nfu_mirna_prediction pre_miRNA 18418730 18418823 . + . ID=nfu_pred_768;biotype=pre-miRNA;gene=mir-2196;geneid=nfu_pred_768_mir-2196;coords=sgr18:18418730-18418823

sgr18 nfu_mirna_prediction pre_miRNA 20202568 20202659 . - . ID=nfu_pred_769;biotype=pre-miRNA;gene=mir-458;geneid=nfu_pred_769_mir-458;coords=sgr18:20202568-20202659

sgr18 nfu_mirna_prediction pre_miRNA 22522745 22522821 . - . ID=nfu_pred_770;biotype=pre-miRNA;gene=;geneid=nfu_pred_770_;coords=sgr18:22522745-22522821

sgr18 nfu_mirna_prediction mature_miRNA 22522760 22522781 . - . ID=nfu_pred_770;biotype=3'-mature-miRNA;gene=;geneid=nfu_pred_770_;coords=sgr18:22522760-22522781

sgr18 nfu_mirna_prediction pre_miRNA 36412649 36412746 . + . ID=nfu_pred_771;biotype=pre-miRNA;gene=mir-6720;geneid=nfu_pred_771_mir-6720;coords=sgr18:36412649-36412746

sgr18 nfu_mirna_prediction pre_miRNA 36546212 36546311 . - . ID=nfu_pred_772;biotype=pre-miRNA;gene=;geneid=nfu_pred_772_;coords=sgr18:36546212-36546311

sgr18 nfu_mirna_prediction mature_miRNA 36546233 36546254 . - . ID=nfu_pred_772;biotype=3'-mature-miRNA;gene=;geneid=nfu_pred_772_;coords=sgr18:36546233-36546254

sgr18 nfu_mirna_prediction pre_miRNA 36883231 36883325 . - . ID=nfu_pred_773;biotype=pre-miRNA;gene=mir-338;geneid=nfu_pred_773_mir-338;coords=sgr18:36883231-36883325

sgr18 nfu_mirna_prediction mature_miRNA 36883248 36883268 . - . ID=nfu_pred_773;biotype=3'-mature-miRNA;gene=mir-338;geneid=nfu_pred_773_mir-338;coords=sgr18:36883248-36883268

sgr18 nfu_mirna_prediction pre_miRNA 38103806 38103885 . - . ID=nfu_pred_774;biotype=pre-miRNA;gene=mir-934;geneid=nfu_pred_774_mir-934;coords=sgr18:38103806-38103885

sgr18 nfu_mirna_prediction pre_miRNA 38131125 38131227 . + . ID=nfu_pred_775;biotype=pre-miRNA;gene=mir-722;geneid=nfu_pred_775_mir-722;coords=sgr18:38131125-38131227

sgr18 nfu_mirna_prediction pre_miRNA 41685214 41685303 . + . ID=nfu_pred_776;biotype=pre-miRNA;gene=mir-672;geneid=nfu_pred_776_mir-672;coords=sgr18:41685214-41685303

sgr18 nfu_mirna_prediction pre_miRNA 42481862 42481954 . + . ID=nfu_pred_777;biotype=pre-miRNA;gene=mir-580;geneid=nfu_pred_777_mir-580;coords=sgr18:42481862-42481954

sgr18 nfu_mirna_prediction mature_miRNA 42481875 42481896 . + . ID=nfu_pred_777;biotype=5'-mature-miRNA;gene=mir-580;geneid=nfu_pred_777_mir-580;coords=sgr18:42481875-42481896

sgr19 nfu_mirna_prediction pre_miRNA 462621 462722 . + . ID=nfu_pred_778;biotype=pre-miRNA;gene=mir-770;geneid=nfu_pred_778_mir-770;coords=sgr19:462621-462722

sgr19 nfu_mirna_prediction pre_miRNA 1287545 1287623 . + . ID=nfu_pred_779;biotype=pre-miRNA;gene=;geneid=nfu_pred_779_;coords=sgr19:1287545-1287623

sgr19 nfu_mirna_prediction mature_miRNA 1287552 1287574 . + . ID=nfu_pred_779;biotype=5'-mature-miRNA;gene=;geneid=nfu_pred_779_;coords=sgr19:1287552-1287574

sgr19 nfu_mirna_prediction mature_miRNA 1287592 1287613 . + . ID=nfu_pred_779;biotype=3'-mature-miRNA;gene=;geneid=nfu_pred_779_;coords=sgr19:1287592-1287613

sgr19 nfu_mirna_prediction pre_miRNA 1785872 1785977 . + . ID=nfu_pred_780;biotype=pre-miRNA;gene=mir-214;geneid=nfu_pred_780_mir-214;coords=sgr19:1785872-1785977

sgr19 nfu_mirna_prediction mature_miRNA 1785898 1785920 . + . ID=nfu_pred_780;biotype=5'-mature-miRNA;gene=mir-214;geneid=nfu_pred_780_mir-214;coords=sgr19:1785898-1785920

sgr19 nfu_mirna_prediction mature_miRNA 1785935 1785950 . + . ID=nfu_pred_780;biotype=3'-mature-miRNA;gene=mir-214;geneid=nfu_pred_780_mir-214;coords=sgr19:1785935-1785950

sgr19 nfu_mirna_prediction pre_miRNA 1948371 1948468 . + . ID=nfu_pred_781;biotype=pre-miRNA;gene=mir-6720;geneid=nfu_pred_781_mir-6720;coords=sgr19:1948371-1948468

sgr19 nfu_mirna_prediction pre_miRNA 1970223 1970320 . + . ID=nfu_pred_782;biotype=pre-miRNA;gene=mir-6720;geneid=nfu_pred_782_mir-6720;coords=sgr19:1970223-1970320

sgr19 nfu_mirna_prediction pre_miRNA 1975220 1975317 . + . ID=nfu_pred_783;biotype=pre-miRNA;gene=mir-6720;geneid=nfu_pred_783_mir-6720;coords=sgr19:1975220-1975317

sgr19 nfu_mirna_prediction pre_miRNA 3964280 3964361 . + . ID=nfu_pred_784;biotype=pre-miRNA;gene=mir-122;geneid=nfu_pred_784_mir-122;coords=sgr19:3964280-3964361

sgr19 nfu_mirna_prediction mature_miRNA 3964292 3964313 . + . ID=nfu_pred_784;biotype=5'-mature-miRNA;gene=mir-122;geneid=nfu_pred_784_mir-122;coords=sgr19:3964292-3964313

sgr19 nfu_mirna_prediction mature_miRNA 3964331 3964351 . + . ID=nfu_pred_784;biotype=3'-mature-miRNA;gene=mir-122;geneid=nfu_pred_784_mir-122;coords=sgr19:3964331-3964351

sgr19 nfu_mirna_prediction pre_miRNA 7744627 7744756 . + . ID=nfu_pred_785;biotype=pre-miRNA;gene=mir-458;geneid=nfu_pred_785_mir-458;coords=sgr19:7744627-7744756

sgr19 nfu_mirna_prediction pre_miRNA 8159778 8159879 . - . ID=nfu_pred_786;biotype=pre-miRNA;gene=mir-577;geneid=nfu_pred_786_mir-577;coords=sgr19:8159778-8159879

sgr19 nfu_mirna_prediction mature_miRNA 8159798 8159818 . - . ID=nfu_pred_786;biotype=3'-mature-miRNA;gene=mir-577;geneid=nfu_pred_786_mir-577;coords=sgr19:8159798-8159818

sgr19 nfu_mirna_prediction pre_miRNA 11408966 11409060 . + . ID=nfu_pred_788;biotype=pre-miRNA;gene=mir-204;geneid=nfu_pred_788_mir-204;coords=sgr19:11408966-11409060

sgr19 nfu_mirna_prediction mature_miRNA 11408985 11409006 . + . ID=nfu_pred_788;biotype=5'-mature-miRNA;gene=mir-204;geneid=nfu_pred_788_mir-204;coords=sgr19:11408985-11409006

sgr19 nfu_mirna_prediction mature_miRNA 11409023 11409045 . + . ID=nfu_pred_788;biotype=3'-mature-miRNA;gene=mir-204;geneid=nfu_pred_788_mir-204;coords=sgr19:11409023-11409045

sgr19 nfu_mirna_prediction pre_miRNA 11555606 11555707 . - . ID=nfu_pred_789;biotype=pre-miRNA;gene=mir-101;geneid=nfu_pred_789_mir-101;coords=sgr19:11555606-11555707

sgr19 nfu_mirna_prediction mature_miRNA 11555628 11555648 . - . ID=nfu_pred_789;biotype=5'-mature-miRNA;gene=mir-101;geneid=nfu_pred_789_mir-101;coords=sgr19:11555628-11555648

sgr19 nfu_mirna_prediction mature_miRNA 11555664 11555684 . - . ID=nfu_pred_789;biotype=3'-mature-miRNA;gene=mir-101;geneid=nfu_pred_789_mir-101;coords=sgr19:11555664-11555684

sgr19 nfu_mirna_prediction pre_miRNA 14583339 14583419 . + . ID=nfu_pred_790;biotype=pre-miRNA;gene=mir-2973;geneid=nfu_pred_790_mir-2973;coords=sgr19:14583339-14583419

sgr19 nfu_mirna_prediction mature_miRNA 14583351 14583372 . + . ID=nfu_pred_790;biotype=5'-mature-miRNA;gene=mir-2973;geneid=nfu_pred_790_mir-2973;coords=sgr19:14583351-14583372

sgr19 nfu_mirna_prediction mature_miRNA 14583389 14583410 . + . ID=nfu_pred_790;biotype=3'-mature-miRNA;gene=mir-2973;geneid=nfu_pred_790_mir-2973;coords=sgr19:14583389-14583410

sgr19 nfu_mirna_prediction pre_miRNA 17634511 17634624 . + . ID=nfu_pred_791;biotype=pre-miRNA;gene=mir-181;geneid=nfu_pred_791_mir-181;coords=sgr19:17634511-17634624

sgr19 nfu_mirna_prediction mature_miRNA 17634542 17634563 . + . ID=nfu_pred_791;biotype=5'-mature-miRNA;gene=mir-181;geneid=nfu_pred_791_mir-181;coords=sgr19:17634542-17634563

sgr19 nfu_mirna_prediction mature_miRNA 17634584 17634605 . + . ID=nfu_pred_791;biotype=3'-mature-miRNA;gene=mir-181;geneid=nfu_pred_791_mir-181;coords=sgr19:17634584-17634605

sgr19 nfu_mirna_prediction pre_miRNA 17634759 17634839 . + . ID=nfu_pred_792;biotype=pre-miRNA;gene=mir-181;geneid=nfu_pred_792_mir-181;coords=sgr19:17634759-17634839

sgr19 nfu_mirna_prediction mature_miRNA 17634769 17634790 . + . ID=nfu_pred_792;biotype=5'-mature-miRNA;gene=mir-181;geneid=nfu_pred_792_mir-181;coords=sgr19:17634769-17634790

sgr19 nfu_mirna_prediction pre_miRNA 19173585 19173641 . + . ID=nfu_pred_793;biotype=pre-miRNA;gene=mir-7641;geneid=nfu_pred_793_mir-7641;coords=sgr19:19173585-19173641

sgr19 nfu_mirna_prediction pre_miRNA 19508671 19508756 . + . ID=nfu_pred_794;biotype=pre-miRNA;gene=mir-599;geneid=nfu_pred_794_mir-599;coords=sgr19:19508671-19508756

sgr19 nfu_mirna_prediction mature_miRNA 19508679 19508700 . + . ID=nfu_pred_794;biotype=5'-mature-miRNA;gene=mir-599;geneid=nfu_pred_794_mir-599;coords=sgr19:19508679-19508700

sgr19 nfu_mirna_prediction mature_miRNA 19508717 19508737 . + . ID=nfu_pred_794;biotype=3'-mature-miRNA;gene=mir-599;geneid=nfu_pred_794_mir-599;coords=sgr19:19508717-19508737

sgr19 nfu_mirna_prediction pre_miRNA 20468419 20468472 . - . ID=nfu_pred_795;biotype=pre-miRNA;gene=mir-7431;geneid=nfu_pred_795_mir-7431;coords=sgr19:20468419-20468472

sgr19 nfu_mirna_prediction pre_miRNA 20512905 20513049 . + . ID=nfu_pred_796;biotype=pre-miRNA;gene=mir-8994;geneid=nfu_pred_796_mir-8994;coords=sgr19:20512905-20513049

sgr19 nfu_mirna_prediction pre_miRNA 21791189 21791267 . + . ID=nfu_pred_797;biotype=pre-miRNA;gene=mir-551;geneid=nfu_pred_797_mir-551;coords=sgr19:21791189-21791267

sgr19 nfu_mirna_prediction mature_miRNA 21791199 21791219 . + . ID=nfu_pred_797;biotype=5'-mature-miRNA;gene=mir-551;geneid=nfu_pred_797_mir-551;coords=sgr19:21791199-21791219

sgr19 nfu_mirna_prediction pre_miRNA 22277681 22277766 . - . ID=nfu_pred_798;biotype=pre-miRNA;gene=mir-11;geneid=nfu_pred_798_mir-11;coords=sgr19:22277681-22277766

sgr19 nfu_mirna_prediction mature_miRNA 22277731 22277753 . - . ID=nfu_pred_798;biotype=5'-mature-miRNA;gene=mir-11;geneid=nfu_pred_798_mir-11;coords=sgr19:22277731-22277753

sgr19 nfu_mirna_prediction mature_miRNA 22277692 22277713 . - . ID=nfu_pred_798;biotype=3'-mature-miRNA;gene=mir-11;geneid=nfu_pred_798_mir-11;coords=sgr19:22277692-22277713

sgr19 nfu_mirna_prediction pre_miRNA 23393987 23394065 . - . ID=nfu_pred_799;biotype=pre-miRNA;gene=mir-24;geneid=nfu_pred_799_mir-24;coords=sgr19:23393987-23394065

sgr19 nfu_mirna_prediction mature_miRNA 23394035 23394055 . - . ID=nfu_pred_799;biotype=5'-mature-miRNA;gene=mir-24;geneid=nfu_pred_799_mir-24;coords=sgr19:23394035-23394055

sgr19 nfu_mirna_prediction mature_miRNA 23393997 23394018 . - . ID=nfu_pred_799;biotype=3'-mature-miRNA;gene=mir-24;geneid=nfu_pred_799_mir-24;coords=sgr19:23393997-23394018

sgr19 nfu_mirna_prediction pre_miRNA 23396107 23396205 . - . ID=nfu_pred_800;biotype=pre-miRNA;gene=mir-27;geneid=nfu_pred_800_mir-27;coords=sgr19:23396107-23396205

sgr19 nfu_mirna_prediction mature_miRNA 23396122 23396142 . - . ID=nfu_pred_800;biotype=3'-mature-miRNA;gene=mir-27;geneid=nfu_pred_800_mir-27;coords=sgr19:23396122-23396142

sgr19 nfu_mirna_prediction pre_miRNA 23396680 23396781 . - . ID=nfu_pred_801;biotype=pre-miRNA;gene=mir-23;geneid=nfu_pred_801_mir-23;coords=sgr19:23396680-23396781

sgr19 nfu_mirna_prediction mature_miRNA 23396742 23396762 . - . ID=nfu_pred_801;biotype=5'-mature-miRNA;gene=mir-23;geneid=nfu_pred_801_mir-23;coords=sgr19:23396742-23396762

sgr19 nfu_mirna_prediction mature_miRNA 23396700 23396720 . - . ID=nfu_pred_801;biotype=3'-mature-miRNA;gene=mir-23;geneid=nfu_pred_801_mir-23;coords=sgr19:23396700-23396720

sgr19 nfu_mirna_prediction pre_miRNA 23849733 23849798 . - . ID=nfu_pred_802;biotype=pre-miRNA;gene=mir-8987;geneid=nfu_pred_802_mir-8987;coords=sgr19:23849733-23849798

sgr19 nfu_mirna_prediction pre_miRNA 25042280 25042358 . + . ID=nfu_pred_803;biotype=pre-miRNA;gene=mir-939;geneid=nfu_pred_803_mir-939;coords=sgr19:25042280-25042358

sgr19 nfu_mirna_prediction mature_miRNA 25042329 25042348 . + . ID=nfu_pred_803;biotype=5'-mature-miRNA;gene=mir-939;geneid=nfu_pred_803_mir-939;coords=sgr19:25042329-25042348

sgr19 nfu_mirna_prediction mature_miRNA 25042288 25042308 . + . ID=nfu_pred_803;biotype=3'-mature-miRNA;gene=mir-939;geneid=nfu_pred_803_mir-939;coords=sgr19:25042288-25042308

sgr19 nfu_mirna_prediction pre_miRNA 26281665 26281761 . - . ID=nfu_pred_804;biotype=pre-miRNA;gene=;geneid=nfu_pred_804_;coords=sgr19:26281665-26281761

sgr19 nfu_mirna_prediction mature_miRNA 26281720 26281740 . - . ID=nfu_pred_804;biotype=5'-mature-miRNA;gene=;geneid=nfu_pred_804_;coords=sgr19:26281720-26281740

sgr19 nfu_mirna_prediction mature_miRNA 26281677 26281694 . - . ID=nfu_pred_804;biotype=3'-mature-miRNA;gene=;geneid=nfu_pred_804_;coords=sgr19:26281677-26281694

sgr19 nfu_mirna_prediction pre_miRNA 26980241 26980338 . - . ID=nfu_pred_805;biotype=pre-miRNA;gene=mir-6720;geneid=nfu_pred_805_mir-6720;coords=sgr19:26980241-26980338

sgr19 nfu_mirna_prediction pre_miRNA 26986632 26986729 . - . ID=nfu_pred_806;biotype=pre-miRNA;gene=mir-6720;geneid=nfu_pred_806_mir-6720;coords=sgr19:26986632-26986729

sgr19 nfu_mirna_prediction pre_miRNA 31412558 31412654 . - . ID=nfu_pred_807;biotype=pre-miRNA;gene=mir-338;geneid=nfu_pred_807_mir-338;coords=sgr19:31412558-31412654

sgr19 nfu_mirna_prediction pre_miRNA 35257869 35257931 . + . ID=nfu_pred_808;biotype=pre-miRNA;gene=;geneid=nfu_pred_808_;coords=sgr19:35257869-35257931

sgr19 nfu_mirna_prediction pre_miRNA 36828364 36828473 . + . ID=nfu_pred_809;biotype=pre-miRNA;gene=mir-6698;geneid=nfu_pred_809_mir-6698;coords=sgr19:36828364-36828473

sgr19 nfu_mirna_prediction pre_miRNA 38259840 38259939 . + . ID=nfu_pred_810;biotype=pre-miRNA;gene=mir-199;geneid=nfu_pred_810_mir-199;coords=sgr19:38259840-38259939

sgr19 nfu_mirna_prediction mature_miRNA 38259860 38259882 . + . ID=nfu_pred_810;biotype=5'-mature-miRNA;gene=mir-199;geneid=nfu_pred_810_mir-199;coords=sgr19:38259860-38259882

sgr19 nfu_mirna_prediction mature_miRNA 38259899 38259919 . + . ID=nfu_pred_810;biotype=3'-mature-miRNA;gene=mir-199;geneid=nfu_pred_810_mir-199;coords=sgr19:38259899-38259919

sgr19 nfu_mirna_prediction pre_miRNA 38262154 38262266 . + . ID=nfu_pred_811;biotype=pre-miRNA;gene=mir-214;geneid=nfu_pred_811_mir-;coords=sgr19:38262154-38262266

sgr19 nfu_mirna_prediction mature_miRNA 38262183 38262204 . + . ID=nfu_pred_811;biotype=5'-mature-miRNA;gene=mir-214;geneid=nfu_pred_811_mir-214;coords=sgr19:38262183-38262204

sgr19 nfu_mirna_prediction mature_miRNA 38262221 38262242 . + . ID=nfu_pred_811;biotype=3'-mature-miRNA;gene=mir-214;geneid=nfu_pred_811_mir-214;coords=sgr19:38262221-38262242

superscaffold00092 nfu_mirna_prediction pre_miRNA 708704 708826 . + . ID=nfu_pred_812;biotype=pre-miRNA;gene=mir-8910;geneid=nfu_pred_812_mir-8910;coords=superscaffold00092:708704-708826

superscaffold00093 nfu_mirna_prediction pre_miRNA 45478 45565 . + . ID=nfu_pred_813;biotype=pre-miRNA;gene=mir-187;geneid=nfu_pred_813_mir-187;coords=superscaffold00093:45478-45565

superscaffold00093 nfu_mirna_prediction mature_miRNA 45485 45506 . + . ID=nfu_pred_813;biotype=5'-mature-miRNA;gene=mir-187;geneid=nfu_pred_813_mir-187;coords=superscaffold00093:45485-45506

superscaffold00093 nfu_mirna_prediction mature_miRNA 45523 45544 . + . ID=nfu_pred_813;biotype=3'-mature-miRNA;gene=mir-187;geneid=nfu_pred_813_mir-187;coords=superscaffold00093:45523-45544

superscaffold00093 nfu_mirna_prediction pre_miRNA 208503 208599 . + . ID=nfu_pred_814;biotype=pre-miRNA;gene=;geneid=nfu_pred_814_;coords=superscaffold00093:208503-208599

superscaffold00093 nfu_mirna_prediction mature_miRNA 208519 208541 . + . ID=nfu_pred_814;biotype=5'-mature-miRNA;gene=;geneid=nfu_pred_814_;coords=superscaffold00093:208519-208541

superscaffold00093 nfu_mirna_prediction mature_miRNA 208557 208578 . + . ID=nfu_pred_814;biotype=3'-mature-miRNA;gene=;geneid=nfu_pred_814_;coords=superscaffold00093:208557-208578
